# Supplementary material for: An updated gene regulatory network reconstruction of multidrug-resistant Pseudomonas aeruginosa CCBH4851
Source: Mem Inst Oswaldo Cruz. 2022 Oct 14;117:e220111. doi: 10.1590/0074-02760220111 (PMC9565603; doi:10.1590/0074-02760220111)
Supplement: Supplementary file 1 [file 1678-8060-mioc-117-e220111-s.pdf]

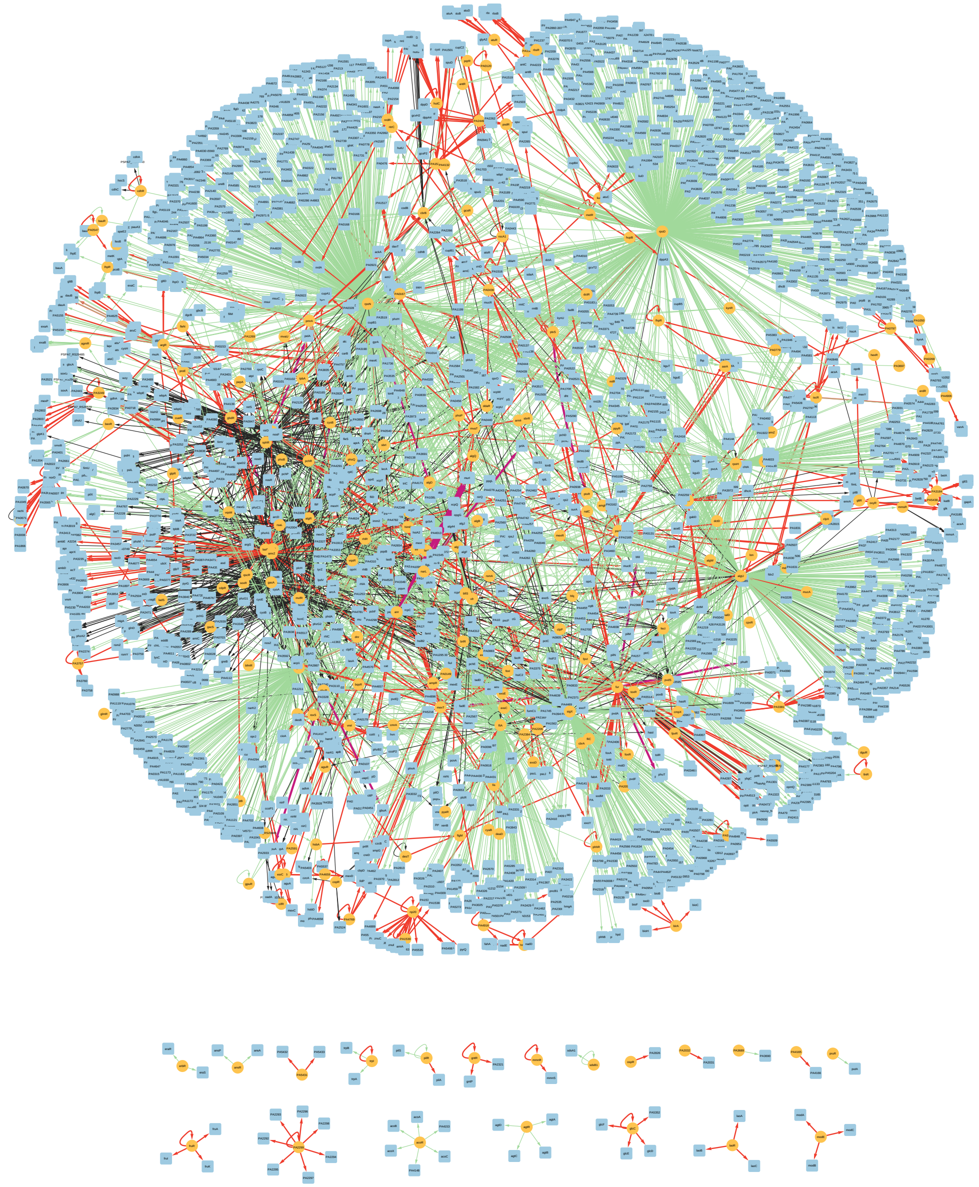

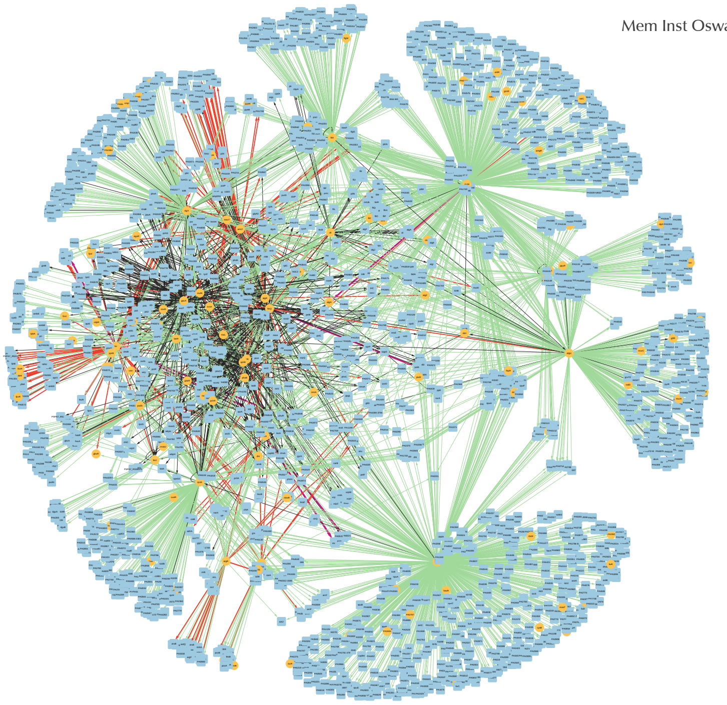

TABLE I

Regulatory genes that are transcriptional factors in CCBH-2022

TFs CCBH-2022	
AL347_16995	transcriptional regulator
acoR (AL347_21955)	transcriptional regulator AcoR
lasR (AL347_05615)	transcriptional regulator LasR
mexT (AL347_32559)	transcriptional regulator MexT
rhlR (AL347_25325)	transcriptional regulator RhlR
fleQ (AL347_07690)	transcriptional regulator FleQ
pchR (AL347_21535)	transcriptional regulator PchR
soxR (AL347_00695)	redox-sensitive transcriptional activator SoxR
AL347_16375	transcriptional regulator
anr (AL347_05030)	transcriptional regulator Anr
aguR (AL347_19255)	transcriptional regulator AguR
mvfR (AL347_08190)	transcriptional regulator MvfR
AL347_01365	TetR/AcrR family transcriptional regulator
AL347_05155	transcriptional regulator
AL347_30475	transcriptional regulator
cysB (AL347_03920)	transcriptional regulator CysB
ampR (AL347_22150)	transcriptional regulator AmpR
psrA (AL347_27830)	transcriptional regulator PsrA
argR (AL347_09030)	transcriptional regulator ArgR
np20 (AL347_17315)	transcriptional regulator
AL347_30490	transcriptional regulator
dnr (AL347_20470)	transcriptional regulator Dnr
AL347_32110	transcriptional regulator
mvaT (AL347_10420)	transcriptional regulator MvaT
narL (AL347_23345)	transcriptional regulator NarL
nalC (AL347_24170)	transcriptional regulator
metR (AL347_24850)	transcriptional regulator MetR
AL347_14000	transcriptional regulator
AL347_18440	HTH-type transcriptional activator BauR
betI (AL347_16655)	BetI family transcriptional regulator
cdhR (AL347_16735)	CdhR family transcriptional regulator
AL347_20000	transcriptional regulator
AL347_07225	transcriptional regulator

AL347_18375	transcriptional regulator
AL347_07150	transcriptional regulator
desT (AL347_14060)	transcriptional regulator
AL347_15075	transcriptional regulator
dhcR (AL347_02620)	transcriptional regulator DhcR
phhR (AL347_09140)	transcriptional regulator PhhR
AL347_11360	transcriptional regulator
AL347_26900	HTH-type transcriptional regulator
AL347_23235	transcriptional regulator
exsA (AL347_04135)	exoenzyme S transcriptional regulator ExsA
fis (AL347_13875)	Fis family transcriptional regulator
AL347_02560	transcriptional regulator
iscR (AL347_23680)	HTH-type transcriptional regulator
AL347_01520	transcriptional regulator
AL347_30085	anaerobic nitric oxide reductase transcriptional regulator
AL347_25740	transcriptional regulator
fruR (AL347_24990)	FruR family transcriptional regulator
ptxS (AL347_00765)	transcriptional regulator PtxS
AL347_32765	transcriptional regulator
glcC (AL347_16540)	DNA-binding transcriptional regulator GlcC
glmR (AL347_17570)	GlmR transcriptional regulator
gntR (AL347_33430)	GntR family transcriptional regulator
gpuR (AL347_19230)	transcriptional activator GpuR
oxyR (AL347_16475)	transcriptional regulator
AL347_02170	transcriptional regulator
AL347_19640	transcriptional regulator
AL347_06815	transcriptional regulator
AL347_13440	transcriptional regulator
AL347_20580	transcriptional regulator
nalD (AL347_24930)	transcriptional regulator
AL347_12475	transcriptional regulator
nfxB (AL347_12495)	transcriptional regulator NfxB
AL347_02370	transcriptional regulator
mmsR (AL347_24945)	transcriptional regulator MmsR
toxR (AL347_10010)	transcriptional regulator ToxR
AL347_16985	transcriptional regulator

nrdR (AL347_22425)	transcriptional regulator NrdR
AL347_19125	transcriptional regulator
AL347_09525	transcriptional regulator
AL347_08505	transcriptional regulator
AL347_00275	transcriptional regulator
AL347_06765	transcriptional regulator
AL347_05240	transcriptional regulator
AL347_02445	transcriptional regulator
AL347_33530	transcriptional regulator
ospR (AL347_28765)	transcriptional regulator
AL347_25825	transcriptional regulator
AL347_24330	transcriptional regulator
AL347_23985	transcriptional regulator
AL347_12815	transcriptional regulator
AL347_14140	transcriptional regulator
AL347_16955	transcriptional regulator
AL347_09825	transcriptional regulator
trpI (AL347_17940)	HTH-type transcriptional regulator TrpI

TABLE II

Regulatory genes that are sigma factors in CCBH-2022

Sigma Factors CCBH-2022	
AL347_18525	ECF subfamily sigma-70 factor
rpoH (AL347_19690)	RNA polymerase sigma factor RpoH
rpoD (AL347_20760)	RNA polymerase sigma factor RpoD
AL347_23230	RNA polymerase sigma factor
rpoS (AL347_24665)	RNA polymerase sigma factor RpoS
AL347_28405	RNA polymerase sigma factor
foxI(AL347_32680)	ECF sigma factor FoxI
foxR (AL347_32685)	anti-sigma factor FoxR
pvdS (AL347_32895)	extracytoplasmic-function sigma-70 factor
fpvI (AL347_33080)	RNA polymerase sigma factor
AL347_02355	RNA polymerase sigma factor
sigX (AL347_03799)	RNA polymerase sigma factor SigX
fliA (AL347_05490)	flagellar biosynthesis sigma factor FliA
mucB (AL347_09695)	sigma factor AlgU regulator MucB
mucA (AL347_09700)	sigma factor AlgU negative regulator MucA
algU (AL347_09705)	RNA polymerase sigma factor AlgU
vreI (AL347_10175)	ECF sigma factor VreI
rpoN (AL347_11175)	RNA polymerase factor sigma-54
algQ (AL347_16020)	anti-RNA polymerase sigma 70 factor

	RNAs as well

TABLE III

Regulatory genes that are RNAs in CCBH-2022

RNAs CCBH-2022	
sigX (AL347_03799)	RNA polymerase sigma factor SigX
rpoH (AL347_19690)	RNA polymerase sigma factor RpoH
rpoD (AL347_20760)	RNA polymerase sigma factor RpoD
rpoN (AL347_11175)	RNA polymerase factor sigma-54
rpoS (AL347_24665)	RNA polymerase sigma factor RpoS
algU (AL347_09705)	RNA polymerase sigma factor AlgU
AL347_28405	RNA polymerase sigma factor
AL347_23230	RNA polymerase sigma factor
AL347_28690	ATP-dependent RNA helicase
hfq (AL347_14340)	RNA-binding protein Hfq
fpvI (AL347_33080)	RNA polymerase sigma factor
algQ (AL347_16020)	anti-RNA polymerase sigma 70 factor
AL347_02355	RNA polymerase sigma factor

	Sigma Factors as well

```
################################################################################
#
# Study of the basic topology of the Pseudomonas aeruginosa Gene Regulation Network
#
################################################################################


library(dplyr)
library(tibble)
library(readr)
library(igraph)
library(scales)


dados <-
  read_csv2("GRN.csv")


# Pre-processing data
c1 <-
  dados$`Regulator (TF or sigma)` %>%
  strsplit(" ") %>%
  unlist()

c1.TF <- c1[gtools::odd(1:length(c1))]
c1.ortologo.TF <- c1[gtools::even(1:length(c1))]

dados$`Regulator (TF)` <- c1.TF
dados$`orthologs of TF` <- c1.ortologo.TF


nrow(dados)  ==
  sum(paste(dados$`Regulator (TF)`,dados$`orthologs of TF`) == dados$`Regulator (TF or
sigma)`)
```

```
dados <-
  dados %>%
  select(`Regulator (TF)`,
         `Target gene`,
         `mode of regulation`,
         `orthologs of TF`,
         `Ortholog of the target gene`
  )

dados$`orthologs of TF` <- gsub("\\(|\\)", "",
                              dados$`orthologs of TF`)

rm(c1, c1.ortologo.TF,c1.TF)

auxTF <- dados[,c(1,4)] %>% setNames(c("gene_CCBH4851", "orthologs"))
auxTarget <- dados[,c(2,5)] %>% setNames(c("gene_CCBH4851", "orthologs"))

vert <-
  dplyr::union(auxTF,auxTarget) %>%
  filter(!is.na(gene_CCBH4851) )

vert$rotulo <- paste0(vert$gene_CCBH4851,
                      " (",
                      vert$orthologs,
                      ")")

arestas <-
  dados[,c(1,2,3)] %>%
  filter(!is.na(`Target gene`))


# GRN with Igraph

Rede <- graph_from_data_frame(d = arestas,
                             directed = TRUE
                             # vertices = vert
```

```
)


# Exploring the Network

V(Rede)$color <- ifelse(V(Rede)$name %in% auxTF$gene_CCBH4851,
                        "yellow", "lightblue")

codificacao <- "'+' = 'green' ; '-' = 'red' ; '?' = 'black' ; 'd' = 'darkgrey'"
E(Rede)$color <- car::Recode(E(Rede)$`mode of regulation`,
                             codificacao)


V(Rede)$size <- 2+ log(1+degree(Rede, mode = "out"))

V(Rede)$name <- V(Rede)$rotulo


# Topology

V(Rede)$name <- vert$gene_CCBH4851


nrow(vert)

nrow(arestas)

sum(V(Rede)$color == "yellow")

sum(V(Rede)$color == "lightblue")

scientific(graph.density(Rede, loops=TRUE))


p.kin <- degree_distribution(Rede, mode="in")
p.kin.na <- ifelse(p.kin == 0,NA,p.kin)
```

```
min.kin <- min(degree(Rede, mode="in"))
max.kin <-  max(degree(Rede, mode="in"))
plot(min.kin:max.kin, p.kin.na,
     xlab= "k-in (degree input)", ylab= "P(k-in)", type="h")
title(sub="Figure 3: Distribution of Degree k-in",
      cex.sub = 0.75, font.sub = 3, col.sub = "black")

### Power Law ajusts kin

x.in <- log10(min.kin:max.kin)
y.in <- log10(p.kin.na)
data.in <-
  data.frame(X=x.in,Y=y.in) %>%
  filter(!is.na(X) & !is.na(Y) & X != -Inf)
ajuste.in <- lm(Y~X,data=data.in)
log.A.in <- ajuste.in$coefficients[1]
A.in <- 10^(log.A.in)
gama.in <- -ajuste.in$coefficients[2]
#
plot(x.in, y.in,
     xlab= "log(k-in)", ylab= "log P(k-in)")
plotrix::ablineclip(log.A.in, -gama.in, x1= 0,x2=log10(max.kin))


# output
p.kout <- degree_distribution(Rede, mode="out")
p.kout.na <- ifelse(p.kout == 0,NA,p.kout)
min.kout <- min(degree(Rede, mode="out"))
max.kout <- max(degree(Rede, mode="out"))

plot(min.kout:max.kout, p.kout.na,
     xlab= "k-out (degree output)", ylab= "P(k-out)", type="h")
title(sub="Figure 3: Distribution of degree k-out",
      cex.sub = 0.75, font.sub = 3, col.sub = "black")

# Axis y in log10
```

```
plot(min.kout:max.kout, p.kout.na,
     xlab= "k-out (degree output)", ylab= "P(k-out)", type="h",log="y")
title(sub="Figure 4: Distribution of Degree k-out",
      cex.sub = 0.75, font.sub = 3, col.sub = "black")


# Power Law ajusts kout
x.out <- log10(min.kout:max.kout)
y.out <- log10(p.kout.na)
data.out <-
  data.frame(X=x.out,Y=y.out) %>%
  filter(!is.na(X) & !is.na(Y) & X != -Inf)
ajuste.out <- lm(Y~X,data=data.out)
log.A.out <- ajuste.out$coefficients[1]
A.out <- 10^(log.A.out)
gama.out <- -ajuste.out$coefficients[2]
#
plot(x.out, y.out,
     xlab= "log(k-out)", ylab= "log P(k-out)")
title(sub="Figure 5: Distribution of Degree k-out log-log",
      cex.sub = 0.75, font.sub = 3, col.sub = "black")
plotrix::ablineclip(log.A.out, -gama.out, x1= 0,x2=log10(max.kout))



# Clustering coefficient


# Global
CoeffCluster.global <- scientific(transitivity(Rede,type="globalundirected"))

# Mean
CoeffCluster.medio <- scientific(transitivity(Rede,type="average"))

# Local
CoeffCluster.i <- transitivity(Rede,type="localundirected",
```

```
                    isolates = "NaN",
                    vids = NULL,
                    weights = NULL)

# Total distribution
hist(CoeffCluster.i,
    xlab= "Local Clustering Coefficient", ylab= "Frequency", main=NULL)
title(sub="Figure 6: Distribution of Total Clustering Coefficient",
     cex.sub = 0.75, font.sub = 3, col.sub = "black")

# Proportion of vertices with zero coef
propCzero <-table(CoeffCluster.i)[1]/nrow(vert)



# Proportion of vertices with coef 1:
propChum <-table(CoeffCluster.i)[nrow(table(CoeffCluster.i))]/nrow(vert)



# Distribution without nulls and without 1
hist(ifelse(CoeffCluster.i ==0 | CoeffCluster.i ==1, NA, CoeffCluster.i),
    xlab= "Local Clustering Coefficient", ylab= "Frequency", main=NULL)
title(sub="Figure 2: Distribution of Partial Clustering Coefficient",
     cex.sub = 0.75, font.sub = 3, col.sub = "black")

################
# Scatter plot : clustering coefficient by degree
k.i <- degree(Rede,mode="all")

C.k.i <- CoeffCluster.i
#
plot(k.i, C.k.i,
    xlab="k (degree total)", ylab= "C(k)")
title(sub="Figure 7: Clustering Coefficient by Degree",
     cex.sub = 0.75, font.sub = 3, col.sub = "black")

# k =0 ou k=1 absent
```

```
C.k.i.filtrado <- transitivity(Rede,type="localundirected",
                              isolates = "NaN")

plot(k.i, C.k.i.filtrado,
     xlab="k (degree total)", ylab= "C(k)")
title(sub="Figure 7: Clustering Coefficient by Degree",
      cex.sub = 0.75, font.sub = 3, col.sub = "black")

##################################

# Connectivity

# Clusters
count_components(Rede)


components(Rede)$csize

# Histogram
plot(components(Rede)$csize)

reguladores.por.grupo <- c()
for(i in 1:count_components(Rede)){
  grupo <- names(components(Rede)$membership)[components(Rede)$membership == i]
  reguladores.por.grupo<-
    c(reguladores.por.grupo,
      sum(grupo %in% auxTF$gene_CCBH4851))
}

###

propor.reguladores.por.grupo <- reguladores.por.grupo/components(Rede)$csize

###
probab.n.reguladores.por.grupo <-
  table(reguladores.por.grupo)/count_components(Rede)
```

```
###
plot(probab.n.reguladores.por.grupo,
     log= "xy",
     ylim=c(0.01,1))


####################################################

# Dominant activity study: activation or non-activation

tab.modoRegula <-
  table(arestas$`mode of regulation`)
tab.modoRegula <- tab.modoRegula[order(tab.modoRegula)]



#
auto_regul <-
  filter(arestas, `Regulator (TF)` == `Target gene`)
#
tab.modo.Auto_regul <-
  table(auto_regul$`mode of regulation`)
tab.modo.Auto_regul <- tab.modo.Auto_regul[order(tab.modo.Auto_regul)]

#####################
# Path Lengths

### The diameter of a graph is the length of the longest geodesic.
diameter(Rede,directed = TRUE,unconnected = TRUE)
diameter(Rede,directed = FALSE,unconnected = TRUE)



### The average path size:
mean_distance(Rede, directed = TRUE, unconnected = TRUE)
mean_distance(Rede, directed = FALSE, unconnected = TRUE)
```

```
####################
# 3 vertices motifs



triad_census(Rede)
triad_census(Rede)[9]
triad_census(Rede)[13]



Rede2 <- graph_from_data_frame(d = filter(arestas,
                                   `mode of regulation` == "+" |
                                    `mode of regulation` == "-"))
los  "+" e "-" somente
triad_census(Rede2)
triad_census(Rede2)[9]
triad_census(Rede2)[13]

Rede3 <- graph_from_data_frame(d = filter(arestas,
                                   `mode of regulation` == "+" )
                          # directed = TRUE, vertices = vert
)

triad_census(Rede3)
triad_census(Rede3)[9]
triad_census(Rede3)[13]



Rede4 <- graph_from_data_frame(d = filter(arestas,
                                   `mode of regulation` == "-" ))

triad_census(Rede4)
triad_census(Rede4)[9]
triad_census(Rede4)[13]

####################
```

```
# Motifs of 2 vertices

dyad_census(Rede)
dyad_census(Rede2)
dyad_census(Rede3)

#####
V(Rede)$name <- V(Rede)$rotulo

# Kleinberg's hub centrality scores
# ref: https://www.sci.unich.it/~francesc/teaching/network/kleinberg.html

hubs.em.ordem.dec <-(hub_score(Rede)$vector)[order((hub_score(Rede)$vector),
                                                   decreasing = TRUE)]

authority.em.ordem.dec <-
  authority_score(Rede)$vector[order((authority_score(Rede)$vector),
                                     decreasing = TRUE)]



# top 10 (Kleinberg's hub centrality scores)
hubs.em.ordem.dec[1:30]

# top 10 (Kleinberg's authority centrality scores)
authority.em.ordem.dec[1:10]

## using  k-out
k.hubs.em.ordem.dec <- degree(Rede, mode="out")[order(degree(Rede, mode="out"),
                                                      decreasing = TRUE)]

# top 10 k-out
k.hubs.em.ordem.dec[1:30]
```

```
Usage = """RBH BLASTOUTPUT1 BLASTOUTPUT2 RBH-list-outfile """

import sys, re

if len(sys.argv) < 3:
  print(Usage)

debug = 9

infl1 = sys.argv[1]
infl2 = sys.argv[2]
outfile = sys.argv[3]

#parse first BLAST results
FL1 = open(infl1, 'r')
D1 = {} #dictionary for BLAST file ONE
for Line in FL1:
  if ( Line[0] != '#' ):
    Line.strip()
    Elements = re.split('\t', Line)
    queryId = Elements[0]
    subjectId = Elements[1]
    if ( not ( queryId in D1.keys() ) ):
      D1[queryId] = subjectId #pick the first hit

if (debug): D1.keys()

#parse second BLAST results
FL2 = open(infl2, 'r')
D2 = {}
for Line in FL2:
  if ( Line[0] != '#' ):
    Line.strip()
    Elements = re.split('\t', Line)
    queryId = Elements[0]
```

```
    subjectId = Elements[1]
    if ( not ( queryId in D2.keys() ) ):
      D2[queryId] = subjectId #pick the first hit

if (debug): D2.keys()

#Now, pick the share pairs
SharedPairs={}
for id1 in D1.keys():
  value1 = D1[id1]
  if ( value1 in D2.keys() ):
    if ( id1 == D2[value1] ) : #a shared best reciprocal pair
      SharedPairs[value1] = id1

if (debug): SharedPairs

#outfl = open("_out.csv", "w")
outfl = open( outfile, 'w')

for k1 in SharedPairs.keys():
  line = k1 + '\t' + SharedPairs[k1] + '\n'
  outfl.write(line)

outfl.close()

print("Done. RBH from", sys.argv[1], "and", sys.argv[2], "are in", sys.argv[3])
```

Exclusive *vfr* interactions in CCBH-2022					
**Regulatory gene**	**Ortholog of the regulatory gene**	**Target gene**	**Ortholog of the target gene**	**Mode of regulation**	**Reference (PubMed)**
vfr (AL347_21140)	vfr	pilP (AL347_14850)	pilP	?	27242034*
vfr (AL347_21140)	vfr	plcH (AL347_09290)	plcH	?	27242034*
vfr (AL347_21140)	vfr	PA0653 (AL347_21145)	PA0653	?	27242034*
vfr (AL347_21140)	vfr	plcN (AL347_26160)	plcN	?	27242034*
vfr (AL347_21140)	vfr	plcR (AL347_09295)	plcR	?	27242034*
vfr (AL347_21140)	vfr	pilM (AL347_14865)	pilM	?	27242034*
vfr (AL347_21140)	vfr	pilO (AL347_14855)	pilO	?	27242034*
vfr (AL347_21140)	vfr	pilN (AL347_14860)	pilN	?	27242034*
vfr (AL347_21140)	vfr	alg8 (AL347_25105)	alg8	?	27242034*
vfr (AL347_21140)	vfr	algZ (AL347_16055)	algZ	?	27242034*
vfr (AL347_21140)	vfr	alg44 (AL347_25100)	alg44	?	27242034*
vfr (AL347_21140)	vfr	algJ (AL347_25060)	algJ	?	27242034*
vfr (AL347_21140)	vfr	algK (AL347_25095)	algK	?	27242034*
vfr (AL347_21140)	vfr	algI (AL347_25065)	algI	?	27242034*
vfr (AL347_21140)	vfr	algL (AL347_25075)	algL	?	27242034*
vfr (AL347_21140)	vfr	lasI (AL347_05605)	lasI	?	27242034*
vfr (AL347_21140)	vfr	algA (AL347_25050)	algA	?	27242034*
vfr (AL347_21140)	vfr	algF (AL347_25055)	algF	?	27242034*
vfr (AL347_21140)	vfr	algG (AL347_25085)	algG	?	27242034*
vfr (AL347_21140)	vfr	algD (AL347_25110)	algD	?	27242034*
vfr (AL347_21140)	vfr	algE (AL347_25090)	algE	?	27242034*
vfr (AL347_21140)	vfr	exoT (AL347_17990)	exoT	?	27242034*
vfr (AL347_21140)	vfr	argH (AL347_16060)	argH	?	27242034*
vfr (AL347_21140)	vfr	pbpG (AL347_09160)	pbpG	?	27242034*
vfr (AL347_21140)	vfr	algX (AL347_25080)	algX	?	27242034*

Exclusive *rsaL* interactions in CCBH-2022					
**Regulatory gene**	**Ortholog of the regulatory gene**	**Target gene**	**Ortholog of the target gene**	**Mode of regulation**	**Reference (PubMed)**
rsaL (AL347_05610)	rsaL	lasI (AL347_05605)	lasI	-	27242034*, 18974177*, 22587778
rsaL (AL347_05610)	rsaL	phzA1 (AL347_21625)	phzA1	-	27242034*, 18974177*, 22587778
rsaL (AL347_05610)	rsaL	lasB (AL347_24155)	lasB	?	27242034*, 18974177*, 22587778
rsaL (AL347_05610)	rsaL	rsaL (AL347_05610)	rsaL	-	27242034*, 18974177*, 22587778
rsaL (AL347_05610)	rsaL	phzM (AL347_21635)	phzM	-	27242034*, 18974177*, 22587778
rsaL (AL347_05610)	rsaL	hcnA (AL347_01585)	hcnA	-	27242034*, 18974177*, 22587778
rsaL (AL347_05610)	rsaL	phzF1 (AL347_21600)	phzF1	?	27242034*
rsaL (AL347_05610)	rsaL	phzF2 (AL347_03100)	phzF2	?	27242034*
rsaL (AL347_05610)	rsaL	phzB1 (AL347_21620)	phzB1	?	27242034*
rsaL (AL347_05610)	rsaL	phzC1 (AL347_21615)	phzC1	?	27242034*
rsaL (AL347_05610)	rsaL	phzE1 (AL347_21605)	phzE1	?	27242034*
rsaL (AL347_05610)	rsaL	phzD1 (AL347_21610)	phzD1	?	27242034*
rsaL (AL347_05610)	rsaL	phzG1 (AL347_21595)	phzG1	?	27242034*
rsaL (AL347_05610)	rsaL	phzG2 (AL347_03095)	phzG2	?	27242034*
rsaL (AL347_05610)	rsaL	hcnC (AL347_01575)	hcnC	?	27242034*
rsaL (AL347_05610)	rsaL	hcnB (AL347_01580)	hcnB	?	27242034*

Regulatory gene	Ortholog of the regulatory gene	Target gene	Ortholog of the target gene	Mode of regulation	Reference (PubMed)
acoR (AL347_21955)	acoR	PA4148 (AL347_21950)	PA4148	+	30058099
acoR (AL347_21955)	acoR	acoX (AL347_21945)	acoX	+	30058099
acoR (AL347_21955)	acoR	acoA (AL347_21940)	acoA	+	30058099
acoR (AL347_21955)	acoR	acoB (AL347_21930)	acoB	+	30058099
acoR (AL347_21955)	acoR	acoC (AL347_21930)	acoC	+	30058099
acoR (AL347_21955)	acoR	PA4153 (AL347_21925)	PA4153	+	30058099
agmR (AL347_02720)	agmR	exaA (AL347_02700)	exaA	+	18974177
agmR (AL347_02720)	agmR	exaB (AL347_02695)	exaB	+	18974177
agmR (AL347_02720)	agmR	exaC (AL347_02690)	exaC	+	18974177
agmR (AL347_02720)	agmR	pqqA (AL347_02685)	pqqA	+	18974177
agmR (AL347_02720)	agmR	pqqB (AL347_02680)	pqqB	+	18974177
agmR (AL347_02720)	agmR	pqqC (AL347_02675)	pqqC	+	18974177
agmR (AL347_02720)	agmR	pqqD (AL347_02670)	pqqD	+	18974177
agmR (AL347_02720)	agmR	pqqE (AL347_02665)	pqqE	+	18974177
agmR (AL347_02720)	agmR	pqqH (AL347_02660)	pqqH	+	19902179
agtR (AL347_20885)	agtR	agtA (AL347_20895)	agtA	+	23982201
agtR (AL347_20885)	agtR	agtB (AL347_20900)	agtB	+	23982201
agtR (AL347_20885)	agtR	agtC (AL347_20905)	agtC	+	23982201
agtR (AL347_20885)	agtR	agtD (AL347_20910)	agtD	+	23982201
aguR (AL347_19255)	aguR	aguA (AL347_19245)	aguA	-	18974177
aguR (AL347_19255)	aguR	aguB (AL347_19250)	aguB	-	18974177
algB (AL347_17235)	algB	algD (AL347_25110)	algD	+	22088575
algD (AL347_25110)	algD	algD (AL347_25110)	algD	?	18440972*
algQ (AL347_16020)	algQ	lasR (AL347_05615)	lasR	-	18440972
algQ (AL347_16020)	algQ	rhlR (AL347_25325)	rhlR	-	18440972
algQ (AL347_16020)	algQ	rpoD (AL347_20760)	rpoD	-	23991093, 18974177
algR (AL347_16050)	algR	PA0328 (AL347_19440)	PA0328 (aaaA)	+	18440972
algR (AL347_16050)	algR	ldcA (AL347_03550)	ldcA	+	18440972
algR (AL347_16050)	algR	PA1819 (AL347_03545)	PA1819	+	18440972
algR (AL347_16050)	algR	braZ (AL347_02755)	braZ	+	18440972
algR (AL347_16050)	algR	PA2042 (AL347_02395)	PA2042	+	18440972
algR (AL347_16050)	algR	rhlB (AL347_25320)	rhlB	-	18440972
algR (AL347_16050)	algR	argG (AL347_25185)	argG	-	18440972
algR (AL347_16050)	algR	alg8 (AL347_25105)	alg8	+	18440972
algR (AL347_16050)	algR	alg44 (AL347_25100)	alg44	+	18440972
algR (AL347_16050)	algR	algK (AL347_25095)	algK	+	18440972
algR (AL347_16050)	algR	algE (AL347_25090)	algE	+	18440972

algR (AL347_16050)	algR	algG (AL347_25085)	algG	+	18440972
algR (AL347_16050)	algR	algX (AL347_25080)	algX	+	18440972
algR (AL347_16050)	algR	algL (AL347_25075)	algL	+	18440972
algR (AL347_16050)	algR	algI (AL347_25065)	algI	+	18440972
algR (AL347_16050)	algR	algJ (AL347_25060)	algJ	+	18440972
algR (AL347_16050)	algR	algF (AL347_25055)	algF	+	18440972
algR (AL347_16050)	algR	algA (AL347_25050)	algA	+	18440972
algR (AL347_16050)	algR	PA3934 (AL347_23045)	PA3934	+	18440972
algR (AL347_16050)	algR	gdhA (AL347_12430)	gdhA	-	18440972
algR (AL347_16050)	algR	PA5152 (AL347_15480)	PA5152	+	18440972
algR (AL347_16050)	algR	PA5153 (AL347_15485)	PA5153	+	18440972
algR (AL347_16050)	algR	argB (AL347_16370)	argB	+	18440972
algR (AL347_16050)	algR	algR (AL347_16050)	algR	+	18974177
algR (AL347_16050)	algR	algZ (AL347_16055)	algZ	-	18974177
algR (AL347_16050)	algR	hcnA (AL347_01585)	hcnA	-	19270096
algR (AL347_16050)	algR	lipA (AL347_28575)	lipA	-	24187093
algR (AL347_16050)	algR	fimU (AL347_12235)	fimU	+	24187093
algR (AL347_16050)	algR	pilV (AL347_12240)	pilV	+	24187093
algR (AL347_16050)	algR	pilW (AL347_12245)	pilW	+	24187093
algR (AL347_16050)	algR	pilX (AL347_12250)	pilX	+	24187093
algR (AL347_16050)	algR	arcD (AL347_15575)	arcD	+	24187093
algR (AL347_16050)	algR	arcA (AL347_15580)	arcA	+	24187093
algR (AL347_16050)	algR	arcB (AL347_15585)	arcB	+	24187093
algR (AL347_16050)	algR	arcC (AL347_15590)	arcC	+	24187093
algR (AL347_16050)	algR	exsA (AL347_04135)	exsA	+	25070741
algR (AL347_16050)	algR	mucR (AL347_04065)	mucR	+	26206672
algR (AL347_16050)	algR	rsmA (AL347_08970)	rsmA	+	28320883
algR (AL347_16050)	algR	czcR (AL347_32045)	czcR	+	28400281
algR (AL347_16050)	algR	rhlI (AL347_25330)	rhlI	-	28400281
algR (AL347_16050)	algR	rhlA (AL347_25315)	rhlA	-	28400281
algR (AL347_16050)	algR	nrdB (AL347_07360)	nrdB	+	29263410
algR (AL347_16050)	algR	nrdA (AL347_07355)	nrdA	+	29263410
algR (AL347_16050)	algR	sigX (AL347_03799)	sigX	?	29729420
algR (AL347_16050)	algR	femI (AL347_03050)	femI	?	29729420
algR (AL347_16050)	algR	dnaA (AL347_17730)	dnaA	?	31270321
algR (AL347_16050)	algR	PA0277 (AL347_19170)	PA0277	?	31270321
algR (AL347_16050)	algR	PA0456 (AL347_20105)	PA0456	?	31270321
algR (AL347_16050)	algR	phdA (AL347_10095)	phdA	?	31270321

algR (AL347_16050)	algR	PA0703 (AL347_10030)	PA0703	?	31270321
algR (AL347_16050)	algR	cat (AL347_10015)	cat	?	31270321
algR (AL347_16050)	algR	putP (AL347_09600)	putP	?	31270321
algR (AL347_16050)	algR	ampDh3 (AL347_09475)	ampDh3	?	31270321
algR (AL347_16050)	algR	PA0839 (AL347_09315)	PA0839	?	31270321
algR (AL347_16050)	algR	PA0947 (AL347_08475)	PA0947	?	31270321
algR (AL347_16050)	algR	pqsA (AL347_08225)	pqsA	?	31270321
algR (AL347_16050)	algR	pqsB (AL347_08220)	pqsB	?	31270321
algR (AL347_16050)	algR	pqsC (AL347_08215)	pqsC	?	31270321
algR (AL347_16050)	algR	pqsD (AL347_08210)	pqsD	?	31270321
algR (AL347_16050)	algR	pqsE (AL347_08205)	pqsE	?	31270321
algR (AL347_16050)	algR	roeA (AL347_07640)	roeA	?	31270321
algR (AL347_16050)	algR	rhlC (AL347_07505)	rhlC	?	31270321
algR (AL347_16050)	algR	PA1159 (AL347_07340)	PA1159	?	31270321
algR (AL347_16050)	algR	cobO (AL347_06750)	cobO	?	31270321
algR (AL347_16050)	algR	PA1287 (AL347_06675)	PA1287	?	31270321
algR (AL347_16050)	algR	cyoA (AL347_06520)	cyoA	?	31270321
algR (AL347_16050)	algR	PA1333 (AL347_06435)	PA1333	?	31270321
algR (AL347_16050)	algR	fliL (AL347_05555)	fliL	?	31270321
algR (AL347_16050)	algR	ccmG (AL347_05360)	ccmG	?	31270321
algR (AL347_16050)	algR	alc (AL347_05185)	alc	?	31270321
algR (AL347_16050)	algR	PA1736 (AL347_04015)	PA1736	?	31270321
algR (AL347_16050)	algR	liuE (AL347_02555)	liuE	?	31270321
algR (AL347_16050)	algR	PA2301 (AL347_33520)	PA2301	?	31270321
algR (AL347_16050)	algR	PA2867 (AL347_28550)	PA2867	?	31270321
algR (AL347_16050)	algR	PA2939 (AL347_28180)	PA2939	?	31270321
algR (AL347_16050)	algR	xcpT (AL347_27335)	xcpT	?	31270321
algR (AL347_16050)	algR	fimV (AL347_27265)	fimV	?	31270321
algR (AL347_16050)	algR	asd (AL347_27255)	asd	?	31270321
algR (AL347_16050)	algR	PA3325 (AL347_26130)	PA3325	?	31270321
algR (AL347_16050)	algR	PA3471 (AL347_25360)	PA3471	?	31270321
algR (AL347_16050)	algR	PA3535 (AL347_25135)	PA3535	?	31270321
algR (AL347_16050)	algR	PA3572 (AL347_24940)	PA3572	?	31270321
algR (AL347_16050)	algR	pyrG (AL347_24590)	pyrG	?	31270321
algR (AL347_16050)	algR	dnaE (AL347_24575)	dnaE	?	31270321
algR (AL347_16050)	algR	mexK (AL347_24395)	mexK	?	31270321
algR (AL347_16050)	algR	PA3722 (AL347_24165)	PA3722	?	31270321
algR (AL347_16050)	algR	lasB (AL347_24155)	lasB	?	31270321

algR (AL347_16050)	algR	PA3740 (AL347_24070)	PA3740	?	31270321
algR (AL347_16050)	algR	nagE (AL347_23965)	nagE	?	31270321
algR (AL347_16050)	algR	PA3774 (AL347_23890)	PA3774	?	31270321
algR (AL347_16050)	algR	PA3840 (AL347_23540)	PA3840	?	31270321
algR (AL347_16050)	algR	ubiX (AL347_22615)	ubiX	?	31270321
algR (AL347_16050)	algR	PA4171 (AL347_21830)	PA4171	?	31270321
algR (AL347_16050)	algR	phzA1 (AL347_21625)	phzA1	?	31270321
algR (AL347_16050)	algR	phzB1 (AL347_21620)	phzB1	?	31270321
algR (AL347_16050)	algR	phzC1 (AL347_21615)	phzC1	?	31270321
algR (AL347_16050)	algR	phzD1 (AL347_21610)	phzD1	?	31270321
algR (AL347_16050)	algR	phzE1 (AL347_21605)	phzE1	?	31270321
algR (AL347_16050)	algR	phzF1 (AL347_21600)	phzF1	?	31270321
algR (AL347_16050)	algR	rcpC (AL347_10360)	rcpC	?	31270321
algR (AL347_16050)	algR	PA4320 (AL347_10445)	PA4320	?	31270321
algR (AL347_16050)	algR	PA4340 (AL347_10545)	PA4340	?	31270321
algR (AL347_16050)	algR	PA4357 (AL347_10630)	PA4357	?	31270321
algR (AL347_16050)	algR	PA4392 (AL347_10815)	PA4392	?	31270321
algR (AL347_16050)	algR	PA4475 (AL347_11240)	PA4475	?	31270321
algR (AL347_16050)	algR	PA4570 (AL347_12335)	PA4570	?	31270321
algR (AL347_16050)	algR	oprJ (AL347_12480)	oprJ	?	31270321
algR (AL347_16050)	algR	glyA3 (AL347_12505)	glyA3	?	31270321
algR (AL347_16050)	algR	PA4654 (AL347_12790)	PA4654	?	31270321
algR (AL347_16050)	algR	PA4676 (AL347_12920)	PA4676	?	31270321
algR (AL347_16050)	algR	PA4735 (AL347_13250)	PA4735	?	31270321
algR (AL347_16050)	algR	PA4948 (AL347_14360)	PA4948	?	31270321
algR (AL347_16050)	algR	opgG (mdoG)	opgG	?	31270321
algR (AL347_16050)	algR	tonB1 (AL347_17475)	tonB1	?	31270321
algR (AL347_16050)	algR	atpI (AL347_17625)	atpI	?	31270321
algR (AL347_16050)	algR	algD (AL347_25110)	algD	+	25488298, 31270321
algR (AL347_16050)	algR	pvdS (AL347_32895)	pvdS	-	28400281, 29729420
algR (AL347_16050)	algR	algC (AL347_16365)	algC	+	22587778
algR (AL347_16050)	algR	speA (AL347_13801)	speA	?	22587778
algU (AL347_09705)	algU	PA0856 (AL347_09230)	PA0856	+	18440972
algU (AL347_09705)	algU	bolA (AL347_09225)	bolA	+	18440972
algU (AL347_09705)	algU	PA1592 (AL347_04765)	PA1592	+	18440972
algU (AL347_09705)	algU	tal (AL347_28920)	tal	+	18440972
algU (AL347_09705)	algU	PA3262 (AL347_00200)	PA3262	+	18440972
algU (AL347_09705)	algU	alg8 (AL347_25105)	alg8	+	18440972

algU (AL347_09705)	algU	alg44 (AL347_25100)	alg44	+	18440972
algU (AL347_09705)	algU	algK (AL347_25095)	algK	+	18440972
algU (AL347_09705)	algU	algE (AL347_25090)	algE	+	18440972
algU (AL347_09705)	algU	algG (AL347_25085)	algG	+	18440972
algU (AL347_09705)	algU	algX (AL347_25080)	algX	+	18440972
algU (AL347_09705)	algU	algL (AL347_25075)	algL	+	18440972
algU (AL347_09705)	algU	algI (AL347_25065)	algI	+	18440972
algU (AL347_09705)	algU	algJ (AL347_25060)	algJ	+	18440972
algU (AL347_09705)	algU	algF (AL347_25055)	algF	+	18440972
algU (AL347_09705)	algU	algA (AL347_25050)	algA	+	18440972
algU (AL347_09705)	algU	PA3819 (AL347_23660)	PA3819	+	18440972
algU (AL347_09705)	algU	ivy (AL347_23215)	ivy/ PA3902	+	18440972, 22587778
algU (AL347_09705)	algU	phuR (AL347_13120)	phuR	+	18440972
algU (AL347_09705)	algU	dksA (AL347_13185)	dksA	+	18440972
algU (AL347_09705)	algU	PA5291 (AL347_16210)	PA5291 (betT2)	+	18440972
algU (AL347_09705)	algU	lptA (AL347_17775)	lptA	+	18974177
algU (AL347_09705)	algU	osmC (AL347_18065)	osmC	+	18974177
algU (AL347_09705)	algU	slyB (AL347_07915)	slyB	+	18974177
algU (AL347_09705)	algU	oprF (AL347_03795)	oprF	+	18974177
algU (AL347_09705)	algU	lptB (AL347_11170)	lptB	+	18974177
algU (AL347_09705)	algU	lptF (AL347_24315)	lptF	+	19332805
algU (AL347_09705)	algU	mucE (AL347_22545)	mucE	+	24138584
algU (AL347_09705)	algU	pyrR (AL347_19830)	pyrR	?	29729420
algU (AL347_09705)	algU	vfr (AL347_21140)	vfr	?	29729420
algU (AL347_09705)	algU	lasR (AL347_05615)	lasR	?	29729420
algU (AL347_09705)	algU	anr (AL347_05030)	anr	?	29729420
algU (AL347_09705)	algU	PA2177 (AL347_01675)	PA2177	?	29729420
algU (AL347_09705)	algU	ppyR (AL347_30095)	ppyR	?	29729420
algU (AL347_09705)	algU	gltR (AL347_00565)	gltR	?	29729420
algU (AL347_09705)	algU	cbrB (AL347_13205)	cbrB	?	29729420
algU (AL347_09705)	algU	kinB (AL347_17240)	kinB	?	29729420
algU (AL347_09705)	algU	rpoH (AL347_19690)	rpoH	+	18974177, 29729420
algU (AL347_09705)	algU	mucA (AL347_09700)	mucA	+	18974177, 29729420
algU (AL347_09705)	algU	mucB (AL347_09695)	mucB	+	18974177, 29729420
algU (AL347_09705)	algU	fleQ (AL347_07690)	fleQ	-	18974177, 29729420
algU (AL347_09705)	algU	amrZ (AL347_25805)	amrZ	+	19168621, 29729420
algU (AL347_09705)	algU	algU (AL347_09705)	algU	+	22088575, 29729420
algU (AL347_09705)	algU	algZ (AL347_16055)	algZ	+	22088575, 29729420

algU (AL347_09705)	algU	algB (AL347_17235)	algB	+	22088575, 29729420
algU (AL347_09705)	algU	algR (AL347_16050)	algR	+	25488298, 29729420
algU (AL347_09705)	algU	algD (AL347_25110)	algD	+	28320883, 30322853
algU (AL347_09705)	algU	rsmA (AL347_08970)	rsmA	+	29718466, 29729420
algU (AL347_09705)	algU	PA5526 (AL347_17450)	PA5526	+	Homology from 25780925
algU (AL347_09705)	algU	PA5514 (AL347_17390)	PA5514	+	Homology from 25780926
algU (AL347_09705)	algU	PA5488 (AL347_17260)	PA5488	+	Homology from 25780927
algU (AL347_09705)	algU	PA5473 (AL347_17180)	PA5473	+	Homology from 25780928
algU (AL347_09705)	algU	PA5472 (AL347_17170)	PA5472	+	Homology from 25780929
algU (AL347_09705)	algU	argB (AL347_16370)	argB	+	Homology from 25780930
algU (AL347_09705)	algU	poxB (AL347_16240)	poxB	+	Homology from 25780931
algU (AL347_09705)	algU	PA5290 (AL347_16205)	PA5290	+	Homology from 25780932
algU (AL347_09705)	algU	PA5258 (AL347_16035)	PA5258	+	Homology from 25780933
algU (AL347_09705)	algU	PA5246 (AL347_15970)	PA5246	+	Homology from 25780934
algU (AL347_09705)	algU	PA5245 (AL347_15965)	PA5245	+	Homology from 25780935
algU (AL347_09705)	algU	ppK (AL347_15950)	ppK	+	Homology from 25780936
algU (AL347_09705)	algU	PA5212 (AL347_15795)	PA5212	+	Homology from 25780937
algU (AL347_09705)	algU	PA5192 (AL347_15690)	PA5192 (pckA)	+	Homology from 25780938
algU (AL347_09705)	algU	PA5148 (AL347_15405)	PA5148	+	Homology from 25780939
algU (AL347_09705)	algU	mutY (AL347_15400)	mutY	+	Homology from 25780940
algU (AL347_09705)	algU	PA5146 (AL347_15395)	PA5146	+	Homology from 25780941
algU (AL347_09705)	algU	PA5131 (AL347_15310)	PA5131 (pgm, yibO)	+	Homology from 25780942
algU (AL347_09705)	algU	PA5121 (AL347_15255)	PA5121	+	Homology from 25780943
algU (AL347_09705)	algU	PA5110 (AL347_15195)	PA5110 (fbp, cfxF, cbbF)	+	Homology from 25780944
algU (AL347_09705)	algU	PA5109 (AL347_15190)	PA5109	+	Homology from 25780945
algU (AL347_09705)	algU	PA5108 (AL347_15185)	PA5108	+	Homology from 25780946
algU (AL347_09705)	algU	PA5107 (AL347_15180)	PA5107 (blc)	+	Homology from 25780947
algU (AL347_09705)	algU	opgG (mdoG)	opgG	+	Homology from 25780948
algU (AL347_09705)	algU	PA5042 (AL347_14855)	PA5042 (pilO)	+	Homology from 25780949
algU (AL347_09705)	algU	PA5038 (AL347_14835)	PA5038 (aroB)	+	Homology from 25780950
algU (AL347_09705)	algU	PA5037 (AL347_14830)	PA5037	+	Homology from 25780951
algU (AL347_09705)	algU	PA4923 (AL347_14225)	PA4923	+	Homology from 25780952
algU (AL347_09705)	algU	azu (AL347_14220)	azu	+	Homology from 25780953
algU (AL347_09705)	algU	PA4881 (AL347_14015)	PA4881	+	Homology from 25780954
algU (AL347_09705)	algU	PA4880 (AL347_14010)	PA4880	+	Homology from 25780955
algU (AL347_09705)	algU	PA4879 (AL347_14005)	PA4879	+	Homology from 25780956
algU (AL347_09705)	algU	PA4877 (AL347_13995)	PA4877	+	Homology from 25780957
algU (AL347_09705)	algU	PA4876 (AL347_13990)	PA4876	+	Homology from 25780958

algU (AL347_09705)	algU	PA4874 (AL347_13980)	PA4874	+	Homology from 25780959
algU (AL347_09705)	algU	gcbA (AL347_13825)	gcbA	+	Homology from 25780960
algU (AL347_09705)	algU	PA4842 (AL347_13815)	PA4842	+	Homology from 25780961
algU (AL347_09705)	algU	PA4841 (AL347_13810)	PA4841	+	Homology from 25780962
algU (AL347_09705)	algU	PA4788 (AL347_13540)	PA4788	+	Homology from 25780963
algU (AL347_09705)	algU	PA4786 (AL347_13530)	PA4786	+	Homology from 25780964
algU (AL347_09705)	algU	PA4717 (AL347_13155)	PA4717	+	Homology from 25780965
algU (AL347_09705)	algU	PA4714 (AL347_13140)	PA4714	+	Homology from 25780966
algU (AL347_09705)	algU	PA4713 (AL347_13135)	PA4713	+	Homology from 25780967
algU (AL347_09705)	algU	PA4700 (AL347_13070)	PA4700 (mrcB, pbpF, ponB)	+	Homology from 25780968
algU (AL347_09705)	algU	PA4699 (AL347_13065)	PA4699	+	Homology from 25780969
algU (AL347_09705)	algU	PA4698 (AL347_13060)	PA4698	+	Homology from 25780970
algU (AL347_09705)	algU	PA4639 (AL347_12715)	PA4639	+	Homology from 25780971
algU (AL347_09705)	algU	PA4575 (AL347_12360)	PA4575	+	Homology from 25780972
algU (AL347_09705)	algU	PA4542 (AL347_12190)	PA4542 (clpB)	+	Homology from 25780973
algU (AL347_09705)	algU	PA4481 (AL347_11270)	PA4481 (mreB,rodY, envB)	+	Homology from 25780974
algU (AL347_09705)	algU	PA4480 (AL347_11265)	PA4480 (mreC)	+	Homology from 25780975
algU (AL347_09705)	algU	PA4479 (AL347_11260)	PA4479 (mreD)	+	Homology from 25780976
algU (AL347_09705)	algU	PA4458 (AL347_11155)	PA4458	+	Homology from 25780977
algU (AL347_09705)	algU	PA4394 (AL347_10825)	PA4394	+	Homology from 25780978
algU (AL347_09705)	algU	PA4390 (AL347_10805)	PA4390	+	Homology from 25780979
algU (AL347_09705)	algU	PA4345 (AL347_10570)	PA4345	+	Homology from 25780980
algU (AL347_09705)	algU	PA4343 (AL347_10560)	PA4343	+	Homology from 25780981
algU (AL347_09705)	algU	PA4338 (AL347_10535)	PA4338	+	Homology from 25780982
algU (AL347_09705)	algU	PA4313 (AL347_10405)	PA4313	+	Homology from 25780983
algU (AL347_09705)	algU	PA4312 (AL347_10400)	PA4312	+	Homology from 25780984
algU (AL347_09705)	algU	PA4311 (AL347_10395)	PA4311	+	Homology from 25780985
algU (AL347_09705)	algU	PA4308 (AL347_10375)	PA4308	+	Homology from 25780986
algU (AL347_09705)	algU	ppgL (AL347_21660)	ppgL	+	Homology from 25780987
algU (AL347_09705)	algU	PA4172 (AL347_21825)	PA4172	+	Homology from 25780988
algU (AL347_09705)	algU	PA4171 (AL347_21830)	PA4171	+	Homology from 25780989
algU (AL347_09705)	algU	PA4170 (AL347_21835)	PA4170	+	Homology from 25780990
algU (AL347_09705)	algU	PA4113 (AL347_22130)	PA4113	+	Homology from 25780991
algU (AL347_09705)	algU	PA4017 (AL347_22625)	PA4017	+	Homology from 25780992
algU (AL347_09705)	algU	PA4001 (AL347_22705)	PA4001 (sltB1, mltB2)	+	Homology from 25780993
algU (AL347_09705)	algU	PA3992 (AL347_22745)	PA3992 (sltB3)	+	Homology from 25780994
algU (AL347_09705)	algU	PA3962 (AL347_22900)	PA3962	+	Homology from 25780995
algU (AL347_09705)	algU	PA3945 (AL347_22990)	PA3945	+	Homology from 25780996

algU (AL347_09705)	algU	PA3940 (AL347_23015)	PA3940	+	Homology from 25780997
algU (AL347_09705)	algU	PA3891 (AL347_23275)	PA3891 (opuCA)	+	Homology from 25780998
algU (AL347_09705)	algU	PA3890 (AL347_23280)	PA3890 (opuCB)	+	Homology from 25780999
algU (AL347_09705)	algU	PA3889 (AL347_23285)	PA3889 (opuCC)	+	Homology from 25781000
algU (AL347_09705)	algU	PA3888 (AL347_23290)	PA3888 (opuCD)	+	Homology from 25781001
algU (AL347_09705)	algU	PA3887 (AL347_23300)	PA3887 (nhaP)	+	Homology from 25781002
algU (AL347_09705)	algU	PA3856 (AL347_23455)	PA3856	+	Homology from 25781003
algU (AL347_09705)	algU	PA3795 (AL347_23780)	PA3795	+	Homology from 25781004
algU (AL347_09705)	algU	PA3794 (AL347_23785)	PA3794	+	Homology from 25781005
algU (AL347_09705)	algU	PA3793 (AL347_23790)	PA3793	+	Homology from 25781006
algU (AL347_09705)	algU	PA3762 (AL347_23960)	PA3762	+	Homology from 25781007
algU (AL347_09705)	algU	PA3734 (AL347_24100)	PA3734	+	Homology from 25781008
algU (AL347_09705)	algU	PA3710 (AL347_24225)	PA3710	+	Homology from 25781009
algU (AL347_09705)	algU	PA3691 (AL347_24320)	PA3691	+	Homology from 25781010
algU (AL347_09705)	algU	mucP (AL347_24530)	mucP	+	Homology from 25781011
algU (AL347_09705)	algU	PA3612 (AL347_24720)	PA3612	+	Homology from 25781012
algU (AL347_09705)	algU	PA3611 (AL347_24725)	PA3611	+	Homology from 25781013
algU (AL347_09705)	algU	PA3461 (AL347_25410)	PA3461	+	Homology from 25781014
algU (AL347_09705)	algU	PA3460 (AL347_25415)	PA3460	+	Homology from 25781015
algU (AL347_09705)	algU	PA3459 (AL347_25420)	PA3459	+	Homology from 25781016
algU (AL347_09705)	algU	PA3386 (AL347_25800)	PA3386	+	Homology from 25781017
algU (AL347_09705)	algU	phnC (AL347_25810)	phnC	+	Homology from 25781018
algU (AL347_09705)	algU	PA3383 (AL347_25815)	PA3383	+	Homology from 25781019
algU (AL347_09705)	algU	PA3382 (AL347_25820)	PA3382 (phnE)	+	Homology from 25781020
algU (AL347_09705)	algU	PA3380 (AL347_25830)	PA3380	+	Homology from 25781021
algU (AL347_09705)	algU	PA3379 (AL347_25835)	PA3379	+	Homology from 25781022
algU (AL347_09705)	algU	PA3378 (AL347_25840)	PA3378	+	Homology from 25781023
algU (AL347_09705)	algU	PA3377 (AL347_25845)	PA3377	+	Homology from 25781024
algU (AL347_09705)	algU	PA3376 (AL347_25850)	PA3376	+	Homology from 25781025
algU (AL347_09705)	algU	PA3375 (AL347_25855)	PA3375	+	Homology from 25781026
algU (AL347_09705)	algU	PA3374 (AL347_25860)	PA3374	+	Homology from 25781027
algU (AL347_09705)	algU	PA3373 (AL347_25865)	PA3373	+	Homology from 25781028
algU (AL347_09705)	algU	PA3372 (AL347_25870)	PA3372	+	Homology from 25781029
algU (AL347_09705)	algU	PA3274 (AL347_26415)	PA3274	+	Homology from 25781030
algU (AL347_09705)	algU	PA3270 (AL347_26435)	PA3270	+	Homology from 25781031
algU (AL347_09705)	algU	PA3256 (AL347_00240)	PA3256	+	Homology from 25781032
algU (AL347_09705)	algU	PA3203 (AL347_00505)	PA3203	+	Homology from 25781033
algU (AL347_09705)	algU	PA3093 (AL347_27390)	PA3093	+	Homology from 25781034

algU (AL347_09705)	algU	PA3076 (AL347_27480)	PA3076	+	Homology from 25781035
algU (AL347_09705)	algU	PA3042 (AL347_27640)	PA3042	+	Homology from 25781036
algU (AL347_09705)	algU	PA3041 (AL347_27645)	PA3041	+	Homology from 25781037
algU (AL347_09705)	algU	PA3040 (AL347_27650)	PA3040	+	Homology from 25781038
algU (AL347_09705)	algU	PA3039 (AL347_27655)	PA3039	+	Homology from 25781039
algU (AL347_09705)	algU	PA3031 (AL347_27700)	PA3031	+	Homology from 25781040
algU (AL347_09705)	algU	PA3012 (AL347_27800)	PA3012	+	Homology from 25781041
algU (AL347_09705)	algU	nagZ (AL347_27835)	nagZ	+	Homology from 25781042
algU (AL347_09705)	algU	PA3004 (AL347_27840)	PA3004	+	Homology from 25781043
algU (AL347_09705)	algU	PA3001 (AL347_27855)	PA3001	+	Homology from 25781044
algU (AL347_09705)	algU	PA2988 (AL347_27925)	PA2988 (lolE)	+	Homology from 25781045
algU (AL347_09705)	algU	PA2987 (AL347_27930)	PA2987 (lolD, ycfV)	+	Homology from 25781046
algU (AL347_09705)	algU	PA2986 (AL347_27935)	PA2986 (lolC)	+	Homology from 25781047
algU (AL347_09705)	algU	sbrR (AL347_28410)	sbrR	+	Homology from 25781048
algU (AL347_09705)	algU	PA2884 (AL347_28465)	PA2884	+	Homology from 25781049
algU (AL347_09705)	algU	PA2883 (AL347_28470)	PA2883	+	Homology from 25781050
algU (AL347_09705)	algU	PA2815 (fadE)	PA2815	+	Homology from 25781051
algU (AL347_09705)	algU	PA2559 (AL347_31845)	PA2559	+	Homology from 25781052
algU (AL347_09705)	algU	PA2546 (AL347_31915)	PA2546	+	Homology from 25781053
algU (AL347_09705)	algU	PA2528 (AL347_32015)	PA2528 (muxA, yegM)	+	Homology from 25781054
algU (AL347_09705)	algU	PA2527 (AL347_32020)	PA2527 (muxB, yegN)	+	Homology from 25781055
algU (AL347_09705)	algU	PA2526 (AL347_32025)	PA2526 (muxC, yegO)	+	Homology from 25781056
algU (AL347_09705)	algU	PA2525 (AL347_32030)	PA2525 (opmB)	+	Homology from 25781057
algU (AL347_09705)	algU	ptrC (AL347_32590)	ptrC	+	Homology from 25781058
algU (AL347_09705)	algU	PA2485 (AL347_32595)	PA2485	+	Homology from 25781059
algU (AL347_09705)	algU	sdaA (AL347_32800)	sdaA	+	Homology from 25781060
algU (AL347_09705)	algU	gcvT2 (AL347_32805)	gcvT2	+	Homology from 25781061
algU (AL347_09705)	algU	PA2434 (AL347_32845)	PA2434	+	Homology from 25781062
algU (AL347_09705)	algU	PA2433 (AL347_32850)	PA2433	+	Homology from 25781063
algU (AL347_09705)	algU	PA2416 (AL347_32945)	PA2416 (treA)	+	Homology from 25781064
algU (AL347_09705)	algU	PA2415 (AL347_32950)	PA2415	+	Homology from 25781065
algU (AL347_09705)	algU	PA2414 (AL347_32955)	PA2414	+	Homology from 25781066
algU (AL347_09705)	algU	pvdH (AL347_32960)	pvdH	+	Homology from 25781067
algU (AL347_09705)	algU	PA2371 (AL347_33165)	PA2371 (clpV3)	+	Homology from 25781068
algU (AL347_09705)	algU	PA2370 (AL347_33170)	PA2370 (hsiH3)	+	Homology from 25781069
algU (AL347_09705)	algU	PA2369 (AL347_33175)	PA2369 (hsiG3)	+	Homology from 25781070
algU (AL347_09705)	algU	PA2368 (AL347_33180)	PA2368 (hsiF3)	+	Homology from 25781071
algU (AL347_09705)	algU	PA2367 (AL347_33185)	PA2367 (hcp3)	+	Homology from 25781072

algU (AL347_09705)	algU	PA2366 (AL347_33190)	PA2366 (hsiC3, puuD)	+	Homology from 25781073
algU (AL347_09705)	algU	PA2365 (AL347_33195)	PA2365 (hsiB3)	+	Homology from 25781074
algU (AL347_09705)	algU	gad (AL347_00735)	gad	+	Homology from 25781075
algU (AL347_09705)	algU	kguD (AL347_00745)	kguD	+	Homology from 25781076
algU (AL347_09705)	algU	kguK (AL347_00755)	kguK	+	Homology from 25781077
algU (AL347_09705)	algU	PA2176 (AL347_01680)	PA2176	+	Homology from 25781078
algU (AL347_09705)	algU	PA2175 (AL347_01685)	PA2175	+	Homology from 25781079
algU (AL347_09705)	algU	PA2172 (AL347_01705)	PA2172	+	Homology from 25781080
algU (AL347_09705)	algU	PA2169 (AL347_01720)	PA2169	+	Homology from 25781081
algU (AL347_09705)	algU	PA2165 (AL347_01745)	PA2165	+	Homology from 25781082
algU (AL347_09705)	algU	PA2164 (AL347_01750)	PA2164	+	Homology from 25781083
algU (AL347_09705)	algU	PA2163 (AL347_01755)	PA2163	+	Homology from 25781084
algU (AL347_09705)	algU	PA2162 (AL347_01760)	PA2162	+	Homology from 25781085
algU (AL347_09705)	algU	PA2161 (AL347_01765)	PA2161	+	Homology from 25781086
algU (AL347_09705)	algU	PA2160 (AL347_01770)	PA2160	+	Homology from 25781087
algU (AL347_09705)	algU	PA2155 (AL347_01800)	PA2155	+	Homology from 25781088
algU (AL347_09705)	algU	PA2153 (AL347_01810)	PA2153 (glgB)	+	Homology from 25781089
algU (AL347_09705)	algU	PA2152 (AL347_01815)	PA2152	+	Homology from 25781090
algU (AL347_09705)	algU	PA2151 (AL347_01820)	PA2151	+	Homology from 25781091
algU (AL347_09705)	algU	PA2150 (AL347_01825)	PA2150	+	Homology from 25781092
algU (AL347_09705)	algU	PA2149 (AL347_01830)	PA2149	+	Homology from 25781093
algU (AL347_09705)	algU	PA2148 (AL347_01835)	PA2148	+	Homology from 25781094
algU (AL347_09705)	algU	PA2147 (AL347_01845)	PA2147 (katE)	+	Homology from 25781095
algU (AL347_09705)	algU	PA2146 (AL347_01850)	PA2146	+	Homology from 25781096
algU (AL347_09705)	algU	PA2144 (AL347_01860)	PA2144	+	Homology from 25781097
algU (AL347_09705)	algU	PA2143 (AL347_01865)	PA2143	+	Homology from 25781098
algU (AL347_09705)	algU	PA2135 (AL347_01915)	PA2135	+	Homology from 25781099
algU (AL347_09705)	algU	PA2108 (AL347_02070)	PA2108	+	Homology from 25781100
algU (AL347_09705)	algU	PA2023 (AL347_02490)	PA2023 (galU, hasC, gtaB)	+	Homology from 25781101
algU (AL347_09705)	algU	pqqH (AL347_02660)	pqqH	+	Homology from 25781102
algU (AL347_09705)	algU	PA1959 (AL347_02820)	PA1959 (bacA)	+	Homology from 25781103
algU (AL347_09705)	algU	PA1958 (AL347_02825)	PA1958	+	Homology from 25781104
algU (AL347_09705)	algU	PA1957 (AL347_02830)	PA1957	+	Homology from 25781105
algU (AL347_09705)	algU	PA1870 (AL347_03270)	PA1870	+	Homology from 25781106
algU (AL347_09705)	algU	PA1857 (AL347_03340)	PA1857	+	Homology from 25781107
algU (AL347_09705)	algU	PA1833 (AL347_03475)	PA1833	+	Homology from 25781108
algU (AL347_09705)	algU	PA1832 (AL347_03480)	PA1832	+	Homology from 25781109
algU (AL347_09705)	algU	PA1745 (AL347_03970)	PA1745	+	Homology from 25781110

algU (AL347_09705)	algU	PA1744 (AL347_03975)	PA1744	+	Homology from 25781111
algU (AL347_09705)	algU	PA1743 (AL347_03980)	PA1743	+	Homology from 25781112
algU (AL347_09705)	algU	hsiF2 (AL347_04415)	hsiF2	+	Homology from 25781113
algU (AL347_09705)	algU	hsiC2 (AL347_04420)	hsiC2	+	Homology from 25781114
algU (AL347_09705)	algU	hsiB2 (AL347_04425)	hsiB2	+	Homology from 25781115
algU (AL347_09705)	algU	hsiA2 (AL347_04430)	hsiA2	+	Homology from 25781116
algU (AL347_09705)	algU	PA1616 (AL347_04645)	PA1616	+	Homology from 25781117
algU (AL347_09705)	algU	PA1615 (AL347_04650)	PA1615	+	Homology from 25781118
algU (AL347_09705)	algU	PA1614 (AL347_04655)	PA1614 (gpsA, gpdA)	+	Homology from 25781119
algU (AL347_09705)	algU	fabA (AL347_04675)	fabA	+	Homology from 25781120
algU (AL347_09705)	algU	PA1609 (AL347_04680)	PA1609 (fabB)	+	Homology from 25781121
algU (AL347_09705)	algU	PA1605 (AL347_04700)	PA1605	+	Homology from 25781122
algU (AL347_09705)	algU	PA1594 (AL347_04755)	PA1594	+	Homology from 25781123
algU (AL347_09705)	algU	PA1593 (AL347_04760)	PA1593	+	Homology from 25781124
algU (AL347_09705)	algU	PA1579 (AL347_04830)	PA1579	+	Homology from 25781125
algU (AL347_09705)	algU	PA1578 (AL347_04840)	PA1578	+	Homology from 25781126
algU (AL347_09705)	algU	PA1577 (AL347_04845)	PA1577	+	Homology from 25781127
algU (AL347_09705)	algU	PA1573 (AL347_04865)	PA1573	+	Homology from 25781128
algU (AL347_09705)	algU	PA1572 (AL347_04870)	PA1572	+	Homology from 25781129
algU (AL347_09705)	algU	PA1571 (AL347_04875)	PA1571	+	Homology from 25781130
algU (AL347_09705)	algU	PA1562 (AL347_04930)	PA1562 (acnA, acn)	+	Homology from 25781131
algU (AL347_09705)	algU	PA1408 (AL347_05780)	PA1408	+	Homology from 25781132
algU (AL347_09705)	algU	PA1324 (AL347_06485)	PA1324	+	Homology from 25781133
algU (AL347_09705)	algU	PA1323 (AL347_06490)	PA1323	+	Homology from 25781134
algU (AL347_09705)	algU	PA1295 (AL347_06635)	PA1295	+	Homology from 25781135
algU (AL347_09705)	algU	PA1294 (AL347_06640)	PA1294 (rnd)	+	Homology from 25781136
algU (AL347_09705)	algU	PA1288 (AL347_06670)	PA1288	+	Homology from 25781137
algU (AL347_09705)	algU	PA1270 (AL347_06760)	PA1270	+	Homology from 25781138
algU (AL347_09705)	algU	PA1170 (AL347_07285)	PA1170	+	Homology from 25781139
algU (AL347_09705)	algU	PA1116 (AL347_07580)	PA1116	+	Homology from 25781140
algU (AL347_09705)	algU	PA1115 (AL347_07585)	PA1115	+	Homology from 25781141
algU (AL347_09705)	algU	PA1114 (AL347_07590)	PA1114	+	Homology from 25781142
algU (AL347_09705)	algU	PA1111 (AL347_07620)	PA1111	+	Homology from 25781143
algU (AL347_09705)	algU	PA0967 (AL347_08370)	PA0967 (ruvB)	+	Homology from 25781144
algU (AL347_09705)	algU	PA0962 (AL347_08395)	PA0962 (dps)	+	Homology from 25781145
algU (AL347_09705)	algU	PA0944 (AL347_08495)	PA0944 (purN)	+	Homology from 25781146
algU (AL347_09705)	algU	PA0943 (AL347_08500)	PA0943	+	Homology from 25781147
algU (AL347_09705)	algU	dctM (AL347_09065)	dctM	+	Homology from 25781148

algU (AL347_09705)	algU	dctQ (AL347_09070)	dctQ	+	Homology from 25781149
algU (AL347_09705)	algU	PA0884 (AL347_09075)	PA0884	+	Homology from 25781150
algU (AL347_09705)	algU	PA0883 (AL347_09080)	PA0883	+	Homology from 25781151
algU (AL347_09705)	algU	PA0882 (AL347_09085)	PA0882	+	Homology from 25781152
algU (AL347_09705)	algU	PA0881 (AL347_09090)	PA0881	+	Homology from 25781153
algU (AL347_09705)	algU	PA0880 (AL347_09095)	PA0880	+	Homology from 25781154
algU (AL347_09705)	algU	PA0879 (AL347_09100)	PA0879	+	Homology from 25781155
algU (AL347_09705)	algU	PA0878 (AL347_09105)	PA0878	+	Homology from 25781156
algU (AL347_09705)	algU	PA0833 (AL347_09350)	PA0833	+	Homology from 25781157
algU (AL347_09705)	algU	PA0805 (AL347_09485)	PA0805	+	Homology from 25781158
algU (AL347_09705)	algU	mucD (AL347_09685)	mucD	+	Homology from 25781159
algU (AL347_09705)	algU	PA0682 (AL347_10140)	PA0682 (hxcX)	+	Homology from 25781160
algU (AL347_09705)	algU	PA0681 (AL347_10145)	PA0681 (hxcT)	+	Homology from 25781161
algU (AL347_09705)	algU	PA0658 (AL347_21170)	PA0658	+	Homology from 25781162
algU (AL347_09705)	algU	PA0598 (AL347_20870)	PA0598	+	Homology from 25781163
algU (AL347_09705)	algU	PA0597 (AL347_20865)	PA0597	+	Homology from 25781164
algU (AL347_09705)	algU	PA0596 (AL347_20860)	PA0596	+	Homology from 25781165
algU (AL347_09705)	algU	surA (AL347_20850)	surA	+	Homology from 25781166
algU (AL347_09705)	algU	PA0567 (AL347_20675)	PA0567	+	Homology from 25781167
algU (AL347_09705)	algU	PA0552 (AL347_20605)	PA0552 (pgk)	+	Homology from 25781168
algU (AL347_09705)	algU	PA0537 (AL347_20530)	PA0537	+	Homology from 25781169
algU (AL347_09705)	algU	PA0536 (AL347_20525)	PA0536	+	Homology from 25781170
algU (AL347_09705)	algU	PA0377 (AL347_19695)	PA0377	+	Homology from 25781171
algU (AL347_09705)	algU	PA0375 (AL347_19685)	PA0375 (ftsX)	+	Homology from 25781172
algU (AL347_09705)	algU	PA0374 (AL347_19680)	PA0374 (ftsE)	+	Homology from 25781173
algU (AL347_09705)	algU	PA0373 (AL347_19675)	PA0373 (ftsY)	+	Homology from 25781174
algU (AL347_09705)	algU	PA0372 (AL347_19670)	PA0372	+	Homology from 25781175
algU (AL347_09705)	algU	PA0371 (AL347_19665)	PA0371	+	Homology from 25781176
algU (AL347_09705)	algU	PA0370 (AL347_19660)	PA0370	+	Homology from 25781177
algU (AL347_09705)	algU	PA0357 (AL347_19590)	PA0357 (mutM)	+	Homology from 25781178
algU (AL347_09705)	algU	PA0355 (AL347_19580)	PA0355 (pfpI)	+	Homology from 25781179
algU (AL347_09705)	algU	PA0354 (AL347_19575)	PA0354	+	Homology from 25781180
algU (AL347_09705)	algU	PA0333 (AL347_19465)	PA0333	+	Homology from 25781181
algU (AL347_09705)	algU	PA0332 (AL347_19460)	PA0332	+	Homology from 25781182
algU (AL347_09705)	algU	PA0329 (AL347_19445)	PA0329	+	Homology from 25781183
algU (AL347_09705)	algU	PA0309 (AL347_19340)	PA0309	+	Homology from 25781184
algU (AL347_09705)	algU	gabD (AL347_19100)	gabD	+	Homology from 25781185
algU (AL347_09705)	algU	PA0232 (AL347_18930)	PA0232 (pcaC)	+	Homology from 25781186

algU (AL347_09705)	algU	triC (AL347_18575)	triC	+	Homology from 25781187
algU (AL347_09705)	algU	triB (AL347_18570)	triB	+	Homology from 25781188
algU (AL347_09705)	algU	PA0156 (AL347_18565)	PA0156 (triA)	+	Homology from 25781189
algU (AL347_09705)	algU	PA0138 (AL347_18465)	PA0138	+	Homology from 25781190
algU (AL347_09705)	algU	PA0137 (AL347_18460)	PA0137	+	Homology from 25781191
algU (AL347_09705)	algU	PA0136 (AL347_18455)	PA0136	+	Homology from 25781192
algU (AL347_09705)	algU	PA0134 (AL347_18445)	PA0134	+	Homology from 25781193
algU (AL347_09705)	algU	PA0071 (AL347_18125)	PA0071 (tagR1)	+	Homology from 25781194
algU (AL347_09705)	algU	PA0062 (AL347_18080)	PA0062	+	Homology from 25781195
algU (AL347_09705)	algU	PA0061 (AL347_18075)	PA0061	+	Homology from 25781196
algU (AL347_09705)	algU	PA0060 (AL347_18070)	PA0060	+	Homology from 25781197
algU (AL347_09705)	algU	PA2754 (AL347_29410)	PA2754	+	Homology from 25781198
algU (AL347_09705)	algU	PA2751 (AL347_29430)	PA2751	+	Homology from 25781199
algU (AL347_09705)	algU	PA2706 (AL347_29875)	PA2706	+	Homology from 25781200
algU (AL347_09705)	algU	PA2705 (AL347_29880)	PA2705	+	Homology from 25781201
algU (AL347_09705)	algU	PA5566 (AL347_17655)	PA5566	+	Homology from 25781202
algU (AL347_09705)	algU	mifS (AL347_17380)	mifS	+	Homology from 25781203
algU (AL347_09705)	algU	mifR (AL347_17375)	mifR	+	Homology from 25780925
algU (AL347_09705)	algU	PA5487 (AL347_17255)	PA5487 (dgcH)	+	Homology from 25780926
algU (AL347_09705)	algU	PA5428 (AL347_16935)	PA5428	+	Homology from 25780927
algU (AL347_09705)	algU	nalD (AL347_24930)	nalD	+	Homology from 25780928
algU (AL347_09705)	algU	PA3458 (AL347_25425)	PA3458	+	Homology from 25780929
algU (AL347_09705)	algU	phnF (AL347_25825)	phnF	+	Homology from 25780930
algU (AL347_09705)	algU	cpxR (AL347_00500)	cpxR	+	Homology from 25780931
algU (AL347_09705)	algU	PA3177 (AL347_26935)	PA3177	+	Homology from 25780932
algU (AL347_09705)	algU	PA3094 (AL347_27385)	PA3094	+	Homology from 25780933
algU (AL347_09705)	algU	sbrI (AL347_28405)	sbrI	?	Homology from 25780934
algU (AL347_09705)	algU	PA2881 (AL347_28480)	PA2881	+	Homology from 25780935
algU (AL347_09705)	algU	PA2798 (AL347_28910)	PA2798	+	Homology from 25780936
algU (AL347_09705)	algU	PA2797 (AL347_28915)	PA2797	+	Homology from 25780937
algU (AL347_09705)	algU	PA2704 (AL347_29885)	PA2704	+	Homology from 25780938
algU (AL347_09705)	algU	PA2484 (AL347_32600)	PA2484	+	Homology from 25780939
algU (AL347_09705)	algU	PA2417 (AL347_32940)	PA2417	+	Homology from 25780940
algU (AL347_09705)	algU	PA2281 (AL347_00655)	PA2281	+	Homology from 25780941
algU (AL347_09705)	algU	ptxR (AL347_33740)	ptxR	+	Homology from 25780942
algU (AL347_09705)	algU	mucC (AL347_09690)	mucC	+	Homology from 25780943
algU (AL347_09705)	algU	PA0356 (AL347_19585)	PA0356	+	Homology from 25780944
algU (AL347_09705)	algU	PA0225 (AL347_18895)	PA0225	+	Homology from 25780945

algU (AL347_09705)	algU	PA3952 (AL347_22951)	PA3952	+	22587778
algU (AL347_09705)	algU	PA1053 (AL347_07915)	PA1053	+	22587778, 27242034*
algU (AL347_09705)	algU	fusA	fusA	?	27242034*, 18974177*, 22587778
algU (AL347_09705)	algU	fliC (AL347_07715)	fliC	?	27242034*
algU (AL347_09705)	algU	osmE (AL347_13990)	osmE	?	27242034*
algW (AL347_11095)	algW	algU (AL347_09705)	algU	+	18974177
algW (AL347_11095)	algW	algD (AL347_25110)	algD	+	18974177
algW (AL347_11095)	algW	mucA (AL347_09700)	mucA	+	27270273
algZ (AL347_16055)	algZ	alg8 (AL347_25105)	alg8	+	18440972
algZ (AL347_16055)	algZ	alg44 (AL347_25100)	alg44	+	18440972
algZ (AL347_16055)	algZ	algK (AL347_25095)	algK	+	18440972
algZ (AL347_16055)	algZ	algE (AL347_25090)	algE	+	18440972
algZ (AL347_16055)	algZ	algG (AL347_25085)	algG	+	18440972
algZ (AL347_16055)	algZ	algX (AL347_25080)	algX	+	18440972
algZ (AL347_16055)	algZ	algL (AL347_25075)	algL	+	18440972
algZ (AL347_16055)	algZ	algI (AL347_25065)	algI	+	18440972
algZ (AL347_16055)	algZ	algJ (AL347_25060)	algJ	+	18440972
algZ (AL347_16055)	algZ	algF (AL347_25055)	algF	+	18440972
algZ (AL347_16055)	algZ	algA (AL347_25050)	algA	+	18440972
algZ (AL347_16055)	algZ	fleQ (AL347_07690)	fleQ	-	18974177
algZ (AL347_16055)	algZ	algZ (AL347_16055)	algZ	-	18974177
algZ (AL347_16055)	algZ	algD (AL347_25110)	algD	+	22088575
algZ (AL347_16055)	algZ	exsA (AL347_04135)	exsA	-	25070741
amgR (AL347_15735)	amgR	mexB (AL347_19950)	mexB	+	30289929
amgR (AL347_15735)	amgR	oprM (AL347_19955)	oprM	+	30289929
ampR (AL347_22150)	ampR	PA5514 (AL347_17390)	PA5514	-	16120476
ampR (AL347_22150)	ampR	lasI (AL347_05605)	lasI	-	18974177
ampR (AL347_22150)	ampR	lasA (AL347_03265)	lasA	-	18974177
ampR (AL347_22150)	ampR	lasB (AL347_24155)	lasB	+	18974177
ampR (AL347_22150)	ampR	creD (AL347_20155)	creD	-	22479525
ampR (AL347_22150)	ampR	prtR (AL347_20935)	prtR	+	22479525
ampR (AL347_22150)	ampR	algU (AL347_09705)	algU	-	22479525
ampR (AL347_22150)	ampR	ampDh3 (AL347_09475)	ampDh3	+	22479525
ampR (AL347_22150)	ampR	mexT (AL347_32559)	mexT	-	22479525
ampR (AL347_22150)	ampR	nagZ (AL347_27835)	nagZ	+	22479525
ampR (AL347_22150)	ampR	rpoS (AL347_24665)	rpoS	+	22479525
ampR (AL347_22150)	ampR	ampP (AL347_21580)	ampP	+	22479525
ampR (AL347_22150)	ampR	ampG (AL347_10820)	ampG	+	22479525

ampR (AL347_22150)	ampR	ampD (AL347_11480)	ampD	+	22479525
ampR (AL347_22150)	ampR	algB (AL347_17235)	algB	-	22479525
ampR (AL347_22150)	ampR	ampDh2 (AL347_17245)	ampDh2	+	22479525
ampR (AL347_22150)	ampR	mexE (AL347_32510)	mexE	+	24464693
ampR (AL347_22150)	ampR	mexF (AL347_32505)	mexF	+	24464693
ampR (AL347_22150)	ampR	oprN (AL347_32500)	oprN	+	24464693
ampR (AL347_22150)	ampR	mvfR (AL347_08190)	mvfR	+	25182487
ampR (AL347_22150)	ampR	rhlR (AL347_25325)	rhlR	+	25182487
ampR (AL347_22150)	ampR	poxB (AL347_16240)	poxB	+	25182487
ampR (AL347_22150)	ampR	PA0149 (AL347_18525)	PA0149	?	29729420
ampR (AL347_22150)	ampR	femI (AL347_03050)	femI	?	29729420
ampR (AL347_22150)	ampR	pvdS (AL347_32895)	pvdS	?	29729420
ampR (AL347_22150)	ampR	sbrI (AL347_28405)	sbrI	?	29729420
ampR (AL347_22150)	ampR	lasR (AL347_05615)	lasR	-	18974177, 25182487
amrZ (AL347_25805)	amrZ	PA0038 (AL347_17945)	PA0038	-	24603766
amrZ (AL347_25805)	amrZ	PA0082 (AL347_18180)	PA0082 (tssA1)	+	24603766
amrZ (AL347_25805)	amrZ	PA0494 (AL347_20305)	PA0494	-	24603766
amrZ (AL347_25805)	amrZ	vreA (AL347_10180)	vreA	+	24603766
amrZ (AL347_25805)	amrZ	PA0938 (AL347_08530)	PA0938 (wzz2)	-	24603766
amrZ (AL347_25805)	amrZ	flgE (AL347_07775)	flgE	-	24603766
amrZ (AL347_25805)	amrZ	flgG (AL347_07765)	flgG	-	24603766
amrZ (AL347_25805)	amrZ	PA1088 (AL347_07735)	PA1088	-	24603766
amrZ (AL347_25805)	amrZ	fleQ (AL347_07690)	fleQ	-	24603766
amrZ (AL347_25805)	amrZ	fliF (AL347_07670)	fliF	-	24603766
amrZ (AL347_25805)	amrZ	PA1199 (AL347_07135)	PA1199	-	24603766
amrZ (AL347_25805)	amrZ	PA1340 (AL347_06395)	PA1340 (aatM)	-	24603766
amrZ (AL347_25805)	amrZ	hsiB2 (AL347_04425)	hsiB2	-	24603766
amrZ (AL347_25805)	amrZ	PA1664 (AL347_04385)	PA1664 (orfX)	-	24603766
amrZ (AL347_25805)	amrZ	PA1668 (AL347_04365)	PA1668 (dotU2)	-	24603766
amrZ (AL347_25805)	amrZ	PA1680 (AL347_04305)	PA1680	-	24603766
amrZ (AL347_25805)	amrZ	PA1738 (AL347_04005)	PA1738	-	24603766
amrZ (AL347_25805)	amrZ	PA1784 (AL347_03760)	PA1784	+	24603766
amrZ (AL347_25805)	amrZ	PA1913 (AL347_03045)	PA1913	-	24603766
amrZ (AL347_25805)	amrZ	PA1941 (AL347_02910)	PA1941	-	24603766
amrZ (AL347_25805)	amrZ	pslA (AL347_00910)	pslA	-	24603766
amrZ (AL347_25805)	amrZ	PA2462 (AL347_32710)	PA2462	-	24603766
amrZ (AL347_25805)	amrZ	PA2567 (AL347_31795)	PA2567	-	24603766
amrZ (AL347_25805)	amrZ	PA3019 (AL347_27760)	PA3019	-	24603766

amrZ (AL347_25805)	amrZ	PA3234 (actP)	PA3234	-	24603766
amrZ (AL347_25805)	amrZ	PA3235 (AL347_26630)	PA3235	-	24603766
amrZ (AL347_25805)	amrZ	PA3353 (AL347_25985)	PA3353 (flgZ)	-	24603766
amrZ (AL347_25805)	amrZ	PA3367 (AL347_25915)	PA3367	-	24603766
amrZ (AL347_25805)	amrZ	PA3401 (AL347_25725)	PA3401	-	24603766
amrZ (AL347_25805)	amrZ	PA3413 (AL347_25660)	PA3413	-	24603766
amrZ (AL347_25805)	amrZ	rhlR (AL347_25325)	rhlR	-	24603766
amrZ (AL347_25805)	amrZ	ygbB (AL347_24640)	ygbB	-	24603766
amrZ (AL347_25805)	amrZ	PA3951 (AL347_22955)	PA3951	-	24603766
amrZ (AL347_25805)	amrZ	mucE (AL347_22545)	mucE	-	24603766
amrZ (AL347_25805)	amrZ	phzA1 (AL347_21625)	phzA1	-	24603766
amrZ (AL347_25805)	amrZ	PA4222 (AL347_21560)	PA4222	-	24603766
amrZ (AL347_25805)	amrZ	pctC (AL347_10370)	pctC	-	24603766
amrZ (AL347_25805)	amrZ	PA4324 (AL347_10465)	PA4324	-	24603766
amrZ (AL347_25805)	amrZ	PA4683 (AL347_12955)	PA4683	-	24603766
amrZ (AL347_25805)	amrZ	PA4980 (AL347_14525)	PA4980	-	24603766
amrZ (AL347_25805)	amrZ	PA5138 (AL347_15345)	PA5138	-	24603766
amrZ (AL347_25805)	amrZ	nirS (AL347_20430)	nirS	+	26755627
amrZ (AL347_25805)	amrZ	prtN (AL347_20930)	prtN	+	26755627
amrZ (AL347_25805)	amrZ	lecA (AL347_31780)	lecA	+	26755627
amrZ (AL347_25805)	amrZ	lecB (AL347_25945)	lecB	+	26755627
amrZ (AL347_25805)	amrZ	nosR (AL347_25775)	nosR	+	26755627
amrZ (AL347_25805)	amrZ	hasAp (AL347_25690)	hasAp	+	26755627
amrZ (AL347_25805)	amrZ	rhlA (AL347_25315)	rhlA	+	26755627
amrZ (AL347_25805)	amrZ	hemH (AL347_12795)	hemH	-	26755627
amrZ (AL347_25805)	amrZ	PA0149 (AL347_18525)	PA0149	?	29729420
amrZ (AL347_25805)	amrZ	PA1363 (AL347_06280)	PA1363	?	29729420
amrZ (AL347_25805)	amrZ	sigX (AL347_03799)	sigX	?	29729420
amrZ (AL347_25805)	amrZ	femI (AL347_03050)	femI	?	29729420
amrZ (AL347_25805)	amrZ	fpvI (AL347_33080)	fpvI	?	29729420
amrZ (AL347_25805)	amrZ	pvdS (AL347_32895)	pvdS	?	29729420
amrZ (AL347_25805)	amrZ	PA0165 (AL347_18610)	PA0165	?	31270321
amrZ (AL347_25805)	amrZ	PA0428 (AL347_19960)	PA0428	?	31270321
amrZ (AL347_25805)	amrZ	surA (AL347_20850)	surA	?	31270321
amrZ (AL347_25805)	amrZ	phdA (AL347_10095)	phdA	?	31270321
amrZ (AL347_25805)	amrZ	putP (AL347_09600)	putP	?	31270321
amrZ (AL347_25805)	amrZ	ampDh3 (AL347_09475)	ampDh3	?	31270321
amrZ (AL347_25805)	amrZ	PA0947 (AL347_08475)	PA0947	?	31270321

amrZ (AL347_25805)	amrZ	pauA (AL347_08110)	pauA	?	31270321
amrZ (AL347_25805)	amrZ	shaB (AL347_07905)	shaB	?	31270321
amrZ (AL347_25805)	amrZ	roeA (AL347_07640)	roeA	?	31270321
amrZ (AL347_25805)	amrZ	rhlC (AL347_07505)	rhlC	?	31270321
amrZ (AL347_25805)	amrZ	PA1159 (AL347_07340)	PA1159	?	31270321
amrZ (AL347_25805)	amrZ	PA1211 (AL347_07075)	PA1211	?	31270321
amrZ (AL347_25805)	amrZ	cobO (AL347_06750)	cobO	?	31270321
amrZ (AL347_25805)	amrZ	PA1383 (AL347_06185)	PA1383	?	31270321
amrZ (AL347_25805)	amrZ	PA1473 (AL347_05400)	PA1473	?	31270321
amrZ (AL347_25805)	amrZ	PA1736 (AL347_04015)	PA1736	?	31270321
amrZ (AL347_25805)	amrZ	PA2229 (AL347_00925)	PA2229	?	31270321
amrZ (AL347_25805)	amrZ	PA2471 (AL347_32665)	PA2471	?	31270321
amrZ (AL347_25805)	amrZ	PA2504 (AL347_32455)	PA2504	?	31270321
amrZ (AL347_25805)	amrZ	cobH (AL347_28360)	cobH	?	31270321
amrZ (AL347_25805)	amrZ	xcpP (AL347_27320)	xcpP	?	31270321
amrZ (AL347_25805)	amrZ	PA3110 (AL347_27290)	PA3110	?	31270321
amrZ (AL347_25805)	amrZ	asd (AL347_27255)	asd	?	31270321
amrZ (AL347_25805)	amrZ	wbpM (AL347_27110)	wbpM	?	31270321
amrZ (AL347_25805)	amrZ	grxD (AL347_25145)	grxD	?	31270321
amrZ (AL347_25805)	amrZ	PA3592 (AL347_24825)	PA3592	?	31270321
amrZ (AL347_25805)	amrZ	dnaE (AL347_24575)	dnaE	?	31270321
amrZ (AL347_25805)	amrZ	PA3836 (AL347_23560)	PA3836	?	31270321
amrZ (AL347_25805)	amrZ	PA3854 (AL347_23465)	PA3854	?	31270321
amrZ (AL347_25805)	amrZ	PA3959 (AL347_22915)	PA3959	?	31270321
amrZ (AL347_25805)	amrZ	PA4392 (AL347_10815)	PA4392	?	31270321
amrZ (AL347_25805)	amrZ	magE (AL347_11305)	magE	?	31270321
amrZ (AL347_25805)	amrZ	oprJ (AL347_12480)	oprJ	?	31270321
amrZ (AL347_25805)	amrZ	PA4987 (AL347_14565)	PA4987	?	31270321
amrZ (AL347_25805)	amrZ	mutY (AL347_15400)	mutY	?	31270321
amrZ (AL347_25805)	amrZ	pstC (AL347_16600)	pstC	?	31270321
amrZ (AL347_25805)	amrZ	gbdR (AL347_16690)	gbdR	?	31270321
amrZ (AL347_25805)	amrZ	fiuI (AL347_20195)	fiuI	-	24603766, 29729420
amrZ (AL347_25805)	amrZ	PA0102 (AL347_18280)	PA0102	+	24603766, 31270321
amrZ (AL347_25805)	amrZ	siaA (AL347_18645)	siaA	-	24603766, 31270321
amrZ (AL347_25805)	amrZ	PA1069 (AL347_07835)	PA1069	+	24603766, 31270321
amrZ (AL347_25805)	amrZ	PA2867 (AL347_28550)	PA2867	-	24603766, 31270321
amrZ (AL347_25805)	amrZ	pelB (AL347_27530)	pelB	+	24603766, 31270321
amrZ (AL347_25805)	amrZ	amrZ (AL347_25805)	amrZ	-	24603766, 31270321

amrZ (AL347_25805)	amrZ	algD (AL347_25110)	algD	+	24603766, 31270321
amrZ (AL347_25805)	amrZ	PA3691 (AL347_24320)	PA3691	-	24603766, 31270321
amrZ (AL347_25805)	amrZ	PA3722 (AL347_24165)	PA3722	-	24603766, 31270321
amrZ (AL347_25805)	amrZ	pchG (AL347_21550)	pchG	-	24603766, 31270321
amrZ (AL347_25805)	amrZ	flp (AL347_10365)	flp	-	24603766, 31270321
amrZ (AL347_25805)	amrZ	PA4677 (AL347_12925)	PA4677	-	24603766, 31270321
amrZ (AL347_25805)	amrZ	ctpL (AL347_13830)	ctpL	-	24603766, 31270321
amrZ (AL347_25805)	amrZ	dctP (AL347_09075)	dctP	-	24603766, 31270321
amrZ (AL347_25805)	amrZ	gcbA (AL347_13825)	gcbA	-	26755627, 31270321
anr (AL347_05030)	anr	dnr (AL347_20470)	dnr	+	17400734
anr (AL347_05030)	anr	anr (AL347_05030)	anr	+	17400734
anr (AL347_05030)	anr	narG (AL347_23365)	narG	+	17400734
anr (AL347_05030)	anr	narX (AL347_23350)	narX	+	17400734
anr (AL347_05030)	anr	aroE (AL347_17875)	aroE	+	18440972
anr (AL347_05030)	anr	PA0521 (AL347_20440)	PA0521	+	18440972
anr (AL347_05030)	anr	PA0522 (AL347_20445)	PA0522	+	18440972
anr (AL347_05030)	anr	murI (AL347_12830)	murI	d	18440972
anr (AL347_05030)	anr	moeB (AL347_12835)	moeB	d	18440972
anr (AL347_05030)	anr	hemK (AL347_12840)	hemK	d	18440972
anr (AL347_05030)	anr	prfA (AL347_12845)	prfA	+	18440972
anr (AL347_05030)	anr	narI (AL347_23380)	narI	+	18832311
anr (AL347_05030)	anr	narJ (AL347_23375)	narJ	+	18832311
anr (AL347_05030)	anr	narH (AL347_23370)	narH	+	18832311
anr (AL347_05030)	anr	hemF (AL347_17870)	hemF	+	18974177
anr (AL347_05030)	anr	oprE (AL347_19240)	oprE	+	18974177
anr (AL347_05030)	anr	nirS (AL347_20430)	nirS	+	18974177
anr (AL347_05030)	anr	nirQ (AL347_20435)	nirQ	+	18974177
anr (AL347_05030)	anr	norC (AL347_20450)	norC	+	18974177
anr (AL347_05030)	anr	norB (AL347_20455)	norB	+	18974177
anr (AL347_05030)	anr	hemN (AL347_05020)	hemN	+	18974177
anr (AL347_05030)	anr	hcnA (AL347_01585)	hcnA	+	18974177
anr (AL347_05030)	anr	hcnB (AL347_01580)	hcnB	+	18974177
anr (AL347_05030)	anr	hcnC (AL347_01575)	hcnC	+	18974177
anr (AL347_05030)	anr	nark2 (AL347_23360)	nark2	+	18974177
anr (AL347_05030)	anr	nark1 (AL347_23355)	nark1	+	18974177
anr (AL347_05030)	anr	PA4352 (AL347_10605)	PA4352	+	18974177
anr (AL347_05030)	anr	hemA (AL347_12850)	hemA	+	18974177
anr (AL347_05030)	anr	azu (AL347_14220)	azu	+	18974177

anr (AL347_05030)	anr	coxB (AL347_18300)	coxB	-	19930444
anr (AL347_05030)	anr	coxA (AL347_18305)	coxA	-	19930444
anr (AL347_05030)	anr	ccoP2 (AL347_04965)	ccoP2	+	19930444
anr (AL347_05030)	anr	ccoQ2 (AL347_04960)	ccoQ2	+	19930444
anr (AL347_05030)	anr	ccoO2 (AL347_04955)	ccoO2	+	19930444
anr (AL347_05030)	anr	ccoN2 (AL347_04950)	ccoN2	+	19930444
anr (AL347_05030)	anr	cioB (AL347_23070)	cioB	-	19930444
anr (AL347_05030)	anr	aer (AL347_04935)	aer	+	24413814
anr (AL347_05030)	anr	PA1746 (AL347_03965)	PA1746	+	24413814
anr (AL347_05030)	anr	arcD (AL347_15575)	arcD	+	24413814
anr (AL347_05030)	anr	arcA (AL347_15580)	arcA	+	24413814
anr (AL347_05030)	anr	arcB (AL347_15585)	arcB	+	24413814
anr (AL347_05030)	anr	arcC (AL347_15590)	arcC	+	24413814
anr (AL347_05030)	anr	plcH (AL347_09290)	plcH	-	25073853
anr (AL347_05030)	anr	PA1476 (AL347_05385)	PA1476 (ccmB, helB, cyt10, cycW	+	25073853
anr (AL347_05030)	anr	PA1571 (AL347_04875)	PA1571	+	25073853
anr (AL347_05030)	anr	PA1300 (AL347_06610)	PA1300 (hxuI)	?	29729420
anr (AL347_05030)	anr	sbrI (AL347_28405)	sbrI	?	29729420
anr (AL347_05030)	anr	fecI (AL347_23230)	fecI	?	29729420
anr (AL347_05030)	anr	glpT (AL347_15915)	glpT	+	30186264
anr (AL347_05030)	anr	adhA (AL347_16930)	adhA	-	31527114
anr (AL347_05030)	anr	narL (AL347_23345)	narL	+	17400734, 21873408
anr (AL347_05030)	anr	cioA (AL347_23065)	cioA	-	19930444, 25073853
anr (AL347_05030)	anr	coIII (AL347_18315)	coIII	-	27242034*
anr (AL347_05030)	anr	PA0526 (AL347_20465)	PA0526	?	27242034*
anr (AL347_05030)	anr	PA3928 (AL347_23075)	PA3928	?	27242034*
anr (AL347_05030)	anr	PA3309 (AL347_26210)	PA3309	?	27242034*
anr (AL347_05030)	anr	PA2127 (AL347_01955)	PA2127 (cgrA)	?	27242034*
anr (AL347_05030)	anr	PA2126 (AL347_01965)	PA2126 (cgrC)	?	27242034*
ansR (AL347_00810)	ansR	ansP (AL347_33775)	ansP	+	29293081
ansR (AL347_00810)	ansR	ansA (AL347_00800)	ansA	+	29293081
antR (AL347_32110)	antR	antB (AL347_32095)	antB	+	22336763
antR (AL347_32110)	antR	antC (AL347_32090)	antC	+	22336763
antR (AL347_32110)	antR	antR (AL347_32110)	antR	+	22609066
antR (AL347_32110)	antR	antA (AL347_32100)	antA	+	22609066
argR (AL347_09030)	argR	PA1819 (AL347_03545)	PA1819	+	15175299
argR (AL347_09030)	argR	aotO (AL347_09040)	aotO	+	18440972
argR (AL347_09030)	argR	PA4754 (AL347_13360)	PA4754	-	18440972

argR (AL347_09030)	argR	greA (AL347_13365)	greA	-	18440972
argR (AL347_09030)	argR	PA0328 (AL347_19440)	PA0328 (aaaA)	+	18974177
argR (AL347_09030)	argR	argR (AL347_09030)	argR	+	18974177
argR (AL347_09030)	argR	aruC (AL347_09020)	aruC	+	18974177
argR (AL347_09030)	argR	aruF (AL347_09015)	aruF	+	18974177
argR (AL347_09030)	argR	aruG (AL347_09010)	aruG	+	18974177
argR (AL347_09030)	argR	aruD (AL347_09005)	aruD	+	18974177
argR (AL347_09030)	argR	aruB (AL347_09000)	aruB	+	18974177
argR (AL347_09030)	argR	PA0900 (AL347_08995)	PA0900	+	18974177
argR (AL347_09030)	argR	aruE (AL347_08990)	aruE	+	18974177
argR (AL347_09030)	argR	braZ (AL347_02755)	braZ	+	18974177
argR (AL347_09030)	argR	PA2042 (AL347_02395)	PA2042	+	18974177
argR (AL347_09030)	argR	gdhB (AL347_27520)	gdhB	+	18974177
argR (AL347_09030)	argR	argG (AL347_25185)	argG	-	18974177
argR (AL347_09030)	argR	argF (AL347_25125)	argF	-	18974177
argR (AL347_09030)	argR	PA3538 (AL347_25120)	PA3538	-	18974177
argR (AL347_09030)	argR	PA3934 (AL347_23045)	PA3934	+	18974177
argR (AL347_09030)	argR	gdhA (AL347_12430)	gdhA	-	18974177
argR (AL347_09030)	argR	carB (AL347_13370)	carB	-	18974177
argR (AL347_09030)	argR	PA4757 (AL347_13375)	PA4757	-	18974177
argR (AL347_09030)	argR	carA (AL347_13380)	carA	-	18974177
argR (AL347_09030)	argR	gltD (AL347_14820)	gltD	-	18974177
argR (AL347_09030)	argR	gltB (AL347_14825)	gltB	-	18974177
argR (AL347_09030)	argR	PA5152 (AL347_15480)	PA5152	+	18974177
argR (AL347_09030)	argR	PA5153 (AL347_15485)	PA5153	+	18974177
argR (AL347_09030)	argR	PA5154 (AL347_15490)	PA5154	+	18974177
argR (AL347_09030)	argR	PA5155 (AL347_15495)	PA5155	+	18974177
argR (AL347_09030)	argR	ldcA (AL347_03550)	ldcA	+	20833801
argR (AL347_09030)	argR	arcD (AL347_15575)	arcD	+	24413814
argR (AL347_09030)	argR	arcA (AL347_15580)	arcA	+	24413814
argR (AL347_09030)	argR	arcB (AL347_15585)	arcB	+	24413814
argR (AL347_09030)	argR	arcC (AL347_15590)	arcC	+	24413814
argR (AL347_09030)	argR	aotJ (AL347_09055)	aotJ	+	26967762
argR (AL347_09030)	argR	aotQ (AL347_09050)	aotQ	+	26967762
argR (AL347_09030)	argR	aotM (AL347_09045)	aotM	+	26967762
argR (AL347_09030)	argR	aotP (AL347_09035)	aotP	+	26967762
argR (AL347_09030)	argR	dauB (AL347_23420)	dauB	+	26967762
argR (AL347_09030)	argR	dauA (AL347_23415)	dauA	+	26967762

argR (AL347_09030)	argR	dauR (AL347_23410)	dauR	+	26967762
argR (AL347_09030)	argR	lysP (AL347_12650)	lysP	-	26967762
argR (AL347_09030)	argR	alr (AL347_14260)	alr	-	26967762
argR (AL347_09030)	argR	aruH (AL347_14505)	aruH	-	26967762
argR (AL347_09030)	argR	PA0891 (AL347_09040)	PA0891	+	18440972*
armR (AL347_24180)	armR	mexR (AL347_19940)	mexR	-	22393435
armZ (AL347_17165)	armZ	algD (AL347_25110)	algD	+	19168621
armZ (AL347_17165)	armZ	mexY (AL347_02515)	mexY	+	31527038
armZ (AL347_17165)	armZ	mexX (AL347_02510)	mexX	+	31527038
artR (AL347_12465)	artR	exsC (AL347_04150)	exsC	-	30949153
artR (AL347_12465)	artR	exsE (AL347_04145)	exsE	-	30949153
artR (AL347_12465)	artR	exsB (AL347_04140)	exsB	-	30949153
artR (AL347_12465)	artR	exsA (AL347_04135)	exsA	-	30949153
artR (AL347_12465)	artR	exsD (AL347_04130)	exsD	-	30949153
artR (AL347_12465)	artR	pscB (AL347_04125)	pscB	-	30949153
artR (AL347_12465)	artR	pscC (AL347_04120)	pscC	-	30949153
artR (AL347_12465)	artR	pscD (AL347_04115)	pscD	-	30949153
artR (AL347_12465)	artR	pscE (AL347_04110)	pscE	-	30949153
artR (AL347_12465)	artR	pscF (AL347_04105)	pscF	-	30949153
artR (AL347_12465)	artR	pscG (AL347_04100)	pscG	-	30949153
artR (AL347_12465)	artR	pscH (AL347_04095)	pscH	-	30949153
artR (AL347_12465)	artR	pscI (AL347_04090)	pscI	-	30949153
artR (AL347_12465)	artR	pscL (AL347_04075)	pscL	-	30949153
artR (AL347_12465)	artR	exoS (AL347_23535)	exoS	-	30949153
artR (AL347_12465)	artR	pscJ (AL347_04085)	pscJ	-	30949153, 30949153
artR (AL347_12465)	artR	exoY (AL347_01600)	exoY	-	30949153, 30949153
asrA (AL347_09620)	asrA	fhpR (AL347_30085)	fhpR	-	18043907
asrA (AL347_09620)	asrA	fhp (AL347_30090)	fhp	+	19767835
asrA (AL347_09620)	asrA	mexY (AL347_02515)	mexY	+	21357290
asrA (AL347_09620)	asrA	mexX (AL347_02510)	mexX	+	21357290
atuR (AL347_28460)	atuR	atuA (AL347_28455)	atuA	-	20487029
atuR (AL347_28460)	atuR	atuB (AL347_28450)	atuB	-	20487029
atuR (AL347_28460)	atuR	atuC (AL347_28445)	atuC	-	20487029
atuR (AL347_28460)	atuR	atuD (AL347_28440)	atuD	-	20487029
atuR (AL347_28460)	atuR	atuE (AL347_28435)	atuE	-	20487029
atuR (AL347_28460)	atuR	atuF (AL347_28430)	atuF	-	20487029
atuR (AL347_28460)	atuR	atuG (AL347_28425)	atuG	-	20487029
atuR (AL347_28460)	atuR	atuH (AL347_28420)	atuH	-	20487029

bauR (AL347_18440)	bauR	bauC (AL347_18425)	bauC	+	21622750
bauR (AL347_18440)	bauR	bauB (AL347_18430)	bauB	+	21622750
bauR (AL347_18440)	bauR	bauA (AL347_18435)	bauA	+	21622750
bauR (AL347_18440)	bauR	bauR (AL347_18440)	bauR	+	21622750
bauR (AL347_18440)	bauR	pauA3 (AL347_04910)	pauA3	+	21622750
betI (AL347_16655)	betI	betA (AL347_16645)	betA	-	29293081
betI (AL347_16655)	betI	betB (AL347_16650)	betB	-	29293081
betI (AL347_16655)	betI	gbdR (AL347_16690)	gbdR	-	29293081
bexR (AL347_32855)	bexR	PA0572 (AL347_20700)	PA0572	+	20041030
bexR (AL347_32855)	bexR	PA1202 (AL347_07120)	PA1202	+	20041030
bexR (AL347_32855)	bexR	PA1203 (AL347_07115)	PA1203	+	20041030
bexR (AL347_32855)	bexR	PA1204 (AL347_07110)	PA1204	+	20041030
bexR (AL347_32855)	bexR	PA1205 (AL347_07105)	PA1205	+	20041030
bexR (AL347_32855)	bexR	aprA (AL347_06875)	aprA	+	20041030
bexR (AL347_32855)	bexR	bexR (AL347_32855)	bexR	+	20041030
bfiS (AL347_21695)	bfiS	gacS (AL347_08580)	gacS	+	19936057
bfiS (AL347_21695)	bfiS	gacA (AL347_30505)	gacA	+	19936057
bfiS (AL347_21695)	bfiS	cafA (AL347_11250)	cafA	+	20656909
bfmR (AL347_22195)	bfmR	phdA (AL347_10095)	phdA	+	21696457
bfmR (AL347_22195)	bfmR	rhlR (AL347_25325)	rhlR	-	25166864
birA (AL347_21250)	birA	bioB (AL347_20330)	bioB	-	27242034*
birA (AL347_21250)	birA	bioF (AL347_20335)	bioF	-	27242034*
birA (AL347_21250)	birA	bioH (AL347_20340)	bioH	-	27242034*
birA (AL347_21250)	birA	bioC (AL347_20345)	bioC	-	27242034*
birA (AL347_21250)	birA	bioD (AL347_20350)	bioD	-	27242034*
bqsR (AL347_30130)	bqsR/PA2657	phnA (AL347_08200)	phnA	+	27242034*, 18974177*, 22587778
bqsR (AL347_30130)	bqsR/PA2657	pqsA (AL347_08225)	pqsA	+	27242034*, 18974177*, 22587778
bqsR (AL347_30130)	bqsR/PA2657	rhlA (AL347_25315)	rhlA	+	27242034*, 18974177*, 22587778
bqsR (AL347_30130)	bqsR/PA2657	rhlB (AL347_25320)	rhlB	+	27242034*, 18974177*, 22587778
bqsR (AL347_30130)	bqsS/PA2656	phnA (AL347_08200)	phnA	+	27242034*, 18974177*, 22587778
bqsR (AL347_30130)	bqsS/PA2656	pqsA (AL347_08225)	pqsA	+	27242034*, 18974177*, 22587778
bqsR (AL347_30130)	bqsS/PA2656	rhlA (AL347_25315)	rhlA	+	27242034*, 18974177*, 22587778
bqsR (AL347_30130)	bqsS/PA2656	rhlB (AL347_25320)	rhlB	+	27242034*, 18974177*, 22587778
brlR (AL347_14000)	brlR	oprH (AL347_07245)	oprH	-	23935054
brlR (AL347_14000)	brlR	phoP (AL347_07240)	phoP	-	23935054
brlR (AL347_14000)	brlR	phoQ (AL347_07235)	phoQ	-	23935054
brlR (AL347_14000)	brlR	mexB (AL347_19950)	mexB	+	29180529
brlR (AL347_14000)	brlR	oprM (AL347_19955)	oprM	+	29180529

brlR (AL347_14000)	brlR	bapB (AL347_03245)	bapB	+	29180529
brlR (AL347_14000)	brlR	bapC (AL347_03240)	bapC	+	29180529
brlR (AL347_14000)	brlR	bapD (AL347_03235)	bapD	+	29180529
brlR (AL347_14000)	brlR	mexE (AL347_32510)	mexE	+	29180529
brlR (AL347_14000)	brlR	mexF (AL347_32505)	mexF	+	29180529
brlR (AL347_14000)	brlR	oprN (AL347_32500)	oprN	+	29180529
brlR (AL347_14000)	brlR	brlR (AL347_14000)	brlR	+	29967320
brlR (AL347_14000)	brlR	mexA (AL347_19946)	mexA	+	23687276
cbrA (AL347_13200)	cbrA	rsmA (AL347_08970)	rsmA	+	28699871
cbrB (AL347_13205)	cbrB	spuI (AL347_19265)	spuI	+	18974177
cbrB (AL347_13205)	cbrB	spuA (AL347_19270)	spuA	+	18974177
cbrB (AL347_13205)	cbrB	spuB (AL347_19275)	spuB	+	18974177
cbrB (AL347_13205)	cbrB	spuC (AL347_19280)	spuC	+	18974177
cbrB (AL347_13205)	cbrB	spuD (AL347_19285)	spuD	+	18974177
cbrB (AL347_13205)	cbrB	spuE (AL347_19290)	spuE	+	18974177
cbrB (AL347_13205)	cbrB	spuF (AL347_19295)	spuF	+	18974177
cbrB (AL347_13205)	cbrB	spuG (AL347_19300)	spuG	+	18974177
cbrB (AL347_13205)	cbrB	spuH (AL347_19305)	spuH	+	18974177
cbrB (AL347_13205)	cbrB	aotJ (AL347_09055)	aotJ	+	18974177
cbrB (AL347_13205)	cbrB	aotQ (AL347_09050)	aotQ	+	18974177
cbrB (AL347_13205)	cbrB	aotM (AL347_09045)	aotM	+	18974177
cbrB (AL347_13205)	cbrB	aotP (AL347_09035)	aotP	+	18974177
cbrB (AL347_13205)	cbrB	cbrB (AL347_13205)	cbrB	+	18974177
cbrB (AL347_13205)	cbrB	pmrA (AL347_13475)	pmrA	-	21169488
cbrB (AL347_13205)	cbrB	lipA (AL347_28575)	lipA	+	21478360
cbrB (AL347_13205)	cbrB	hutU (AL347_15145)	hutU	+	21478360
cbrB (AL347_13205)	cbrB	rsmA (AL347_08970)	rsmA	+	28699871
cbrB (AL347_13205)	cbrB	hutI (AL347_15105)	hutI	?	27242034*
cbrB (AL347_13205)	cbrB	hutH (AL347_15135)	hutH	?	27242034*
cbrB (AL347_13205)	cbrB	exoS (AL347_23535)	exoS	?	27242034*
cbrB (AL347_13205)	cbrB	hutG (AL347_15100)	hutG	?	27242034*
cbrB (AL347_13205)	cbrB	hutC (AL347_15170)	hutC	?	27242034*
cbrB (AL347_13205)	cbrB	PA5104 (AL347_15165)	PA5104	?	27242034*
cbrB (AL347_13205)	cbrB	PA5101 (AL347_15150)	PA5101	?	27242034*
cbrB (AL347_13205)	cbrB	PA5102 (AL347_15155)	PA5102	?	27242034*
cbrB (AL347_13205)	cbrB	PA5099 (AL347_15140)	PA5099	?	27242034*
cbrB (AL347_13205)	cbrB	PA5093 (AL347_15110)	PA5093	?	27242034*
cbrB (AL347_13205)	cbrB	PA5097 (AL347_15130)	PA5097	?	27242034*

cbrB (AL347_13205)	cbrB	PA5096 (AL347_15125)	PA5096	?	27242034*
cbrB (AL347_13205)	cbrB	PA5095 (AL347_15120)	PA5095	?	27242034*
cbrB (AL347_13205)	cbrB	PA5094 (AL347_15115)	PA5094	?	27242034*
cdhR (AL347_16735)	cdhR	hocS (AL347_16710)	hocS	+	29435492
cdhR (AL347_16735)	cdhR	cdhB (AL347_16715)	cdhB	+	29435492
cdhR (AL347_16735)	cdhR	cdhA (AL347_16720)	cdhA	+	29435492
cdhR (AL347_16735)	cdhR	cdhC (AL347_16725)	cdhC	+	29435492
cdhR (AL347_16735)	cdhR	caiX (AL347_16730)	caiX	+	29435492
cdhR (AL347_16735)	cdhR	cdhR (AL347_16735)	cdhR	-	29435492
cdhR (AL347_16735)	PSPA7_RS29445	PSPA7_RS29440 (AL347_1673[illegible]	PSPA7_RS29440 (PSPA7_6174)	?	27242034*
cdhR (AL347_16735)	PSPA7_RS29445	cdhC (AL347_16725)	PSPA7_RS29435 (PSPA7_6173)	?	27242034*
cdpR (AL347_30490)	cdpR	cerN (AL347_09285)	cerN	?	31270321
cdpR (AL347_30490)	cdpR	PA1333 (AL347_06435)	PA1333	?	31270321
cdpR (AL347_30490)	cdpR	lpdV (AL347_00815)	lpdV	?	31270321
cdpR (AL347_30490)	cdpR	cdpR (AL347_30490)	cdpR	?	31270321
cdpR (AL347_30490)	cdpR	amiR (AL347_25935)	amiR	?	31270321
cdpR (AL347_30490)	cdpR	PA3638 (AL347_24585)	PA3638	?	31270321
cdpR (AL347_30490)	cdpR	PA3854 (AL347_23465)	PA3854	?	31270321
cdpR (AL347_30490)	cdpR	PA4340 (AL347_10545)	PA4340	?	31270321
cdpR (AL347_30490)	cdpR	PA4676 (AL347_12920)	PA4676	?	31270321
cdpR (AL347_30490)	cdpR	mutY (AL347_15400)	mutY	?	31270321
cifR (AL347_28225)	cifR	cifR (AL347_28225)	cifR	-	22843844
cifR (AL347_28225)	cifR	morB (AL347_28220)	morB	-	22843844
cifR (AL347_28225)	cifR	cif (AL347_28210)	cif	-	25112474
cifR (AL347_28225)	cifR	PA2933 (AL347_28215)	PA2933	?	27242034*
clpP (AL347_03640)	clpP	algU (AL347_09705)	algU	-	18599839
clpX (AL347_03635)	clpX	algU (AL347_09705)	algU	-	18599839
cmpX (AL347_03805)	cmpX	sigX (AL347_03799)	sigX	+	29063141
cmpX (AL347_03805)	cmpX	oprF (AL347_03795)	oprF	+	29063141
cmpX (AL347_03805)	cmpX	exoS (AL347_23535)	exoS	+	29063141
cmrA (AL347_02370)	cmrA	mexE (AL347_32510)	mexE	+	28507116
cmrA (AL347_02370)	cmrA	mexF (AL347_32505)	mexF	+	28507116
cmrA (AL347_02370)	cmrA	oprN (AL347_32500)	oprN	+	28507116
copR (AL347_28855)	copR	PA2524 (AL347_32040)	PA2524 (czcS)	?	27242034*
copR (AL347_28855)	copR	czcR (AL347_32045)	czcR	?	27242034*
copR (AL347_28855)	copR	czcB (AL347_32055)	czcB	?	27242034*
copR (AL347_28855)	copR	czcC (AL347_32050)	czcC	?	27242034*
copR (AL347_28855)	copR	czcA (AL347_32060)	czcA	?	27242034*

copR (AL347_28855)	copR	ptrA (AL347_28860)	ptrA	?	27242034*
cpxR (AL347_00500)	cpxR	mexB (AL347_19950)	mexB	+	30289929
cpxR (AL347_00500)	cpxR	oprM (AL347_19955)	oprM	+	30289929
crc (AL347_16415)	crc	PA0884 (AL347_09075)	PA0884	-	23253107
crc (AL347_16415)	crc	dctQ (AL347_09070)	dctQ	-	23253107
crc (AL347_16415)	crc	dctM (AL347_09065)	dctM	-	23253107
crc (AL347_16415)	crc	dctR (AL347_07225)	dctR	-	23253107
crc (AL347_16415)	crc	nirS (AL347_20430)	nirS	+	23292701
crc (AL347_16415)	crc	ptrB (AL347_20940)	ptrB	-	23292701
crc (AL347_16415)	crc	prpC (AL347_09535)	prpC	-	23292701
crc (AL347_16415)	crc	acsA (AL347_09060)	acsA	-	23292701
crc (AL347_16415)	crc	fimL (AL347_03530)	fimL	-	23292701
crc (AL347_16415)	crc	ptrC (AL347_32590)	ptrC	+	23292701
crc (AL347_16415)	crc	gltR (AL347_00565)	gltR	-	23292701
crc (AL347_16415)	crc	rpoS (AL347_24665)	rpoS	-	23292701
crc (AL347_16415)	crc	cpdA (AL347_14470)	cpdA	-	23292701
crc (AL347_16415)	crc	PA5530 (AL347_17470)	PA5530	+	26114434
crc (AL347_16415)	crc	zwf (AL347_26905)	zwf	?	30429516
crc (AL347_16415)	crc	bkdB (AL347_00820)	bkdB	?	30429516
crc (AL347_16415)	crc	mtlD (AL347_33315)	mtlD	?	30429516
crc (AL347_16415)	crc	bkdA1 (AL347_00830)	bkdA1	?	30429516
crc (AL347_16415)	crc	bkdA2 (AL347_00825)	bkdA2	?	30429516
crc (AL347_16415)	crc	pilB (AL347_11505)	pilB	?	27242034*
crc (AL347_16415)	crc	lpdV (AL347_00815)	lpdV	?	27242034*
cueR (AL347_13485)	cueR	mexP (AL347_25195)	mexP	+	25342258, 24175918*
cueR (AL347_13485)	cueR	PA3920 (AL347_23120)	PA3920 (copA1, yvgX, cueA)	+	24175918*
cueR (AL347_13485)	cueR	mexQ (AL347_25200)	mexQ	+	24175918*
cueR (AL347_13485)	cueR	PA3521 (AL347_25205)	PA3521 (opmE)	+	24175918*
cyaB (AL347_00435)	cyaB	exsA (AL347_04135)	exsA	+	25070741
cysB (AL347_03920)	cysB	alg8 (AL347_25105)	alg8	+	18440972
cysB (AL347_03920)	cysB	alg44 (AL347_25100)	alg44	+	18440972
cysB (AL347_03920)	cysB	algK (AL347_25095)	algK	+	18440972
cysB (AL347_03920)	cysB	algE (AL347_25090)	algE	+	18440972
cysB (AL347_03920)	cysB	algG (AL347_25085)	algG	+	18440972
cysB (AL347_03920)	cysB	algX (AL347_25080)	algX	+	18440972
cysB (AL347_03920)	cysB	algL (AL347_25075)	algL	+	18440972
cysB (AL347_03920)	cysB	algI (AL347_25065)	algI	+	18440972
cysB (AL347_03920)	cysB	algJ (AL347_25060)	algJ	+	18440972

cysB (AL347_03920)	cysB	algF (AL347_25055)	algF	+	18440972
cysB (AL347_03920)	cysB	algA (AL347_25050)	algA	+	18440972
cysB (AL347_03920)	cysB	cysB (AL347_03920)	cysB	-	18974177
cysB (AL347_03920)	cysB	msuC (AL347_33245)	msuC	+	18974177
cysB (AL347_03920)	cysB	msuD (AL347_33240)	msuD	+	18974177
cysB (AL347_03920)	cysB	msuE (AL347_33235)	msuE	+	18974177
cysB (AL347_03920)	cysB	cysH (AL347_03910)	cysH	+	30618115
cysB (AL347_03920)	cysB	algD (AL347_25110)	algD	+	30618115
cysB (AL347_03920)	cysB	retS (AL347_13890)	retS	-	30618115
cysB (AL347_03920)	cysB	atsA (AL347_18700)	atsA	+	10715018, 10717312
cysB (AL347_03920)	cysB	atsB (AL347_18710)	atsB	+	10715018, 10717313
cysB (AL347_03920)	cysB	atsC (AL347_18705)	atsC	+	10715018, 10717314
cysB (AL347_03920)	cysB	atsR (AL347_18715)	atsR	+	10715018, 10717315
cysB (AL347_03920)	cysB	mvfR (AL347_08190)	mvfR	d	25845844, 30618115
cysB (AL347_03920)	cysB	pvdS (AL347_32895)	pvdS	+	30618115, 29729420
cysB (AL347_03920)	cysB	PA2355 (AL347_33245)	PA2355	+	22587778
cysB (AL347_03920)	cysB	PA0185 (AL347_18710)	PA0185	?	27242034*
cysB (AL347_03920)	cysB	aruG (AL347_09010)	aruG	+	22587778
cysB (AL347_03920)	cysB	aruB (AL347_09000)	aruB	+	22587778
cysB (AL347_03920)	cysB	aruC (AL347_09020)	aruC	+	22587778
czcR (AL347_32045)	czcR	lasI (AL347_05605)	lasI	-	22666466
czcR (AL347_32045)	czcR	czcB (AL347_32055)	czcB	+	22666466
czcR (AL347_32045)	czcR	czcA (AL347_32060)	czcA	+	27706108
czcR (AL347_32045)	czcR	czcC (AL347_32050)	czcC	+	27706108
czcR (AL347_32045)	czcR	phzA1 (AL347_21625)	phzA1	-	28400281
dctD (AL347_15555)	dctD	PA0884 (AL347_09075)	PA0884	+	23253107
dctD (AL347_15555)	dctD	dctQ (AL347_09070)	dctQ	+	23253107
dctD (AL347_15555)	dctD	dctM (AL347_09065)	dctM	+	23253107
dctD (AL347_15555)	dctD	dctA (AL347_07215)	dctA	+	23253107
dctR (AL347_07225)	dctR	dctA (AL347_07215)	dctA	-	23253107
ddaR (AL347_07150)	ddaR	ddaH (AL347_07155)	ddaH	+	28167521
deaD (AL347_28690)	deaD	exsA (AL347_04135)	exsA	+	26055113
desT (AL347_14060)	desT	fabA (AL347_04675)	fabA	-	27242034*
desT (AL347_14060)	desT	PA4889 (AL347_14055)	PA4889	-	27242034*
desT (AL347_14060)	desT	desB (AL347_14050)	desB	-	27242034*
desT (AL347_14060)	desT	desT (AL347_14060)	desT	?	27242034*
dguR (AL347_15075)	dguR	dguC (AL347_15060)	dguC	+	25082951
dguR (AL347_15075)	dguR	dguB (AL347_15065)	dguB	+	25082951

dguR (AL347_15075)	dguR	dguA (AL347_15070)	dguA	+	25082951
dhcR (AL347_02620)	dhcR	dhcA (AL347_02615)	dhcA	+	27706108
dhcR (AL347_02620)	dhcR	dhcB (AL347_02610)	dhcB	+	27706108
dnr (AL347_20470)	dnr	nirS (AL347_20430)	nirS	+	17400734
dnr (AL347_20470)	dnr	nirQ (AL347_20435)	nirQ	+	17400734
dnr (AL347_20470)	dnr	norC (AL347_20450)	norC	+	17400734
dnr (AL347_20470)	dnr	norB (AL347_20455)	norB	+	17400734
dnr (AL347_20470)	dnr	narG (AL347_23365)	narG	+	17400734
dnr (AL347_20470)	dnr	aroE (AL347_17875)	aroE	+	18440972
dnr (AL347_20470)	dnr	nirN (AL347_20380)	nirN	+	18440972
dnr (AL347_20470)	dnr	nirE (AL347_20385)	nirE	+	18440972, 22587778
dnr (AL347_20470)	dnr	nirJ (AL347_20390)	nirJ	+	18440972
dnr (AL347_20470)	dnr	nirH (AL347_20395)	nirH	+	18440972
dnr (AL347_20470)	dnr	nirG (AL347_20400)	nirG	+	18440972
dnr (AL347_20470)	dnr	nirL (AL347_20405)	nirL	+	18440972
dnr (AL347_20470)	dnr	nirF (AL347_20415)	nirF	+	18440972
dnr (AL347_20470)	dnr	nirC (AL347_20420)	nirC	+	18440972
dnr (AL347_20470)	dnr	nirM (AL347_20425)	nirM	+	18440972
dnr (AL347_20470)	dnr	nirD (AL347_03780)	nirD	+	18440972
dnr (AL347_20470)	dnr	hemF (AL347_17870)	hemF	+	18974177
dnr (AL347_20470)	dnr	dnr (AL347_20470)	dnr	+	18974177
dnr (AL347_20470)	dnr	anr (AL347_05030)	anr	+	18974177
dnr (AL347_20470)	dnr	hemN (AL347_05020)	hemN	+	18974177
dnr (AL347_20470)	dnr	nosR (AL347_25775)	nosR	+	18974177
dnr (AL347_20470)	dnr	nosZ (AL347_25770)	nosZ	+	18974177
dnr (AL347_20470)	dnr	nosD (AL347_25765)	nosD	+	18974177
dnr (AL347_20470)	dnr	nosF (AL347_25760)	nosF	+	18974177
dnr (AL347_20470)	dnr	nosY (AL347_25755)	nosY	+	18974177
dnr (AL347_20470)	dnr	nosL (AL347_25750)	nosL	+	18974177
dnr (AL347_20470)	dnr	narI (AL347_23380)	narI	+	18974177
dnr (AL347_20470)	dnr	narJ (AL347_23375)	narJ	+	18974177
dnr (AL347_20470)	dnr	narH (AL347_23370)	narH	+	18974177
dnr (AL347_20470)	dnr	nark2 (AL347_23360)	nark2	+	18974177
dnr (AL347_20470)	dnr	nark1 (AL347_23355)	nark1	+	18974177
dnr (AL347_20470)	dnr	narX (AL347_23350)	narX	+	18974177
dnr (AL347_20470)	dnr	narL (AL347_23345)	narL	+	18974177
dnr (AL347_20470)	dnr	hemA (AL347_12850)	hemA	+	18974177
erbR (AL347_02720)	agmR	eraS (AL347_02715)	exaD	+	27242034*

erbR (AL347_02720)	agmR	eraR (AL347_02710)	exaE	+	27242034*
erdR (AL347_24760)	erdR	acsA (AL347_09060)	acsA	+	20482591
esrC (AL347_12475)	esrC	oprJ (AL347_12480)	oprJ	-	25143244
esrC (AL347_12475)	esrC	mexD (AL347_12485)	mexD	-	25143244
esrC (AL347_12475)	esrC	mexC (AL347_12490)	mexC	-	25143244
exsA (AL347_04135)	exsA	pscB (AL347_04125)	pscB	+	18440972
exsA (AL347_04135)	exsA	pscC (AL347_04120)	pscC	+	18440972
exsA (AL347_04135)	exsA	pscD (AL347_04115)	pscD	+	18440972
exsA (AL347_04135)	exsA	pscE (AL347_04110)	pscE	+	18440972
exsA (AL347_04135)	exsA	pscF (AL347_04105)	pscF	+	18440972
exsA (AL347_04135)	exsA	pscG (AL347_04100)	pscG	+	18440972
exsA (AL347_04135)	exsA	pscH (AL347_04095)	pscH	+	18440972
exsA (AL347_04135)	exsA	pscI (AL347_04090)	pscI	+	18440972
exsA (AL347_04135)	exsA	pscJ (AL347_04085)	pscJ	+	18440972
exsA (AL347_04135)	exsA	pscK (AL347_04080)	pscK	+	18440972
exsA (AL347_04135)	exsA	pscL (AL347_04075)	pscL	+	18440972
exsA (AL347_04135)	exsA	PA3842 (AL347_23525)	PA3842 (spcS, orf1)	+	18440972
exsA (AL347_04135)	exsA	exoT (AL347_17990)	exoT	+	23292701
exsA (AL347_04135)	exsA	exsC (AL347_04150)	exsC	+	23292701
exsA (AL347_04135)	exsA	exsE (AL347_04145)	exsE	+	23292701
exsA (AL347_04135)	exsA	exsA (AL347_04135)	exsA	+	23292701
exsA (AL347_04135)	exsA	exsD (AL347_04130)	exsD	+	23292701
exsA (AL347_04135)	exsA	exoS (AL347_23535)	exoS	+	23292701
exsA (AL347_04135)	exsA	PA0456 (AL347_20105)	PA0456	?	31270321
exsA (AL347_04135)	exsA	surA (AL347_20850)	surA	?	31270321
exsA (AL347_04135)	exsA	pqsA (AL347_08225)	pqsA	?	31270321
exsA (AL347_04135)	exsA	pqsB (AL347_08220)	pqsB	?	31270321
exsA (AL347_04135)	exsA	pqsC (AL347_08215)	pqsC	?	31270321
exsA (AL347_04135)	exsA	pqsD (AL347_08210)	pqsD	?	31270321
exsA (AL347_04135)	exsA	pqsE (AL347_08205)	pqsE	?	31270321
exsA (AL347_04135)	exsA	PA2004 (AL347_02590)	PA2004	?	31270321
exsA (AL347_04135)	exsA	exsB (AL347_04140)	exsB	+	23292701, 31270321
exsA (AL347_04135)	exsA	PA3843 (AL347_23521)	PA3843	+	18440972*
exsC (AL347_04150)	exsC	exsD (AL347_04130)	exsD	-	23292701
exsD (AL347_04130)	exsD	exsA (AL347_04135)	exsA	-	23292701
exsE (AL347_04145)	exsE	exsC (AL347_04150)	exsC	-	23292701
fecI (AL347_23230)	fecI	dguA (AL347_15070)	dguA	+	Homology from 25780925
fecI (AL347_23230)	fecI	dguB (AL347_15065)	dguB	+	Homology from 25780925

fecI (AL347_23230)	fecI	dguC (AL347_15060)	dguC	+	Homology from 25780925
fecI (AL347_23230)	fecI	PA4986 (AL347_14560)	PA4986	+	Homology from 25780925
fecI (AL347_23230)	fecI	PA4881 (AL347_14015)	PA4881	+	Homology from 25780925
fecI (AL347_23230)	fecI	PA4200 (AL347_21680)	PA4200	+	Homology from 25780925
fecI (AL347_23230)	fecI	PA4199 (AL347_21685)	PA4199	+	Homology from 25780925
fecI (AL347_23230)	fecI	PA4198 (AL347_21690)	PA4198	+	Homology from 25780925
fecI (AL347_23230)	fecI	PA4067 (AL347_22375)	PA4067 (oprG, yciD, ompW)	+	Homology from 25780925
fecI (AL347_23230)	fecI	PA3610 (AL347_24730)	PA3610 (potD)	+	Homology from 25780925
fecI (AL347_23230)	fecI	hasR (AL347_25685)	hasR	+	Homology from 25780925
fecI (AL347_23230)	fecI	pvdA (AL347_33085)	pvdA	+	Homology from 25780925
fecI (AL347_23230)	fecI	PA1925 (AL347_02975)	PA1925	+	Homology from 25780925
fecI (AL347_23230)	fecI	PA1924 (AL347_02980)	PA1924	+	Homology from 25780925
fecI (AL347_23230)	fecI	PA1923 (AL347_02985)	PA1923	+	Homology from 25780925
fecI (AL347_23230)	fecI	PA1922 (AL347_02990)	PA1922	+	Homology from 25780925
fecI (AL347_23230)	fecI	PA1342 (AL347_06385)	PA1342 (aatJ)	+	Homology from 25780925
fecI (AL347_23230)	fecI	PA0492 (AL347_20295)	PA0492	+	Homology from 25780925
fecI (AL347_23230)	fecI	PA0387 (AL347_19745)	PA0387	+	Homology from 25780925
fecI (AL347_23230)	fecI	PA0096 (AL347_18250)	PA0096	+	Homology from 25780925
fecI (AL347_23230)	fecI	PA5229 (AL347_15885)	PA5229	+	Homology from 25780925
fecI (AL347_23230)	fecI	phuR (AL347_13120)	phuR	+	Homology from 25780925
fecI (AL347_23230)	fecI	pchR (AL347_21535)	pchR	+	Homology from 25780925
fecI (AL347_23230)	fecI	PA4090 (AL347_22255)	PA4090	+	Homology from 25780925
fecI (AL347_23230)	fecI	hasI (AL347_25675)	hasI	+	Homology from 25780925
fecI (AL347_23230)	fecI	hasAp (AL347_25690)	hasAp	+	Homology from 25780925
fecI (AL347_23230)	fecI	PA3268 (AL347_00160)	PA3268	+	Homology from 25780925
fecI (AL347_23230)	fecI	PA3118 (AL347_27250)	PA3118 (leuB)	+	Homology from 25780925
fecI (AL347_23230)	fecI	pvdS (AL347_32895)	pvdS	?	Homology from 25780925
fecI (AL347_23230)	fecI	PA2380 (AL347_33115)	PA2380	+	Homology from 25780925
fecI (AL347_23230)	fecI	PA1873 (AL347_03255)	PA1873	+	Homology from 25780925
fecI (AL347_23230)	fecI	PA1872 (AL347_03260)	PA1872	+	Homology from 25780925
fecI (AL347_23230)	fecI	PA1631 (AL347_04565)	PA1631	+	Homology from 25780925
fecI (AL347_23230)	fecI	PA1630 (AL347_04570)	PA1630	+	Homology from 25780925
fecI (AL347_23230)	fecI	hxuA (AL347_06600)	hxuA	+	Homology from 25780925
fecI (AL347_23230)	fecI	PA0097 (AL347_18255)	PA0097	+	Homology from 25780925
fecI (AL347_23230)	fecI	PA4987 (AL347_14565)	PA4987	+	Homology from 25780926
fecI (AL347_23230)	fecI	bfiS (AL347_21695)	bfiS	+	Homology from 25780927
fecI (AL347_23230)	fecI	bfiR (AL347_21700)	bfiR	+	Homology from 25780928
fecI (AL347_23230)	fecI	fecR (AL347_23225)	fecR	+	Homology from 25780929

fecI (AL347_23230)	fecI	fecI (AL347_23230)	fecI	?	Homology from 25780930
fhpR (AL347_30085)	fhpR	fhp (AL347_30090)	fhp	+	19767835
fhpR (AL347_30085)	fhpR	fhpR (AL347_30085)	fhpR	-	19767835
fhpR (AL347_30085)	fhpR	ppyR (AL347_30095)	ppyR	+	27242034*, 18974177*, 22587778
fhpR (AL347_30085)	fhpR	PA2662 (AL347_30100)	PA2662	+	27242034*, 18974177*, 22587778
finR (AL347_25740)	finR	fprA (AL347_25745)	fprA	+	28187184
finR (AL347_25740)	finR	finR (AL347_25740)	finR	-	28187184
fis (AL347_13875)	fis	exsB (AL347_04140)	exsB	+	28469612
fis (AL347_13875)	fis	exsA (AL347_04135)	exsA	+	28469612
fleN (AL347_05495)	fleN	fleQ (AL347_07690)	fleQ	-	24167275
fleN (AL347_05495)	fleN	lapA (AL347_10110)	lapA	-	28517238
fleQ (AL347_07690)	fleQ	fliH (AL347_07660)	fliH	+	14617143
fleQ (AL347_07690)	fleQ	pelB (AL347_27530)	pelB	-	18440972
fleQ (AL347_07690)	fleQ	fliD (AL347_07705)	fliD	+	18974177
fleQ (AL347_07690)	fleQ	fleS (AL347_07685)	fleS	+	18974177
fleQ (AL347_07690)	fleQ	fleR (AL347_07680)	fleR	+	18974177
fleQ (AL347_07690)	fleQ	fliE (AL347_07675)	fliE	+	18974177
fleQ (AL347_07690)	fleQ	fliF (AL347_07670)	fliF	+	18974177
fleQ (AL347_07690)	fleQ	fliG (AL347_07665)	fliG	+	18974177
fleQ (AL347_07690)	fleQ	fliI (AL347_07655)	fliI	+	18974177
fleQ (AL347_07690)	fleQ	fliJ (AL347_07650)	fliJ	+	18974177
fleQ (AL347_07690)	fleQ	fliM (AL347_05550)	fliM	+	18974177
fleQ (AL347_07690)	fleQ	fliN (AL347_05545)	fliN	+	18974177
fleQ (AL347_07690)	fleQ	fliO (AL347_05540)	fliO	+	18974177
fleQ (AL347_07690)	fleQ	fliP (AL347_05535)	fliP	+	18974177
fleQ (AL347_07690)	fleQ	fliQ (AL347_05530)	fliQ	+	18974177
fleQ (AL347_07690)	fleQ	fliR (AL347_05525)	fliR	+	18974177
fleQ (AL347_07690)	fleQ	flhB (AL347_05520)	flhB	+	18974177
fleQ (AL347_07690)	fleQ	flhA (AL347_05505)	flhA	+	18974177
fleQ (AL347_07690)	fleQ	fur (AL347_13415)	fur	-	18974177
fleQ (AL347_07690)	fleQ	fliS (AL347_07700)	fliS	+	27636892
fleQ (AL347_07690)	fleQ	lapA (AL347_10110)	lapA	-	28517238
fleQ (AL347_07690)	fleQ	PA0042 (AL347_17975)	PA0042	?	31270321
fleQ (AL347_07690)	fleQ	siaA (AL347_18645)	siaA	?	31270321
fleQ (AL347_07690)	fleQ	PA0643 (AL347_21095)	PA0643	?	31270321
fleQ (AL347_07690)	fleQ	tyrZ (AL347_21220)	tyrZ	?	31270321
fleQ (AL347_07690)	fleQ	PA0718 (AL347_09950)	PA0718	?	31270321
fleQ (AL347_07690)	fleQ	PA0959 (AL347_08410)	PA0959	?	31270321

fleQ (AL347_07690)	fleQ	PA0981 (AL347_08300)	PA0981	?	31270321
fleQ (AL347_07690)	fleQ	cupC3 (AL347_08235)	cupC3	?	31270321
fleQ (AL347_07690)	fleQ	imm2 (AL347_07400)	imm2	?	31270321
fleQ (AL347_07690)	fleQ	aprA (AL347_06875)	aprA	?	31270321
fleQ (AL347_07690)	fleQ	qsrO (AL347_33910)	qsrO	?	31270321
fleQ (AL347_07690)	fleQ	pslA (AL347_00910)	pslA	?	31270321
fleQ (AL347_07690)	fleQ	vgrG3 (AL347_33150)	vgrG3	?	31270321
fleQ (AL347_07690)	fleQ	PA2569 (AL347_31785)	PA2569	?	31270321
fleQ (AL347_07690)	fleQ	nuoA (AL347_30235)	nuoA	?	31270321
fleQ (AL347_07690)	fleQ	PA2793 (AL347_29170)	PA2793	?	31270321
fleQ (AL347_07690)	fleQ	PA2795 (AL347_28925)	PA2795 (dusA, yjbN)	?	31270321
fleQ (AL347_07690)	fleQ	oprI (AL347_28620)	oprI	?	31270321
fleQ (AL347_07690)	fleQ	acpP (AL347_28040)	acpP	?	31270321
fleQ (AL347_07690)	fleQ	rocA2 (AL347_27625)	rocA2	?	31270321
fleQ (AL347_07690)	fleQ	PA3047 (AL347_27615)	PA3047	?	31270321
fleQ (AL347_07690)	fleQ	fimV (AL347_27265)	fimV	?	31270321
fleQ (AL347_07690)	fleQ	wbpJ (AL347_27090)	wbpJ	?	31270321
fleQ (AL347_07690)	fleQ	hisF2 (AL347_27070)	hisF2	?	31270321
fleQ (AL347_07690)	fleQ	wzy (AL347_27055)	wzy	?	31270321
fleQ (AL347_07690)	fleQ	wbpE (AL347_27050)	wbpE	?	31270321
fleQ (AL347_07690)	fleQ	wbpA (AL347_27030)	wbpA	?	31270321
fleQ (AL347_07690)	fleQ	wzz (AL347_27025)	wzz	?	31270321
fleQ (AL347_07690)	fleQ	clpP2 (AL347_26125)	clpP2	?	31270321
fleQ (AL347_07690)	fleQ	PA3489 (AL347_25275)	PA3489	?	31270321
fleQ (AL347_07690)	fleQ	PA3835 (AL347_23570)	PA3835	?	31270321
fleQ (AL347_07690)	fleQ	PA3836 (AL347_23560)	PA3836	?	31270321
fleQ (AL347_07690)	fleQ	PA4080 (AL347_22305)	PA4080	?	31270321
fleQ (AL347_07690)	fleQ	PA4087 (AL347_22270)	PA4087	?	31270321
fleQ (AL347_07690)	fleQ	PA4139 (AL347_21995)	PA4139	?	31270321
fleQ (AL347_07690)	fleQ	phzA1 (AL347_21625)	phzA1	?	31270321
fleQ (AL347_07690)	fleQ	phzB1 (AL347_21620)	phzB1	?	31270321
fleQ (AL347_07690)	fleQ	phzC1 (AL347_21615)	phzC1	?	31270321
fleQ (AL347_07690)	fleQ	phzD1 (AL347_21610)	phzD1	?	31270321
fleQ (AL347_07690)	fleQ	phzE1 (AL347_21605)	phzE1	?	31270321
fleQ (AL347_07690)	fleQ	phzF1 (AL347_21600)	phzF1	?	31270321
fleQ (AL347_07690)	fleQ	rpoC (AL347_21325)	rpoC	?	31270321
fleQ (AL347_07690)	fleQ	PA4523 (AL347_11485)	PA4523	?	31270321
fleQ (AL347_07690)	fleQ	ponA (AL347_14870)	ponA	?	31270321

fleQ (AL347_07690)	fleQ	arcD (AL347_15575)	arcD	?	31270321
fleQ (AL347_07690)	fleQ	PA5446 (AL347_17040)	PA5446	?	31270321
fleQ (AL347_07690)	fleQ	PA5546 (AL347_17550)	PA5546	?	31270321
fleQ (AL347_07690)	fleQ	pelA (AL347_27525)	pelA	d	18440972, 22581773
fleQ (AL347_07690)	fleQ	fliL (AL347_05555)	fliL	+	18440972, 31270321
fleQ (AL347_07690)	fleQ	pslG (AL347_00880)	pslG	?	27242034*
fleQ (AL347_07690)	fleQ	pslF (AL347_008850)	pslF	?	27242034*
fleQ (AL347_07690)	fleQ	pslE (AL347_00890)	pslE	?	27242034*
fleQ (AL347_07690)	fleQ	fleN (AL347_05495)	fleN	?	27242034*
fleQ (AL347_07690)	fleQ	pslD (AL347_00895)	pslD	?	27242034*
fleQ (AL347_07690)	fleQ	fleQ (AL347_07690)	fleQ	?	22587778
fleQ (AL347_07690)	fleQ	PA1442 (AL347_05555)	PA1442	+	22587778
fleQ (AL347_07690)	fleQ	PA1096 (AL347_07695)	PA1096	?	27242034*
fleQ (AL347_07690)	fleQ	pslI (AL347_00870)	pslI	?	27242034*
fleQ (AL347_07690)	fleQ	flhF (AL347_05500)	flhF	?	27242034*
fleQ (AL347_07690)	fleQ	pelE (AL347_27545)	pelE	?	27242034*
fleQ (AL347_07690)	fleQ	pelD (AL347_27540)	pelD	?	27242034*
fleQ (AL347_07690)	fleQ	pelG (AL347_27555)	pelG	?	27242034*
fleQ (AL347_07690)	fleQ	pelF (AL347_27550)	pelF	?	27242034*
fleQ (AL347_07690)	fleQ	pelC (AL347_27535)	pelC	?	27242034*
fleQ (AL347_07690)	fleQ	PA2441 (AL347_32810)	PA2441	?	27242034*
fleQ (AL347_07690)	fleQ	pslK (AL347_00860)	pslK	?	27242034*
fleQ (AL347_07690)	fleQ	pslJ (AL347_00865)	pslJ	?	27242034*
fleQ (AL347_07690)	fleQ	pslH (AL347_00875)	pslH	?	27242034*
fleQ (AL347_07690)	fleQ	pslL (AL347_00855)	pslL	?	27242034*
fleQ (AL347_07690)	fleQ	pslC (AL347_00900)	pslC	?	27242034*
fleQ (AL347_07690)	fleQ	pslB (AL347_00905)	pslB	?	27242034*
flgM (AL347_25995)	flgM	fliC (AL347_07715)	fliC	-	18974177
flgM (AL347_25995)	flgM	fliA (AL347_05490)	fliA	-	18974177
fliA (AL347_05490)	fliA	fliC (AL347_07715)	fliC	+	18974177
fliA (AL347_05490)	fliA	fliL (AL347_05555)	fliL	+	18974177
fliA (AL347_05490)	fliA	flgM (AL347_25995)	flgM	+	18974177
fliA (AL347_05490)	fliA	bifA (AL347_10685)	bifA	+	27175902
fliA (AL347_05490)	fliA	PA5376 (AL347_16670)	PA5376 (cbcV)	+	Homology from 25780925
fliA (AL347_05490)	fliA	PA5285 (AL347_16180)	PA5285 (sutA)	+	Homology from 25780926
fliA (AL347_05490)	fliA	PA5284 (AL347_16170)	PA5284	+	Homology from 25780927
fliA (AL347_05490)	fliA	PA5273 (AL347_16110)	PA5273	+	Homology from 25780928
fliA (AL347_05490)	fliA	PA5272 (AL347_16105)	PA5272 (cyaA)	+	Homology from 25780929

fliA (AL347_05490)	fliA	PA5218 (AL347_15825)	PA5218	+	Homology from 25780930
fliA (AL347_05490)	fliA	PA5217 (AL347_15820)	PA5217	+	Homology from 25780931
fliA (AL347_05490)	fliA	PA5216 (AL347_15815)	PA5216	+	Homology from 25780932
fliA (AL347_05490)	fliA	PA5137 (AL347_15340)	PA5137	+	Homology from 25780933
fliA (AL347_05490)	fliA	PA5072 (AL347_15010)	PA5072 (mcpK)	+	Homology from 25780934
fliA (AL347_05490)	fliA	pilM (AL347_14865)	pilM	+	Homology from 25780935
fliA (AL347_05490)	fliA	pilN (AL347_14860)	pilN	+	Homology from 25780936
fliA (AL347_05490)	fliA	PA5042 (AL347_14855)	PA5042 (pilO)	+	Homology from 25780937
fliA (AL347_05490)	fliA	pilP (AL347_14850)	pilP	+	Homology from 25780938
fliA (AL347_05490)	fliA	PA5040 (AL347_14845)	PA5040 (pilQ)	+	Homology from 25780939
fliA (AL347_05490)	fliA	PA5037 (AL347_14830)	PA5037	+	Homology from 25780940
fliA (AL347_05490)	fliA	PA5018 (AL347_14730)	PA5018 (msrA, pmsR)	+	Homology from 25780941
fliA (AL347_05490)	fliA	PA5017 (AL347_14725)	PA5017 (dipA)	+	Homology from 25780942
fliA (AL347_05490)	fliA	PA4955 (AL347_14395)	PA4955	+	Homology from 25780943
fliA (AL347_05490)	fliA	PA4954 (AL347_14390)	PA4954 (motA)	+	Homology from 25780944
fliA (AL347_05490)	fliA	PA4953 (AL347_14385)	PA4953 (motB, rpmB)	+	Homology from 25780945
fliA (AL347_05490)	fliA	PA4861 (AL347_13915)	PA4861	+	Homology from 25780946
fliA (AL347_05490)	fliA	gcbA (AL347_13825)	gcbA	+	Homology from 25780947
fliA (AL347_05490)	fliA	PA4656 (AL347_12800)	PA4656	+	Homology from 25780948
fliA (AL347_05490)	fliA	hemH (AL347_12795)	hemH	+	Homology from 25780949
fliA (AL347_05490)	fliA	PA4633 (AL347_12675)	PA4633	+	Homology from 25780950
fliA (AL347_05490)	fliA	PA4616 (AL347_12580)	PA4616	+	Homology from 25780951
fliA (AL347_05490)	fliA	glyA3 (AL347_12505)	glyA3	+	Homology from 25780952
fliA (AL347_05490)	fliA	PA4601 (AL347_12500)	PA4601 (morA)	+	Homology from 25780953
fliA (AL347_05490)	fliA	mexD (AL347_12485)	mexD	+	Homology from 25780954
fliA (AL347_05490)	fliA	PA4583 (AL347_12405)	PA4583	+	Homology from 25780955
fliA (AL347_05490)	fliA	PA4520 (AL347_11470)	PA4520	+	Homology from 25780956
fliA (AL347_05490)	fliA	fumC1 (AL347_16940)	fumC1	+	Homology from 25780957
fliA (AL347_05490)	fliA	sodM (AL347_11205)	sodM	+	Homology from 25780958
fliA (AL347_05490)	fliA	PA4467 (AL347_11200)	PA4467	+	Homology from 25780959
fliA (AL347_05490)	fliA	xenB (AL347_10625)	xenB	+	Homology from 25780960
fliA (AL347_05490)	fliA	pyeM (AL347_10620)	pyeM	+	Homology from 25780961
fliA (AL347_05490)	fliA	pyeR (AL347_10615)	pyeR	+	Homology from 25780962
fliA (AL347_05490)	fliA	PA4326 (AL347_10475)	PA4326	+	Homology from 25780963
fliA (AL347_05490)	fliA	PA4325 (AL347_10470)	PA4325	+	Homology from 25780964
fliA (AL347_05490)	fliA	PA4310 (AL347_10385)	PA4310 (pctB)	+	Homology from 25780965
fliA (AL347_05490)	fliA	PA4309 (AL347_10380)	PA4309 (pctA)	+	Homology from 25780966
fliA (AL347_05490)	fliA	pctC (AL347_10370)	pctC	+	Homology from 25780967

fliA (AL347_05490)	fliA	rcpC (AL347_10360)	rcpC	+	Homology from 25780968
fliA (AL347_05490)	fliA	rcpA (AL347_10355)	rcpA	+	Homology from 25780969
fliA (AL347_05490)	fliA	tadA (AL347_10345)	tadA	+	Homology from 25780970
fliA (AL347_05490)	fliA	tadB (AL347_10340)	tadB	+	Homology from 25780971
fliA (AL347_05490)	fliA	tadZ (AL347_10350)	tadZ	+	Homology from 25780972
fliA (AL347_05490)	fliA	tadD (AL347_10330)	tadD	+	Homology from 25780973
fliA (AL347_05490)	fliA	PA4298 (AL347_10325)	PA4298	+	Homology from 25780974
fliA (AL347_05490)	fliA	tadG (AL347_10320)	tadG	+	Homology from 25780975
fliA (AL347_05490)	fliA	pprA (AL347_10300)	pprA	+	Homology from 25780976
fliA (AL347_05490)	fliA	PA4182 (AL347_21770)	PA4182	+	Homology from 25780977
fliA (AL347_05490)	fliA	PA4014 (AL347_22640)	PA4014	+	Homology from 25780978
fliA (AL347_05490)	fliA	PA4013 (AL347_22645)	PA4013	+	Homology from 25780979
fliA (AL347_05490)	fliA	PA4012 (AL347_22650)	PA4012	+	Homology from 25780980
fliA (AL347_05490)	fliA	rocS1 (AL347_22985)	rocS1	+	Homology from 25780981
fliA (AL347_05490)	fliA	PA3936 (AL347_23035)	PA3936	+	Homology from 25780982
fliA (AL347_05490)	fliA	PA3740 (AL347_24070)	PA3740	+	Homology from 25780983
fliA (AL347_05490)	fliA	PA3599 (AL347_24785)	PA3599	+	Homology from 25780984
fliA (AL347_05490)	fliA	PA3592 (AL347_24825)	PA3592	+	Homology from 25780985
fliA (AL347_05490)	fliA	PA3590 (AL347_24835)	PA3590	+	Homology from 25780986
fliA (AL347_05490)	fliA	algA (AL347_25050)	algA	+	Homology from 25780987
fliA (AL347_05490)	fliA	algF (AL347_25055)	algF	+	Homology from 25780988
fliA (AL347_05490)	fliA	algL (AL347_25075)	algL	+	Homology from 25780989
fliA (AL347_05490)	fliA	algX (AL347_25080)	algX	+	Homology from 25780990
fliA (AL347_05490)	fliA	algG (AL347_25085)	algG	+	Homology from 25780991
fliA (AL347_05490)	fliA	algE (AL347_25090)	algE	+	Homology from 25780992
fliA (AL347_05490)	fliA	algK (AL347_25095)	algK	+	Homology from 25780993
fliA (AL347_05490)	fliA	alg44 (AL347_25100)	alg44	+	Homology from 25780994
fliA (AL347_05490)	fliA	alg8 (AL347_25105)	alg8	+	Homology from 25780995
fliA (AL347_05490)	fliA	PA3526 (AL347_25180)	PA3526 (motY)	+	Homology from 25780996
fliA (AL347_05490)	fliA	argG (AL347_25185)	argG	+	Homology from 25780997
fliA (AL347_05490)	fliA	mexQ (AL347_25200)	mexQ	+	Homology from 25780998
fliA (AL347_05490)	fliA	PA3516 (AL347_25230)	PA3516	+	Homology from 25780999
fliA (AL347_05490)	fliA	PA3515 (AL347_25235)	PA3515	+	Homology from 25781000
fliA (AL347_05490)	fliA	PA3443 (ssuC)	PA3443	+	Homology from 25781001
fliA (AL347_05490)	fliA	PA3442 (AL347_25510)	PA3442	+	Homology from 25781002
fliA (AL347_05490)	fliA	PA3429 (AL347_25575)	PA3429	+	Homology from 25781003
fliA (AL347_05490)	fliA	PA3422 (AL347_25610)	PA3422	+	Homology from 25781004
fliA (AL347_05490)	fliA	PA3379 (AL347_25835)	PA3379	+	Homology from 25781005

fliA (AL347_05490)	fliA	PA3377 (AL347_25845)	PA3377	+	Homology from 25781006
fliA (AL347_05490)	fliA	PA3375 (AL347_25855)	PA3375	+	Homology from 25781007
fliA (AL347_05490)	fliA	PA3353 (AL347_25985)	PA3353 (flgZ)	+	Homology from 25781008
fliA (AL347_05490)	fliA	PA3352 (AL347_25990)	PA3352	+	Homology from 25781009
fliA (AL347_05490)	fliA	PA3349 (AL347_26005)	PA3349	+	Homology from 25781010
fliA (AL347_05490)	fliA	plcN (AL347_26160)	plcN	+	Homology from 25781011
fliA (AL347_05490)	fliA	PA3314 (AL347_26185)	PA3314	+	Homology from 25781012
fliA (AL347_05490)	fliA	PA3126 (AL347_27210)	PA3126 (ibpA, hslT)	+	Homology from 25781013
fliA (AL347_05490)	fliA	xcpQ (AL347_27315)	xcpQ	+	Homology from 25781014
fliA (AL347_05490)	fliA	xcpP (AL347_27320)	xcpP	+	Homology from 25781015
fliA (AL347_05490)	fliA	PA3026 (AL347_27725)	PA3026	+	Homology from 25781016
fliA (AL347_05490)	fliA	PA3025 (AL347_27730)	PA3025	+	Homology from 25781017
fliA (AL347_05490)	fliA	PA3024 (AL347_27735)	PA3024	+	Homology from 25781018
fliA (AL347_05490)	fliA	PA2935 (AL347_28205)	PA2935	+	Homology from 25781019
fliA (AL347_05490)	fliA	PA2917 (AL347_28300)	PA2917	+	Homology from 25781020
fliA (AL347_05490)	fliA	PA2916 (AL347_28305)	PA2916	+	Homology from 25781021
fliA (AL347_05490)	fliA	PA2880 (AL347_28485)	PA2880	+	Homology from 25781022
fliA (AL347_05490)	fliA	PA2870 (AL347_28535)	PA2870	+	Homology from 25781023
fliA (AL347_05490)	fliA	PA2869 (AL347_28540)	PA2869	+	Homology from 25781024
fliA (AL347_05490)	fliA	PA2868 (AL347_28545)	PA2868	+	Homology from 25781025
fliA (AL347_05490)	fliA	PA2867 (AL347_28550)	PA2867	+	Homology from 25781026
fliA (AL347_05490)	fliA	PA2856 (AL347_28605)	PA2856 (tesA, apeA)	+	Homology from 25781027
fliA (AL347_05490)	fliA	PA2855 (AL347_28610)	PA2855	+	Homology from 25781028
fliA (AL347_05490)	fliA	PA2788 (AL347_29195)	PA2788	+	Homology from 25781029
fliA (AL347_05490)	fliA	PA2697 (AL347_29925)	PA2697	+	Homology from 25781030
fliA (AL347_05490)	fliA	PA2673 (AL347_30045)	PA2673	+	Homology from 25781031
fliA (AL347_05490)	fliA	PA2672 (AL347_30050)	PA2672	+	Homology from 25781032
fliA (AL347_05490)	fliA	PA2670 (AL347_30060)	PA2670	+	Homology from 25781033
fliA (AL347_05490)	fliA	PA2669 (AL347_30065)	PA2669	+	Homology from 25781034
fliA (AL347_05490)	fliA	PA2654 (AL347_30145)	PA2654 (tlpQ)	+	Homology from 25781035
fliA (AL347_05490)	fliA	PA2652 (AL347_30155)	PA2652	+	Homology from 25781036
fliA (AL347_05490)	fliA	PA2599 (AL347_30435)	PA2599	+	Homology from 25781037
fliA (AL347_05490)	fliA	PA2598 (AL347_30440)	PA2598	+	Homology from 25781038
fliA (AL347_05490)	fliA	PA2594 (AL347_30460)	PA2594	+	Homology from 25781039
fliA (AL347_05490)	fliA	PA2571 (AL347_31770)	PA2571	+	Homology from 25781040
fliA (AL347_05490)	fliA	PA2567 (AL347_31795)	PA2567	+	Homology from 25781041
fliA (AL347_05490)	fliA	PA2561 (AL347_31830)	PA2561 (ctpH)	+	Homology from 25781042
fliA (AL347_05490)	fliA	PA2535 (AL347_31970)	PA2535	+	Homology from 25781043

fliA (AL347_05490)	fliA	czcC (AL347_32050)	czcC	+	Homology from 25781044
fliA (AL347_05490)	fliA	czcB (AL347_32055)	czcB	+	Homology from 25781045
fliA (AL347_05490)	fliA	oprN (AL347_32500)	oprN	+	Homology from 25781046
fliA (AL347_05490)	fliA	mexF (AL347_32505)	mexF	+	Homology from 25781047
fliA (AL347_05490)	fliA	mexE (AL347_32510)	mexE	+	Homology from 25781048
fliA (AL347_05490)	fliA	PA2410 (AL347_32975)	PA2410 (fpvF)	+	Homology from 25781049
fliA (AL347_05490)	fliA	PA2409 (AL347_32980)	PA2409 (fpvE)	+	Homology from 25781050
fliA (AL347_05490)	fliA	PA2408 (AL347_32985)	PA2408 (fpvD)	+	Homology from 25781051
fliA (AL347_05490)	fliA	PA2407 (AL347_32990)	PA2407 (fpvC)	+	Homology from 25781052
fliA (AL347_05490)	fliA	PA2406 (AL347_32995)	PA2406 (fpvK)	+	Homology from 25781053
fliA (AL347_05490)	fliA	PA2405 (AL347_33000)	PA2405 (fpvJ)	+	Homology from 25781054
fliA (AL347_05490)	fliA	PA2404 (AL347_33005)	PA2404 (fpvH)	+	Homology from 25781055
fliA (AL347_05490)	fliA	pvdF (AL347_33035)	pvdF	+	Homology from 25781056
fliA (AL347_05490)	fliA	PA2348 (AL347_33280)	PA2348	+	Homology from 25781057
fliA (AL347_05490)	fliA	PA2310 (AL347_33475)	PA2310	+	Homology from 25781058
fliA (AL347_05490)	fliA	PA2309 (AL347_33480)	PA2309	+	Homology from 25781059
fliA (AL347_05490)	fliA	PA2308 (AL347_33485)	PA2308	+	Homology from 25781060
fliA (AL347_05490)	fliA	PA2307 (AL347_33490)	PA2307	+	Homology from 25781061
fliA (AL347_05490)	fliA	PA2217 (AL347_01465)	PA2217	+	Homology from 25781062
fliA (AL347_05490)	fliA	PA2216 (AL347_01470)	PA2216	+	Homology from 25781063
fliA (AL347_05490)	fliA	PA2215 (AL347_01475)	PA2215 (lyxD)	+	Homology from 25781064
fliA (AL347_05490)	fliA	PA2165 (AL347_01745)	PA2165	+	Homology from 25781065
fliA (AL347_05490)	fliA	PA2164 (AL347_01750)	PA2164	+	Homology from 25781066
fliA (AL347_05490)	fliA	PA2163 (AL347_01755)	PA2163	+	Homology from 25781067
fliA (AL347_05490)	fliA	PA2162 (AL347_01760)	PA2162	+	Homology from 25781068
fliA (AL347_05490)	fliA	PA2161 (AL347_01765)	PA2161	+	Homology from 25781069
fliA (AL347_05490)	fliA	PA2160 (AL347_01770)	PA2160	+	Homology from 25781070
fliA (AL347_05490)	fliA	PA2158 (AL347_01785)	PA2158	+	Homology from 25781071
fliA (AL347_05490)	fliA	PA2153 (AL347_01810)	PA2153 (glgB)	+	Homology from 25781072
fliA (AL347_05490)	fliA	PA2152 (AL347_01815)	PA2152	+	Homology from 25781073
fliA (AL347_05490)	fliA	PA2151 (AL347_01820)	PA2151	+	Homology from 25781074
fliA (AL347_05490)	fliA	PA2131 (AL347_01935)	PA2131 (cupA4)	+	Homology from 25781075
fliA (AL347_05490)	fliA	PA2077 (AL347_02205)	PA2077	+	Homology from 25781076
fliA (AL347_05490)	fliA	PA2036 (AL347_02425)	PA2036	+	Homology from 25781077
fliA (AL347_05490)	fliA	PA2034 (AL347_02435)	PA2034	+	Homology from 25781078
fliA (AL347_05490)	fliA	PA2033 (AL347_02440)	PA2033	+	Homology from 25781079
fliA (AL347_05490)	fliA	PA1913 (AL347_03045)	PA1913	+	Homology from 25781080
fliA (AL347_05490)	fliA	PA1835 (AL347_03465)	PA1835	+	Homology from 25781081

fliA (AL347_05490)	fliA	PA1778 (AL347_03790)	PA1778 (cobA)	+	Homology from 25781082
fliA (AL347_05490)	fliA	PA1740 (AL347_03995)	PA1740	+	Homology from 25781083
fliA (AL347_05490)	fliA	PA1738 (AL347_04005)	PA1738	+	Homology from 25781084
fliA (AL347_05490)	fliA	PA1679 (AL347_04310)	PA1679	+	Homology from 25781085
fliA (AL347_05490)	fliA	PA1646 (AL347_04485)	PA1646	+	Homology from 25781086
fliA (AL347_05490)	fliA	PA1608 (AL347_04685)	PA1608	+	Homology from 25781087
fliA (AL347_05490)	fliA	PA1607 (AL347_04690)	PA1607	+	Homology from 25781088
fliA (AL347_05490)	fliA	PA1569 (AL347_04890)	PA1569	+	Homology from 25781089
fliA (AL347_05490)	fliA	PA1568 (AL347_04895)	PA1568	+	Homology from 25781090
fliA (AL347_05490)	fliA	PA1567 (AL347_04900)	PA1567	+	Homology from 25781091
fliA (AL347_05490)	fliA	aer (AL347_04935)	aer	+	Homology from 25781092
fliA (AL347_05490)	fliA	PA1545 (AL347_05025)	PA1545	+	Homology from 25781093
fliA (AL347_05490)	fliA	PA1538 (AL347_05060)	PA1538	+	Homology from 25781094
fliA (AL347_05490)	fliA	PA1537 (AL347_05065)	PA1537	+	Homology from 25781095
fliA (AL347_05490)	fliA	PA1510 (AL347_05210)	PA1510 (tplE, tle4)	+	Homology from 25781096
fliA (AL347_05490)	fliA	PA1509 (AL347_05215)	PA1509 (tplEi, tli4)	+	Homology from 25781097
fliA (AL347_05490)	fliA	PA1498 (AL347_05270)	PA1498 (pykF, pyk-I)	+	Homology from 25781098
fliA (AL347_05490)	fliA	PA1474 (AL347_05395)	PA1474	+	Homology from 25781099
fliA (AL347_05490)	fliA	PA1473 (AL347_05400)	PA1473	+	Homology from 25781100
fliA (AL347_05490)	fliA	PA1467 (AL347_05430)	PA1467	+	Homology from 25781101
fliA (AL347_05490)	fliA	PA1465 (AL347_05440)	PA1465	+	Homology from 25781102
fliA (AL347_05490)	fliA	PA1462 (AL347_05455)	PA1462	+	Homology from 25781103
fliA (AL347_05490)	fliA	motD (AL347_05460)	motD	+	Homology from 25781104
fliA (AL347_05490)	fliA	PA1460 (AL347_05465)	PA1460 (motC)	+	Homology from 25781105
fliA (AL347_05490)	fliA	PA1459 (AL347_05470)	PA1459	+	Homology from 25781106
fliA (AL347_05490)	fliA	PA1458 (AL347_05475)	PA1458	+	Homology from 25781107
fliA (AL347_05490)	fliA	PA1456 (AL347_05485)	PA1456 (cheY)	+	Homology from 25781108
fliA (AL347_05490)	fliA	fliA (AL347_05490)	fliA	?	Homology from 25781109
fliA (AL347_05490)	fliA	PA1423 (AL347_05700)	PA1423 (bdlA)	+	Homology from 25781110
fliA (AL347_05490)	fliA	gbuA (AL347_05710)	gbuA	+	Homology from 25781111
fliA (AL347_05490)	fliA	PA1420 (AL347_05715)	PA1420	+	Homology from 25781112
fliA (AL347_05490)	fliA	PA1419 (AL347_05720)	PA1419	+	Homology from 25781113
fliA (AL347_05490)	fliA	PA1367 (AL347_06265)	PA1367	+	Homology from 25781114
fliA (AL347_05490)	fliA	PA1354 (AL347_06325)	PA1354	+	Homology from 25781115
fliA (AL347_05490)	fliA	PA1353 (AL347_06330)	PA1353	+	Homology from 25781116
fliA (AL347_05490)	fliA	PA1346 (AL347_06365)	PA1346	+	Homology from 25781117
fliA (AL347_05490)	fliA	cyoC (AL347_06510)	cyoC	+	Homology from 25781118
fliA (AL347_05490)	fliA	PA1311 (AL347_06555)	PA1311 (phnX)	+	Homology from 25781119

fliA (AL347_05490)	fliA	PA1254 (AL347_06850)	PA1254 (lhpC)	+	Homology from 25781120
fliA (AL347_05490)	fliA	PA1253 (AL347_06855)	PA1253 (lhpG)	+	Homology from 25781121
fliA (AL347_05490)	fliA	PA1251 (AL347_06865)	PA1251	+	Homology from 25781122
fliA (AL347_05490)	fliA	PA1244 (AL347_06910)	PA1244 (qslA)	+	Homology from 25781123
fliA (AL347_05490)	fliA	PA1232 (AL347_06970)	PA1232	+	Homology from 25781124
fliA (AL347_05490)	fliA	PA1221 (AL347_07025)	PA1221	+	Homology from 25781125
fliA (AL347_05490)	fliA	PA1220 (AL347_07030)	PA1220	+	Homology from 25781126
fliA (AL347_05490)	fliA	PA1219 (AL347_07035)	PA1219	+	Homology from 25781127
fliA (AL347_05490)	fliA	PA1218 (AL347_07040)	PA1218	+	Homology from 25781128
fliA (AL347_05490)	fliA	PA1217 (AL347_07045)	PA1217	+	Homology from 25781129
fliA (AL347_05490)	fliA	PA1216 (AL347_07050)	PA1216	+	Homology from 25781130
fliA (AL347_05490)	fliA	PA1214 (AL347_07060)	PA1214	+	Homology from 25781131
fliA (AL347_05490)	fliA	PA1213 (AL347_07065)	PA1213	+	Homology from 25781132
fliA (AL347_05490)	fliA	PA1212 (AL347_07070)	PA1212	+	Homology from 25781133
fliA (AL347_05490)	fliA	PA1211 (AL347_07075)	PA1211	+	Homology from 25781134
fliA (AL347_05490)	fliA	PA1181 (AL347_07230)	PA1181	+	Homology from 25781135
fliA (AL347_05490)	fliA	rhlC (AL347_07505)	rhlC	+	Homology from 25781136
fliA (AL347_05490)	fliA	fliJ (AL347_07650)	fliJ	+	Homology from 25781137
fliA (AL347_05490)	fliA	fliI (AL347_07655)	fliI	+	Homology from 25781138
fliA (AL347_05490)	fliA	fliH (AL347_07660)	fliH	+	Homology from 25781139
fliA (AL347_05490)	fliA	fliG (AL347_07665)	fliG	+	Homology from 25781140
fliA (AL347_05490)	fliA	fliF (AL347_07670)	fliF	+	Homology from 25781141
fliA (AL347_05490)	fliA	fliE (AL347_07675)	fliE	+	Homology from 25781142
fliA (AL347_05490)	fliA	PA1096 (AL347_07695)	PA1096	+	Homology from 25781143
fliA (AL347_05490)	fliA	fliS (AL347_07700)	fliS	+	Homology from 25781144
fliA (AL347_05490)	fliA	fliD (AL347_07705)	fliD	+	Homology from 25781145
fliA (AL347_05490)	fliA	PA1093 (AL347_07710)	PA1093	+	Homology from 25781146
fliA (AL347_05490)	fliA	PA1090 (AL347_07725)	PA1090	+	Homology from 25781147
fliA (AL347_05490)	fliA	PA1089 (AL347_07730)	PA1089	+	Homology from 25781148
fliA (AL347_05490)	fliA	PA1088 (AL347_07735)	PA1088	+	Homology from 25781149
fliA (AL347_05490)	fliA	flgL (AL347_07740)	flgL	+	Homology from 25781150
fliA (AL347_05490)	fliA	PA1028 (AL347_08045)	PA1028 (amaA, amaB)	+	Homology from 25781151
fliA (AL347_05490)	fliA	PA1023 (AL347_08075)	PA1023	+	Homology from 25781152
fliA (AL347_05490)	fliA	PA0982 (AL347_08290)	PA0982	+	Homology from 25781153
fliA (AL347_05490)	fliA	PA0980 (AL347_08305)	PA0980	+	Homology from 25781154
fliA (AL347_05490)	fliA	tolB (AL347_08345)	tolB	+	Homology from 25781155
fliA (AL347_05490)	fliA	tolA (AL347_08350)	tolA	+	Homology from 25781156
fliA (AL347_05490)	fliA	PA0967 (AL347_08370)	PA0967 (ruvB)	+	Homology from 25781157

fliA (AL347_05490)	fliA	dctM (AL347_09065)	dctM	+	Homology from 25781158
fliA (AL347_05490)	fliA	dctQ (AL347_09070)	dctQ	+	Homology from 25781159
fliA (AL347_05490)	fliA	PA0884 (AL347_09075)	PA0884	+	Homology from 25781160
fliA (AL347_05490)	fliA	PA0883 (AL347_09080)	PA0883	+	Homology from 25781161
fliA (AL347_05490)	fliA	PA0882 (AL347_09085)	PA0882	+	Homology from 25781162
fliA (AL347_05490)	fliA	PA0818 (AL347_09420)	PA0818	+	Homology from 25781163
fliA (AL347_05490)	fliA	mucB (AL347_09695)	mucB	+	Homology from 25781164
fliA (AL347_05490)	fliA	PA0728 (AL347_09900)	PA0728	+	Homology from 25781165
fliA (AL347_05490)	fliA	PA0727 (AL347_09905)	PA0727	+	Homology from 25781166
fliA (AL347_05490)	fliA	PA0721 (AL347_09935)	PA0721	+	Homology from 25781167
fliA (AL347_05490)	fliA	PA0720 (AL347_09940)	PA0720	+	Homology from 25781168
fliA (AL347_05490)	fliA	PA0701 (AL347_10045)	PA0701	+	Homology from 25781169
fliA (AL347_05490)	fliA	PA0699 (AL347_10055)	PA0699	+	Homology from 25781170
fliA (AL347_05490)	fliA	phdA (AL347_10095)	phdA	+	Homology from 25781171
fliA (AL347_05490)	fliA	PA0690 (AL347_10100)	PA0690 (pdtA)	+	Homology from 25781172
fliA (AL347_05490)	fliA	PA0684 (AL347_10130)	PA0684 (hxcZ)	+	Homology from 25781173
fliA (AL347_05490)	fliA	PA0669 (dnaE2)	PA0669	+	Homology from 25781174
fliA (AL347_05490)	fliA	PA0665 (AL347_21205)	PA0665	+	Homology from 25781175
fliA (AL347_05490)	fliA	PA0452 (AL347_20085)	PA0452	+	Homology from 25781176
fliA (AL347_05490)	fliA	PA0451 (AL347_20080)	PA0451	+	Homology from 25781177
fliA (AL347_05490)	fliA	PA0444 (AL347_20050)	PA0444	+	Homology from 25781178
fliA (AL347_05490)	fliA	PA0259 (AL347_19065)	PA0259 (tla3, tli3)	+	Homology from 25781179
fliA (AL347_05490)	fliA	cttP (AL347_18685)	cttP	+	Homology from 25781180
fliA (AL347_05490)	fliA	PA0179 (AL347_18680)	PA0179	+	Homology from 25781181
fliA (AL347_05490)	fliA	PA0178 (AL347_18675)	PA0178	+	Homology from 25781182
fliA (AL347_05490)	fliA	PA0177 (AL347_18670)	PA0177	+	Homology from 25781183
fliA (AL347_05490)	fliA	PA0175 (AL347_18660)	PA0175 (cheR2)	+	Homology from 25781184
fliA (AL347_05490)	fliA	PA0174 (AL347_18655)	PA0174	+	Homology from 25781185
fliA (AL347_05490)	fliA	PA0173 (AL347_18650)	PA0173	+	Homology from 25781186
fliA (AL347_05490)	fliA	PA0154 (AL347_18550)	PA0154 (pcaG)	+	Homology from 25781187
fliA (AL347_05490)	fliA	PA0153 (AL347_18545)	PA0153 (pcaH)	+	Homology from 25781188
fliA (AL347_05490)	fliA	algU (AL347_09705)	algU	?	Homology from 25781189
fliA (AL347_05490)	fliA	PA1442 (AL347_05555)	PA1442	+	22587778
fliC (AL347_07715)	fliC	exoT (AL347_17990)	exoT	-	16988269
fliC (AL347_07715)	fliC	exoY (AL347_01600)	exoY	-	16988269
fliC (AL347_07715)	fliC	exoS (AL347_23535)	exoS	-	16988269
foxI (AL347_32680)	foxI	foxA (AL347_32690)	foxA	+	25809487
foxR (AL347_32685)	foxR	foxI (AL347_32680)	foxI	-	25809487

fpvI (AL347_33080)	fpvI	PA5106 (AL347_15175)	PA5106	+	Homology from 25780925
fpvI (AL347_33080)	fpvI	PA4287 (AL347_10270)	PA4287	+	Homology from 25780926
fpvI (AL347_33080)	fpvI	hasR (AL347_25685)	hasR	+	Homology from 25780927
fpvI (AL347_33080)	fpvI	PA2406 (AL347_32995)	PA2406 (fpvK)	+	Homology from 25780928
fpvI (AL347_33080)	fpvI	PA2405 (AL347_33000)	PA2405 (fpvJ)	+	Homology from 25780929
fpvI (AL347_33080)	fpvI	PA2404 (AL347_33005)	PA2404 (fpvH)	+	Homology from 25780930
fpvI (AL347_33080)	fpvI	pvdA (AL347_33085)	pvdA	+	Homology from 25780931
fpvI (AL347_33080)	fpvI	PA1254 (AL347_06850)	PA1254 (lhpC)	+	Homology from 25780932
fpvI (AL347_33080)	fpvI	PA1253 (AL347_06855)	PA1253 (lhpG)	+	Homology from 25780933
fpvI (AL347_33080)	fpvI	PA4902 (AL347_14120)	PA4902	+	Homology from 25780934
fpvI (AL347_33080)	fpvI	fpvI (AL347_33080)	fpvI	?	Homology from 25780935
fpvR (AL347_33075)	fpvR	fpvI (AL347_33080)	fpvI	-	18974177
fpvR (AL347_33075)	fpvR	pvdS (AL347_32895)	pvdS	-	18974177
fruR (AL347_24990)	fruR	fruR (AL347_24990)	fruR	-	27242034*
fruR (AL347_24990)	fruR	fruK (AL347_25000)	fruK	-	27242034*
fruR (AL347_24990)	fruR	fruA (AL347_25005)	fruA	-	27242034*
fruR (AL347_24990)	fruR	fruI (AL347_24995)	fruI	-	27242034*
fur (AL347_13415)	fur	pvdQ (AL347_33090)	pvdQ	+	18440972
fur (AL347_13415)	fur	fpvR (AL347_33075)	fpvR	+	18440972
fur (AL347_13415)	fur	foxR (AL347_32685)	foxR	-	18440972
fur (AL347_13415)	fur	fptB (AL347_21570)	fptB	+	18440972
fur (AL347_13415)	fur	fptA (AL347_21565)	fptA	+	18440972
fur (AL347_13415)	fur	pchR (AL347_21535)	pchR	-	18440972
fur (AL347_13415)	fur	rplL (AL347_21315)	rplL	+	18440972
fur (AL347_13415)	fur	rplJ (AL347_21310)	rplJ	+	18440972
fur (AL347_13415)	fur	rplA (AL347_21305)	rplA	+	18440972
fur (AL347_13415)	fur	icmP (AL347_10700)	icmP	+	18440972
fur (AL347_13415)	fur	fagA (AL347_11220)	fagA	-	18440972
fur (AL347_13415)	fur	tolQ (AL347_08360)	tolQ	-	18974177
fur (AL347_13415)	fur	tolR (AL347_08355)	tolR	-	18974177
fur (AL347_13415)	fur	tolA (AL347_08350)	tolA	-	18974177
fur (AL347_13415)	fur	tolB (AL347_08345)	tolB	-	18974177
fur (AL347_13415)	fur	oprL (AL347_08340)	oprL	-	18974177
fur (AL347_13415)	fur	toxA (AL347_07415)	toxA	-	18974177
fur (AL347_13415)	fur	gbuR (AL347_05705)	gbuR	-	18974177
fur (AL347_13415)	fur	hasR (AL347_25685)	hasR	+	18974177
fur (AL347_13415)	fur	pchF (AL347_21545)	pchF	-	18974177
fur (AL347_13415)	fur	pchE (AL347_21540)	pchE	-	18974177

fur (AL347_13415)	fur	pchD (AL347_21530)	pchD	-	18974177
fur (AL347_13415)	fur	pchC (AL347_21525)	pchC	-	18974177
fur (AL347_13415)	fur	pchB (AL347_21520)	pchB	-	18974177
fur (AL347_13415)	fur	pchA (AL347_21515)	pchA	-	18974177
fur (AL347_13415)	fur	phuT (AL347_13110)	phuT	+	18974177
fur (AL347_13415)	fur	pfeR (AL347_29975)	pfeR	-	18974177
fur (AL347_13415)	fur	pirR (AL347_08575)	pirR	-	19130263
fur (AL347_13415)	fur	pirA (AL347_08565)	pirA	-	19130263
fur (AL347_13415)	fur	hxuA (AL347_06600)	hxuA	-	19130263
fur (AL347_13415)	fur	pfuA (AL347_06495)	pfuA	-	19130263
fur (AL347_13415)	fur	PA2033 (AL347_02440)	PA2033	-	19130263
fur (AL347_13415)	fur	PA2034 (AL347_02435)	PA2034	-	19130263
fur (AL347_13415)	fur	optO (AL347_33350)	optO	-	19130263
fur (AL347_13415)	fur	foxA (AL347_32690)	foxA	-	19130263
fur (AL347_13415)	fur	pfeA (AL347_29965)	pfeA	-	19130263
fur (AL347_13415)	fur	PA3268 (AL347_00160)	PA3268	-	19130263
fur (AL347_13415)	fur	fpvB (AL347_21845)	fpvB	-	19130263
fur (AL347_13415)	fur	PA4467 (AL347_11200)	PA4467	-	19130263
fur (AL347_13415)	fur	sodM (AL347_11205)	sodM	-	19130263
fur (AL347_13415)	fur	PA4469 (AL347_11210)	PA4469	-	19130263
fur (AL347_13415)	fur	fumC1 (AL347_16940)	fumC1	-	19130263
fur (AL347_13415)	fur	PA4514 (AL347_11440)	PA4514	-	19130263
fur (AL347_13415)	fur	chtA (AL347_12915)	chtA	-	19130263
fur (AL347_13415)	fur	phuU (AL347_13105)	phuU	d	19130263
fur (AL347_13415)	fur	phuS (AL347_13115)	phuS	d	19130263
fur (AL347_13415)	fur	optI (AL347_14095)	optI	-	19130263
fur (AL347_13415)	fur	PA5216 (AL347_15815)	PA5216	-	19130263
fur (AL347_13415)	fur	PA5217 (AL347_15820)	PA5217	-	19130263
fur (AL347_13415)	fur	hemO (AL347_10190)	hemO	+	21943078, 27242034*
fur (AL347_13415)	fur	PA4516 (AL347_11450)	PA4516	+	21943078
fur (AL347_13415)	fur	PA4895 (AL347_14085)	PA4895	+	21943078
fur (AL347_13415)	fur	bfd (AL347_25160)	bfd	d	24413814
fur (AL347_13415)	fur	PA0149 (AL347_18525)	PA0149	?	29729420
fur (AL347_13415)	fur	fiuI (AL347_20195)	fiuI	?	29729420
fur (AL347_13415)	fur	femI (AL347_03050)	femI	?	29729420
fur (AL347_13415)	fur	PA2050 (AL347_02355)	PA2050	?	29729420
fur (AL347_13415)	fur	hasI (AL347_25675)	hasI	?	29729420, 27242034*
fur (AL347_13415)	fur	hasAp (AL347_25690)	hasAp	+	18440972, 23967252

fur (AL347_13415)	fur	foxI (AL347_32680)	foxI	-	18440972, 29729420
fur (AL347_13415)	fur	pvdS (AL347_32895)	pvdS	-	18974177, 29729420
fur (AL347_13415)	fur	phuR (AL347_13120)	phuR	d	19130263, 18974177
fur (AL347_13415)	fur	fecI (AL347_23230)	fecI	-	19130263,29729420, 29729420
fur (AL347_13415)	fur	PA4896 (AL347_14090)	PA4896	+	21943078, 29729420
fur (AL347_13415)	fur	piuC (AL347_11445)	piuC	-	24413814, 23422914
fur (AL347_13415)	fur	fpvI (AL347_33080)	fpvI	-	28971540, 29729420
fur (AL347_13415)	fur	PA1300 (AL347_06610)	PA1300 (hxuI)	?	29729420, 29729420
fur (AL347_13415)	fur	PA4705 (AL347_13095)	PA4705 (phuW)	+	22587778
fur (AL347_13415)	fur	toxR (AL347_10010)	toxR	-	22587778
fur (AL347_13415)	fur	PA1322 (AL347_06495)	PA1322	?	27242034*
fur (AL347_13415)	fur	aprA (AL347_06875)	aprA	?	27242034*
fur (AL347_13415)	fur	PA4513 (AL347_11435)	PA4513	?	27242034*
fur (AL347_13415)	fur	PA0473 (AL347_20200)	PA0473	?	27242034*
fur (AL347_13415)	fur	PA3842 (AL347_23525)	PA3842 (spcS, orf1)	-	22587778
fur (AL347_13415)	fur	ybgC (AL347_08365)	ybgC	?	22587778
fur (AL347_13415)	fur	PSPA7_RS25870 (AL347_13100	PSPA7_RS25870	-	22587778
fur (AL347_13415)	fur	sodM (AL347_11205)	sodA	-	22587778
fur (AL347_13415)	fur	PA4570 (AL347_12335)	PA4570	+	22587778
fur (AL347_13415)	fur	PA3409 (AL347_25680)	PA3409 (hasS)	+	22587778
fur (AL347_13415)	fur	fecR (AL347_23225)	fecR	-	22587778
fur (AL347_13415)	fur	PA1301 (AL347_06605)	PA1301 (hxuR)	-	22587778
fur (AL347_13415)	fur	motD (AL347_05460)	motD	-	22587778
fur (AL347_13415)	fur	PA0071 (AL347_18125)	PA0071 (tagR1)	-	22587778
fur (AL347_13415)	fur	PA0072 (AL347_18130)	PA0072 (tagS1)	-	22587778
fur (AL347_13415)	fur	PA0929 (AL347_08575)	PA0929	-	22587778
fur (AL347_13415)	fur	PA0930 (AL347_08570)	PA0930	-	22587778
fur (AL347_13415)	fur	hasAp_P	hasAp_P	+	22587778
fur (AL347_13415)	fur	hasAp_N	hasAp_N	-	22587778
gacA (AL347_30505)	gacA	lasR (AL347_05615)	lasR	+	18974177
gacA (AL347_30505)	gacA	hcnA (AL347_01585)	hcnA	+	18974177
gacA (AL347_30505)	gacA	hcnB (AL347_01580)	hcnB	+	18974177
gacA (AL347_30505)	gacA	hcnC (AL347_01575)	hcnC	+	18974177
gacA (AL347_30505)	gacA	rsmA (AL347_08970)	rsmA	+	24187093
gacA (AL347_30505)	gacA	vreI (AL347_10175)	vreI	?	29729420
gacA (AL347_30505)	gacA	PA1300 (AL347_06610)	PA1300 (hxuI)	?	29729420
gacA (AL347_30505)	gacA	PA1363 (AL347_06280)	PA1363	?	29729420
gacA (AL347_30505)	gacA	pvdS (AL347_32895)	pvdS	?	29729420

gacA (AL347_30505)	gacA	foxI (AL347_32680)	foxI	?	29729420
gacA (AL347_30505)	gacA	PA4896 (AL347_14090)	PA4896	?	29729420
gacA (AL347_30505)	gacA	PA0028 (AL347_17890)	PA0028	?	31270321
gacA (AL347_30505)	gacA	PA0102 (AL347_18280)	PA0102	?	31270321
gacA (AL347_30505)	gacA	PA0138 (AL347_18465)	PA0138	?	31270321
gacA (AL347_30505)	gacA	triB (AL347_18570)	triB	?	31270321
gacA (AL347_30505)	gacA	triC (AL347_18575)	triC	?	31270321
gacA (AL347_30505)	gacA	PA0165 (AL347_18610)	PA0165	?	31270321
gacA (AL347_30505)	gacA	siaA (AL347_18645)	siaA	?	31270321
gacA (AL347_30505)	gacA	PA0277 (AL347_19170)	PA0277	?	31270321
gacA (AL347_30505)	gacA	aguR (AL347_19255)	aguR	?	31270321
gacA (AL347_30505)	gacA	PA0344 (AL347_19525)	PA0344	?	31270321
gacA (AL347_30505)	gacA	PA0422 (AL347_19930)	PA0422	?	31270321
gacA (AL347_30505)	gacA	PA0428 (AL347_19960)	PA0428	?	31270321
gacA (AL347_30505)	gacA	PA0540 (AL347_20545)	PA0540	?	31270321
gacA (AL347_30505)	gacA	PA0557 (AL347_20630)	PA0557	?	31270321
gacA (AL347_30505)	gacA	phdA (AL347_10095)	phdA	?	31270321
gacA (AL347_30505)	gacA	PA0703 (AL347_10030)	PA0703	?	31270321
gacA (AL347_30505)	gacA	PA0802 (AL347_09500)	PA0802	?	31270321
gacA (AL347_30505)	gacA	PA0851 (AL347_09255)	PA0851	?	31270321
gacA (AL347_30505)	gacA	PA0874 (AL347_09130)	PA0874	?	31270321
gacA (AL347_30505)	gacA	PA1012 (AL347_08145)	PA1012	?	31270321
gacA (AL347_30505)	gacA	pauA (AL347_08110)	pauA	?	31270321
gacA (AL347_30505)	gacA	shaB (AL347_07905)	shaB	?	31270321
gacA (AL347_30505)	gacA	PA1069 (AL347_07835)	PA1069	?	31270321
gacA (AL347_30505)	gacA	roeA (AL347_07640)	roeA	?	31270321
gacA (AL347_30505)	gacA	PA1197 (AL347_07145)	PA1197	?	31270321
gacA (AL347_30505)	gacA	PA1200 (AL347_07130)	PA1200	?	31270321
gacA (AL347_30505)	gacA	PA1211 (AL347_07075)	PA1211	?	31270321
gacA (AL347_30505)	gacA	aprE (AL347_06895)	aprE	?	31270321
gacA (AL347_30505)	gacA	PA1287 (AL347_06675)	PA1287	?	31270321
gacA (AL347_30505)	gacA	PA1295 (AL347_06635)	PA1295	?	31270321
gacA (AL347_30505)	gacA	cyoA (AL347_06520)	cyoA	?	31270321
gacA (AL347_30505)	gacA	PA1473 (AL347_05400)	PA1473	?	31270321
gacA (AL347_30505)	gacA	ccmG (AL347_05360)	ccmG	?	31270321
gacA (AL347_30505)	gacA	exsB (AL347_04140)	exsB	?	31270321
gacA (AL347_30505)	gacA	liuE (AL347_02555)	liuE	?	31270321
gacA (AL347_30505)	gacA	lpdV (AL347_00815)	lpdV	?	31270321

gacA (AL347_30505)	gacA	PA2301 (AL347_33520)	PA2301	?	31270321
gacA (AL347_30505)	gacA	PA2471 (AL347_32665)	PA2471	?	31270321
gacA (AL347_30505)	gacA	PA2502 (AL347_32465)	PA2502	?	31270321
gacA (AL347_30505)	gacA	PA2504 (AL347_32455)	PA2504	?	31270321
gacA (AL347_30505)	gacA	cdpR (AL347_30490)	cdpR	?	31270321
gacA (AL347_30505)	gacA	greB (AL347_28590)	greB	?	31270321
gacA (AL347_30505)	gacA	PA2864 (AL347_28565)	PA2864	?	31270321
gacA (AL347_30505)	gacA	PA2874 (AL347_28515)	PA2874	?	31270321
gacA (AL347_30505)	gacA	cobH (AL347_28360)	cobH	?	31270321
gacA (AL347_30505)	gacA	pelC (AL347_27535)	pelC	?	31270321
gacA (AL347_30505)	gacA	pelB (AL347_27530)	pelB	?	31270321
gacA (AL347_30505)	gacA	cprR (AL347_27475)	cprR	?	31270321
gacA (AL347_30505)	gacA	xcpT (AL347_27335)	xcpT	?	31270321
gacA (AL347_30505)	gacA	PA3110 (AL347_27290)	PA3110	?	31270321
gacA (AL347_30505)	gacA	PA3214 (AL347_00450)	PA3214	?	31270321
gacA (AL347_30505)	gacA	PA3309 (AL347_26210)	PA3309	?	31270321
gacA (AL347_30505)	gacA	PA3325 (AL347_26130)	PA3325	?	31270321
gacA (AL347_30505)	gacA	PA3328 (AL347_26115)	PA3328	?	31270321
gacA (AL347_30505)	gacA	PA3342 (AL347_26040)	PA3342	?	31270321
gacA (AL347_30505)	gacA	amiR (AL347_25935)	amiR	?	31270321
gacA (AL347_30505)	gacA	amiC (AL347_25930)	amiC	?	31270321
gacA (AL347_30505)	gacA	PA3375 (AL347_25855)	PA3375	?	31270321
gacA (AL347_30505)	gacA	PA3457 (AL347_25430)	PA3457	?	31270321
gacA (AL347_30505)	gacA	rhlR (AL347_25325)	rhlR	?	31270321
gacA (AL347_30505)	gacA	grxD (AL347_25145)	grxD	?	31270321
gacA (AL347_30505)	gacA	PA3572 (AL347_24940)	PA3572	?	31270321
gacA (AL347_30505)	gacA	PA3592 (AL347_24825)	PA3592	?	31270321
gacA (AL347_30505)	gacA	pyrG (AL347_24590)	pyrG	?	31270321
gacA (AL347_30505)	gacA	glnD (AL347_24485)	glnD	?	31270321
gacA (AL347_30505)	gacA	PA3669 (AL347_24430)	PA3669	?	31270321
gacA (AL347_30505)	gacA	PA3749 (AL347_24025)	PA3749	?	31270321
gacA (AL347_30505)	gacA	nagE (AL347_23965)	nagE	?	31270321
gacA (AL347_30505)	gacA	PA3892 (AL347_23270)	PA3892	?	31270321
gacA (AL347_30505)	gacA	eddB (AL347_23180)	eddB	?	31270321
gacA (AL347_30505)	gacA	PA3959 (AL347_22915)	PA3959	?	31270321
gacA (AL347_30505)	gacA	PA3973 (AL347_22840)	PA3973	?	31270321
gacA (AL347_30505)	gacA	ubiX (AL347_22615)	ubiX	?	31270321
gacA (AL347_30505)	gacA	PA4112 (AL347_22135)	PA4112	?	31270321

gacA (AL347_30505)	gacA	PA4171 (AL347_21830)	PA4171	?	31270321
gacA (AL347_30505)	gacA	ppgL (AL347_21660)	ppgL	?	31270321
gacA (AL347_30505)	gacA	pchG (AL347_21550)	pchG	?	31270321
gacA (AL347_30505)	gacA	pchF (AL347_21545)	pchF	?	31270321
gacA (AL347_30505)	gacA	recD (AL347_10250)	recD	?	31270321
gacA (AL347_30505)	gacA	PA4289 (AL347_10280)	PA4289	?	31270321
gacA (AL347_30505)	gacA	PA4291 (AL347_10290)	PA4291	?	31270321
gacA (AL347_30505)	gacA	PA4312 (AL347_10400)	PA4312	?	31270321
gacA (AL347_30505)	gacA	PA4340 (AL347_10545)	PA4340	?	31270321
gacA (AL347_30505)	gacA	PA4475 (AL347_11240)	PA4475	?	31270321
gacA (AL347_30505)	gacA	magE (AL347_11305)	magE	?	31270321
gacA (AL347_30505)	gacA	PA4510 (AL347_11420)	PA4510	?	31270321
gacA (AL347_30505)	gacA	PA4586 (AL347_12420)	PA4586	?	31270321
gacA (AL347_30505)	gacA	PA4611 (AL347_12550)	PA4611	?	31270321
gacA (AL347_30505)	gacA	PA4654 (AL347_12790)	PA4654	?	31270321
gacA (AL347_30505)	gacA	PA4676 (AL347_12920)	PA4676	?	31270321
gacA (AL347_30505)	gacA	PA4685 (AL347_12965)	PA4685	?	31270321
gacA (AL347_30505)	gacA	speD2 (AL347_13460)	speD2	?	31270321
gacA (AL347_30505)	gacA	desB (AL347_14050)	desB	?	31270321
gacA (AL347_30505)	gacA	PA4925 (AL347_14235)	PA4925	?	31270321
gacA (AL347_30505)	gacA	PA4948 (AL347_14360)	PA4948	?	31270321
gacA (AL347_30505)	gacA	aruI (AL347_14510)	aruI	?	31270321
gacA (AL347_30505)	gacA	PA4987 (AL347_14565)	PA4987	?	31270321
gacA (AL347_30505)	gacA	PA4992 (AL347_14590)	PA4992	?	31270321
gacA (AL347_30505)	gacA	PA5136 (AL347_15335)	PA5136	?	31270321
gacA (AL347_30505)	gacA	mutY (AL347_15400)	mutY	?	31270321
gacA (AL347_30505)	gacA	dctP (AL347_09075)	dctP	?	31270321
gacA (AL347_30505)	gacA	PA5210 (AL347_15785)	PA5210	?	31270321
gacA (AL347_30505)	gacA	PA5342 (AL347_16465)	PA5342	?	31270321
gacA (AL347_30505)	gacA	PA5517 (AL347_17405)	PA5517	?	31270321
gacA (AL347_30505)	gacA	tonB1 (AL347_17475)	tonB1	?	31270321
gacA (AL347_30505)	gacA	PA5543 (AL347_17535)	PA5543	?	31270321
gacA (AL347_30505)	gacA	fecI (AL347_23230)	fecI	?	31270321, 29729420
gacA (AL347_30505)	gacA	rsmY (AL347_20474)	rsmY	+	22587778
gacA (AL347_30505)	gacA	rsmZ (AL347_24668)	rsmZ	+	22587778
gacA (AL347_30505)	gacA	lasI (AL347_05605)	lasI	?	22587778
gacS (AL347_08580)	gacS	gacA (AL347_30505)	gacA	+	19243444
gacS (AL347_08580)	gacS	rsmY (AL347_20474)	rsmY	+	27242034*

gacS (AL347_08580)	gacS	rsmZ (AL347_24668)	rsmZ	+	27242034*
gbdR (AL347_16690)	gbdR	sdaB (AL347_16685)	sdaB	+	24097953
gbdR (AL347_16690)	gbdR	dgcA (AL347_16780)	dgcA	+	24097953
gbdR (AL347_16690)	gbdR	dgcB (AL347_16785)	dgcB	+	24097953
gbdR (AL347_16690)	gbdR	gbcA (AL347_16840)	gbcA	+	24097953
gbdR (AL347_16690)	gbdR	gbcB (AL347_16845)	gbcB	+	24097953
gbdR (AL347_16690)	gbdR	caiX (AL347_16730)	caiX	-	24187093
gbdR (AL347_16690)	gbdR	soxB (AL347_16870)	soxB	+	24187093
gbdR (AL347_16690)	gbdR	soxD (AL347_16875)	soxD	+	24187093
gbdR (AL347_16690)	gbdR	soxA (AL347_16880)	soxA	+	24187093
gbdR (AL347_16690)	gbdR	soxG (AL347_16885)	soxG	+	24187093
gbdR (AL347_16690)	gbdR	PA2698 (AL347_29920)	PA2698	?	31270321
gbdR (AL347_16690)	gbdR	PA2795 (AL347_28925)	PA2795 (dusA, yjbN)	?	31270321
gbdR (AL347_16690)	gbdR	oprI (AL347_28620)	oprI	?	31270321
gbdR (AL347_16690)	gbdR	rocsS2 (AL347_27630)	rocsS2	?	31270321
gbdR (AL347_16690)	gbdR	rocA2 (AL347_27625)	rocA2	?	31270321
gbdR (AL347_16690)	gbdR	fimV (AL347_27265)	fimV	?	31270321
gbdR (AL347_16690)	gbdR	wbpG (AL347_27075)	wbpG	?	31270321
gbdR (AL347_16690)	gbdR	hisF2 (AL347_27070)	hisF2	?	31270321
gbdR (AL347_16690)	gbdR	wzy (AL347_27055)	wzy	?	31270321
gbdR (AL347_16690)	gbdR	wbpA (AL347_27030)	wbpA	?	31270321
gbdR (AL347_16690)	gbdR	bfrB (AL347_25155)	bfrB	?	31270321
gbdR (AL347_16690)	gbdR	arnB (AL347_25045)	arnB	?	31270321
gbdR (AL347_16690)	gbdR	PA3835 (AL347_23570)	PA3835	?	31270321
gbdR (AL347_16690)	gbdR	PA3836 (AL347_23560)	PA3836	?	31270321
gbdR (AL347_16690)	gbdR	cupB3 (AL347_22285)	cupB3	?	31270321
gbdR (AL347_16690)	gbdR	PA4087 (AL347_22270)	PA4087	?	31270321
gbdR (AL347_16690)	gbdR	PA4139 (AL347_21995)	PA4139	?	31270321
gbdR (AL347_16690)	gbdR	phzA1 (AL347_21625)	phzA1	?	31270321
gbdR (AL347_16690)	gbdR	phzB1 (AL347_21620)	phzB1	?	31270321
gbdR (AL347_16690)	gbdR	phzC1 (AL347_21615)	phzC1	?	31270321
gbdR (AL347_16690)	gbdR	phzD1 (AL347_21610)	phzD1	?	31270321
gbdR (AL347_16690)	gbdR	phzE1 (AL347_21605)	phzE1	?	31270321
gbdR (AL347_16690)	gbdR	phzF1 (AL347_21600)	phzF1	?	31270321
gbdR (AL347_16690)	gbdR	rpsG (AL347_21335)	rpsG	?	31270321
gbdR (AL347_16690)	gbdR	plcH (AL347_09290)	plcH	d	24187093, 19103776
gbdR (AL347_16690)	gbdR	pchP (AL347_16215)	pchP	+	24187093, 19103776
gbdR (AL347_16690)	gbdR	gbdR (AL347_16690)	gbdR	+	24187093, 31270321

gbdR (AL347_16690)	PSPA7_RS29400	SPA7_RS29485 (AL347_16775	PSPA7_RS29485 (PSPA7_6184)	?	27242034*
gbdR (AL347_16690)	PSPA7_RS29400	soxB (AL347_16870)	soxB	?	27242034*
gbdR (AL347_16690)	PSPA7_RS29400	soxA (AL347_16880)	soxA	?	27242034*
gbdR (AL347_16690)	PSPA7_RS29400	soxG (AL347_16885)	soxG	?	27242034*
gbdR (AL347_16690)	PSPA7_RS29400	dgcA (AL347_16780)	PSPA7_RS29490 (PSPA7_6185)	?	27242034*
gbdR (AL347_16690)	PSPA7_RS29400	gbcA (AL347_16840)	PSPA7_RS29550 (PSPA7_6197)	?	27242034*
gbdR (AL347_16690)	PSPA7_RS29400	SPA7_RS29480 (AL347_16770	PSPA7_RS29480 (PSPA7_6182)	?	27242034*
gbuR (AL347_05705)	gbuR	gbuA (AL347_05710)	gbuA	d	18974177
gbuR (AL347_05705)	gbuR	glpR (AL347_24870)	glpR	+	18974177
gbuR (AL347_05705)	gbuR	glmS (AL347_17565)	glmS	?	27242034*
gbuR (AL347_05705)	gbuR	gpuP (AL347_19220)	gpuP	+	27242034*
gbuR (AL347_05705)	gbuR	gpuA (AL347_19225)	gpuA	d	27242034*
gbuR (AL347_05705)	gbuR	ptxS (AL347_00765)	ptxS	+	27242034*
gcsR (AL347_32765)	gcsR	gcvT2 (AL347_32805)	gcvT2	+	27303730
gcsR (AL347_32765)	gcsR	sdaA (AL347_32800)	sdaA	+	27303730
gcsR (AL347_32765)	gcsR	gcvP2 (AL347_32785)	gcvP2	+	27303730
glcC (AL347_16540)	glcC	glcD (AL347_16535)	glcD	-	27242034*
glcC (AL347_16540)	glcC	glcE (AL347_16530)	glcE	-	27242034*
glcC (AL347_16540)	glcC	glcF (AL347_16525)	glcF	-	27242034*
glcC (AL347_16540)	glcC	PA5352 (AL347_16520)	PA5352	-	27242034*
glcC (AL347_16540)	glcC	glcC (AL347_16540)	glcC	-	27242034*
glmR (AL347_17570)	PSPA7_RS30295	glmS (AL347_17565)	glmS	?	18440972*
glpR (AL347_24870)	glpR	agmR (AL347_02720)	agmR	+	18974177
glpR (AL347_24870)	glpR	himA (AL347_29525)	himA	-	18974177
glpR (AL347_24870)	glpR	glpF (AL347_24885)	glpF	-	18974177
glpR (AL347_24870)	glpR	glpD (AL347_24865)	glpD	-	18974177
glpR (AL347_24870)	glpR	glpT (AL347_15915)	glpT	-	18974177
glpR (AL347_24870)	glpR	pelA (AL347_27525)	pelA	-	27392247
glpR (AL347_24870)	glpR	glpK1 (AL347_24895)	glpK1	?	22587778, 18974177*
gltR (AL347_00565)	gltR	toxA (AL347_07415)	toxA	-	24920832
gltR (AL347_00565)	gltR	oprB (AL347_00600)	oprB	-	29607620
gltR (AL347_00565)	gltR	glk (AL347_00560)	glk	-	29607620
gltR (AL347_00565)	gltR	edd (AL347_00555)	edd	-	29607620
gntR (AL347_33430)	gntR	gntP (AL347_33420)	gntP	-	28752954, 27242034*
gntR (AL347_33430)	gntR	PA2321 (AL347_33430)	PA2321 (gntK, gntV, gnuK)	-	27242034*
gntR (AL347_33430)	gntR	gntR (AL347_33430)	gntR	-	27242034*
gpuR (AL347_19230)	gpuR	gpuP (AL347_19220)	gpuP	+	18974177
gpuR (AL347_19230)	gpuR	gpuA (AL347_19225)	gpuA	+	18974177

gpuR (AL347_19230)	gpuR	gpuR (AL347_19230)	gpuR	+	18974177
hexR (AL347_26900)	hexR	zwf (AL347_26905)	zwf	-	29607620
hfq (AL347_14340)	hfq	PA0149 (AL347_18525)	PA0149	?	29729420
hfq (AL347_14340)	hfq	PA1300 (AL347_06610)	PA1300 (hxuI)	?	29729420
hfq (AL347_14340)	hfq	PA1363 (AL347_06280)	PA1363	?	29729420
hfq (AL347_14340)	hfq	sigX (AL347_03799)	sigX	?	29729420
hfq (AL347_14340)	hfq	femI (AL347_03050)	femI	?	29729420
hfq (AL347_14340)	hfq	foxI (AL347_32680)	foxI	?	29729420
hfq (AL347_14340)	hfq	pvdS (AL347_32895)	pvdS	?	29729420, 29729420
himA (AL347_29525)	himA	oprE (AL347_19240)	oprE	+	18440972
himA (AL347_29525)	himA	fliD (AL347_07705)	fliD	+	18440972
himA (AL347_29525)	himA	fleS (AL347_07685)	fleS	+	18440972
himA (AL347_29525)	himA	fleR (AL347_07680)	fleR	+	18440972
himA (AL347_29525)	himA	alg8 (AL347_25105)	alg8	+	18440972
himA (AL347_29525)	himA	alg44 (AL347_25100)	alg44	+	18440972
himA (AL347_29525)	himA	algK (AL347_25095)	algK	+	18440972
himA (AL347_29525)	himA	algE (AL347_25090)	algE	+	18440972
himA (AL347_29525)	himA	algG (AL347_25085)	algG	+	18440972
himA (AL347_29525)	himA	algX (AL347_25080)	algX	+	18440972
himA (AL347_29525)	himA	algL (AL347_25075)	algL	+	18440972
himA (AL347_29525)	himA	algI (AL347_25065)	algI	+	18440972
himA (AL347_29525)	himA	algJ (AL347_25060)	algJ	+	18440972
himA (AL347_29525)	himA	algF (AL347_25055)	algF	+	18440972
himA (AL347_29525)	himA	algA (AL347_25050)	algA	+	18440972
himA (AL347_29525)	himA	murI (AL347_12830)	murI	+	18440972
himA (AL347_29525)	himA	moeB (AL347_12835)	moeB	+	18440972
himA (AL347_29525)	himA	hemK (AL347_12840)	hemK	+	18440972
himA (AL347_29525)	himA	lasR (AL347_05615)	lasR	+	18974177
himA (AL347_29525)	himA	algD (AL347_25110)	algD	+	18974177
himA (AL347_29525)	himA	narI (AL347_23380)	narI	+	18974177
himA (AL347_29525)	himA	narJ (AL347_23375)	narJ	+	18974177
himA (AL347_29525)	himA	narH (AL347_23370)	narH	+	18974177
himA (AL347_29525)	himA	narG (AL347_23365)	narG	+	18974177
himA (AL347_29525)	himA	nark2 (AL347_23360)	nark2	+	18974177
himA (AL347_29525)	himA	nark1 (AL347_23355)	nark1	+	18974177
himA (AL347_29525)	himA	hemA (AL347_12850)	hemA	+	18974177
himA (AL347_29525)	himA	algB (AL347_17235)	algB	+	18974177
himA (AL347_29525)	himA	gbdR (AL347_16690)	gbdR	+	20398205

himA (AL347_29525)	himA	pchP (AL347_16215)	pchP	+	20869215
himD (AL347_27015)	himD	oprE (AL347_19240)	oprE	+	18440972
himD (AL347_27015)	himD	fliD (AL347_07705)	fliD	+	18440972
himD (AL347_27015)	himD	fleS (AL347_07685)	fleS	+	18440972
himD (AL347_27015)	himD	fleR (AL347_07680)	fleR	+	18440972
himD (AL347_27015)	himD	alg8 (AL347_25105)	alg8	+	18440972
himD (AL347_27015)	himD	alg44 (AL347_25100)	alg44	+	18440972
himD (AL347_27015)	himD	algK (AL347_25095)	algK	+	18440972
himD (AL347_27015)	himD	algE (AL347_25090)	algE	+	18440972
himD (AL347_27015)	himD	algG (AL347_25085)	algG	+	18440972
himD (AL347_27015)	himD	algX (AL347_25080)	algX	+	18440972
himD (AL347_27015)	himD	algL (AL347_25075)	algL	+	18440972
himD (AL347_27015)	himD	algI (AL347_25065)	algI	+	18440972
himD (AL347_27015)	himD	algJ (AL347_25060)	algJ	+	18440972
himD (AL347_27015)	himD	algF (AL347_25055)	algF	+	18440972
himD (AL347_27015)	himD	algA (AL347_25050)	algA	+	18440972
himD (AL347_27015)	himD	murI (AL347_12830)	murI	+	18440972
himD (AL347_27015)	himD	moeB (AL347_12835)	moeB	+	18440972
himD (AL347_27015)	himD	hemK (AL347_12840)	hemK	+	18440972
himD (AL347_27015)	himD	lasR (AL347_05615)	lasR	+	18974177
himD (AL347_27015)	himD	algD (AL347_25110)	algD	+	18974177
himD (AL347_27015)	himD	narI (AL347_23380)	narI	+	18974177
himD (AL347_27015)	himD	narJ (AL347_23375)	narJ	+	18974177
himD (AL347_27015)	himD	narH (AL347_23370)	narH	+	18974177
himD (AL347_27015)	himD	narG (AL347_23365)	narG	+	18974177
himD (AL347_27015)	himD	nark2 (AL347_23360)	nark2	+	18974177
himD (AL347_27015)	himD	nark1 (AL347_23355)	nark1	+	18974177
himD (AL347_27015)	himD	hemA (AL347_12850)	hemA	+	18974177
himD (AL347_27015)	himD	algB (AL347_17235)	algB	+	18974177
himD (AL347_27015)	himD	gbdR (AL347_16690)	gbdR	+	20398205
himD (AL347_27015)	himD	pchP (AL347_16215)	pchP	+	20869215
hmgR (AL347_02560)	hmgR	hmgA (AL347_02565)	hmgA	-	28752954
hptB (AL347_26025)	hptB	PA3346 (AL347_26020)	PA3346 (hsbR)	-	20398205
hptB (AL347_26025)	hptB	hsbA (AL347_26015)	hsbA	+	27792789
hsbA (AL347_26015)	hsbA	hsbD (AL347_26035)	hsbD	+	27792789
hsbA (AL347_26015)	hsbA	flgM (AL347_25995)	flgM	-	27792789
hupB (AL347_03625)	hupB	ptxS (AL347_00765)	ptxS	+	15528665
hutC (AL347_15170)	hutC	hutC (AL347_15170)	hutC	-	27242034*

hutC (AL347_15170)	hutC	PA5104 (AL347_15165)	PA5104	-	27242034*
hutC (AL347_15170)	hutC	PA5106 (AL347_15175)	PA5106	-	27242034*
hutC (AL347_15170)	hutC	hutU (AL347_15145)	hutU	-	27242034*
hutC (AL347_15170)	hutC	PA5099 (AL347_15140)	PA5099	-	27242034*
hutC (AL347_15170)	hutC	hutH (AL347_15135)	hutH	-	27242034*
hutC (AL347_15170)	hutC	PA5097 (AL347_15130)	PA5097	-	27242034*
hutC (AL347_15170)	hutC	PA5096 (AL347_15125)	PA5096	-	27242034*
hutC (AL347_15170)	hutC	PA5095 (AL347_15120)	PA5095	-	27242034*
hutC (AL347_15170)	hutC	PA5094 (AL347_15115)	PA5094	-	27242034*
hutC (AL347_15170)	hutC	PA5093 (AL347_15110)	PA5093	-	27242034*
hutC (AL347_15170)	hutC	hutI (AL347_15105)	hutI	-	27242034*
hutC (AL347_15170)	hutC	hutG (AL347_15100)	hutG	-	27242034*
ihf	ihf	alg44 (AL347_25100)	alg44	+	22587778
ihf	ihf	alg8 (AL347_25105)	alg8	+	22587778
ihf	ihf	algA (AL347_25050)	algA	+	22587778
ihf	ihf	algB (AL347_17235)	algB	+	22587778
ihf	ihf	algD (AL347_25110)	algD	+	22587778
ihf	ihf	algE (AL347_25090)	algE	+	22587778
ihf	ihf	algF (AL347_25055)	algF	+	22587778
ihf	ihf	algG (AL347_25085)	algG	+	22587778
ihf	ihf	algI (AL347_25065)	algI	+	22587778
ihf	ihf	algJ (AL347_25060)	algJ	+	22587778
ihf	ihf	algK (AL347_25095)	algK	+	22587778
ihf	ihf	algL (AL347_25075)	algL	+	22587778
ihf	ihf	algX (AL347_25080)	algX	+	22587778
ihf	ihf	fleR (AL347_07680)	fleR	+	22587778
ihf	ihf	fleS (AL347_07685)	fleS	+	22587778
ihf	ihf	fliD (AL347_07705)	fliD	+	22587778
ihf	ihf	fumC1 (AL347_16940)	fumC1	+	22587778
ihf	ihf	hemA (AL347_12850)	hemA	+	22587778
ihf	ihf	hemK (AL347_12840)	hemK	+	22587778
ihf	ihf	lasR (AL347_05615)	lasR	+	22587778
ihf	ihf	moeB (AL347_12835)	moeB	+	22587778
ihf	ihf	murI (AL347_12830)	murI	+	22587778
ihf	ihf	narG (AL347_23365)	narG	+	22587778
ihf	ihf	narH (AL347_23370)	narH	+	22587778
ihf	ihf	narI (AL347_23380)	narI	+	22587778
ihf	ihf	narJ (AL347_23375)	narJ	+	22587778

ihf	ihf	narK1 (AL347_23355)	narK1	+	22587778
ihf	ihf	narK2 (AL347_23360)	narK2	+	22587778
ihf	ihf	oprE (AL347_19240)	oprE	+	22587778
ihf	ihf	algQ (AL347_16020)	algQ	+	22587778
iscR (AL347_23680)	iscR	fdx2 (AL347_23710)	fdx2	-	24466226
iscR (AL347_23680)	iscR	hscA (AL347_23705)	hscA	-	24466226
iscR (AL347_23680)	iscR	hscB (AL347_23700)	hscB	-	24466226
iscR (AL347_23680)	iscR	iscA (AL347_23695)	iscA	-	24466226
iscR (AL347_23680)	iscR	iscU (AL347_23690)	iscU	-	24466226
iscR (AL347_23680)	iscR	iscS (AL347_23685)	iscS	-	24466226
iscR (AL347_23680)	iscR	fprB (AL347_12575)	fprB	+	26230408
iscR (AL347_23680)	iscR	nfuA (AL347_03395)	nfuA	+	30092083
iscR (AL347_23680)	iscR	iscX (AL347_23715)	iscX	-	31251744
iscR (AL347_23680)	iscR	PA3808 (AL347_23715)	PA3808	-	27242034*
iscR (AL347_23680)	iscR	PA0665 (AL347_21205)	PA0665	-	27242034*
iscR (AL347_23680)	iscR	iscR (AL347_23680)	iscR	-	27242034*
kynR (AL347_02170)	kynR	PA2079 (AL347_02190)	PA2079	+	21965577
kynR (AL347_02170)	kynR	kynU (AL347_02180)	kynU	+	21965577
kynR (AL347_02170)	kynR	kynB (AL347_02175)	kynB	+	21965577
kynR (AL347_02170)	kynR	kynR (AL347_02170)	kynR	+	21965577
kynR (AL347_02170)	kynR	kynA (AL347_31730)	kynA	+	21965577
ladS (AL347_22835)	ladS	gacS (AL347_08580)	gacS	+	19936057
ladS (AL347_22835)	ladS	gacA (AL347_30505)	gacA	+	19936057
ladS (AL347_22835)	ladS	rsaL (AL347_05610)	rsaL	+	24034668
laoR (AL347_19640)	laoR	laoA (AL347_19625)	laoA	-	29678916
laoR (AL347_19640)	laoR	laoB (AL347_19630)	laoB	-	29678916
laoR (AL347_19640)	laoR	laoC (AL347_19635)	laoC	-	29678916
lasI (AL347_05605)	lasI	rsaL (AL347_05610)	rsaL	?	22587778, 27242034*
lasI (AL347_05605)	lasI	xcpP (AL347_27320)	xcpP	?	22587778, 27242034*
lasI (AL347_05605)	lasI	lasB (AL347_24155)	lasB	?	22587778, 27242034*
lasI (AL347_05605)	lasI	lasI (AL347_05605)	lasI	?	22587778, 27242034*
lasI (AL347_05605)	lasI	gacA (AL347_30505)	gacA	?	27242034*
lasI (AL347_05605)	lasI	xcpW (AL347_27350)	xcpW	?	27242034*
lasI (AL347_05605)	lasI	xcpV (AL347_27345)	xcpV	?	27242034*
lasI (AL347_05605)	lasI	xcpT (AL347_27335)	xcpT	?	27242034*
lasI (AL347_05605)	lasI	xcpS (AL347_27330)	xcpS	?	27242034*
lasI (AL347_05605)	lasI	xcpR (AL347_27325)	xcpR	?	27242034*
lasI (AL347_05605)	lasI	xcpQ (AL347_27315)	xcpQ	?	27242034*

lasI (AL347_05605)	lasI	xcpZ (AL347_27365)	xcpZ	?	27242034*
lasI (AL347_05605)	lasI	xcpX (AL347_27355)	xcpX	?	27242034*
lasI (AL347_05605)	lasI	qscR (AL347_03130)	qscR	?	27242034*
lasI (AL347_05605)	lasI	lasR (AL347_05615)	lasR	?	27242034*
lasI (AL347_05605)	lasI	rhlB (AL347_25320)	rhlB	?	27242034*
lasI (AL347_05605)	lasI	rhlA (AL347_25315)	rhlA	?	27242034*
lasI (AL347_05605)	lasI	xcpU (AL347_27340)	xcpU	?	27242034*
lasI (AL347_05605)	lasI	rhlI (AL347_25330)	rhlI	?	27242034*
lasI (AL347_05605)	lasI	lasA (AL347_03265)	lasA	?	27242034*
lasI (AL347_05605)	lasI	rhlR (AL347_25325)	rhlR	?	27242034*
lasI (AL347_05605)	lasI	ampR (AL347_22150)	ampR	?	27242034*
lasI (AL347_05605)	lasI	fagA (AL347_11220)	fagA	?	27242034*
lasI (AL347_05605)	lasI	PA4469 (AL347_11210)	PA4469	?	27242034*
lasI (AL347_05605)	lasI	ptxR (AL347_33740)	ptxR	?	27242034*
lasI (AL347_05605)	lasI	pprB (AL347_10315)	pprB	?	27242034*
lasI (AL347_05605)	lasI	eta	eta	?	27242034*
lasI (AL347_05605)	lasI	vfr (AL347_21140)	vfr	?	27242034*
lasR (AL347_05615)	lasR	rpoS (AL347_24665)	rpoS	+	9150205
lasR (AL347_05615)	lasR	plcB (AL347_17880)	plcB	+	18440972
lasR (AL347_05615)	lasR	nuh (AL347_18495)	nuh	+	18440972
lasR (AL347_05615)	lasR	aprD (AL347_06900)	aprD	+	18440972
lasR (AL347_05615)	lasR	aprF (AL347_06890)	aprF	+	18440972
lasR (AL347_05615)	lasR	xcpQ (AL347_27315)	xcpQ	+	18440972
lasR (AL347_05615)	lasR	rhlG (AL347_25795)	rhlG	+	18440972
lasR (AL347_05615)	lasR	bphO (AL347_22115)	bphO	+	18440972
lasR (AL347_05615)	lasR	bphP (AL347_22110)	bphP	+	18440972
lasR (AL347_05615)	lasR	phzB1 (AL347_21620)	phzB1	+	18440972
lasR (AL347_05615)	lasR	phzC1 (AL347_21615)	phzC1	+	18440972
lasR (AL347_05615)	lasR	phzD1 (AL347_21610)	phzD1	+	18440972
lasR (AL347_05615)	lasR	mexR (AL347_19940)	mexR	+	18974177
lasR (AL347_05615)	lasR	hcnB (AL347_01580)	hcnB	+	18974177
lasR (AL347_05615)	lasR	hcnC (AL347_01575)	hcnC	+	18974177
lasR (AL347_05615)	lasR	acpP (AL347_28040)	acpP	+	18974177
lasR (AL347_05615)	lasR	tpbA (AL347_23315)	tpbA	+	19543378
lasR (AL347_05615)	lasR	hvn (AL347_03040)	hvn	+	19682264
lasR (AL347_05615)	lasR	kynU (AL347_02180)	kynU	+	19682264
lasR (AL347_05615)	lasR	kynB (AL347_02175)	kynB	+	19682264
lasR (AL347_05615)	lasR	rhlA (AL347_25315)	rhlA	+	19682264

lasR (AL347_05615)	lasR	rahU (AL347_18385)	rahU	+	19682264, 22587778, 27242034*
lasR (AL347_05615)	lasR	PA0144 (AL347_18500)	PA0144	+	19682264
lasR (AL347_05615)	lasR	PA0572 (AL347_20700)	PA0572	+	19682264
lasR (AL347_05615)	lasR	PA0805 (AL347_09485)	PA0805	+	19682264
lasR (AL347_05615)	lasR	hsiB2 (AL347_04425)	hsiB2	+	19682264, 22587778, 27242034*
lasR (AL347_05615)	lasR	hsiF2 (AL347_04415)	hsiF2	+	19682264, 22587778, 27242034*
lasR (AL347_05615)	lasR	acp1 (AL347_03275)	acp1	+	19682264
lasR (AL347_05615)	lasR	ambE (AL347_33515)	ambE	+	19682264
lasR (AL347_05615)	lasR	ambD (AL347_33510)	ambD	+	19682264
lasR (AL347_05615)	lasR	ambC (AL347_33505)	ambC	+	19682264
lasR (AL347_05615)	lasR	ambB (AL347_33500)	ambB	+	19682264
lasR (AL347_05615)	lasR	PA3904 (AL347_23205)	PA3904	+	19682264
lasR (AL347_05615)	lasR	tecT (AL347_23200)	tecT	+	19682264, 22587778, 27242034*
lasR (AL347_05615)	lasR	PA3906 (AL347_23195)	PA3906	+	19682264
lasR (AL347_05615)	lasR	tseT (AL347_23190)	tseT	+	19682264, 22587778, 27242034*
lasR (AL347_05615)	lasR	tsiT (AL347_23185)	tsiT	+	19682264, 22587778, 27242034*
lasR (AL347_05615)	lasR	cueR (AL347_13485)	cueR	+	19682264
lasR (AL347_05615)	lasR	PA5230 (AL347_15890)	PA5230	+	19682264
lasR (AL347_05615)	lasR	aprX (AL347_06905)	aprX	+	24413814
lasR (AL347_05615)	lasR	aprI (AL347_06870)	aprI	+	24413814
lasR (AL347_05615)	lasR	clpP (AL347_03640)	clpP	+	24413814
lasR (AL347_05615)	lasR	lpdV (AL347_00815)	lpdV	+	24413814
lasR (AL347_05615)	lasR	vqsR (AL347_30475)	vqsR	+	24413814, 22587778, 27242034*
lasR (AL347_05615)	lasR	rhlI (AL347_25330)	rhlI	+	24413814
lasR (AL347_05615)	lasR	aprA (AL347_06875)	aprA	+	28466892
lasR (AL347_05615)	lasR	lasI (AL347_05605)	lasI	+	28466892
lasR (AL347_05615)	lasR	hcnA (AL347_01585)	hcnA	+	28466892
lasR (AL347_05615)	lasR	phzM (AL347_21635)	phzM	+	28466892
lasR (AL347_05615)	lasR	phzA1 (AL347_21625)	phzA1	+	28466892
lasR (AL347_05615)	lasR	rsaL (AL347_05610)	rsaL	+	30083519
lasR (AL347_05615)	lasR	amrZ (AL347_25805)	amrZ	?	31270321
lasR (AL347_05615)	lasR	hsiC2 (AL347_04420)	hsiC2	+	32715566, 22587778, 27242034*
lasR (AL347_05615)	lasR	phzG2 (AL347_03095)	phzG2	+	32715566
lasR (AL347_05615)	lasR	pvdS (AL347_32895)	pvdS	+	18440972, 29729420
lasR (AL347_05615)	lasR	pqsA (AL347_08225)	pqsA	+	18440972, 31270321
lasR (AL347_05615)	lasR	pqsB (AL347_08220)	pqsB	+	18440972, 31270321
lasR (AL347_05615)	lasR	pqsC (AL347_08215)	pqsC	+	18440972, 31270321
lasR (AL347_05615)	lasR	pqsD (AL347_08210)	pqsD	+	18440972, 31270321

lasR (AL347_05615)	lasR	pqsE (AL347_08205)	pqsE	+	18440972, 31270321
lasR (AL347_05615)	lasR	aprE (AL347_06895)	aprE	+	18440972, 31270321
lasR (AL347_05615)	lasR	pslA (AL347_00910)	pslA	+	18440972, 31270321
lasR (AL347_05615)	lasR	xcpP (AL347_27320)	xcpP	+	18440972, 31270321
lasR (AL347_05615)	lasR	phnC (AL347_25810)	phnC	+	18440972, 31270321
lasR (AL347_05615)	lasR	flp (AL347_10365)	flp	+	18440972, 31270321
lasR (AL347_05615)	lasR	mvfR (AL347_08190)	mvfR	+	18974177, 30083519, 31270321
lasR (AL347_05615)	lasR	rhlB (AL347_25320)	rhlB	+	19682264, 24413814
lasR (AL347_05615)	lasR	PA1159 (AL347_07340)	PA1159	+	19682264, 31270321
lasR (AL347_05615)	lasR	hsiA2 (AL347_04430)	hsiA2	+	19682264, 31270321,, 27242034*
lasR (AL347_05615)	lasR	PA2939 (AL347_28180)	PA2939	+	19682264, 31270321
lasR (AL347_05615)	lasR	clpP2 (AL347_26125)	clpP2	+	19682264, 31270321
lasR (AL347_05615)	lasR	PA3535 (AL347_25135)	PA3535	+	19682264, 31270321
lasR (AL347_05615)	lasR	PA4677 (AL347_12925)	PA4677	+	19682264, 31270321
lasR (AL347_05615)	lasR	PA5184 (AL347_15650)	PA5184	+	19682264, 31270321
lasR (AL347_05615)	lasR	PA5232 (AL347_15900)	PA5232	+	19682264, 31270321
lasR (AL347_05615)	lasR	cdpR (AL347_30490)	cdpR	+	24413814, 31270321
lasR (AL347_05615)	lasR	pqsH (AL347_30495)	pqsH	+	28454561, 31270321
lasR (AL347_05615)	lasR	lasB (AL347_24155)	lasB	+	28466892, 31270321
lasR (AL347_05615)	lasR	rhlR (AL347_25325)	rhlR	+	30083519, 31270321
lasR (AL347_05615)	lasR	PA0028 (AL347_17890)	PA0028	+	31270321, 19682264
lasR (AL347_05615)	lasR	PA1869 (AL347_03275)	PA1869	+	22587778, 27242034*
lasR (AL347_05615)	lasR	kinB (AL347_17240)	kinB	+	22587778, 27242034*
lasR (AL347_05615)	lasR	PA0027 (AL347_17885)	PA0027	+	22587778, 27242034*
lasR (AL347_05615)	lasR	PA0855 (AL347_09235)	PA0855	+	22587778, 27242034*
lasR (AL347_05615)	lasR	PA1419 (AL347_05720)	PA1419	+	22587778, 27242034*
lasR (AL347_05615)	lasR	PA2588 (AL347_30490)	PA2588	+	22587778, 27242034*
lasR (AL347_05615)	lasR	PA5181 (AL347_15630)	PA5181	+	22587778, 27242034*
lasR (AL347_05615)	lasR	PA5231 (AL347_15895)	PA5231	+	22587778, 27242034*
lasR (AL347_05615)	lasR	phzE1 (AL347_21605)	phzE1	+	22587778, 27242034*
lasR (AL347_05615)	lasR	phzF1 (AL347_21600)	phzF1	+	22587778, 27242034*
lasR (AL347_05615)	lasR	phzG1 (AL347_21595)	phzG1	+	22587778, 27242034*
lasR (AL347_05615)	lasR	pslB (AL347_00905)	pslB	+	22587778, 27242034*
lasR (AL347_05615)	lasR	pslC (AL347_00900)	pslC	+	22587778, 27242034*
lasR (AL347_05615)	lasR	pslD (AL347_00895)	pslD	+	22587778, 27242034*
lasR (AL347_05615)	lasR	pslE (AL347_00890)	pslE	+	22587778, 27242034*
lasR (AL347_05615)	lasR	pslF (AL347_008850)	pslF	+	22587778, 27242034*
lasR (AL347_05615)	lasR	pslG (AL347_00880)	pslG	+	22587778, 27242034*

lasR (AL347_05615)	lasR	pslH (AL347_00875)	pslH	+	22587778, 27242034*
lasR (AL347_05615)	lasR	pslI (AL347_00870)	pslI	+	22587778, 27242034*
lasR (AL347_05615)	lasR	pslJ (AL347_00865)	pslJ	+	22587778, 27242034*
lasR (AL347_05615)	lasR	pslK (AL347_00860)	pslK	+	22587778, 27242034*
lasR (AL347_05615)	lasR	pslL (AL347_00855)	pslL	+	22587778, 27242034*
lasR (AL347_05615)	lasR	PA1897 (AL347_03135)	PA1897	+	22587778, 27242034*
lasR (AL347_05615)	lasR	phzF2 (AL347_03100)	phzF2	+	27242034*
lasR (AL347_05615)	lasR	PA2592 (AL347_30470)	PA2592	?	27242034*
lasR (AL347_05615)	lasR	qteE (AL347_30465)	qteE	?	27242034*
lexA (AL347_27825)	lexA	PA0069 (AL347_18115)	PA0069	-	27242034*
lexA (AL347_27825)	lexA	PA0922 (AL347_08615)	PA0922	-	27242034*
lexA (AL347_27825)	lexA	PA0671 (AL347_10196)	PA0671	-	27242034*
lexA (AL347_27825)	lexA	PA0670 (AL347_10200)	PA0670	-	27242034*
lexA (AL347_27825)	lexA	PA0669 (AL347_10205)	PA0669	-	27242034*
lexA (AL347_27825)	lexA	lexA (AL347_27825)	lexA	-	27242034*
lexA (AL347_27825)	lexA	PA3008 (AL347_27820)	PA3008 (sulA)	-	27242034*
lexA (AL347_27825)	lexA	recN (AL347_13410)	recN	-	27242034*
lexA (AL347_27825)	lexA	recA (AL347_24695)	recA	-	27242034*
lexA (AL347_27825)	lexA	PA3616 (AL347_24700)	PA3616	-	27242034*
lexA (AL347_27825)	lexA	PA1045 (AL347_07960)	PA1045	-	27242034*
lexA (AL347_27825)	lexA	PA1865 (AL347_03300)	PA1865	-	27242034*
lexA (AL347_27825)	lexA	PA1866 (AL347_03295)	PA1866	-	27242034*
lexA (AL347_27825)	lexA	PA2288 (AL347_00620)	PA2288	-	27242034*
lexA (AL347_27825)	lexA	PA3413 (AL347_25660)	PA3413	-	27242034*
lhpR (AL347_06815)	lhpR	lhpO (AL347_06840)	lhpO	+	27145750
lhpR (AL347_06815)	lhpR	lhpN (AL347_06835)	lhpN	+	27145750
lhpR (AL347_06815)	lhpR	lhpM (AL347_06830)	lhpM	+	27145750
lhpR (AL347_06815)	lhpR	lhpP (AL347_06820)	lhpP	+	27145750
lhpR (AL347_06815)	lhpR	lhpE (AL347_06785)	lhpE	+	27145750
lhpR (AL347_06815)	lhpR	lhpB (AL347_06775)	lhpB	+	27145750
lhpR (AL347_06815)	lhpR	lhpA (AL347_06770)	lhpA	+	27145750
liuR (AL347_02530)	liuR	liuE (AL347_02555)	liuE	-	29787835
liuR (AL347_02530)	liuR	liuD (AL347_02550)	liuD	-	29787835
liuR (AL347_02530)	liuR	liuC (AL347_02545)	liuC	-	29787835
liuR (AL347_02530)	liuR	liuB (AL347_02540)	liuB	-	29787835
liuR (AL347_02530)	liuR	liuA (AL347_02535)	liuA	-	29787835
liuR (AL347_02530)	liuR	liuR (AL347_02530)	liuR	-	29787835
lon (AL347_03630)	lon	hfq (AL347_14340)	hfq	-	27229357

mdrR1 (AL347_23235)	mdrR1	mexB (AL347_19950)	mexB	-	30297364
mdrR1 (AL347_23235)	mdrR1	oprM (AL347_19955)	oprM	-	30297364
mdrR1 (AL347_23235)	mdrR1	emrA (AL347_15515)	emrA	+	30297364
mdrR1 (AL347_23235)	mdrR1	emrB	emrB	+	30297364
metR (AL347_24850)	metR	PA0633 (AL347_21045)	PA0633	-	19592586
metR (AL347_24850)	metR	PA0848 (AL347_09270)	PA0848 (ahpB)	-	19592586
metR (AL347_24850)	metR	metH (AL347_03415)	metH	+	19592586
metR (AL347_24850)	metR	pvdH (AL347_32960)	pvdH	+	19592586
metR (AL347_24850)	metR	atuF (AL347_28430)	atuF	+	19592586
metR (AL347_24850)	metR	PA4144 (AL347_21970)	PA4144	+	19592586
metR (AL347_24850)	metR	PA4223 (AL347_21555)	PA4223	+	19592586
metR (AL347_24850)	metR	ntrC (AL347_15275)	ntrC	-	19592586
metR (AL347_24850)	metR	pvdL (AL347_32905)	pvdL	+	27242034*, 18974177*, 22587778
metR (AL347_24850)	metR	metR (AL347_24850)	metR	-	27242034*, 18974177*, 22587778
metR (AL347_24850)	metR	metE (AL347_02965)	metE	-	27242034*, 18974177*, 22587778
mexR (AL347_19940)	mexR	mexB (AL347_19950)	mexB	-	18974177
mexR (AL347_19940)	mexR	oprM (AL347_19955)	oprM	-	18974177
mexR (AL347_19940)	mexR	mexT (AL347_32559)	mexT	-	18974177
mexR (AL347_19940)	mexR	nalC (AL347_24170)	nalC	-	18974177
mexR (AL347_19940)	mexR	mexA (AL347_19946)	mexA	-	27242034*, 18974177*, 22587778
mexT (AL347_32559)	mexT	mexT (AL347_32559)	mexT	+	18974177
mexT (AL347_32559)	mexT	rhlI (AL347_25330)	rhlI	+	18974177
mexT (AL347_32559)	mexT	exsA (AL347_04135)	exsA	-	19683048
mexT (AL347_32559)	mexT	rhlA (AL347_25315)	rhlA	-	19683048
mexT (AL347_32559)	mexT	lasB (AL347_24155)	lasB	-	19683048
mexT (AL347_32559)	mexT	cbpD (AL347_09250)	cbpD	-	19846594
mexT (AL347_32559)	mexT	pqsA (AL347_08225)	pqsA	-	19846594
mexT (AL347_32559)	mexT	pqsB (AL347_08220)	pqsB	-	19846594
mexT (AL347_32559)	mexT	pqsC (AL347_08215)	pqsC	-	19846594
mexT (AL347_32559)	mexT	pqsD (AL347_08210)	pqsD	-	19846594
mexT (AL347_32559)	mexT	pqsE (AL347_08205)	pqsE	-	19846594
mexT (AL347_32559)	mexT	phnA (AL347_08200)	phnA	-	19846594
mexT (AL347_32559)	mexT	phnB (AL347_08195)	phnB	-	19846594
mexT (AL347_32559)	mexT	hsiB2 (AL347_04425)	hsiB2	-	19846594, 27242034*, 22587778
mexT (AL347_32559)	mexT	hsiC2 (AL347_04420)	hsiC2	-	19846594
mexT (AL347_32559)	mexT	pscE (AL347_04110)	pscE	-	19846594
mexT (AL347_32559)	mexT	PA1744 (AL347_03975)	PA1744	+	19846594
mexT (AL347_32559)	mexT	acp1 (AL347_03275)	acp1	-	19846594

mexT (AL347_32559)	mexT	PA1970 (AL347_02760)	PA1970	+	19846594
mexT (AL347_32559)	mexT	hcnA (AL347_01585)	hcnA	-	19846594
mexT (AL347_32559)	mexT	hcnB (AL347_01580)	hcnB	-	19846594
mexT (AL347_32559)	mexT	hcnC (AL347_01575)	hcnC	-	19846594
mexT (AL347_32559)	mexT	pvdA (AL347_33085)	pvdA	+	19846594
mexT (AL347_32559)	mexT	ptrC (AL347_32590)	ptrC	+	19846594, 27242034*, 22587778
mexT (AL347_32559)	mexT	PA2759 (AL347_29375)	PA2759	+	19846594
mexT (AL347_32559)	mexT	PA2811 (AL347_28845)	PA2811	+	19846594
mexT (AL347_32559)	mexT	PA2812 (AL347_28840)	PA2812	+	19846594
mexT (AL347_32559)	mexT	PA2813 (AL347_28835)	PA2813	+	19846594
mexT (AL347_32559)	mexT	PA3205 (AL347_00495)	PA3205	+	19846594
mexT (AL347_32559)	mexT	PA3229 (AL347_00375)	PA3229	+	19846594
mexT (AL347_32559)	mexT	clpP2 (AL347_26125)	clpP2	-	19846594, 27242034*, 22587778
mexT (AL347_32559)	mexT	PA3331 (AL347_26100)	PA3331	-	19846594
mexT (AL347_32559)	mexT	PA3332 (AL347_26095)	PA3332	-	19846594
mexT (AL347_32559)	mexT	fabH2 (AL347_26090)	fabH2	-	19846594
mexT (AL347_32559)	mexT	PA4141 (AL347_21985)	PA4141	+	19846594
mexT (AL347_32559)	mexT	pyeR (AL347_10615)	pyeR	+	19846594
mexT (AL347_32559)	mexT	pyeM (AL347_10620)	pyeM	+	[illegible]6594, 27242034*, 18974177*, 22587778
mexT (AL347_32559)	mexT	xenB (AL347_10625)	xenB	+	19846594
mexT (AL347_32559)	mexT	PA4623 (AL347_12625)	PA4623	+	19846594
mexT (AL347_32559)	mexT	lldP (AL347_13445)	lldP	-	19846594
mexT (AL347_32559)	mexT	lldD (AL347_13450)	lldD	-	19846594
mexT (AL347_32559)	mexT	PA4772 (AL347_13455)	PA4772	-	19846594
mexT (AL347_32559)	mexT	PA4881 (AL347_14015)	PA4881	+	19846594
mexT (AL347_32559)	mexT	bfd (AL347_25160)	bfd	+	24413814
mexT (AL347_32559)	mexT	mexE (AL347_32510)	mexE	+	28507116
mexT (AL347_32559)	mexT	mexF (AL347_32505)	mexF	+	28507116
mexT (AL347_32559)	mexT	oprN (AL347_32500)	oprN	+	28507116
mexT (AL347_32559)	mexT	PA1942 (AL347_02905)	PA1942	+	31358608
mexT (AL347_32559)	mexT	mexS (AL347_32565)	mexS	+	31358608
mexT (AL347_32559)	mexT	PA1333 (AL347_06435)	PA1333	+	31270321, 31358608
mexT (AL347_32559)	mexT	PA1869 (AL347_03275)	PA1869	-	27242034*, 18974177*, 22587778
mexT (AL347_32559)	mexT	PA4354 (AL347_10615)	PA4354	+	27242034*, 18974177*, 22587778
mexT (AL347_32559)	mexT	qrh	qrh	+	27242034*, 18974177*, 22587778
mgtE (AL347_08915)	mgtE	exsA (AL347_04135)	exsA	-	20028803
mgtE (AL347_08915)	mgtE	gacS (AL347_08580)	gacS	+	28847924
mifR (AL347_17375)	mifR	PA5530 (AL347_17470)	PA5530	+	26114434

mmnR (AL347_05575)	mmnR	mmnS (AL347_05580)	mmnS	-	30420483
mmnR (AL347_05575)	mmnR	mmnR (AL347_05575)	mmnR	-	30420483
mmsR (AL347_24945)	mmsR	mmsB (AL347_24955)	mmsB	?	27242034*
mmsR (AL347_24945)	mmsR	mmsA (AL347_24950)	mmsA	?	27242034*
modE (AL347_20270)	modE	modA (AL347_03310)	modA	-	27242034*
modE (AL347_20270)	modE	modB (AL347_03315)	modB	-	27242034*
modE (AL347_20270)	modE	modC (AL347_03320)	modC	-	27242034*
mucA (AL347_09700)	mucA	algU (AL347_09705)	algU	-	27270273
mucB (AL347_09695)	mucB	algU (AL347_09705)	algU	-	18974177
mucC (AL347_09690)	mucC	algU (AL347_09705)	algU	-	18974177
mucD (AL347_09685)	mucD	algU (AL347_09705)	algU	-	18974177
mvaT (AL347_10420)	mvaT	ptxS (AL347_00765)	ptxS	+	15528665
mvaT (AL347_10420)	mvaT	mexE (AL347_32510)	mexE	-	16448502
mvaT (AL347_10420)	mvaT	mexF (AL347_32505)	mexF	-	16448502
mvaT (AL347_10420)	mvaT	oprN (AL347_32500)	oprN	-	16448502
mvaT (AL347_10420)	mvaT	aotJ (AL347_09055)	aotJ	-	19684136
mvaT (AL347_10420)	mvaT	cupA1 (AL347_01950)	cupA1	-	19684136
mvaT (AL347_10420)	mvaT	cupB1 (AL347_22275)	cupB1	-	19832907
mvaT (AL347_10420)	mvaT	sigX (AL347_03799)	sigX	?	29729420
mvaT (AL347_10420)	mvaT	cupC1 (AL347_08245)	cupC1	-	27242034*, 18974177*, 22587778
mvaU (AL347_30075)	mvaU	aotJ (AL347_09055)	aotJ	-	19684136
mvaU (AL347_30075)	mvaU	cupA1 (AL347_01950)	cupA1	-	19684136
mvfR (AL347_08190)	mvfR	phnA (AL347_08200)	phnA	+	18974177
mvfR (AL347_08190)	mvfR	phnB (AL347_08195)	phnB	+	18974177
mvfR (AL347_08190)	mvfR	rhlI (AL347_25330)	rhlI	+	18974177
mvfR (AL347_08190)	mvfR	algU (AL347_09705)	algU	?	29729420
mvfR (AL347_08190)	mvfR	sigX (AL347_03799)	sigX	?	29729420
mvfR (AL347_08190)	mvfR	femI (AL347_03050)	femI	?	29729420
mvfR (AL347_08190)	mvfR	PA0138 (AL347_18465)	PA0138	?	31270321
mvfR (AL347_08190)	mvfR	triB (AL347_18570)	triB	?	31270321
mvfR (AL347_08190)	mvfR	aguR (AL347_19255)	aguR	?	31270321
mvfR (AL347_08190)	mvfR	PA0422 (AL347_19930)	PA0422	?	31270321
mvfR (AL347_08190)	mvfR	PA0540 (AL347_20545)	PA0540	?	31270321
mvfR (AL347_08190)	mvfR	PA0557 (AL347_20630)	PA0557	?	31270321
mvfR (AL347_08190)	mvfR	PA0570 (AL347_20690)	PA0570	?	31270321
mvfR (AL347_08190)	mvfR	PA0643 (AL347_21095)	PA0643	?	31270321
mvfR (AL347_08190)	mvfR	PA0802 (AL347_09500)	PA0802	?	31270321
mvfR (AL347_08190)	mvfR	PA0851 (AL347_09255)	PA0851	?	31270321

mvfR (AL347_08190)	mvfR	PA0874 (AL347_09130)	PA0874	?	31270321
mvfR (AL347_08190)	mvfR	PA0883 (AL347_09080)	PA0883	?	31270321
mvfR (AL347_08190)	mvfR	lysC (AL347_08975)	lysC	?	31270321
mvfR (AL347_08190)	mvfR	PA1200 (AL347_07130)	PA1200	?	31270321
mvfR (AL347_08190)	mvfR	PA1295 (AL347_06635)	PA1295	?	31270321
mvfR (AL347_08190)	mvfR	PA2004 (AL347_02590)	PA2004	?	31270321
mvfR (AL347_08190)	mvfR	lpdV (AL347_00815)	lpdV	?	31270321
mvfR (AL347_08190)	mvfR	PA2864 (AL347_28565)	PA2864	?	31270321
mvfR (AL347_08190)	mvfR	PA2874 (AL347_28515)	PA2874	?	31270321
mvfR (AL347_08190)	mvfR	pelC (AL347_27535)	pelC	?	31270321
mvfR (AL347_08190)	mvfR	pelB (AL347_27530)	pelB	?	31270321
mvfR (AL347_08190)	mvfR	cprR (AL347_27475)	cprR	?	31270321
mvfR (AL347_08190)	mvfR	xcpT (AL347_27335)	xcpT	?	31270321
mvfR (AL347_08190)	mvfR	PA3214 (AL347_00450)	PA3214	?	31270321
mvfR (AL347_08190)	mvfR	amiR (AL347_25935)	amiR	?	31270321
mvfR (AL347_08190)	mvfR	amiC (AL347_25930)	amiC	?	31270321
mvfR (AL347_08190)	mvfR	PA3375 (AL347_25855)	PA3375	?	31270321
mvfR (AL347_08190)	mvfR	rhlR (AL347_25325)	rhlR	?	31270321
mvfR (AL347_08190)	mvfR	PA3592 (AL347_24825)	PA3592	?	31270321
mvfR (AL347_08190)	mvfR	PA3740 (AL347_24070)	PA3740	?	31270321
mvfR (AL347_08190)	mvfR	PA3759 (AL347_23975)	PA3759	?	31270321
mvfR (AL347_08190)	mvfR	nagE (AL347_23965)	nagE	?	31270321
mvfR (AL347_08190)	mvfR	PA3840 (AL347_23540)	PA3840	?	31270321
mvfR (AL347_08190)	mvfR	eddB (AL347_23180)	eddB	?	31270321
mvfR (AL347_08190)	mvfR	PA4112 (AL347_22135)	PA4112	?	31270321
mvfR (AL347_08190)	mvfR	pchG (AL347_21550)	pchG	?	31270321
mvfR (AL347_08190)	mvfR	PA4289 (AL347_10280)	PA4289	?	31270321
mvfR (AL347_08190)	mvfR	PA4291 (AL347_10290)	PA4291	?	31270321
mvfR (AL347_08190)	mvfR	PA4340 (AL347_10545)	PA4340	?	31270321
mvfR (AL347_08190)	mvfR	PA4611 (AL347_12550)	PA4611	?	31270321
mvfR (AL347_08190)	mvfR	PA4676 (AL347_12920)	PA4676	?	31270321
mvfR (AL347_08190)	mvfR	PA4735 (AL347_13250)	PA4735	?	31270321
mvfR (AL347_08190)	mvfR	aruI (AL347_14510)	aruI	?	31270321
mvfR (AL347_08190)	mvfR	PA4987 (AL347_14565)	PA4987	?	31270321
mvfR (AL347_08190)	mvfR	PA4992 (AL347_14590)	PA4992	?	31270321
mvfR (AL347_08190)	mvfR	PA5136 (AL347_15335)	PA5136	?	31270321
mvfR (AL347_08190)	mvfR	PA5210 (AL347_15785)	PA5210	?	31270321
mvfR (AL347_08190)	mvfR	PA5543 (AL347_17535)	PA5543	?	31270321

mvfR (AL347_08190)	mvfR	pqsA (AL347_08225)	pqsA	+	18974177, 31270321
mvfR (AL347_08190)	mvfR	pqsB (AL347_08220)	pqsB	+	18974177, 31270321
mvfR (AL347_08190)	mvfR	pqsC (AL347_08215)	pqsC	+	18974177, 31270321
mvfR (AL347_08190)	mvfR	pqsD (AL347_08210)	pqsD	+	18974177, 31270321
mvfR (AL347_08190)	mvfR	pqsE (AL347_08205)	pqsE	+	18974177, 31270321
mvfR (AL347_08190)	mvfR	mvfR (AL347_08190)	mvfR	-	18974177, 31270321
mvfR (AL347_08190)	mvfR	rsmA (AL347_08970)	rsmA	?	27242034*
mvfR (AL347_08190)	mvfR	mexH (AL347_21650)	mexH	?	27242034*
mvfR (AL347_08190)	mvfR	opmD (AL347_21640)	opmD	?	27242034*
mvfR (AL347_08190)	mvfR	mexI (AL347_21645)	mexI	?	27242034*
mvfR (AL347_08190)	mvfR	mexG (AL347_21655)	mexG	?	27242034*
nalC (AL347_24170)	nalC	mexB (AL347_19950)	mexB	-	15387820
nalC (AL347_24170)	nalC	oprM (AL347_19955)	oprM	-	15387820
nalC (AL347_24170)	nalC	armR (AL347_24180)	armR	-	22393435
nalC (AL347_24170)	nalC	mexA (AL347_19946)	mexA	-	27242034*, 18974177*, 22587778
nalD (AL347_24930)	nalD	mexB (AL347_19950)	mexB	?	27242034*
nalD (AL347_24930)	nalD	oprM (AL347_19955)	oprM	?	27242034*
nalD (AL347_24930)	nalD	mexA (AL347_19946)	mexA	?	27242034*
narL (AL347_23345)	narL	nirQ (AL347_20435)	nirQ	+	17400734
narL (AL347_23345)	narL	narI (AL347_23380)	narI	+	17400734
narL (AL347_23345)	narL	narJ (AL347_23375)	narJ	+	17400734
narL (AL347_23345)	narL	narH (AL347_23370)	narH	+	17400734
narL (AL347_23345)	narL	narG (AL347_23365)	narG	+	17400734
narL (AL347_23345)	narL	nark2 (AL347_23360)	nark2	+	17400734
narL (AL347_23345)	narL	nark1 (AL347_23355)	nark1	+	17400734
narL (AL347_23345)	narL	hemA (AL347_12850)	hemA	d	27242034*, 18974177*, 22587778
narL (AL347_23345)	narL	hemK (AL347_12840)	hemK	d	27242034*, 18974177*, 22587778
narL (AL347_23345)	narL	moeB (AL347_12835)	moeB	d	27242034*, 18974177*, 22587778
narL (AL347_23345)	narL	murI (AL347_12830)	murI	d	27242034*, 18974177*, 22587778
narL (AL347_23345)	narL	prfA (AL347_12845)	prfA	d	27242034*, 18974177*, 22587778
narL (AL347_23345)	narL	arcD (AL347_15575)	arcD	?	27242034*, 18974177*, 22587778
narL (AL347_23345)	narL	arcC (AL347_15590)	arcC	?	27242034*, 18974177*, 22587778
narL (AL347_23345)	narL	arcB (AL347_15585)	arcB	?	27242034*, 18974177*, 22587778
narL (AL347_23345)	narL	arcA (AL347_15580)	arcA	?	27242034*, 18974177*, 22587778
nfxB (AL347_12495)	nfxB	oprJ (AL347_12480)	oprJ	-	23924707
nfxB (AL347_12495)	nfxB	mexD (AL347_12485)	mexD	-	23924707
nfxB (AL347_12495)	nfxB	mexC (AL347_12490)	mexC	-	23924707
nfxB (AL347_12495)	nfxB	nfxB (AL347_12495)	nfxB	-	27242034*

np20 (AL347_17315)	np20	np20 (AL347_17315)	np20	-	27242034*
np20 (AL347_17315)	np20	znuC (AL347_17320)	znuC	-	27242034*
np20 (AL347_17315)	np20	znuB (AL347_17325)	znuB	-	27242034*
np20 (AL347_17315)	np20	PA0781 (AL347_09610)	PA0781	-	27242034*
np20 (AL347_17315)	np20	PA3601 (AL347_24775)	PA3601	-	27242034*
np20 (AL347_17315)	np20	PA3600 (AL347_24780)	PA3600	-	27242034*
np20 (AL347_17315)	np20	PA5536 (AL347_17500)	PA5536 (dksA2)	-	27242034*
np20 (AL347_17315)	np20	PA5535 (AL347_17495)	PA5535	-	27242034*
np20 (AL347_17315)	np20	PA5534 (AL347_17490)	PA5534	-	27242034*
np20 (AL347_17315)	np20	PA4838 (AL347_13795)	PA4838	-	27242034*
np20 (AL347_17315)	np20	PA5537 (AL347_17505)	PA5537	-	27242034*
np20 (AL347_17315)	np20	PA5539 (AL347_17515)	PA5539	-	27242034*
np20 (AL347_17315)	np20	PA5540 (AL347_17520)	PA5540	-	27242034*
np20 (AL347_17315)	np20	pyrQ (AL347_17525)	pyrQ	-	27242034*
np20 (AL347_17315)	np20	amiA (AL347_17510)	amiA	-	27242034*
np20 (AL347_17315)	np20	PA4063 (AL347_22396)	PA4063	-	27242034*
np20 (AL347_17315)	np20	PA5498 (AL347_17310)	PA5498 (znuA)	-	27242034*
nrdR (AL347_22425)	nrdR	nrdD (AL347_03000)	nrdD	-	27242034*
nrdR (AL347_22425)	nrdR	nrdG (AL347_03010)	nrdG	-	27242034*
nrdR (AL347_22425)	nrdR	nrdJb (AL347_17300)	nrdJb	-	27242034*
nrdR (AL347_22425)	nrdR	nrdJa (AL347_17305)	nrdJa	-	27242034*
nrdR (AL347_22425)	nrdR	nrdA (AL347_07355)	nrdA	-	27242034*
nrdR (AL347_22425)	nrdR	nrdB (AL347_07360)	nrdB	-	27242034*
nrdR (AL347_22425)	nrdR	topA (AL347_27805)	topA	-	27242034*
ntrC (AL347_15275)	ntrC	ntrB (AL347_15270)	ntrB	+	27242034*, 18974177*, 22587778
ntrC (AL347_15275)	ntrC	ntrC (AL347_15275)	ntrC	+	27242034*, 18974177*, 22587778
ntrC (AL347_15275)	ntrC	PA1730 (AL347_04045)	PA1730	+	27242034*, 18974177*, 22587778
ntrC (AL347_15275)	ntrC	PA1731 (AL347_04040)	PA1731	+	27242034*, 18974177*, 22587778
ntrC (AL347_15275)	ntrC	PA1732 (AL347_04035)	PA1732	+	27242034*, 18974177*, 22587778
ntrC (AL347_15275)	ntrC	glnA (AL347_15245)	glnA	+	27242034*, 18974177*, 22587778
ntrC (AL347_15275)	ntrC	glnK (AL347_16195)	glnK	+	27242034*, 18974177*, 22587778
ntrC (AL347_15275)	ntrC	amtB (AL347_16190)	amtB	+	27242034*, 18974177*, 22587778
ospR (AL347_28765)	ospR	PA2826 (AL347_28760)	PA2826	-	27242034*
oxyR (AL347_16475)	oxyR	pvdS (AL347_32895)	pvdS	?	29729420
oxyR (AL347_16475)	oxyR	katB (AL347_12560)	katB	?	27242034*
oxyR (AL347_16475)	oxyR	PA0848 (AL347_09270)	PA0848 (ahpB)	?	27242034*
oxyR (AL347_16475)	oxyR	PA4612 (AL347_12555)	PA4612	?	27242034*
PA0120 (AL347_18375)	PA0120	dctA (AL347_07215)	dctA	-	27242034*

PA0120 (AL347_18375)	PA0120	PA0120 (AL347_18375)	PA0120	-	27242034*
PA0149 (AL347_18525)	PA0149	PA2384 (AL347_33095)	PA2384	?	29729420
PA0167 (AL347_01365)	PA0167	PA1517 (AL347_05175)	PA1517	-	27242034*, 18974177*, 22587778
PA0167 (AL347_01365)	PA0167	PA1516 (AL347_05180)	PA1516	-	27242034*, 18974177*, 22587778
PA0167 (AL347_01365)	PA0167	alc (AL347_05185)	alc	-	27242034*, 18974177*, 22587778
PA0167 (AL347_01365)	PA0167	PA1514 (AL347_05190)	PA1514	-	27242034*, 18974177*, 22587778
PA0167 (AL347_01365)	PA0167	PA1513 (AL347_05195)	PA1513	-	27242034*, 18974177*, 22587778
PA0167 (AL347_01365)	PA0167	PA0136 (AL347_18455)	PA0136	-	27242034*, 18974177*, 22587778
PA0167 (AL347_01365)	PA0167	PA0137 (AL347_18460)	PA0137	-	27242034*, 18974177*, 22587778
PA0167 (AL347_01365)	PA0167	PA0138 (AL347_18465)	PA0138	-	27242034*, 18974177*, 22587778
PA0167 (AL347_01365)	PA0167	PA0165 (AL347_18610)	PA0165	-	27242034*, 18974177*, 22587778
PA0167 (AL347_01365)	PA0167	PA0166 (AL347_18615)	PA0166	-	27242034*, 18974177*, 22587778
PA0167 (AL347_01365)	PA0167	PA0167 (AL347_18620)	PA0167	-	27242034*, 18974177*, 22587778
PA0167 (AL347_01365)	PA0167	PA0168 (AL347_18625)	PA0168	-	27242034*, 18974177*, 22587778
PA0268 (AL347_19125)	PA0268	PA0269 (AL347_19130)	PA0269	-	27242034*
PA0268 (AL347_19125)	PA0268	PA0270 (AL347_19135)	PA0270	-	27242034*
PA0436 (AL347_20000)	PA0436	PA0443 (AL347_20045)	PA0443	-	27242034*
PA0436 (AL347_20000)	PA0436	PA0444 (AL347_20050)	PA0444	-	27242034*
PA0436 (AL347_20000)	PA0436	dht (AL347_20030)	dht	-	27242034*
PA0436 (AL347_20000)	PA0436	PA0440 (AL347_20025)	PA0440	-	27242034*
PA0436 (AL347_20000)	PA0436	PA0439 (AL347_20020)	PA0439	-	27242034*
PA0436 (AL347_20000)	PA0436	codB (AL347_20010)	codB	-	27242034*
PA0436 (AL347_20000)	PA0436	codA (AL347_20005)	codA	-	27242034*
PA0547 (AL347_20580)	PA0547	PA0547 (AL347_20580)	PA0547	-	27242034*, 18974177*, 22587778
PA0547 (AL347_20580)	PA0547	metK (AL347_20575)	metK	-	27242034*, 18974177*, 22587778
PA0779 (AL347_09620)	PA0779	fhp (AL347_30090)	fhp	+	27242034*, 18974177*, 22587778
PA0779 (AL347_09620)	PA0779	fhpR (AL347_30085)	fhpR	-	27242034*, 18974177*, 22587778
PA0797 (AL347_09525)	PA0797	PA0797 (AL347_09525)	PA0797	-	27242034*
PA0797 (AL347_09525)	PA0797	prpB (AL347_09530)	prpB	-	27242034*
PA0797 (AL347_09525)	PA0797	prpC (AL347_09535)	prpC	-	27242034*
PA0797 (AL347_09525)	PA0797	PA0794 (AL347_09540)	PA0794	-	27242034*
PA0797 (AL347_09525)	PA0797	PA0793 (AL347_09545)	PA0793	-	27242034*
PA0797 (AL347_09525)	PA0797	prpD (AL347_09550)	prpD	-	27242034*
PA1050 (AL347_07935)	PA1050	PA1051 (AL347_07930)	PA1051	+	27242034*
PA1050 (AL347_07935)	PA1050	PA1052 (AL347_07925)	PA1052	+	27242034*
PA1269 (AL347_06765)	PA1269	PA1269 (AL347_06765)	PA1269	+	27242034*
PA1269 (AL347_06765)	PA1269	lhpA (AL347_06770)	lhpA	+	27242034*
PA1269 (AL347_06765)	PA1269	lhpB (AL347_06775)	lhpB	+	27242034*

PA1269 (AL347_06765)	PA1269	lhpP (AL347_06820)	lhpP	+	27242034*
PA1269 (AL347_06765)	PA1269	PA1259 (AL347_06825)	PA1259 (lhpH)	+	27242034*
PA1269 (AL347_06765)	PA1269	lhpM (AL347_06830)	lhpM	+	27242034*
PA1269 (AL347_06765)	PA1269	lhpN (AL347_06835)	lhpN	+	27242034*
PA1269 (AL347_06765)	PA1269	lhpO (AL347_06840)	lhpO	+	27242034*
PA1269 (AL347_06765)	PA1269	PA1255 (AL347_06845)	PA1255 (lhpK)	+	27242034*
PA1269 (AL347_06765)	PA1269	PA1254 (AL347_06850)	PA1254 (lhpC)	+	27242034*
PA1269 (AL347_06765)	PA1269	PA1253 (AL347_06855)	PA1253 (lhpG)	+	27242034*
PA1504 (AL347_05240)	PA1504	xdhA (AL347_05135)	xdhA	-	27242034*, 18974177*, 22587778
PA1504 (AL347_05240)	PA1504	xdhB (AL347_05140)	xdhB	-	27242034*, 18974177*, 22587778
PA1504 (AL347_05240)	PA1504	PA1522 (AL347_05145)	PA1522	-	27242034*, 18974177*, 22587778
PA1504 (AL347_05240)	PA1504	PA1521 (AL347_05150)	PA1521	-	27242034*, 18974177*, 22587778
PA1504 (AL347_05240)	PA1504	PA1519 (AL347_05165)	PA1519	-	27242034*, 18974177*, 22587778
PA1520 (AL347_05155)	PA1520	PA2938 (AL347_28190)	PA2938	-	27242034*, 18974177*, 22587778
PA1520 (AL347_05155)	PA1520	PA1520 (AL347_05155)	PA1520	-	27242034*, 18974177*, 22587778
PA1520 (AL347_05155)	PA1520	PA1518 (AL347_05170)	PA1518	-	27242034*, 18974177*, 22587778
PA1520 (AL347_05155)	PA1520	gcl (AL347_05250)	gcl	-	27242034*, 18974177*, 22587778
PA1520 (AL347_05155)	PA1520	PA1501 (AL347_05255)	PA1501	-	27242034*, 18974177*, 22587778
PA1520 (AL347_05155)	PA1520	PA1500 (AL347_05260)	PA1500	-	27242034*, 18974177*, 22587778
PA1520 (AL347_05155)	PA1520	PA0165 (AL347_18610)	PA0165	-	27242034*, 18974177*, 22587778
PA1520 (AL347_05155)	PA1520	PA1503 (AL347_05245)	PA1503	-	27242034*, 18974177*, 22587778
PA1520 (AL347_05155)	PA1520	PA1507 (AL347_05225)	PA1507	-	27242034*, 18974177*, 22587778
PA1520 (AL347_05155)	PA1520	PA1517 (AL347_05175)	PA1517	-	27242034*, 18974177*, 22587778
PA1520 (AL347_05155)	PA1520	PA1516 (AL347_05180)	PA1516	-	27242034*, 18974177*, 22587778
PA1520 (AL347_05155)	PA1520	PA1514 (AL347_05190)	PA1514	-	27242034*, 18974177*, 22587778
PA1520 (AL347_05155)	PA1520	PA1513 (AL347_05195)	PA1513	-	27242034*, 18974177*, 22587778
PA1520 (AL347_05155)	PA1520	PA0476 (AL347_20215)	PA0476	-	27242034*, 18974177*, 22587778
PA1520 (AL347_05155)	PA1520	PA1519 (AL347_05165)	PA1519	-	27242034*, 18974177*, 22587778
PA1520 (AL347_05155)	PA1520	alc (AL347_05185)	alc	-	27242034*, 18974177*, 22587778
PA1539 (AL347_05056)	PA1539	PA1539 (AL347_05056)	PA1539	-	27242034*
PA1539 (AL347_05056)	PA1539	PA1538 (AL347_05060)	PA1538	-	27242034*
PA1539 (AL347_05056)	PA1539	PA1537 (AL347_05065)	PA1537	-	27242034*
hmgR (AL347_02560)	hmgR	hmgR (AL347_02560)	hmgR	-	27242034*
hmgR (AL347_02560)	hmgR	fahA (AL347_02570)	fahA	-	27242034*
hmgR (AL347_02560)	hmgR	maiA (AL347_02575)	maiA	-	27242034*
hmgR (AL347_02560)	hmgR	PA2006 (AL347_02580)	PA2006	-	27242034*
PA2032 (AL347_02445)	PA2032	PA2031 (AL347_02450)	PA2031	-	27242034*
PA2050 (AL347_02355)	PA2050	PA2384 (AL347_33095)	PA2384	?	29729420

PA2206 (AL347_01520)	PA2206	fiuI (AL347_20195)	fiuI	?	29729420
PA2206 (AL347_01520)	PA2206	femI (AL347_03050)	femI	?	29729420
PA2206 (AL347_01520)	PA2206	pvdS (AL347_32895)	pvdS	?	29729420
PA2299 (AL347_33530)	PA2299	PA2299 (AL347_33530)	PA2299	-	27242034*
PA2299 (AL347_33530)	PA2299	PA2298 (AL347_33535)	PA2298	-	27242034*
PA2299 (AL347_33530)	PA2299	PA2297 (AL347_33540)	PA2297	-	27242034*
PA2299 (AL347_33530)	PA2299	PA2296 (AL347_33545)	PA2296	-	27242034*
PA2299 (AL347_33530)	PA2299	PA2295 (AL347_33550)	PA2295	-	27242034*
PA2299 (AL347_33530)	PA2299	PA2294 (AL347_33555)	PA2294	-	27242034*
PA2299 (AL347_33530)	PA2299	PA2293 (AL347_33560)	PA2293	-	27242034*
PA2299 (AL347_33530)	PA2299	PA2292 (AL347_33565)	PA2292	-	27242034*
PA2384 (AL347_33095)	PA2384	vreI (AL347_10175)	vreI	?	29729420
PA2384 (AL347_33095)	PA2384	PA1300 (AL347_06610)	PA1300 (hxuI)	?	29729420
PA2384 (AL347_33095)	PA2384	sigX (AL347_03799)	sigX	?	29729420
PA2384 (AL347_33095)	PA2384	pvdS (AL347_32895)	pvdS	?	29729420
PA2384 (AL347_33095)	PA2384	hasI (AL347_25675)	hasI	?	29729420
PA2384 (AL347_33095)	PA2384	PA4896 (AL347_14090)	PA4896	?	29729420
PA2449 (AL347_32765)	PA2449	gcvH2 (AL347_32780)	gcvH2	+	27242034*
PA2449 (AL347_32765)	PA2449	gcvP2 (AL347_32785)	gcvP2	+	27242034*
PA2449 (AL347_32765)	PA2449	glyA2 (AL347_32795)	glyA2	+	27242034*
PA2449 (AL347_32765)	PA2449	sdaA (AL347_32800)	sdaA	+	27242034*
PA2449 (AL347_32765)	PA2449	gcvT2 (AL347_32805)	gcvT2	+	27242034*
PA2591 (AL347_30475)	PA2591	pprB (AL347_10315)	pprB	-	27242034*, 18974177*, 22587778
PA3184 (AL347_26900)	PA3184	PA3184 (AL347_26900)	PA3184	-	27242034*
PA3184 (AL347_26900)	PA3184	edd (AL347_00555)	edd	-	27242034*
PA3184 (AL347_26900)	PA3184	glk (AL347_00560)	glk	-	27242034*
PA3184 (AL347_26900)	PA3184	gltR (AL347_00565)	gltR	-	27242034*
PA3184 (AL347_26900)	PA3184	gltS (AL347_26940)	gltS	-	27242034*
PA3184 (AL347_26900)	PA3184	gapA (AL347_00550)	gapA	-	27242034*
PA3184 (AL347_26900)	PA3184	zwf (AL347_26905)	zwf	-	27242034*
PA3184 (AL347_26900)	PA3184	pgl (AL347_26910)	pgl	-	27242034*
PA3184 (AL347_26900)	PA3184	PA3181 (AL347_26915)	PA3181	-	27242034*
PA3249 (AL347_00275)	PA3249	PA3249 (AL347_00275)	PA3249	-	27242034*
PA3249 (AL347_00275)	PA3249	PA3250 (AL347_00270)	PA3250	-	27242034*
PA3249 (AL347_00275)	PA3249	PA3251 (AL347_00265)	PA3251	-	27242034*
PA3249 (AL347_00275)	PA3249	PA3252 (AL347_00260)	PA3252	-	27242034*
PA3249 (AL347_00275)	PA3249	PA3253 (AL347_00255)	PA3253	-	27242034*
PA3249 (AL347_00275)	PA3249	PA3254 (AL347_00250)	PA3254	-	27242034*

PA3249 (AL347_00275)	PA3249	PA3255 (AL347_00244)	PA3255	-	27242034*
PA3249 (AL347_00275)	PA3249	PA2802 (AL347_28890)	PA2802	-	27242034*
PA3249 (AL347_00275)	PA3249	PA2803 (AL347_28885)	PA2803	-	27242034*
PA3249 (AL347_00275)	PA3249	PA2804 (AL347_28880)	PA2804	-	27242034*
PA3249 (AL347_00275)	PA3249	PA1144 (AL347_04890)	PA1144	-	27242034*
PA3249 (AL347_00275)	PA3249	PA1143 (AL347_07440)	PA1143	-	27242034*
PA3249 (AL347_00275)	PA3249	PA1142 (AL347_07445)	PA1142	-	27242034*
PA3381 (AL347_25825)	PA3381	PA3381 (AL347_25825)	PA3381	-	27242034*
PA3381 (AL347_25825)	PA3381	PA3380 (AL347_25830)	PA3380	-	27242034*
PA3381 (AL347_25825)	PA3381	PA3379 (AL347_25835)	PA3379	-	27242034*
PA3381 (AL347_25825)	PA3381	PA3378 (AL347_25840)	PA3378	-	27242034*
PA3381 (AL347_25825)	PA3381	PA3377 (AL347_25845)	PA3377	-	27242034*
PA3381 (AL347_25825)	PA3381	PA3376 (AL347_25850)	PA3376	-	27242034*
PA3381 (AL347_25825)	PA3381	PA3375 (AL347_25855)	PA3375	-	27242034*
PA3381 (AL347_25825)	PA3381	PA3374 (AL347_25860)	PA3374	-	27242034*
PA3381 (AL347_25825)	PA3381	PA3373 (AL347_25865)	PA3373	-	27242034*
PA3381 (AL347_25825)	PA3381	PA3372 (AL347_25870)	PA3372	-	27242034*
PA3689 (AL347_24330)	PA3689	PA3690 (AL347_24325)	PA3690	+	27242034*
PA3697 (AL347_24290)	PA3697	fhp (AL347_30090)	fhp	+	19767835
PA3757 (AL347_23985)	PA3757 (nagR)	PA3757 (AL347_23985)	PA3757 (nagR)	-	27242034*
PA3757 (AL347_23985)	PA3757 (nagR)	PA3758 (AL347_23980)	PA3758	-	27242034*
PA3757 (AL347_23985)	PA3757 (nagR)	PA3759 (AL347_23975)	PA3759	-	27242034*
PA3757 (AL347_23985)	PA3757 (nagR)	PA3760 (AL347_23970)	PA3760	-	27242034*
PA3757 (AL347_23985)	PA3757 (nagR)	nagE (AL347_23965)	nagE	-	27242034*
PA4132 (AL347_22035)	PA4132 (mpaR)	cysI (AL347_03450)	cysI	-	27242034*
PA4132 (AL347_22035)	PA4132 (mpaR)	PA4129 (AL347_22050)	PA4129	-	27242034*
PA4132 (AL347_22035)	PA4132 (mpaR)	PA4133 (AL347_22030)	PA4133	-	27242034*
PA4165 (AL347_21855)	PA4165	PA4166 (AL347_21855)	PA4166	-	27242034*
PA4659 (AL347_12815)	PA4659	PA4657 (AL347_12805)	PA4657	-	27242034*
PA4659 (AL347_12815)	PA4659	PA4658 (AL347_12810)	PA4658	-	27242034*
PA4659 (AL347_12815)	PA4659	PA4659 (AL347_12815)	PA4659	-	27242034*
PA4659 (AL347_12815)	PA4659	phr (AL347_12820)	phr	-	27242034*
PA4769 (AL347_13440)	PA4769	PA4769 (AL347_13440)	PA4769	-	27242034*, 18974177*, 22587778
PA4769 (AL347_13440)	PA4769	lldP (AL347_13445)	lldP	-	27242034*, 18974177*, 22587778
PA4769 (AL347_13440)	PA4769	lldD (AL347_13450)	lldD	-	27242034*, 18974177*, 22587778
PA4769 (AL347_13440)	PA4769	PA4772 (AL347_13455)	PA4772	-	27242034*, 18974177*, 22587778
PA4906 (AL347_14140)	PA4906	vanA (AL347_14130)	vanA	-	27242034*
PA4906 (AL347_14140)	PA4906	vanB (AL347_14135)	vanB	-	27242034*

PA4906 (AL347_14140)	PA4906	PA4903 (AL347_14125)	PA4903	-	27242034*
PA4916 (AL347_14190)	PA4916 (nrtR)	nadD (AL347_22680)	nadD	-	27242034*, 18974177*, 22587778
PA4916 (AL347_14190)	PA4916 (nrtR)	PA4916 (AL347_14190)	PA4916 (nrtR)	-	27242034*, 18974177*, 22587778
PA4916 (AL347_14190)	PA4916 (nrtR)	pcnA (AL347_14200)	pcnA	-	27242034*, 18974177*, 22587778
PA4916 (AL347_14190)	PA4916 (nrtR)	pncB1 (AL347_14205)	pncB1	-	27242034*, 18974177*, 22587778
PA4916 (AL347_14190)	PA4916 (nrtR)	nadE (AL347_14210)	nadE	-	27242034*, 18974177*, 22587778
PA5431 (AL347_16955)	PA5431	PA5432 (AL347_16960)	PA5432	-	27242034*
PA5431 (AL347_16955)	PA5431	PA5433 (AL347_16965)	PA5433	-	27242034*
PA5438 (AL347_16995)	PA5438	aceA (AL347_30250)	aceA	?	31187593
PA5438 (AL347_16995)	PA5438	zwf (AL347_26905)	zwf	-	27242034*, 18974177*, 22587778
PA5438 (AL347_16995)	PA5438	aceE (AL347_14705)	aceE	-	27242034*, 18974177*, 22587778
PA5438 (AL347_16995)	PA5438	aceF (AL347_14715)	aceF	-	27242034*, 18974177*, 22587778
PA5438 (AL347_16995)	PA5438	PA5438 (AL347_16995)	PA5438	-	27242034*, 18974177*, 22587778
PA5438 (AL347_16995)	PA5438	PA3185 (AL347_26895)	PA3185	-	27242034*, 18974177*, 22587778
PA5506 (AL347_17350)	PA5506	PA5506 (AL347_17350)	PA5506	-	27242034*
PA5506 (AL347_17350)	PA5506	PA5507 (AL347_17355)	PA5507	-	27242034*
PA5506 (AL347_17350)	PA5506	PA5508 (AL347_17360)	PA5508 (pauA7)	-	27242034*
PA5506 (AL347_17350)	PA5506	PA5509 (AL347_17365)	PA5509	-	27242034*
PA5506 (AL347_17350)	PA5506	PA5510 (AL347_17370)	PA5510	-	27242034*
pchR (AL347_21535)	pchR	fptA (AL347_21565)	fptA	+	8626326
pchR (AL347_21535)	pchR	pchF (AL347_21545)	pchF	+	16194235
pchR (AL347_21535)	pchR	pchE (AL347_21540)	pchE	+	16194235
pchR (AL347_21535)	pchR	pchR (AL347_21535)	pchR	-	16194235
pchR (AL347_21535)	pchR	pchD (AL347_21530)	pchD	+	16194235
pchR (AL347_21535)	pchR	pchC (AL347_21525)	pchC	+	16194235
pchR (AL347_21535)	pchR	pchB (AL347_21520)	pchB	+	16194235
pchR (AL347_21535)	pchR	pchA (AL347_21515)	pchA	+	16194235
pchR (AL347_21535)	pchR	PA0042 (AL347_17975)	PA0042	?	31270321
pchR (AL347_21535)	pchR	phzH (AL347_18030)	phzH	?	31270321
pchR (AL347_21535)	pchR	PA0643 (AL347_21095)	PA0643	?	31270321
pchR (AL347_21535)	pchR	PA0981 (AL347_08300)	PA0981	?	31270321
pchR (AL347_21535)	pchR	PA0984 (AL347_08280)	PA0984	?	31270321
pchR (AL347_21535)	pchR	mvaU (AL347_30075)	mvaU	?	31270321
pchR (AL347_21535)	pchR	PA2698 (AL347_29920)	PA2698	?	31270321
pchR (AL347_21535)	pchR	PA2795 (AL347_28925)	PA2795 (dusA, yjbN)	?	31270321
pchR (AL347_21535)	pchR	acpP (AL347_28040)	acpP	?	31270321
pchR (AL347_21535)	pchR	rocsS2 (AL347_27630)	rocsS2	?	31270321
pchR (AL347_21535)	pchR	rocA2 (AL347_27625)	rocA2	?	31270321

pchR (AL347_21535)	pchR	fimV (AL347_27265)	fimV	?	31270321
pchR (AL347_21535)	pchR	wbpM (AL347_27110)	wbpM	?	31270321
pchR (AL347_21535)	pchR	wbpH (AL347_27080)	wbpH	?	31270321
pchR (AL347_21535)	pchR	wbpG (AL347_27075)	wbpG	?	31270321
pchR (AL347_21535)	pchR	hisF2 (AL347_27070)	hisF2	?	31270321
pchR (AL347_21535)	pchR	hisH2 (AL347_27065)	hisH2	?	31270321
pchR (AL347_21535)	pchR	wzz (AL347_27025)	wzz	?	31270321
pchR (AL347_21535)	pchR	PA3190 (AL347_00580)	PA3190	?	31270321
pchR (AL347_21535)	pchR	PA3327 (AL347_26120)	PA3327	?	31270321
pchR (AL347_21535)	pchR	PA3520 (AL347_25210)	PA3520	?	31270321
pchR (AL347_21535)	pchR	bfrB (AL347_25155)	bfrB	?	31270321
pchR (AL347_21535)	pchR	algD (AL347_25110)	algD	?	31270321
pchR (AL347_21535)	pchR	arnB (AL347_25045)	arnB	?	31270321
pchR (AL347_21535)	pchR	PA3691 (AL347_24320)	PA3691	?	31270321
pchR (AL347_21535)	pchR	PA3835 (AL347_23570)	PA3835	?	31270321
pchR (AL347_21535)	pchR	PA3836 (AL347_23560)	PA3836	?	31270321
pchR (AL347_21535)	pchR	PA4080 (AL347_22305)	PA4080	?	31270321
pchR (AL347_21535)	pchR	PA4087 (AL347_22270)	PA4087	?	31270321
pchR (AL347_21535)	pchR	PA4139 (AL347_21995)	PA4139	?	31270321
pchR (AL347_21535)	pchR	phzA1 (AL347_21625)	phzA1	?	31270321
pchR (AL347_21535)	pchR	phzB1 (AL347_21620)	phzB1	?	31270321
pchR (AL347_21535)	pchR	phzC1 (AL347_21615)	phzC1	?	31270321
pchR (AL347_21535)	pchR	phzD1 (AL347_21610)	phzD1	?	31270321
pchR (AL347_21535)	pchR	phzE1 (AL347_21605)	phzE1	?	31270321
pchR (AL347_21535)	pchR	phzF1 (AL347_21600)	phzF1	?	31270321
pchR (AL347_21535)	pchR	rpoC (AL347_21325)	rpoC	?	31270321
pchR (AL347_21535)	pchR	arcD (AL347_15575)	arcD	?	31270321
pchR (AL347_21535)	pchR	PA5232 (AL347_15900)	PA5232	?	31270321
pepA (AL347_23595)	pepA	algD (AL347_25110)	algD	?	27242034*, 18974177*, 22587778
pfeR (AL347_29975)	pfeR	pfeA (AL347_29965)	pfeA	+	8808923
phhR (AL347_09140)	phhR	hpd (AL347_09180)	hpd	+	8939433
phhR (AL347_09140)	phhR	phhC (AL347_09155)	phhC	+	8939433
phhR (AL347_09140)	phhR	phhB (AL347_08195)	phhB	+	8939433
phhR (AL347_09140)	phhR	phnA (AL347_08200)	phnA	+	8939433
phhR (AL347_09140)	phhR	dhcA (AL347_02615)	dhcA	+	8939433
phhR (AL347_09140)	phhR	hmgA (AL347_02565)	hmgA	+	8939433
phhR (AL347_09140)	phhR	phhR (AL347_09140)	phhR	-	27242034*
phoB (AL347_16560)	phoB	vreI (AL347_10175)	vreI	?	29729420

phoB (AL347_16560)	phoB	PA0165 (AL347_18610)	PA0165	?	31270321
phoB (AL347_16560)	phoB	phdA (AL347_10095)	phdA	?	31270321
phoB (AL347_16560)	phoB	PA0959 (AL347_08410)	PA0959	?	31270321
phoB (AL347_16560)	phoB	qscR (AL347_03130)	qscR	?	31270321
phoB (AL347_16560)	phoB	mvaU (AL347_30075)	mvaU	?	31270321
phoB (AL347_16560)	phoB	PA2793 (AL347_29170)	PA2793	?	31270321
phoB (AL347_16560)	phoB	rocsS2 (AL347_27630)	rocsS2	?	31270321
phoB (AL347_16560)	phoB	rocA2 (AL347_27625)	rocA2	?	31270321
phoB (AL347_16560)	phoB	wbpH (AL347_27080)	wbpH	?	31270321
phoB (AL347_16560)	phoB	wbpG (AL347_27075)	wbpG	?	31270321
phoB (AL347_16560)	phoB	PA3190 (AL347_00580)	PA3190	?	31270321
phoB (AL347_16560)	phoB	oprP (AL347_26390)	oprP	?	31270321
phoB (AL347_16560)	phoB	PA3327 (AL347_26120)	PA3327	?	31270321
phoB (AL347_16560)	phoB	phnC (AL347_25810)	phnC	?	31270321
phoB (AL347_16560)	phoB	amrZ (AL347_25805)	amrZ	?	31270321
phoB (AL347_16560)	phoB	rhlR (AL347_25325)	rhlR	?	31270321
phoB (AL347_16560)	phoB	PA3519 (AL347_25215)	PA3519	?	31270321
phoB (AL347_16560)	phoB	PA3520 (AL347_25210)	PA3520	?	31270321
phoB (AL347_16560)	phoB	algD (AL347_25110)	algD	?	31270321
phoB (AL347_16560)	phoB	arnB (AL347_25045)	arnB	?	31270321
phoB (AL347_16560)	phoB	PA3835 (AL347_23570)	PA3835	?	31270321
phoB (AL347_16560)	phoB	PA4080 (AL347_22305)	PA4080	?	31270321
phoB (AL347_16560)	phoB	cupB3 (AL347_22285)	cupB3	?	31270321
phoB (AL347_16560)	phoB	PA4087 (AL347_22270)	PA4087	?	31270321
phoB (AL347_16560)	phoB	PA4139 (AL347_21995)	PA4139	?	31270321
phoB (AL347_16560)	phoB	nmoA (AL347_21670)	nmoA	?	31270321
phoB (AL347_16560)	phoB	phzA1 (AL347_21625)	phzA1	?	31270321
phoB (AL347_16560)	phoB	phzB1 (AL347_21620)	phzB1	?	31270321
phoB (AL347_16560)	phoB	phzC1 (AL347_21615)	phzC1	?	31270321
phoB (AL347_16560)	phoB	phzD1 (AL347_21610)	phzD1	?	31270321
phoB (AL347_16560)	phoB	phzE1 (AL347_21605)	phzE1	?	31270321
phoB (AL347_16560)	phoB	phzF1 (AL347_21600)	phzF1	?	31270321
phoB (AL347_16560)	phoB	rplC (AL347_21355)	rplC	?	31270321
phoB (AL347_16560)	phoB	rcpC (AL347_10360)	rcpC	?	31270321
phoB (AL347_16560)	phoB	ctpL (AL347_13830)	ctpL	?	31270321
phoB (AL347_16560)	phoB	opgG (mdoG)	opgG	?	31270321
phoB (AL347_16560)	phoB	pstC (AL347_16600)	pstC	?	31270321
phoB (AL347_16560)	phoB	SPA7_RS26570 (AL347_1383	PSPA7_RS26570 (PSPA7_5564)	?	27242034*

phoB (AL347_16560)	phoB	phoU (AL347_16585)	phoU	?	27242034*
phoP (AL347_07240)	phoP	oprH (AL347_07245)	oprH	+	10564474
phoP (AL347_07240)	phoP	phoP (AL347_07240)	phoP	+	10564474
phoP (AL347_07240)	phoP	phoQ (AL347_07235)	phoQ	+	10564474
phoP (AL347_07240)	phoP	gabD (AL347_19100)	gabD	+	16707691
phoP (AL347_07240)	phoP	davT (AL347_19110)	davT	+	16707691
phoP (AL347_07240)	phoP	PA0921 (AL347_08620)	PA0921	+	16707691
phoP (AL347_07240)	phoP	ddaR (AL347_07150)	ddaR	-	7691, 27242034*, 18974177*, 22587778
phoP (AL347_07240)	phoP	PA3309 (AL347_26210)	PA3309	-	16707691
phoP (AL347_07240)	phoP	mexQ (AL347_25200)	mexQ	+	7691, 27242034*, 18974177*, 22587778
phoP (AL347_07240)	phoP	mucP (AL347_24530)	mucP	+	7691, 27242034*, 18974177*, 22587778
phoP (AL347_07240)	phoP	tpbA (AL347_23315)	tpbA	+	16707691
phoP (AL347_07240)	phoP	sodB (AL347_10680)	sodB	+	16707691
phoP (AL347_07240)	phoP	PA4453 (AL347_11130)	PA4453	+	16707691
phoP (AL347_07240)	phoP	PA4454 (AL347_11135)	PA4454	+	16707691
phoP (AL347_07240)	phoP	PA4455 (AL347_11140)	PA4455	+	16707691
phoP (AL347_07240)	phoP	pcnA (AL347_14200)	pcnA	-	7691, 27242034*, 18974177*, 22587778
phoP (AL347_07240)	phoP	arnB (AL347_25045)	arnB	+	19243444
phoP (AL347_07240)	phoP	arnC (AL347_25040)	arnC	+	19243444
phoP (AL347_07240)	phoP	arnA (AL347_25035)	arnA	+	19243444
phoP (AL347_07240)	phoP	arnD (AL347_25030)	arnD	+	19243444
phoP (AL347_07240)	phoP	arnT (AL347_25025)	arnT	+	19243444
phoP (AL347_07240)	phoP	arnE (AL347_25020)	arnE	+	19243444
phoP (AL347_07240)	phoP	arnF (AL347_25015)	arnF	+	19243444
phoP (AL347_07240)	phoP	PA3559 (AL347_25010)	PA3559	+	19243444
phoP (AL347_07240)	phoP	PA4010 (AL347_22660)	PA4010	+	33572426
phoP (AL347_07240)	phoP	PA4011 (AL347_22655)	PA4011	+	33572426
phoP (AL347_07240)	phoP	gabT (AL347_19110)	gabT	+	27242034*, 18974177*, 22587778
phoP (AL347_07240)	phoP	ackA (AL347_09335)	ackA	-	27242034*, 18974177*, 22587778
phoP (AL347_07240)	phoP	PA1343 (AL347_06380)	PA1343	?	27242034*
phoQ (AL347_07235)	phoQ	oprH (AL347_07245)	oprH	+	10564474
phoQ (AL347_07235)	phoQ	phoP (AL347_07240)	phoP	+	10564474
phoQ (AL347_07235)	phoQ	phoQ (AL347_07235)	phoQ	+	10564474
phoQ (AL347_07235)	phoQ	algR (AL347_16050)	algR	-	27242034*, 18974177*, 22587778
phoQ (AL347_07235)	phoQ	arnB (AL347_25045)	arnB	-	27242034*, 18974177*, 22587778
phoQ (AL347_07235)	phoQ	pmrA (AL347_13475)	pmrA	-	27242034*, 18974177*, 22587778
pilR (AL347_12220)	pilR	pilA	pilA	-	27242034*
pilR (AL347_12220)	pilR	pilR (AL347_12220)	pilR	+	27242034*

pilR (AL347_12220)	pilR	pilS (AL347_12215)	pilS	+	27242034*
pmrA (AL347_13475)	pmrA	PA0201 (AL347_18800)	PA0201	+	16707691
pmrA (AL347_13475)	pmrA	cysT (AL347_19195)	cysT	+	16707691
pmrA (AL347_13475)	pmrA	dnr (AL347_20470)	dnr	+	16707691
pmrA (AL347_13475)	pmrA	metK (AL347_20575)	metK	+	16707691
pmrA (AL347_13475)	pmrA	mgtE (AL347_08915)	mgtE	+	16707691
pmrA (AL347_13475)	pmrA	pcoB (AL347_02285)	pcoB	-	16707691
pmrA (AL347_13475)	pmrA	pcoA (AL347_02280)	pcoA	-	16707691
pmrA (AL347_13475)	pmrA	PA2359 (AL347_33225)	PA2359	+	16707691
pmrA (AL347_13475)	pmrA	PA3515 (AL347_25235)	PA3515	-	16707691
pmrA (AL347_13475)	pmrA	PA3516 (AL347_25230)	PA3516	-	16707691
pmrA (AL347_13475)	pmrA	PA3517 (AL347_25225)	PA3517	-	16707691
pmrA (AL347_13475)	pmrA	PA3518 (AL347_25220)	PA3518	-	16707691
pmrA (AL347_13475)	pmrA	mexG (AL347_21655)	mexG	-	16707691
pmrA (AL347_13475)	pmrA	mexH (AL347_21650)	mexH	-	16707691
pmrA (AL347_13475)	pmrA	mexI (AL347_21645)	mexI	-	16707691
pmrA (AL347_13475)	pmrA	feoB (AL347_10635)	feoB	+	16707691
pmrA (AL347_13475)	pmrA	PA4359 (AL347_10640)	PA4359	+	16707691
pmrA (AL347_13475)	pmrA	speD2 (AL347_13460)	speD2	+	7691, 27242034*, 18974177*, 22587778
pmrA (AL347_13475)	pmrA	speE2 (AL347_13465)	speE2	+	7691, 27242034*, 18974177*, 22587778
pmrA (AL347_13475)	pmrA	PA4775 (AL347_13470)	PA4775	+	16707691
pmrA (AL347_13475)	pmrA	cueR (AL347_13485)	cueR	+	16707691
pmrA (AL347_13475)	pmrA	PA4781 (AL347_13505)	PA4781	+	16707691
pmrA (AL347_13475)	pmrA	PA4782 (AL347_13510)	PA4782	+	16707691
pmrA (AL347_13475)	pmrA	PA4822 (AL347_13715)	PA4822	+	16707691
pmrA (AL347_13475)	pmrA	PA4823 (AL347_13720)	PA4823	+	16707691
pmrA (AL347_13475)	pmrA	PA4824 (AL347_13725)	PA4824	+	16707691
pmrA (AL347_13475)	pmrA	mgtA (AL347_13730)	mgtA	+	16707691
pmrA (AL347_13475)	pmrA	PA4826 (AL347_13735)	PA4826	+	16707691
pmrA (AL347_13475)	pmrA	arnB (AL347_25045)	arnB	+	19243444
pmrA (AL347_13475)	pmrA	arnC (AL347_25040)	arnC	+	19243444
pmrA (AL347_13475)	pmrA	arnA (AL347_25035)	arnA	+	19243444
pmrA (AL347_13475)	pmrA	arnD (AL347_25030)	arnD	+	19243444
pmrA (AL347_13475)	pmrA	arnT (AL347_25025)	arnT	+	19243444
pmrA (AL347_13475)	pmrA	arnE (AL347_25020)	arnE	+	19243444
pmrA (AL347_13475)	pmrA	arnF (AL347_25015)	arnF	+	19243444
pmrA (AL347_13475)	pmrA	PA3559 (AL347_25010)	PA3559	+	19243444
pmrA (AL347_13475)	pmrA	pmrA (AL347_13475)	pmrA	+	19243444

pmrA (AL347_13475)	pmrA	pmrB (AL347_13480)	pmrB	+	19243444
pmrA (AL347_13475)	pmrA	feoA (AL347_10640)	feoA	+	27242034*, 18974177*, 22587778
pmrA (AL347_13475)	pmrA	PA3446 (AL347_25490)	PA3446	+	27242034*, 18974177*, 22587778
pmrA (AL347_13475)	pmrA	PA0202 (AL347_10555)	PA0202	+	27242034*, 18974177*, 22587778
pmrB (AL347_13480)	pmrB	pmrA (AL347_13475)	pmrA	+	19243444
pprB (AL347_10315)	pprB	vqsR (AL347_30475)	vqsR	+	2421, 27242034*, 18974177*, 22587778
pprB (AL347_10315)	pprB	rpoS (AL347_24665)	rpoS	+	15882421
pprB (AL347_10315)	pprB	bapB (AL347_03245)	bapB	+	31492668
pprB (AL347_10315)	pprB	bapC (AL347_03240)	bapC	+	31492668
pprB (AL347_10315)	pprB	bapD (AL347_03235)	bapD	+	31492668
pprB (AL347_10315)	pprB	pprA (AL347_10300)	pprA	+	31492668
pprB (AL347_10315)	pprB	PA4294 (AL347_10305)	PA4294	+	31492668
pprB (AL347_10315)	pprB	pprB (AL347_10315)	pprB	-	31492668
pprB (AL347_10315)	pprB	tadG (AL347_10320)	tadG	+	31492668
pprB (AL347_10315)	pprB	PA4298 (AL347_10325)	PA4298	+	31492668
pprB (AL347_10315)	pprB	tadD (AL347_10330)	tadD	+	31492668
pprB (AL347_10315)	pprB	tadB (AL347_10340)	tadB	+	31492668
pprB (AL347_10315)	pprB	tadA (AL347_10345)	tadA	+	31492668
pprB (AL347_10315)	pprB	rcpA (AL347_10355)	rcpA	+	31492668
pprB (AL347_10315)	pprB	rcpC (AL347_10360)	rcpC	+	31492668
pprB (AL347_10315)	pprB	flp (AL347_10365)	flp	+	31492668
pprB (AL347_10315)	pprB	cupE1 (AL347_12760)	cupE1	+	31492668
pprB (AL347_10315)	pprB	cupE2 (AL347_12765)	cupE2	+	31492668
pprB (AL347_10315)	pprB	cupE3 (AL347_12770)	cupE3	+	31492668
pprB (AL347_10315)	pprB	cupE4 (AL347_12775)	cupE4	+	31492668
pprB (AL347_10315)	pprB	cupE5 (AL347_12780)	cupE5	+	31492668
pprB (AL347_10315)	pprB	cupE6 (AL347_12785)	cupE6	+	31492668
pprB (AL347_10315)	pprB	tadZ (AL347_10350)	tadZ	+	31492668, 31492668
pprB (AL347_10315)	pprB	rsaL (AL347_05610)	rsaL	?	27242034*
pprB (AL347_10315)	pprB	fppA (AL347_10310)	fppA	?	27242034*
pprB (AL347_10315)	pprB	lasB (AL347_24155)	lasB	?	27242034*
pprB (AL347_10315)	pprB	lasI (AL347_05605)	lasI	?	27242034*
pprB (AL347_10315)	pprB	tadC (AL347_10335)	tadC	?	27242034*
ppyR (AL347_30095)	ppyR	fhp (AL347_30090)	fhp	+	19767835
ppyR (AL347_30095)	ppyR	lasB (AL347_24155)	lasB	-	27242034*, 18974177*, 22587778
pqrR (AL347_08505)	pqrR	PA0941 (AL347_08510)	PA0941	-	27242034*
pqrR (AL347_08505)	pqrR	PA0940 (AL347_08515)	PA0940	-	27242034*
pqrR (AL347_08505)	pqrR	PA0939 (AL347_08520)	PA0939	-	27242034*

pqrR (AL347_08505)	pqrR	pqrR (AL347_08505)	pqrR	+	27242034*
proE (AL347_16230)	proE	pslA (AL347_00910)	pslA	-	32582123
proE (AL347_16230)	proE	pelB (AL347_27530)	pelB	-	32582123
proE (AL347_16230)	proE	pelA (AL347_27525)	pelA	-	32582123
pruR (AL347_09615)	pruR	putA (AL347_09605)	putA	+	29632523
psdR (AL347_11360)	psdR	mdpA (AL347_11355)	mdpA	-	18667554
psdR (AL347_11360)	psdR	dppA3 (AL347_11365)	dppA3	-	18667554, 27242034*
psdR (AL347_11360)	psdR	dppA4 (AL347_11380)	dppA4	-	18667554
psdR (AL347_11360)	psdR	dppB (AL347_11385)	dppB	-	18667554
psdR (AL347_11360)	psdR	dppC (AL347_11390)	dppC	-	18667554
psdR (AL347_11360)	psdR	dppD	dppD	-	18667554
psdR (AL347_11360)	psdR	dppF (AL347_11400)	dppF	-	18667554
PSPA7_RS29685 (AL347_16985)	PSPA7_RS29685	oadA (AL347_16975)	oadA	?	27242034*, 18974177*, 22587778
PSPA7_RS29685 (AL347_16985)	PSPA7_RS29685	SPA7_RS01190 (AL347_1864	PSPA7_RS01190	?	27242034*, 18974177*, 22587778
PSPA7_RS29685 (AL347_16985)	PSPA7_RS29685	norC (AL347_20450)	norC	?	27242034*, 18974177*, 22587778
PSPA7_RS29685 (AL347_16985)	PSPA7_RS29685	SPA7_RS29685 (AL347_1698	PSPA7_RS29685	?	27242034*, 18974177*, 22587778
PSPA7_RS29685 (AL347_16985)	PSPA7_RS29685	nosZ (AL347_25770)	nosZ	?	27242034*, 18974177*, 22587778
psrA (AL347_27830)	psrA	exsC (AL347_04150)	exsC	+	19508282
psrA (AL347_27830)	psrA	exsE (AL347_04145)	exsE	+	19508282
psrA (AL347_27830)	psrA	exsB (AL347_04140)	exsB	+	19508282
psrA (AL347_27830)	psrA	exsA (AL347_04135)	exsA	+	19508282
psrA (AL347_27830)	psrA	fadB (AL347_04010)	fadB	-	19508282
psrA (AL347_27830)	psrA	rpoS (AL347_24665)	rpoS	+	19508282
psrA (AL347_27830)	psrA	fadE (AL347_20360)	fadE	-	29220387
psrA (AL347_27830)	psrA	psrA (AL347_27830)	psrA	-	29220387
psrA (AL347_27830)	psrA	etfA (AL347_28115)	etfA	-	27242034*, 18974177*, 22587778
psrA (AL347_27830)	psrA	etfB (AL347_28110)	etfB	-	27242034*, 18974177*, 22587778
psrA (AL347_27830)	psrA	exoS (AL347_23535)	exoS	+	27242034*, 18974177*, 22587778
psrA (AL347_27830)	psrA	mmsR (AL347_24945)	mmsR	+	27242034*, 18974177*, 22587778
psrA (AL347_27830)	psrA	PA0506 (AL347_20360)	PA0506	-	27242034*, 18974177*, 22587778
psrA (AL347_27830)	psrA	PA2953 (AL347_28105)	PA2953	-	27242034*, 18974177*, 22587778
psrA (AL347_27830)	psrA	PA3595 (AL347_24805)	PA3595	?	27242034*, 18974177*, 22587778
psrA (AL347_27830)	psrA	PA0507 (AL347_20365)	PA0507	-	27242034*, 18974177*, 22587778
psrA (AL347_27830)	psrA	PA0508 (AL347_20375)	PA0508	-	27242034*, 18974177*, 22587778
psrA (AL347_27830)	psrA	PA1831 (AL347_03485)	PA1831	-	27242034*, 18974177*, 22587778
psrA (AL347_27830)	psrA	PA1830 (AL347_03490)	PA1830	-	27242034*, 18974177*, 22587778
psrA (AL347_27830)	psrA	faoA (AL347_27790)	faoA	-	27242034*, 18974177*, 22587778
psrA (AL347_27830)	psrA	algQ (AL347_16020)	algQ	-	27242034*, 18974177*, 22587778

ptxR (AL347_33740)	ptxR	toxA (AL347_07415)	toxA	+	9645431
ptxR (AL347_33740)	ptxR	pqsA (AL347_08225)	pqsA	-	16803594
ptxR (AL347_33740)	ptxR	pqsB (AL347_08220)	pqsB	-	16803594
ptxR (AL347_33740)	ptxR	pqsC (AL347_08215)	pqsC	-	16803594
ptxR (AL347_33740)	ptxR	pqsD (AL347_08210)	pqsD	-	16803594
ptxR (AL347_33740)	ptxR	pqsE (AL347_08205)	pqsE	-	16803594
ptxR (AL347_33740)	ptxR	rhlI (AL347_25330)	rhlI	-	16803594
ptxR (AL347_33740)	ptxR	kguE (AL347_00760)	kguE	+	22844393
ptxR (AL347_33740)	ptxR	kguK (AL347_00760)	kguK	+	22844393
ptxR (AL347_33740)	ptxR	kguT (AL347_00750)	kguT	+	22844393
ptxR (AL347_33740)	ptxR	kguD (AL347_00745)	kguD	+	22844393
ptxR (AL347_33740)	ptxR	gad (AL347_00735)	gad	+	22844393
ptxR (AL347_33740)	ptxR	pvcA (AL347_00790)	pvcA	+	30528249
ptxR (AL347_33740)	ptxR	pvcB (AL347_00785)	pvcB	+	30528249
ptxR (AL347_33740)	ptxR	pvcC (AL347_00780)	pvcC	+	30528249
ptxR (AL347_33740)	ptxR	pvcD (AL347_33745)	pvcD	+	30528249
ptxR (AL347_33740)	ptxR	ptxS (AL347_00765)	ptxS	d	24019239, 10438759
ptxS (AL347_00765)	ptxS	ptxR (AL347_33740)	ptxR	-	22844393
ptxS (AL347_00765)	ptxS	kguE (AL347_00760)	kguE	+	22844393, 22587778
ptxS (AL347_00765)	ptxS	kguK (AL347_00755)	kguK	+	22844393
ptxS (AL347_00765)	ptxS	kguT (AL347_00750)	kguT	+	22844393
ptxS (AL347_00765)	ptxS	kguD (AL347_00745)	kguD	+	22844393
ptxS (AL347_00765)	ptxS	gad (AL347_00735)	gad	+	22844393
ptxS (AL347_00765)	ptxS	PA2263 (AL347_00745)	PA2263	+	27242034*, 18974177*, 22587778
ptxS (AL347_00765)	ptxS	ptxS (AL347_00765)	ptxS	-	27242034*, 18974177*, 22587778
ptxS (AL347_00765)	ptxS	PA2264 (AL347_00740)	PA2264	-	27242034*, 18974177*, 22587778
ptxS (AL347_00765)	ptxS	PA2265 (AL347_00735)	PA2265	-	27242034*, 18974177*, 22587778
ptxS (AL347_00765)	ptxS	PA2266 (AL347_00730)	PA2266	-	27242034*, 18974177*, 22587778
ptxS (AL347_00765)	ptxS	PA2262 (AL347_00750)	PA2262	+	27242034*, 18974177*, 22587778
pvdS (AL347_32895)	pvdS	toxA (AL347_07415)	toxA	+	10383985
pvdS (AL347_32895)	pvdS	pvcA (AL347_00790)	pvcA	+	10383985
pvdS (AL347_32895)	pvdS	pvcB (AL347_00785)	pvcB	+	10383985
pvdS (AL347_32895)	pvdS	pvcC (AL347_00780)	pvcC	+	10383985
pvdS (AL347_32895)	pvdS	pvcD (AL347_33745)	pvcD	+	10383985
pvdS (AL347_32895)	pvdS	pvdH (AL347_32960)	pvdH	+	10383985
pvdS (AL347_32895)	pvdS	PA4833 (AL347_13770)	PA4833	+	10383985
pvdS (AL347_32895)	pvdS	PA0346 (AL347_19535)	PA0346	+	12207696
pvdS (AL347_32895)	pvdS	PA0818 (AL347_09420)	PA0818	+	12207696

pvdS (AL347_32895)	pvdS	mvfR (AL347_08190)	mvfR	?	29729420
pvdS (AL347_32895)	pvdS	lasR (AL347_05615)	lasR	?	29729420
pvdS (AL347_32895)	pvdS	PA2383 (AL347_33100)	PA2383	?	29729420
pvdS (AL347_32895)	pvdS	rhlR (AL347_25325)	rhlR	?	29729420
pvdS (AL347_32895)	pvdS	ptxR (AL347_33740)	ptxR	+	10383985, 29729420
pvdS (AL347_32895)	pvdS	PA5204 (AL347_15755)	PA5204 (argA)	+	Homology from 25780925
pvdS (AL347_32895)	pvdS	PA5150 (AL347_15470)	PA5150	+	Homology from 25780925
pvdS (AL347_32895)	pvdS	PA5131 (AL347_15310)	PA5131 (pgm, yibO)	+	Homology from 25780925
pvdS (AL347_32895)	pvdS	PA5130 (AL347_15305)	PA5130	+	Homology from 25780925
pvdS (AL347_32895)	pvdS	PA5128 (AL347_15295)	PA5128 (secB)	+	Homology from 25780925
pvdS (AL347_32895)	pvdS	PA5106 (AL347_15175)	PA5106	+	Homology from 25780925
pvdS (AL347_32895)	pvdS	PA4796 (AL347_13580)	PA4796	+	Homology from 25780925
pvdS (AL347_32895)	pvdS	PA4795 (AL347_13575)	PA4795	+	Homology from 25780925
pvdS (AL347_32895)	pvdS	PA4794 (AL347_13570)	PA4794	+	Homology from 25780925
pvdS (AL347_32895)	pvdS	PA4793 (AL347_13565)	PA4793	+	Homology from 25780925
pvdS (AL347_32895)	pvdS	PA4656 (AL347_12800)	PA4656	+	Homology from 25780925
pvdS (AL347_32895)	pvdS	hemH (AL347_12795)	hemH	+	Homology from 25780925
pvdS (AL347_32895)	pvdS	PA4431 (AL347_11020)	PA4431	+	Homology from 25780925
pvdS (AL347_32895)	pvdS	PA4430 (AL347_11015)	PA4430	+	Homology from 25780925
pvdS (AL347_32895)	pvdS	PA4429 (AL347_11010)	PA4429	+	Homology from 25780925
pvdS (AL347_32895)	pvdS	PA4428 (AL347_11005)	PA4428 (sspA)	+	Homology from 25780925
pvdS (AL347_32895)	pvdS	PA4427 (AL347_11000)	PA4427 (sspB)	+	Homology from 25780925
pvdS (AL347_32895)	pvdS	PA4390 (AL347_10805)	PA4390	+	Homology from 25780925
pvdS (AL347_32895)	pvdS	PA4290 (AL347_10285)	PA4290	+	Homology from 25780925
pvdS (AL347_32895)	pvdS	PA4177 (AL347_21800)	PA4177	+	Homology from 25780925
pvdS (AL347_32895)	pvdS	PA4144 (AL347_21970)	PA4144	+	Homology from 25780925
pvdS (AL347_32895)	pvdS	PA4143 (AL347_21975)	PA4143	+	Homology from 25780925
pvdS (AL347_32895)	pvdS	PA4142 (AL347_21980)	PA4142	+	Homology from 25780925
pvdS (AL347_32895)	pvdS	PA4141 (AL347_21985)	PA4141	+	Homology from 25780925
pvdS (AL347_32895)	pvdS	PA3641 (AL347_24570)	PA3641	+	Homology from 25780925
pvdS (AL347_32895)	pvdS	PA3452 (AL347_25460)	PA3452 (mqoA)	+	Homology from 25780925
pvdS (AL347_32895)	pvdS	fprA (AL347_25745)	fprA	+	Homology from 25780925
pvdS (AL347_32895)	pvdS	PA3286 (AL347_00060)	PA3286	+	Homology from 25780925
pvdS (AL347_32895)	pvdS	PA3226 (AL347_00390)	PA3226	+	Homology from 25780925
pvdS (AL347_32895)	pvdS	PA3165 (AL347_26995)	PA3165 (hisC2)	+	Homology from 25780925
pvdS (AL347_32895)	pvdS	PA2912 (AL347_28325)	PA2912	+	Homology from 25780925
pvdS (AL347_32895)	pvdS	PA2567 (AL347_31795)	PA2567	+	Homology from 25780925
pvdS (AL347_32895)	pvdS	PA2531 (AL347_31990)	PA2531	+	Homology from 25780925

pvdS (AL347_32895)	pvdS	PA2427 (AL347_32890)	PA2427	+	Homology from 25780925
pvdS (AL347_32895)	pvdS	PA2412 (AL347_32965)	PA2412	+	Homology from 25780925
pvdS (AL347_32895)	pvdS	PA2411 (AL347_32970)	PA2411	+	Homology from 25780925
pvdS (AL347_32895)	pvdS	PA2406 (AL347_32995)	PA2406 (fpvK)	+	Homology from 25780925
pvdS (AL347_32895)	pvdS	PA2405 (AL347_33000)	PA2405 (fpvJ)	+	Homology from 25780925
pvdS (AL347_32895)	pvdS	PA2404 (AL347_33005)	PA2404 (fpvH)	+	Homology from 25780925
pvdS (AL347_32895)	pvdS	PA2395 (AL347_33040)	PA2395 (pvdO)	+	Homology from 25780925
pvdS (AL347_32895)	pvdS	PA2390 (AL347_33065)	PA2390 (pvdT)	+	Homology from 25780925
pvdS (AL347_32895)	pvdS	PA2389 (AL347_33070)	PA2389 (pvdR)	+	Homology from 25780925
pvdS (AL347_32895)	pvdS	pvdA (AL347_33085)	pvdA	+	Homology from 25780925
pvdS (AL347_32895)	pvdS	PA2290 (AL347_00605)	PA2290 (gcd)	+	Homology from 25780925
pvdS (AL347_32895)	pvdS	exsB (AL347_04140)	exsB	+	Homology from 25780925
pvdS (AL347_32895)	pvdS	PA1186 (AL347_07200)	PA1186	+	Homology from 25780925
pvdS (AL347_32895)	pvdS	PA1185 (AL347_07205)	PA1185	+	Homology from 25780925
pvdS (AL347_32895)	pvdS	PA1162 (AL347_07325)	PA1162 (dapE)	+	Homology from 25780925
pvdS (AL347_32895)	pvdS	PA1161 (AL347_07330)	PA1161 (rrmA)	+	Homology from 25780925
pvdS (AL347_32895)	pvdS	PA1160 (AL347_07335)	PA1160	+	Homology from 25780925
pvdS (AL347_32895)	pvdS	PA1133 (AL347_07490)	PA1133	+	Homology from 25780925
pvdS (AL347_32895)	pvdS	PA0938 (AL347_08530)	PA0938 (wzz2)	+	Homology from 25780925
pvdS (AL347_32895)	pvdS	PA0661 (AL347_21185)	PA0661	+	Homology from 25780925
pvdS (AL347_32895)	pvdS	PA0660 (AL347_21180)	PA0660	+	Homology from 25780925
pvdS (AL347_32895)	pvdS	exoT (AL347_17990)	exoT	+	Homology from 25780925
pvdS (AL347_32895)	pvdS	PA3285 (AL347_00065)	PA3285	+	Homology from 25780925
pvdS (AL347_32895)	pvdS	PA3225 (AL347_00395)	PA3225	+	Homology from 25780926
pvdS (AL347_32895)	pvdS	pvdS (AL347_32895)	pvdS	?	Homology from 25780927
pvdS (AL347_32895)	pvdS	exsA (AL347_04135)	exsA	+	Homology from 25780928
pvdS (AL347_32895)	pvdS	toxR (AL347_10010)	toxR	+	Homology from 25780929
pvdS (AL347_32895)	pvdS	cat (AL347_10015)	cat	+	27242034*, 18974177*, 22587778
pvdS (AL347_32895)	pvdS	grx (AL347_15300)	grx	+	27242034*, 18974177*, 22587778
pvdS (AL347_32895)	pvdS	imm2 (AL347_07400)	imm2	?	27242034*, 18974177*, 22587778
pvdS (AL347_32895)	pvdS	opmQ (AL347_33060)	opmQ	+	27242034*, 18974177*, 22587778
pvdS (AL347_32895)	pvdS	PA2393 (AL347_33050)	PA2393	+	27242034*, 18974177*, 22587778
pvdS (AL347_32895)	pvdS	PA3794 (AL347_23785)	PA3794	?	27242034*, 18974177*, 22587778
pvdS (AL347_32895)	pvdS	PA5190 (AL347_15680)	PA5190	?	27242034*, 18974177*, 22587778
pvdS (AL347_32895)	pvdS	piv (AL347_21810)	prpL	+	27242034*, 18974177*, 22587778
pvdS (AL347_32895)	pvdS	pvdE (AL347_33030)	pvdE	+	27242034*, 18974177*, 22587778
pvdS (AL347_32895)	pvdS	pvdF (AL347_33035)	pvdF	+	27242034*, 18974177*, 22587778
pvdS (AL347_32895)	pvdS	pvdG (AL347_32900)	pvdG	+	27242034*, 18974177*, 22587778

pvdS (AL347_32895)	pvdS	pvdN (AL347_33045)	pvdN	+	27242034*, 18974177*, 22587778
pvdS (AL347_32895)	pvdS	pvdL (AL347_32905)	pvdL	+	27242034*, 18974177*, 22587778
pyeR (AL347_10615)	pyeR	pyeR (AL347_10615)	pyeR	+	22820840
pyeR (AL347_10615)	pyeR	pyeM (AL347_10620)	pyeM	+	22820840
pyeR (AL347_10615)	pyeR	xenB (AL347_10625)	xenB	+	22820840
qscR (AL347_03130)	qscR	rsaL (AL347_05610)	rsaL	-	18045385
qscR (AL347_03130)	qscR	PA1300 (AL347_06610)	PA1300 (hxuI)	?	29729420
qscR (AL347_03130)	qscR	PA1897 (AL347_03135)	PA1897	+	27242034*, 18974177*, 22587778
qscR (AL347_03130)	qscR	phzA2 (AL347_03125)	phzA2	?	27242034*
qscR (AL347_03130)	qscR	rubA1 (AL347_16515)	rubA1	?	27242034*
qscR (AL347_03130)	qscR	lasI (AL347_05605)	lasI	?	27242034*
qscR (AL347_03130)	qscR	phzF2 (AL347_03100)	phzF2	?	27242034*
qscR (AL347_03130)	qscR	phzB1 (AL347_21620)	phzB1	?	27242034*
qscR (AL347_03130)	qscR	phzB2 (AL347_03120)	phzB2	?	27242034*
qscR (AL347_03130)	qscR	hcnA (AL347_01585)	hcnA	?	27242034*
qscR (AL347_03130)	qscR	hcnC (AL347_01575)	hcnC	?	27242034*
qscR (AL347_03130)	qscR	hcnB (AL347_01580)	hcnB	?	27242034*
qscR (AL347_03130)	qscR	phzD1 (AL347_21610)	phzD1	?	27242034*
qscR (AL347_03130)	qscR	rhlI (AL347_25330)	rhlI	?	27242034*
qscR (AL347_03130)	qscR	phzA1 (AL347_21625)	phzA1	?	27242034*
qscR (AL347_03130)	qscR	lasB (AL347_24155)	lasB	?	27242034*
qscR (AL347_03130)	qscR	PA1896 (AL347_03140)	PA1896	?	27242034*
qscR (AL347_03130)	qscR	PA1895 (AL347_03145)	PA1895	?	27242034*
qscR (AL347_03130)	qscR	PA1894 (AL347_03150)	PA1894	?	27242034*
qscR (AL347_03130)	qscR	PA1893 (AL347_03155)	PA1893	?	27242034*
qscR (AL347_03130)	qscR	PA1892 (AL347_03160)	PA1892	?	27242034*
qscR (AL347_03130)	qscR	PA1891 (AL347_03165)	PA1891	?	27242034*
qscR (AL347_03130)	qscR	phzG2 (AL347_03095)	phzG2	?	27242034*
qscR (AL347_03130)	qscR	phzG1 (AL347_21595)	phzG1	?	27242034*
qscR (AL347_03130)	qscR	phzC1 (AL347_21615)	phzC1	?	27242034*
qscR (AL347_03130)	qscR	phzE1 (AL347_21605)	phzE1	?	27242034*
rbsR (AL347_02870)	rbsR	rbsB (AL347_02885)	rbsB	-	27242034*
rbsR (AL347_02870)	rbsR	rbsA (AL347_02880)	rbsA	-	27242034*
rbsR (AL347_02870)	rbsR	rbsC (AL347_02875)	rbsC	-	27242034*
rbsR (AL347_02870)	rbsR	rbsR (AL347_02870)	rbsR	-	27242034*
rbsR (AL347_02870)	rbsR	rbsK (AL347_02865)	rbsK	-	27242034*
recA (AL347_24695)	recA	recA (AL347_24695)	recA	?	27242034*
rhlR (AL347_25325)	rhlR	migA (AL347_10020)	migA	+	11021926

rhlR (AL347_25325)	rhlR	hcnA (AL347_01585)	hcnA	+	11092854
rhlR (AL347_25325)	rhlR	hcnB (AL347_01580)	hcnB	+	11092854
rhlR (AL347_25325)	rhlR	hcnC (AL347_01575)	hcnC	+	11092854
rhlR (AL347_25325)	rhlR	phzC1 (AL347_21615)	phzC1	+	11544214
rhlR (AL347_25325)	rhlR	phzD1 (AL347_21610)	phzD1	+	11544214
rhlR (AL347_25325)	rhlR	rpoS (AL347_24665)	rpoS	-	15073294
rhlR (AL347_25325)	rhlR	exoS (AL347_23535)	exoS	-	15073294
rhlR (AL347_25325)	rhlR	pqsA (AL347_08225)	pqsA	-	16735731
rhlR (AL347_25325)	rhlR	pqsB (AL347_08220)	pqsB	-	16735731
rhlR (AL347_25325)	rhlR	pqsC (AL347_08215)	pqsC	-	16735731
rhlR (AL347_25325)	rhlR	pqsD (AL347_08210)	pqsD	-	16735731
rhlR (AL347_25325)	rhlR	pqsE (AL347_08205)	pqsE	-	16735731
rhlR (AL347_25325)	rhlR	phzE1 (AL347_21605)	phzE1	+	21614486
rhlR (AL347_25325)	rhlR	rmlB (AL347_15530)	rmlB	+	22262098
rhlR (AL347_25325)	rhlR	rmlD (AL347_15535)	rmlD	+	22262098
rhlR (AL347_25325)	rhlR	rmlA (AL347_15540)	rmlA	+	22262098
rhlR (AL347_25325)	rhlR	rmlC (AL347_15545)	rmlC	+	22262098
rhlR (AL347_25325)	rhlR	mvfR (AL347_08190)	mvfR	-	25225275
rhlR (AL347_25325)	rhlR	PA0165 (AL347_18610)	PA0165	?	31270321
rhlR (AL347_25325)	rhlR	PA0883 (AL347_09080)	PA0883	?	31270321
rhlR (AL347_25325)	rhlR	PA1473 (AL347_05400)	PA1473	?	31270321
rhlR (AL347_25325)	rhlR	pchF (AL347_21545)	pchF	?	31270321
rhlR (AL347_25325)	rhlR	desB (AL347_14050)	desB	?	31270321
rhlR (AL347_25325)	rhlR	lecA (AL347_31780)	lecA	+	32622078
rhlR (AL347_25325)	rhlR	lecB (AL347_25945)	lecB	+	32622078
rhlR (AL347_25325)	rhlR	rhlI (AL347_25330)	rhlI	+	32622078
rhlR (AL347_25325)	rhlR	rhlA (AL347_25315)	rhlA	+	32622078
rhlR (AL347_25325)	rhlR	lasB (AL347_24155)	lasB	+	32622078
rhlR (AL347_25325)	rhlR	acp1 (AL347_03275)	acp1	+	32715566
rhlR (AL347_25325)	rhlR	phzG2 (AL347_03095)	phzG2	+	32715566
rhlR (AL347_25325)	rhlR	phzA1 (AL347_21625)	phzA1	+	32715566
rhlR (AL347_25325)	rhlR	phzB1 (AL347_21620)	phzB1	+	32715566
rhlR (AL347_25325)	rhlR	rhlB (AL347_25320)	rhlB	+	32622078, 21719541
rhlR (AL347_25325)	rhlR	rhlC (AL347_07505)	rhlC	+	8051059, 32622078
rhlR (AL347_25325)	rhlR	acpP (AL347_28040)	acpP	+	27242034*, 18974177*, 22587778
rhlR (AL347_25325)	rhlR	PA1869 (AL347_03275)	PA1869	+	27242034*, 18974177*, 22587778
rhlR (AL347_25325)	rhlR	phzF1 (AL347_21600)	phzF1	+	27242034*, 18974177*, 22587778
rhlR (AL347_25325)	rhlR	phzG1 (AL347_21595)	phzG1	+	27242034*, 18974177*, 22587778

rhlR (AL347_25325)	rhlR	rhlG (AL347_25795)	rhlG	?	27242034*, 18974177*, 22587778
rhlR (AL347_25325)	rhlR	rhlR (AL347_25325)	rhlR	-	27242034*, 18974177*, 22587778
rhlR (AL347_25325)	rhlR	xcpW (AL347_27350)	xcpW	?	27242034*
rhlR (AL347_25325)	rhlR	xcpV (AL347_27345)	xcpV	?	27242034*
rhlR (AL347_25325)	rhlR	xcpU (AL347_27340)	xcpU	?	27242034*
rhlR (AL347_25325)	rhlR	xcpT (AL347_27335)	xcpT	?	27242034*
rhlR (AL347_25325)	rhlR	xcpS (AL347_27330)	xcpS	?	27242034*
rhlR (AL347_25325)	rhlR	xcpR (AL347_27325)	xcpR	?	27242034*
rhlR (AL347_25325)	rhlR	xcpQ (AL347_27315)	xcpQ	?	27242034*
rhlR (AL347_25325)	rhlR	phzF2 (AL347_03100)	phzF2	?	27242034*
rhlR (AL347_25325)	rhlR	xcpZ (AL347_27365)	xcpZ	?	27242034*
rhlR (AL347_25325)	rhlR	xcpX (AL347_27355)	xcpX	?	27242034*
rhlR (AL347_25325)	rhlR	chiC (AL347_33525)	chiC	?	27242034*
rhlR (AL347_25325)	rhlR	PA0179 (AL347_18680)	PA0179	?	27242034*
rhlR (AL347_25325)	rhlR	lasA (AL347_03265)	lasA	?	27242034*
rhlR (AL347_25325)	rhlR	lasI (AL347_05605)	lasI	?	27242034*
rhlR (AL347_25325)	rhlR	xcpP (AL347_27320)	xcpP	?	27242034*
rocA1 (AL347_22975)	rocA1	cupB1 (AL347_22275)	cupB1	+	11381121
rocA1 (AL347_22975)	rocA1	cupC2 (AL347_08240)	cupC2	-	15659157
rocA1 (AL347_22975)	rocA1	cupC3 (AL347_08235)	cupC3	-	15659157
rocA1 (AL347_22975)	rocA1	cupB6 (AL347_22300)	cupB6	+	15659157
rocA1 (AL347_22975)	rocA1	cupB5 (AL347_22295)	cupB5	+	15659157
rocA1 (AL347_22975)	rocA1	cupB4 (AL347_22290)	cupB4	+	15659157
rocA1 (AL347_22975)	rocA1	cupB3 (AL347_22285)	cupB3	+	15659157
rocA1 (AL347_22975)	rocA1	cupB2 (AL347_22280)	cupB2	+	15659157
rocA1 (AL347_22975)	rocA1	cupC1 (AL347_08245)	cupC1	-	27242034*, 18974177*, 22587778
rocA1 (AL347_22975)	rocA1	rocR (AL347_22980)	rocR	?	27242034*
rocA1 (AL347_22975)	rocA1	rocS1 (AL347_22985)	rocS1	?	27242034*
rocA1 (AL347_22975)	rocA1	rocA1 (AL347_22975)	rocA1	?	27242034*
roxR (AL347_11330)	roxR	cyoA (AL347_06520)	cyoA	+	19930444
roxR (AL347_11330)	roxR	cyoB (AL347_06515)	cyoB	+	19930444
roxR (AL347_11330)	roxR	cyoC (AL347_06510)	cyoC	+	19930444
roxR (AL347_11330)	roxR	cyoD (AL347_06505)	cyoD	+	19930444
roxR (AL347_11330)	roxR	cyoE (AL347_06500)	cyoE	+	19930444
roxR (AL347_11330)	roxR	ccoP1 (AL347_04985)	ccoP1	+	19930444
roxR (AL347_11330)	roxR	ccoQ1 (AL347_04980)	ccoQ1	+	19930444
roxR (AL347_11330)	roxR	ccoO1 (AL347_04975)	ccoO1	+	19930444
roxR (AL347_11330)	roxR	ccoN1 (AL347_04970)	ccoN1	+	19930444

roxR (AL347_11330)	roxR	ccoP2 (AL347_04965)	ccoP2	+	19930444
roxR (AL347_11330)	roxR	ccoQ2 (AL347_04960)	ccoQ2	+	19930444
roxR (AL347_11330)	roxR	ccoO2 (AL347_04955)	ccoO2	+	19930444
roxR (AL347_11330)	roxR	ccoN2 (AL347_04950)	ccoN2	+	19930444
roxR (AL347_11330)	roxR	cioB (AL347_23070)	cioB	+	19930444
roxR (AL347_11330)	roxR	cioA (AL347_23065)	cioA	+	19930444
roxR (AL347_11330)	roxR	coxB (AL347_18300)	coxB	-	19930444, 21833336
roxR (AL347_11330)	roxR	coxA (AL347_18305)	coxA	-	19930444, 21833336
roxR (AL347_11330)	roxR	PA0107 (AL347_18310)	PA0107	-	19930444, 21833336
roxR (AL347_11330)	roxR	coIII (AL347_18315)	coIII	-	19930444, 21833336
roxR (AL347_11330)	roxR	roxR (AL347_11330)	roxR	+	27242034*, 18974177*, 22587778
roxS (AL347_11335)	roxS	cyoA (AL347_06520)	cyoA	+	19930444
roxS (AL347_11335)	roxS	cyoB (AL347_06515)	cyoB	+	19930444
roxS (AL347_11335)	roxS	cyoC (AL347_06510)	cyoC	+	19930444
roxS (AL347_11335)	roxS	cyoD (AL347_06505)	cyoD	+	19930444
roxS (AL347_11335)	roxS	cyoE (AL347_06500)	cyoE	+	19930444
roxS (AL347_11335)	roxS	ccoP1 (AL347_04985)	ccoP1	+	19930444
roxS (AL347_11335)	roxS	ccoQ1 (AL347_04980)	ccoQ1	+	19930444
roxS (AL347_11335)	roxS	ccoO1 (AL347_04975)	ccoO1	+	19930444
roxS (AL347_11335)	roxS	ccoN1 (AL347_04970)	ccoN1	+	19930444
roxS (AL347_11335)	roxS	ccoP2 (AL347_04965)	ccoP2	+	19930444
roxS (AL347_11335)	roxS	ccoQ2 (AL347_04960)	ccoQ2	+	19930444
roxS (AL347_11335)	roxS	ccoO2 (AL347_04955)	ccoO2	+	19930444
roxS (AL347_11335)	roxS	ccoN2 (AL347_04950)	ccoN2	+	19930444
roxS (AL347_11335)	roxS	cioB (AL347_23070)	cioB	+	19930444
roxS (AL347_11335)	roxS	cioA (AL347_23065)	cioA	+	19930444
roxS (AL347_11335)	roxS	coxB (AL347_18300)	coxB	-	19930444, 21833336
roxS (AL347_11335)	roxS	coxA (AL347_18305)	coxA	-	19930444, 21833336
roxS (AL347_11335)	roxS	PA0107 (AL347_18310)	PA0107	-	19930444, 21833336
roxS (AL347_11335)	roxS	coIII (AL347_18315)	coIII	-	19930444, 21833336
rpoD (AL347_20760)	rpoD	tonB1 (AL347_17475)	tonB1	+	Homology from 25780925
rpoD (AL347_20760)	rpoD	PA5525 (AL347_17445)	PA5525	+	Homology from 25780925
rpoD (AL347_20760)	rpoD	PA5524 (AL347_17440)	PA5524	+	Homology from 25780925
rpoD (AL347_20760)	rpoD	PA5523 (AL347_17435)	PA5523	+	Homology from 25780925
rpoD (AL347_20760)	rpoD	PA5522 (AL347_17430)	PA5522 (pauA6)	+	Homology from 25780925
rpoD (AL347_20760)	rpoD	PA5477 (AL347_17205)	PA5477	+	Homology from 25780925
rpoD (AL347_20760)	rpoD	PA5473 (AL347_17180)	PA5473	+	Homology from 25780925
rpoD (AL347_20760)	rpoD	PA5429 (AL347_16940)	PA5429 (aspA)	+	Homology from 25780925

rpoD (AL347_20760)	rpoD	PA5428 (AL347_16935)	PA5428	+	Homology from 25780925
rpoD (AL347_20760)	rpoD	PA5421 (AL347_16895)	PA5421 (fdhA)	+	Homology from 25780925
rpoD (AL347_20760)	rpoD	PA5383 (AL347_16705)	PA5383	+	Homology from 25780925
rpoD (AL347_20760)	rpoD	PA5382 (AL347_16700)	PA5382	+	Homology from 25780925
rpoD (AL347_20760)	rpoD	BetB (AL347_16650)	BetB	+	Homology from 25780925
rpoD (AL347_20760)	rpoD	PA5331 (AL347_16410)	PA5331 (pyrE)	+	Homology from 25780925
rpoD (AL347_20760)	rpoD	PA5314 (AL347_16325)	PA5314	+	Homology from 25780925
rpoD (AL347_20760)	rpoD	PA5313 (AL347_16320)	PA5313 (gabT2, paaT)	+	Homology from 25780925
rpoD (AL347_20760)	rpoD	PA5312 (AL347_16315)	PA5312 (pauC, kauB)	+	Homology from 25780925
rpoD (AL347_20760)	rpoD	PA5307 (AL347_16290)	PA5307	+	Homology from 25780925
rpoD (AL347_20760)	rpoD	dadA (AL347_16275)	dadA	+	Homology from 25780925
rpoD (AL347_20760)	rpoD	PA5303 (AL347_16270)	PA5303	+	Homology from 25780925
rpoD (AL347_20760)	rpoD	PA5301 (AL347_16260)	PA5301 (pauR, ycjC)	+	Homology from 25780925
rpoD (AL347_20760)	rpoD	PA5290 (AL347_16205)	PA5290	+	Homology from 25780925
rpoD (AL347_20760)	rpoD	PA5289 (AL347_16200)	PA5289	+	Homology from 25780925
rpoD (AL347_20760)	rpoD	PA5281 (AL347_16155)	PA5281	+	Homology from 25780925
rpoD (AL347_20760)	rpoD	PA5280 (AL347_16150)	PA5280 (xerC)	+	Homology from 25780925
rpoD (AL347_20760)	rpoD	PA5279 (AL347_16145)	PA5279	+	Homology from 25780925
rpoD (AL347_20760)	rpoD	PA5278 (AL347_16140)	PA5278 (dapF)	+	Homology from 25780925
rpoD (AL347_20760)	rpoD	PA5277 (AL347_16135)	PA5277 (lysA)	+	Homology from 25780925
rpoD (AL347_20760)	rpoD	PA5276 (AL347_16130)	PA5276 (lppL)	+	Homology from 25780925
rpoD (AL347_20760)	rpoD	PA5275 (AL347_16125)	PA5275	+	Homology from 25780925
rpoD (AL347_20760)	rpoD	algQ (AL347_16020)	algQ	+	Homology from 25780925
rpoD (AL347_20760)	rpoD	PA5254 (AL347_16015)	PA5254	+	Homology from 25780925
rpoD (AL347_20760)	rpoD	PA5244 (AL347_16015)	PA5244	+	Homology from 25780925
rpoD (AL347_20760)	rpoD	PA5243 (AL347_15955)	PA5243 (hemB)	+	Homology from 25780925
rpoD (AL347_20760)	rpoD	ppK (AL347_15950)	ppK	+	Homology from 25780925
rpoD (AL347_20760)	rpoD	PA5241 (AL347_15945)	PA5241 (ppx, gppA)	+	Homology from 25780925
rpoD (AL347_20760)	rpoD	PA5240 (AL347_15940)	PA5240 (trxA)	+	Homology from 25780925
rpoD (AL347_20760)	rpoD	PA5238 (AL347_15930)	PA5238	+	Homology from 25780925
rpoD (AL347_20760)	rpoD	PA5237 (AL347_15925)	PA5237	+	Homology from 25780925
rpoD (AL347_20760)	rpoD	PA5236 (AL347_15920)	PA5236	+	Homology from 25780925
rpoD (AL347_20760)	rpoD	PA5228 (AL347_15880)	PA5228	+	Homology from 25780925
rpoD (AL347_20760)	rpoD	PA5227 (AL347_15870)	PA5227	+	Homology from 25780925
rpoD (AL347_20760)	rpoD	PA5226 (AL347_15865)	PA5226	+	Homology from 25780925
rpoD (AL347_20760)	rpoD	PA5225 (AL347_15860)	PA5225	+	Homology from 25780925
rpoD (AL347_20760)	rpoD	PA5224 (AL347_15855)	PA5224 (pepP)	+	Homology from 25780925
rpoD (AL347_20760)	rpoD	PA5223 (AL347_15850)	PA5223 (ubiH, visB)	+	Homology from 25780925

rpoD (AL347_20760)	rpoD	PA5222 (AL347_15845)	PA5222	+	Homology from 25780925
rpoD (AL347_20760)	rpoD	PA5221 (AL347_15840)	PA5221	+	Homology from 25780925
rpoD (AL347_20760)	rpoD	PA5220 (AL347_15835)	PA5220	+	Homology from 25780925
rpoD (AL347_20760)	rpoD	PA5219 (AL347_15830)	PA5219	+	Homology from 25780925
rpoD (AL347_20760)	rpoD	PA5205 (AL347_15760)	PA5205	+	Homology from 25780925
rpoD (AL347_20760)	rpoD	PA5195 (AL347_15710)	PA5195	+	Homology from 25780925
rpoD (AL347_20760)	rpoD	PA5190 (AL347_15680)	PA5190	+	Homology from 25780925
rpoD (AL347_20760)	rpoD	rmlC (AL347_15545)	rmlC	+	Homology from 25780925
rpoD (AL347_20760)	rpoD	rmlA (AL347_15540)	rmlA	+	Homology from 25780925
rpoD (AL347_20760)	rpoD	rmlD (AL347_15535)	rmlD	+	Homology from 25780925
rpoD (AL347_20760)	rpoD	rmlB (AL347_15530)	rmlB	+	Homology from 25780925
rpoD (AL347_20760)	rpoD	PA5130 (AL347_15305)	PA5130	+	Homology from 25780925
rpoD (AL347_20760)	rpoD	grx (AL347_15300)	grx	+	Homology from 25780925
rpoD (AL347_20760)	rpoD	PA5128 (AL347_15295)	PA5128 (secB)	+	Homology from 25780925
rpoD (AL347_20760)	rpoD	PA5115 (AL347_15220)	PA5115	+	Homology from 25780925
rpoD (AL347_20760)	rpoD	PA5080 (AL347_15050)	PA5080	+	Homology from 25780925
rpoD (AL347_20760)	rpoD	PA5079 (AL347_15045)	PA5079	+	Homology from 25780925
rpoD (AL347_20760)	rpoD	PA5071 (AL347_15005)	PA5071	+	Homology from 25780925
rpoD (AL347_20760)	rpoD	PA5070 (AL347_15000)	PA5070 (tatC, mttB, yigU)	+	Homology from 25780925
rpoD (AL347_20760)	rpoD	PA5069 (AL347_14995)	PA5069 (tatB, mttA, yigT)	+	Homology from 25780925
rpoD (AL347_20760)	rpoD	PA5062 (AL347_14960)	PA5062	+	Homology from 25780925
rpoD (AL347_20760)	rpoD	PA5029 (AL347_14790)	PA5029	+	Homology from 25780925
rpoD (AL347_20760)	rpoD	PA5023 (AL347_14760)	PA5023	+	Homology from 25780925
rpoD (AL347_20760)	rpoD	PA5022 (AL347_14755)	PA5022	+	Homology from 25780925
rpoD (AL347_20760)	rpoD	PA5021 (AL347_14750)	PA5021	+	Homology from 25780925
rpoD (AL347_20760)	rpoD	PA5019 (AL347_14735)	PA5019	+	Homology from 25780925
rpoD (AL347_20760)	rpoD	aceE (AL347_14705)	aceE	+	Homology from 25780925
rpoD (AL347_20760)	rpoD	PA5014 (AL347_14700)	PA5014 (glnE)	+	Homology from 25780925
rpoD (AL347_20760)	rpoD	PA5000 (AL347_14630)	PA5000 (wapR)	+	Homology from 25780925
rpoD (AL347_20760)	rpoD	PA4985 (AL347_14555)	PA4985	+	Homology from 25780925
rpoD (AL347_20760)	rpoD	PA4963 (AL347_14440)	PA4963	+	Homology from 25780925
rpoD (AL347_20760)	rpoD	PA4959 (AL347_14420)	PA4959	+	Homology from 25780925
rpoD (AL347_20760)	rpoD	PA4958 (AL347_14415)	PA4958 (fimW)	+	Homology from 25780925
rpoD (AL347_20760)	rpoD	PA4957 (AL347_14405)	PA4957 (psd)	+	Homology from 25780925
rpoD (AL347_20760)	rpoD	PA4956 (AL347_14400)	PA4956 (rhdA)	+	Homology from 25780925
rpoD (AL347_20760)	rpoD	PA4952 (AL347_14380)	PA4952	+	Homology from 25780925
rpoD (AL347_20760)	rpoD	PA4949 (AL347_14365)	PA4949	+	Homology from 25780925
rpoD (AL347_20760)	rpoD	PA4948 (AL347_14360)	PA4948	+	Homology from 25780925

rpoD (AL347_20760)	rpoD	PA4947 (AL347_14355)	PA4947 (amiB)	+	Homology from 25780925
rpoD (AL347_20760)	rpoD	PA4946 (AL347_14350)	PA4946 (mutL)	+	Homology from 25780925
rpoD (AL347_20760)	rpoD	PA4945 (AL347_14345)	PA4945 (miaA)	+	Homology from 25780925
rpoD (AL347_20760)	rpoD	hfq (AL347_14340)	hfq	+	Homology from 25780925
rpoD (AL347_20760)	rpoD	PA4943 (AL347_14335)	PA4943	+	Homology from 25780925
rpoD (AL347_20760)	rpoD	PA4928 (AL347_14250)	PA4928	+	Homology from 25780925
rpoD (AL347_20760)	rpoD	PA4913 (AL347_14175)	PA4913	+	Homology from 25780925
rpoD (AL347_20760)	rpoD	PA4903 (AL347_14125)	PA4903	+	Homology from 25780925
rpoD (AL347_20760)	rpoD	PA4902 (AL347_14120)	PA4902	+	Homology from 25780925
rpoD (AL347_20760)	rpoD	PA4900 (AL347_14110)	PA4900	+	Homology from 25780925
rpoD (AL347_20760)	rpoD	PA4899 (AL347_14105)	PA4899	+	Homology from 25780925
rpoD (AL347_20760)	rpoD	PA4838 (AL347_13795)	PA4838	+	Homology from 25780925
rpoD (AL347_20760)	rpoD	PA4837 (AL347_13790)	PA4837 (cntO, zrmA)	+	Homology from 25780925
rpoD (AL347_20760)	rpoD	PA4836 (AL347_13785)	PA4836 (cntL, zrmB)	+	Homology from 25780925
rpoD (AL347_20760)	rpoD	PA4835 (AL347_13780)	PA4835 (cntM, zrmC)	+	Homology from 25780925
rpoD (AL347_20760)	rpoD	PA4834 (AL347_13775)	PA4834 (cntI, zrmD)	+	Homology from 25780925
rpoD (AL347_20760)	rpoD	PA4833 (AL347_13770)	PA4833	+	Homology from 25780925
rpoD (AL347_20760)	rpoD	PA4832 (AL347_13765)	PA4832	+	Homology from 25780925
rpoD (AL347_20760)	rpoD	PA4828 (AL347_13745)	PA4828	+	Homology from 25780925
rpoD (AL347_20760)	rpoD	PA4824 (AL347_13725)	PA4824	+	Homology from 25780925
rpoD (AL347_20760)	rpoD	PA4823 (AL347_13720)	PA4823	+	Homology from 25780925
rpoD (AL347_20760)	rpoD	PA4822 (AL347_13715)	PA4822	+	Homology from 25780925
rpoD (AL347_20760)	rpoD	PA4821 (AL347_13705)	PA4821	+	Homology from 25780925
rpoD (AL347_20760)	rpoD	PA4806 (AL347_13630)	PA4806	+	Homology from 25780925
rpoD (AL347_20760)	rpoD	PA4765 (AL347_13420)	PA4765 (omlA, oprX)	+	Homology from 25780925
rpoD (AL347_20760)	rpoD	fur (AL347_13415)	fur	+	Homology from 25780925
rpoD (AL347_20760)	rpoD	carA (AL347_13380)	carA	+	Homology from 25780925
rpoD (AL347_20760)	rpoD	PA4757 (AL347_13375)	PA4757	+	Homology from 25780925
rpoD (AL347_20760)	rpoD	carB (AL347_13370)	carB	+	Homology from 25780925
rpoD (AL347_20760)	rpoD	PA4747 (AL347_13325)	PA4747 (secG)	+	Homology from 25780925
rpoD (AL347_20760)	rpoD	PA4741 (AL347_13285)	PA4741 (rpsO)	+	Homology from 25780925
rpoD (AL347_20760)	rpoD	PA4734 (AL347_13245)	PA4734	+	Homology from 25780925
rpoD (AL347_20760)	rpoD	PA4714 (AL347_13140)	PA4714	+	Homology from 25780925
rpoD (AL347_20760)	rpoD	PA4697 (AL347_13055)	PA4697	+	Homology from 25780925
rpoD (AL347_20760)	rpoD	PA4696 (AL347_13050)	PA4696 (ilvI)	+	Homology from 25780925
rpoD (AL347_20760)	rpoD	PA4695 (AL347_13045)	PA4695 (ilvH)	+	Homology from 25780925
rpoD (AL347_20760)	rpoD	PA4694 (AL347_13040)	PA4694 (ilvC)	+	Homology from 25780925
rpoD (AL347_20760)	rpoD	PA4693 (AL347_13035)	PA4693 (pssA)	+	Homology from 25780925

rpoD (AL347_20760)	rpoD	chtA (AL347_12915)	chtA	+	Homology from 25780925
rpoD (AL347_20760)	rpoD	PA4669 (AL347_12865)	PA4669 (ipk, ychB)	+	Homology from 25780925
rpoD (AL347_20760)	rpoD	PA4668 (AL347_12860)	PA4668 (lolB)	+	Homology from 25780925
rpoD (AL347_20760)	rpoD	PA4667 (AL347_12855)	PA4667 (lbcA)	+	Homology from 25780925
rpoD (AL347_20760)	rpoD	hemA (AL347_12850)	hemA	+	Homology from 25780925
rpoD (AL347_20760)	rpoD	prfA (AL347_12845)	prfA	+	Homology from 25780925
rpoD (AL347_20760)	rpoD	hemK (AL347_12840)	hemK	+	Homology from 25780925
rpoD (AL347_20760)	rpoD	moeB (AL347_12835)	moeB	+	Homology from 25780925
rpoD (AL347_20760)	rpoD	murI (AL347_12830)	murI	+	Homology from 25780925
rpoD (AL347_20760)	rpoD	PA4632 (AL347_12670)	PA4632	+	Homology from 25780925
rpoD (AL347_20760)	rpoD	PA4594 (AL347_12460)	PA4594	+	Homology from 25780925
rpoD (AL347_20760)	rpoD	PA4593 (AL347_12455)	PA4593	+	Homology from 25780925
rpoD (AL347_20760)	rpoD	PA4592 (AL347_12450)	PA4592	+	Homology from 25780925
rpoD (AL347_20760)	rpoD	PA4591 (AL347_12445)	PA4591	+	Homology from 25780925
rpoD (AL347_20760)	rpoD	PA4590 (AL347_12440)	PA4590 (pra)	+	Homology from 25780925
rpoD (AL347_20760)	rpoD	PA4581 (AL347_12390)	PA4581 (rtcR)	+	Homology from 25780925
rpoD (AL347_20760)	rpoD	PA4569 (AL347_12330)	PA4569 (ispB, cel)	+	Homology from 25780925
rpoD (AL347_20760)	rpoD	PA4567 (AL347_12320)	PA4567 (rpmA)	+	Homology from 25780925
rpoD (AL347_20760)	rpoD	PA4566 (AL347_12315)	PA4566 (obg)	+	Homology from 25780925
rpoD (AL347_20760)	rpoD	PA4565 (AL347_12310)	PA4565 (proB)	+	Homology from 25780925
rpoD (AL347_20760)	rpoD	PA4564 (AL347_12305)	PA4564	+	Homology from 25780925
rpoD (AL347_20760)	rpoD	PA4545 (AL347_12205)	PA4545 (comL, ypiY)	+	Homology from 25780925
rpoD (AL347_20760)	rpoD	PA4540 (AL347_11575)	PA4540	+	Homology from 25780925
rpoD (AL347_20760)	rpoD	PA4524 (AL347_11490)	PA4524 (nadC)	+	Homology from 25780925
rpoD (AL347_20760)	rpoD	PA4523 (AL347_11485)	PA4523	+	Homology from 25780925
rpoD (AL347_20760)	rpoD	PA4512 (AL347_11430)	PA4512 (lpxO1)	+	Homology from 25780925
rpoD (AL347_20760)	rpoD	dppA3 (AL347_11365)	dppA3	+	Homology from 25780925
rpoD (AL347_20760)	rpoD	psdR (AL347_11360)	psdR	+	Homology from 25780925
rpoD (AL347_20760)	rpoD	mdpA (AL347_11355)	mdpA	+	Homology from 25780925
rpoD (AL347_20760)	rpoD	PA4473 (AL347_11230)	PA4473	+	Homology from 25780925
rpoD (AL347_20760)	rpoD	PA4466 (AL347_11195)	PA4466	+	Homology from 25780925
rpoD (AL347_20760)	rpoD	PA4465 (AL347_11190)	PA4465	+	Homology from 25780925
rpoD (AL347_20760)	rpoD	PA4464 (AL347_11185)	PA4464 (ptsN)	+	Homology from 25780925
rpoD (AL347_20760)	rpoD	PA4463 (AL347_11180)	PA4463	+	Homology from 25780925
rpoD (AL347_20760)	rpoD	PA4459 (AL347_11160)	PA4459 (lptC, yrbK)	+	Homology from 25780925
rpoD (AL347_20760)	rpoD	PA4458 (AL347_11155)	PA4458	+	Homology from 25780925
rpoD (AL347_20760)	rpoD	PA4457 (AL347_11150)	PA4457	+	Homology from 25780925
rpoD (AL347_20760)	rpoD	PA4437 (AL347_11050)	PA4437	+	Homology from 25780925

rpoD (AL347_20760)	rpoD	PA4434 (AL347_11035)	PA4434	+	Homology from 25780925
rpoD (AL347_20760)	rpoD	PA4407 (AL347_10895)	PA4407 (ftsZ)	+	Homology from 25780925
rpoD (AL347_20760)	rpoD	PA4402 (AL347_10865)	PA4402 (argJ)	+	Homology from 25780925
rpoD (AL347_20760)	rpoD	PA4388 (AL347_10795)	PA4388	+	Homology from 25780925
rpoD (AL347_20760)	rpoD	PA4375 (AL347_10725)	PA4375 (mexW)	+	Homology from 25780925
rpoD (AL347_20760)	rpoD	PA4374 (AL347_10720)	PA4374 (mexV)	+	Homology from 25780925
rpoD (AL347_20760)	rpoD	PA4369 (AL347_10695)	PA4369	+	Homology from 25780925
rpoD (AL347_20760)	rpoD	PA4368 (AL347_10690)	PA4368	+	Homology from 25780925
rpoD (AL347_20760)	rpoD	bifA (AL347_10685)	bifA	+	Homology from 25780925
rpoD (AL347_20760)	rpoD	PA4351 (AL347_10600)	PA4351 (olsA)	+	Homology from 25780925
rpoD (AL347_20760)	rpoD	PA4350 (AL347_10595)	PA4350 (olsB)	+	Homology from 25780925
rpoD (AL347_20760)	rpoD	PA4323 (AL347_10460)	PA4323	+	Homology from 25780925
rpoD (AL347_20760)	rpoD	PA4322 (AL347_10455)	PA4322	+	Homology from 25780925
rpoD (AL347_20760)	rpoD	PA4321 (AL347_10450)	PA4321	+	Homology from 25780925
rpoD (AL347_20760)	rpoD	PA4320 (AL347_10445)	PA4320	+	Homology from 25780925
rpoD (AL347_20760)	rpoD	PA4319 (AL347_10440)	PA4319	+	Homology from 25780925
rpoD (AL347_20760)	rpoD	PA4318 (AL347_10435)	PA4318	+	Homology from 25780925
rpoD (AL347_20760)	rpoD	PA4317 (AL347_10430)	PA4317	+	Homology from 25780925
rpoD (AL347_20760)	rpoD	mvaT (AL347_10420)	mvaT	+	Homology from 25780925
rpoD (AL347_20760)	rpoD	PA4314 (AL347_10415)	PA4314 (purU1)	+	Homology from 25780925
rpoD (AL347_20760)	rpoD	PA4291 (AL347_10290)	PA4291	+	Homology from 25780925
rpoD (AL347_20760)	rpoD	pchA (AL347_21515)	pchA	+	Homology from 25780925
rpoD (AL347_20760)	rpoD	pchB (AL347_21520)	pchB	+	Homology from 25780925
rpoD (AL347_20760)	rpoD	pchC (AL347_21525)	pchC	+	Homology from 25780925
rpoD (AL347_20760)	rpoD	pchD (AL347_21530)	pchD	+	Homology from 25780925
rpoD (AL347_20760)	rpoD	ppgL (AL347_21660)	ppgL	+	Homology from 25780925
rpoD (AL347_20760)	rpoD	PA4203 (AL347_21665)	PA4203 (nmoR)	+	Homology from 25780925
rpoD (AL347_20760)	rpoD	nmoA (AL347_21670)	nmoA	+	Homology from 25780925
rpoD (AL347_20760)	rpoD	PA4201 (AL347_21675)	PA4201 (ddlA)	+	Homology from 25780925
rpoD (AL347_20760)	rpoD	bfiS (AL347_21695)	bfiS	+	Homology from 25780925
rpoD (AL347_20760)	rpoD	bfiR (AL347_21700)	bfiR	+	Homology from 25780925
rpoD (AL347_20760)	rpoD	PA4185 (AL347_21755)	PA4185	+	Homology from 25780925
rpoD (AL347_20760)	rpoD	prpL (AL347_21810)	prpL	+	Homology from 25780925
rpoD (AL347_20760)	rpoD	PA4167 (AL347_21850)	PA4167	+	Homology from 25780925
rpoD (AL347_20760)	rpoD	PA4161 (AL347_21885)	PA4161 (fepG)	+	Homology from 25780925
rpoD (AL347_20760)	rpoD	PA4160 (AL347_21890)	PA4160 (fepD)	+	Homology from 25780925
rpoD (AL347_20760)	rpoD	PA4159 (AL347_21895)	PA4159 (fepB)	+	Homology from 25780925
rpoD (AL347_20760)	rpoD	PA4158 (AL347_21900)	PA4158 (fepC)	+	Homology from 25780925

rpoD (AL347_20760)	rpoD	PA4137 (AL347_22010)	PA4137	+	Homology from 25780925
rpoD (AL347_20760)	rpoD	PA4135 (AL347_22020)	PA4135	+	Homology from 25780925
rpoD (AL347_20760)	rpoD	PA4123 (AL347_22080)	PA4123 (hpcC, hpaE)	+	Homology from 25780925
rpoD (AL347_20760)	rpoD	PA4122 (AL347_22085)	PA4122	+	Homology from 25780925
rpoD (AL347_20760)	rpoD	PA4121 (AL347_22090)	PA4121	+	Homology from 25780925
rpoD (AL347_20760)	rpoD	PA4115 (AL347_22120)	PA4115	+	Homology from 25780925
rpoD (AL347_20760)	rpoD	PA4114 (AL347_22125)	PA4114	+	Homology from 25780925
rpoD (AL347_20760)	rpoD	PA4113 (AL347_22130)	PA4113	+	Homology from 25780925
rpoD (AL347_20760)	rpoD	PA4089 (AL347_22260)	PA4089	+	Homology from 25780925
rpoD (AL347_20760)	rpoD	PA4088 (AL347_22265)	PA4088	+	Homology from 25780925
rpoD (AL347_20760)	rpoD	PA4087 (AL347_22270)	PA4087	+	Homology from 25780925
rpoD (AL347_20760)	rpoD	cupB3 (AL347_22285)	cupB3	+	Homology from 25780925
rpoD (AL347_20760)	rpoD	cupB4 (AL347_22290)	cupB4	+	Homology from 25780925
rpoD (AL347_20760)	rpoD	PA4077 (AL347_22320)	PA4077	+	Homology from 25780925
rpoD (AL347_20760)	rpoD	PA4076 (AL347_22325)	PA4076	+	Homology from 25780925
rpoD (AL347_20760)	rpoD	PA4040 (AL347_22510)	PA4040	+	Homology from 25780925
rpoD (AL347_20760)	rpoD	PA4039 (AL347_22515)	PA4039	+	Homology from 25780925
rpoD (AL347_20760)	rpoD	PA4038 (AL347_22520)	PA4038	+	Homology from 25780925
rpoD (AL347_20760)	rpoD	PA4037 (AL347_22525)	PA4037	+	Homology from 25780925
rpoD (AL347_20760)	rpoD	PA4021 (AL347_22605)	PA4021 (eatR)	+	Homology from 25780925
rpoD (AL347_20760)	rpoD	PA4011 (AL347_22655)	PA4011	+	Homology from 25780925
rpoD (AL347_20760)	rpoD	PA4010 (AL347_22660)	PA4010	+	Homology from 25780925
rpoD (AL347_20760)	rpoD	PA4009 (AL347_22665)	PA4009	+	Homology from 25780925
rpoD (AL347_20760)	rpoD	PA3995 (AL347_22735)	PA3995	+	Homology from 25780925
rpoD (AL347_20760)	rpoD	PA3973 (AL347_22840)	PA3973	+	Homology from 25780925
rpoD (AL347_20760)	rpoD	PA3972 (AL347_22845)	PA3972	+	Homology from 25780925
rpoD (AL347_20760)	rpoD	PA3971 (AL347_22850)	PA3971	+	Homology from 25780925
rpoD (AL347_20760)	rpoD	PA3970 (AL347_22855)	PA3970 (amn)	+	Homology from 25780925
rpoD (AL347_20760)	rpoD	PA3966 (AL347_22880)	PA3966	+	Homology from 25780925
rpoD (AL347_20760)	rpoD	PA3965 (AL347_22885)	PA3965	+	Homology from 25780925
rpoD (AL347_20760)	rpoD	PA3939 (AL347_23020)	PA3939	+	Homology from 25780925
rpoD (AL347_20760)	rpoD	PA3933 (AL347_23050)	PA3933 (betT3)	+	Homology from 25780925
rpoD (AL347_20760)	rpoD	PA3920 (AL347_23120)	PA3920 (copA1, yvgX, cueA)	+	Homology from 25780925
rpoD (AL347_20760)	rpoD	PA3919 (AL347_23130)	PA3919	+	Homology from 25780925
rpoD (AL347_20760)	rpoD	PA3892 (AL347_23270)	PA3892	+	Homology from 25780925
rpoD (AL347_20760)	rpoD	PA3887 (AL347_23300)	PA3887 (nhaP)	+	Homology from 25780925
rpoD (AL347_20760)	rpoD	PA3880 (AL347_23340)	PA3880	+	Homology from 25780925
rpoD (AL347_20760)	rpoD	PA3842 (AL347_23525)	PA3842 (spcS, orf1)	+	Homology from 25780925

rpoD (AL347_20760)	rpoD	PA3835 (AL347_23570)	PA3835	+	Homology from 25780925
rpoD (AL347_20760)	rpoD	PA3835 (AL347_23570)	PA3828 (yjgP)	+	Homology from 25780925
rpoD (AL347_20760)	rpoD	PA3827 (AL347_23615)	PA3827 (lptG, yjgQ)	+	Homology from 25780925
rpoD (AL347_20760)	rpoD	PA3826 (AL347_23620)	PA3826	+	Homology from 25780925
rpoD (AL347_20760)	rpoD	PA3821 (AL347_23650)	PA3821 (secD)	+	Homology from 25780925
rpoD (AL347_20760)	rpoD	PA3820 (AL347_23655)	PA3820 (secF)	+	Homology from 25780925
rpoD (AL347_20760)	rpoD	iscR (AL347_23680)	iscR	+	Homology from 25780925
rpoD (AL347_20760)	rpoD	iscS (AL347_23685)	iscS	+	Homology from 25780925
rpoD (AL347_20760)	rpoD	iscU (AL347_23690)	iscU	+	Homology from 25780925
rpoD (AL347_20760)	rpoD	iscA (AL347_23695)	iscA	+	Homology from 25780925
rpoD (AL347_20760)	rpoD	hscB (AL347_23700)	hscB	+	Homology from 25780925
rpoD (AL347_20760)	rpoD	hscA (AL347_23705)	hscA	+	Homology from 25780925
rpoD (AL347_20760)	rpoD	PA3771 (AL347_23905)	PA3771	+	Homology from 25780925
rpoD (AL347_20760)	rpoD	PA3748 (AL347_24030)	PA3748	+	Homology from 25780925
rpoD (AL347_20760)	rpoD	PA3747 (AL347_24035)	PA3747	+	Homology from 25780925
rpoD (AL347_20760)	rpoD	PA3746 (AL347_24040)	PA3746 (ffh)	+	Homology from 25780925
rpoD (AL347_20760)	rpoD	PA3730 (AL347_24125)	PA3730	+	Homology from 25780925
rpoD (AL347_20760)	rpoD	PA3729 (AL347_24130)	PA3729	+	Homology from 25780925
rpoD (AL347_20760)	rpoD	lasB (AL347_24155)	lasB	+	Homology from 25780925
rpoD (AL347_20760)	rpoD	PA3717 (AL347_24190)	PA3717	+	Homology from 25780925
rpoD (AL347_20760)	rpoD	PA3711 (AL347_24220)	PA3711	+	Homology from 25780925
rpoD (AL347_20760)	rpoD	PA3687 (AL347_24340)	PA3687 (ppc)	+	Homology from 25780925
rpoD (AL347_20760)	rpoD	PA3678 (AL347_24385)	PA3678 (mexL)	+	Homology from 25780925
rpoD (AL347_20760)	rpoD	PA3677 (AL347_24390)	PA3677 (mexJ)	+	Homology from 25780925
rpoD (AL347_20760)	rpoD	mexK (AL347_24395)	mexK	+	Homology from 25780925
rpoD (AL347_20760)	rpoD	PA3662 (AL347_24465)	PA3662	+	Homology from 25780925
rpoD (AL347_20760)	rpoD	PA3632 (AL347_24615)	PA3632	+	Homology from 25780925
rpoD (AL347_20760)	rpoD	PA3631 (AL347_24620)	PA3631	+	Homology from 25780925
rpoD (AL347_20760)	rpoD	PA3621 (AL347_24670)	PA3621 (fdxA)	+	Homology from 25780925
rpoD (AL347_20760)	rpoD	PA3610 (AL347_24730)	PA3610 (potD)	+	Homology from 25780925
rpoD (AL347_20760)	rpoD	PA3609 (AL347_24735)	PA3609 (potC)	+	Homology from 25780925
rpoD (AL347_20760)	rpoD	PA3608 (AL347_24740)	PA3608 (potB)	+	Homology from 25780925
rpoD (AL347_20760)	rpoD	PA3607 (AL347_24745)	PA3607 (potA)	+	Homology from 25780925
rpoD (AL347_20760)	rpoD	PA3605 (AL347_24755)	PA3605	+	Homology from 25780925
rpoD (AL347_20760)	rpoD	PA3602 (AL347_24770)	PA3602	+	Homology from 25780925
rpoD (AL347_20760)	rpoD	PA3573 (AL347_24935)	PA3573	+	Homology from 25780925
rpoD (AL347_20760)	rpoD	PA3559 (AL347_25010)	PA3559	+	Homology from 25780925
rpoD (AL347_20760)	rpoD	arnF (AL347_25015)	arnF	+	Homology from 25780925

rpoD (AL347_20760)	rpoD	arnE (AL347_25020)	arnE	+	Homology from 25780925
rpoD (AL347_20760)	rpoD	arnT (AL347_25025)	arnT	+	Homology from 25780925
rpoD (AL347_20760)	rpoD	arnD (AL347_25030)	arnD	+	Homology from 25780925
rpoD (AL347_20760)	rpoD	arnA (AL347_25035)	arnA	+	Homology from 25780925
rpoD (AL347_20760)	rpoD	arnC (AL347_25040)	arnC	+	Homology from 25780925
rpoD (AL347_20760)	rpoD	arnB (AL347_25045)	arnB	+	Homology from 25780925
rpoD (AL347_20760)	rpoD	PA3535 (AL347_25135)	PA3535	+	Homology from 25780925
rpoD (AL347_20760)	rpoD	bfd (AL347_25160)	bfd	+	Homology from 25780925
rpoD (AL347_20760)	rpoD	PA3529 (AL347_25165)	PA3529	+	Homology from 25780925
rpoD (AL347_20760)	rpoD	PA3524 (AL347_25190)	PA3524 (gloA1)	+	Homology from 25780925
rpoD (AL347_20760)	rpoD	PA3519 (AL347_25215)	PA3519	+	Homology from 25780925
rpoD (AL347_20760)	rpoD	PA3518 (AL347_25220)	PA3518	+	Homology from 25780925
rpoD (AL347_20760)	rpoD	PA3517 (AL347_25225)	PA3517	+	Homology from 25780925
rpoD (AL347_20760)	rpoD	PA3516 (AL347_25230)	PA3516	+	Homology from 25780925
rpoD (AL347_20760)	rpoD	PA3515 (AL347_25235)	PA3515	+	Homology from 25780925
rpoD (AL347_20760)	rpoD	PA3481 (AL347_25300)	PA3481	+	Homology from 25780925
rpoD (AL347_20760)	rpoD	rhlI (AL347_25330)	rhlI	+	Homology from 25780925
rpoD (AL347_20760)	rpoD	PA3475 (AL347_25335)	PA3475 (pheC)	+	Homology from 25780925
rpoD (AL347_20760)	rpoD	PA3456 (AL347_25435)	PA3456	+	Homology from 25780925
rpoD (AL347_20760)	rpoD	PA3455 (AL347_25445)	PA3455	+	Homology from 25780925
rpoD (AL347_20760)	rpoD	PA3432 (AL347_25560)	PA3432	+	Homology from 25780925
rpoD (AL347_20760)	rpoD	PA3431 (AL347_25565)	PA3431	+	Homology from 25780925
rpoD (AL347_20760)	rpoD	PA3428 (AL347_25580)	PA3428	+	Homology from 25780925
rpoD (AL347_20760)	rpoD	PA3390 (AL347_25785)	PA3390	+	Homology from 25780925
rpoD (AL347_20760)	rpoD	amrZ (AL347_25805)	amrZ	+	Homology from 25780925
rpoD (AL347_20760)	rpoD	PA3358 (AL347_25960)	PA3358	+	Homology from 25780925
rpoD (AL347_20760)	rpoD	PA3357 (AL347_25965)	PA3357 (dsdA)	+	Homology from 25780925
rpoD (AL347_20760)	rpoD	PA3356 (AL347_25970)	PA3356 (pauA5)	+	Homology from 25780925
rpoD (AL347_20760)	rpoD	PA3341 (AL347_26045)	PA3341	+	Homology from 25780925
rpoD (AL347_20760)	rpoD	PA3308 (AL347_26220)	PA3308 (hepA)	+	Homology from 25780925
rpoD (AL347_20760)	rpoD	PA3302 (AL347_26255)	PA3302	+	Homology from 25780925
rpoD (AL347_20760)	rpoD	PA3301 (AL347_26260)	PA3301	+	Homology from 25780925
rpoD (AL347_20760)	rpoD	PA3300 (AL347_26265)	PA3300 (fadD2)	+	Homology from 25780925
rpoD (AL347_20760)	rpoD	PA3299 (AL347_26270)	PA3299 (fadD1)	+	Homology from 25780925
rpoD (AL347_20760)	rpoD	PA3288 (AL347_00050)	PA3288	+	Homology from 25780925
rpoD (AL347_20760)	rpoD	PA3276 (AL347_00120)	PA3276	+	Homology from 25780925
rpoD (AL347_20760)	rpoD	PA3275 (AL347_00125)	PA3275	+	Homology from 25780925
rpoD (AL347_20760)	rpoD	PA3266 (AL347_26455)	PA3266 (capB, cspA)	+	Homology from 25780925

rpoD (AL347_20760)	rpoD	PA3263 (AL347_26470)	PA3263	+	Homology from 25780925
rpoD (AL347_20760)	rpoD	PA3230 (AL347_00370)	PA3230	+	Homology from 25780925
rpoD (AL347_20760)	rpoD	trkH (AL347_00470)	trkH	+	Homology from 25780925
rpoD (AL347_20760)	rpoD	PA3190 (AL347_00580)	PA3190	+	Homology from 25780925
rpoD (AL347_20760)	rpoD	PA3189 (AL347_26870)	PA3189	+	Homology from 25780925
rpoD (AL347_20760)	rpoD	PA3188 (AL347_00590)	PA3188	+	Homology from 25780925
rpoD (AL347_20760)	rpoD	PA3187 (AL347_00595)	PA3187	+	Homology from 25780925
rpoD (AL347_20760)	rpoD	oprB (AL347_00600)	oprB	+	Homology from 25780925
rpoD (AL347_20760)	rpoD	hexR (AL347_26900)	hexR	+	Homology from 25780925
rpoD (AL347_20760)	rpoD	gyrA (AL347_26980)	gyrA	+	Homology from 25780925
rpoD (AL347_20760)	rpoD	wbpI (AL347_27085)	wbpI	+	Homology from 25780925
rpoD (AL347_20760)	rpoD	PA3138 (AL347_27130)	PA3138 (uvrB)	+	Homology from 25780925
rpoD (AL347_20760)	rpoD	PA3134 (AL347_27150)	PA3134 (gltX)	+	Homology from 25780925
rpoD (AL347_20760)	rpoD	PA3133 (AL347_27175)	PA3133 (sawR)	+	Homology from 25780925
rpoD (AL347_20760)	rpoD	PA3132 (AL347_27180)	PA3132	+	Homology from 25780925
rpoD (AL347_20760)	rpoD	PA3121 (AL347_27235)	PA3121 (leuC)	+	Homology from 25780925
rpoD (AL347_20760)	rpoD	PA3120 (AL347_27240)	PA3120 (leuD)	+	Homology from 25780925
rpoD (AL347_20760)	rpoD	xcpQ (AL347_27315)	xcpQ	+	Homology from 25780925
rpoD (AL347_20760)	rpoD	xcpP (AL347_27320)	xcpP	+	Homology from 25780925
rpoD (AL347_20760)	rpoD	xcpR (AL347_27325)	xcpR	+	Homology from 25780925
rpoD (AL347_20760)	rpoD	xcpS (AL347_27330)	xcpS	+	Homology from 25780925
rpoD (AL347_20760)	rpoD	xcpT (AL347_27335)	xcpT	+	Homology from 25780925
rpoD (AL347_20760)	rpoD	xcpU (AL347_27340)	xcpU	+	Homology from 25780925
rpoD (AL347_20760)	rpoD	xcpV (AL347_27345)	xcpV	+	Homology from 25780925
rpoD (AL347_20760)	rpoD	xcpW (AL347_27350)	xcpW	+	Homology from 25780925
rpoD (AL347_20760)	rpoD	PA3097 (AL347_27355)	PA3097	+	Homology from 25780925
rpoD (AL347_20760)	rpoD	PA3096 (AL347_27360)	PA3096 (xcpY)	+	Homology from 25780925
rpoD (AL347_20760)	rpoD	xcpZ (AL347_27365)	xcpZ	+	Homology from 25780925
rpoD (AL347_20760)	rpoD	pelA (AL347_27525)	pelA	+	Homology from 25780925
rpoD (AL347_20760)	rpoD	pelB (AL347_27530)	pelB	+	Homology from 25780925
rpoD (AL347_20760)	rpoD	pelC (AL347_27535)	pelC	+	Homology from 25780925
rpoD (AL347_20760)	rpoD	pelD (AL347_27540)	pelD	+	Homology from 25780925
rpoD (AL347_20760)	rpoD	pelE (AL347_27545)	pelE	+	Homology from 25780925
rpoD (AL347_20760)	rpoD	pelF (AL347_27550)	pelF	+	Homology from 25780925
rpoD (AL347_20760)	rpoD	pelG (AL347_27555)	pelG	+	Homology from 25780925
rpoD (AL347_20760)	rpoD	PA3057 (AL347_27560)	PA3057	+	Homology from 25780925
rpoD (AL347_20760)	rpoD	PA3052 (AL347_27585)	PA3052	+	Homology from 25780925
rpoD (AL347_20760)	rpoD	PA3049 (AL347_27600)	PA3049 (rmf)	+	Homology from 25780925

rpoD (AL347_20760)	rpoD	PA3037 (AL347_27665)	PA3037	+	Homology from 25780925
rpoD (AL347_20760)	rpoD	PA3036 (AL347_27670)	PA3036	+	Homology from 25780925
rpoD (AL347_20760)	rpoD	PA3035 (AL347_27675)	PA3035	+	Homology from 25780925
rpoD (AL347_20760)	rpoD	PA3015 (AL347_27785)	PA3015	+	Homology from 25780925
rpoD (AL347_20760)	rpoD	faoA (AL347_27790)	faoA	+	Homology from 25780925
rpoD (AL347_20760)	rpoD	PA3003 (AL347_27845)	PA3003	+	Homology from 25780925
rpoD (AL347_20760)	rpoD	PA3002 (AL347_27850)	PA3002 (mfd)	+	Homology from 25780925
rpoD (AL347_20760)	rpoD	PA2975 (AL347_27995)	PA2975 (rluC, yceC)	+	Homology from 25780925
rpoD (AL347_20760)	rpoD	PA2974 (AL347_28000)	PA2974	+	Homology from 25780925
rpoD (AL347_20760)	rpoD	PA2973 (AL347_28005)	PA2973	+	Homology from 25780925
rpoD (AL347_20760)	rpoD	PA2972 (AL347_28010)	PA2972	+	Homology from 25780925
rpoD (AL347_20760)	rpoD	PA2960 (AL347_28070)	PA2960 (pilZ)	+	Homology from 25780925
rpoD (AL347_20760)	rpoD	PA2953 (AL347_28105)	PA2953	+	Homology from 25780925
rpoD (AL347_20760)	rpoD	etfB (AL347_28110)	etfB	+	Homology from 25780925
rpoD (AL347_20760)	rpoD	etfA (AL347_28115)	etfA	+	Homology from 25780925
rpoD (AL347_20760)	rpoD	PA2940 (AL347_28175)	PA2940	+	Homology from 25780925
rpoD (AL347_20760)	rpoD	PA2929 (AL347_28235)	PA2929	+	Homology from 25780925
rpoD (AL347_20760)	rpoD	PA2909 (AL347_28340)	PA2909	+	Homology from 25780925
rpoD (AL347_20760)	rpoD	PA2908 (AL347_28345)	PA2908 (cbiD)	+	Homology from 25780925
rpoD (AL347_20760)	rpoD	PA2907 (AL347_28350)	PA2907 (cobL, cbiE, cbiT)	+	Homology from 25780925
rpoD (AL347_20760)	rpoD	PA2894 (AL347_28415)	PA2894	+	Homology from 25780925
rpoD (AL347_20760)	rpoD	PA2877 (AL347_28500)	PA2877	+	Homology from 25780925
rpoD (AL347_20760)	rpoD	PA2876 (AL347_28505)	PA2876 (pyrF)	+	Homology from 25780925
rpoD (AL347_20760)	rpoD	PA2871 (AL347_28530)	PA2871	+	Homology from 25780925
rpoD (AL347_20760)	rpoD	PA2860 (AL347_28585)	PA2860	+	Homology from 25780925
rpoD (AL347_20760)	rpoD	PA2842 (AL347_28680)	PA2842	+	Homology from 25780925
rpoD (AL347_20760)	rpoD	PA2820 (AL347_28790)	PA2820	+	Homology from 25780925
rpoD (AL347_20760)	rpoD	PA2799 (AL347_28905)	PA2799	+	Homology from 25780925
rpoD (AL347_20760)	rpoD	PA2798 (AL347_28910)	PA2798	+	Homology from 25780925
rpoD (AL347_20760)	rpoD	PA2797 (AL347_28915)	PA2797	+	Homology from 25780925
rpoD (AL347_20760)	rpoD	PA2776 (AL347_29260)	PA2776 (pauB3, ordL)	+	Homology from 25780925
rpoD (AL347_20760)	rpoD	PA2775 (AL347_29275)	PA2775 (tsi4)	+	Homology from 25780925
rpoD (AL347_20760)	rpoD	PA2774 (AL347_29280)	PA2774 (tse4)	+	Homology from 25780925
rpoD (AL347_20760)	rpoD	PA2765 (AL347_29335)	PA2765	+	Homology from 25780925
rpoD (AL347_20760)	rpoD	PA2763 (AL347_29355)	PA2763	+	Homology from 25780925
rpoD (AL347_20760)	rpoD	PA2759 (AL347_29375)	PA2759	+	Homology from 25780925
rpoD (AL347_20760)	rpoD	PA2748 (AL347_29460)	PA2748	+	Homology from 25780925
rpoD (AL347_20760)	rpoD	PA2743 (AL347_29500)	PA2743 (infC)	+	Homology from 25780925

rpoD (AL347_20760)	rpoD	PA2742 (AL347_29505)	PA2742 (rpmI)	+	Homology from 25780925
rpoD (AL347_20760)	rpoD	PA2741 (AL347_29510)	PA2741 (rplT)	+	Homology from 25780925
rpoD (AL347_20760)	rpoD	himA (AL347_29525)	himA	+	Homology from 25780925
rpoD (AL347_20760)	rpoD	PA2723 (AL347_29775)	PA2723	+	Homology from 25780925
rpoD (AL347_20760)	rpoD	PA2712 (AL347_29840)	PA2712	+	Homology from 25780925
rpoD (AL347_20760)	rpoD	PA2678 (AL347_30020)	PA2678	+	Homology from 25780925
rpoD (AL347_20760)	rpoD	PA2677 (AL347_30025)	PA2677	+	Homology from 25780925
rpoD (AL347_20760)	rpoD	PA2676 (AL347_30030)	PA2676	+	Homology from 25780925
rpoD (AL347_20760)	rpoD	PA2675 (AL347_30035)	PA2675	+	Homology from 25780925
rpoD (AL347_20760)	rpoD	PA2674 (AL347_30040)	PA2674	+	Homology from 25780925
rpoD (AL347_20760)	rpoD	PA2673 (AL347_30045)	PA2673	+	Homology from 25780925
rpoD (AL347_20760)	rpoD	PA2672 (AL347_30050)	PA2672	+	Homology from 25780925
rpoD (AL347_20760)	rpoD	PA2671 (AL347_30055)	PA2671	+	Homology from 25780925
rpoD (AL347_20760)	rpoD	fhpR (AL347_30085)	fhpR	+	Homology from 25780925
rpoD (AL347_20760)	rpoD	PA2624 (AL347_30305)	PA2624 (idh)	+	Homology from 25780925
rpoD (AL347_20760)	rpoD	PA2623 (AL347_30310)	PA2623 (icd, icdA)	+	Homology from 25780925
rpoD (AL347_20760)	rpoD	PA2622 (AL347_30315)	PA2622 (cspD)	+	Homology from 25780925
rpoD (AL347_20760)	rpoD	PA2620 (AL347_30325)	PA2620 (clpA)	+	Homology from 25780925
rpoD (AL347_20760)	rpoD	PA2619 (AL347_30330)	PA2619 (infA)	+	Homology from 25780925
rpoD (AL347_20760)	rpoD	PA2603 (AL347_30415)	PA2603	+	Homology from 25780925
rpoD (AL347_20760)	rpoD	PA2602 (AL347_30420)	PA2602	+	Homology from 25780925
rpoD (AL347_20760)	rpoD	PA2601 (AL347_30425)	PA2601	+	Homology from 25780925
rpoD (AL347_20760)	rpoD	PA2590 (AL347_30480)	PA2590	+	Homology from 25780925
rpoD (AL347_20760)	rpoD	PA2589 (AL347_30485)	PA2589	+	Homology from 25780925
rpoD (AL347_20760)	rpoD	PA2581 (AL347_31720)	PA2581	+	Homology from 25780925
rpoD (AL347_20760)	rpoD	PA2576 (AL347_31745)	PA2576	+	Homology from 25780925
rpoD (AL347_20760)	rpoD	lecA (AL347_31780)	lecA	+	Homology from 25780925
rpoD (AL347_20760)	rpoD	PA2557 (AL347_31855)	PA2557	+	Homology from 25780925
rpoD (AL347_20760)	rpoD	PA2556 (AL347_31860)	PA2556	+	Homology from 25780925
rpoD (AL347_20760)	rpoD	PA2551 (AL347_31885)	PA2551	+	Homology from 25780925
rpoD (AL347_20760)	rpoD	PA2534 (AL347_31975)	PA2534	+	Homology from 25780925
rpoD (AL347_20760)	rpoD	antC (AL347_32090)	antC	+	Homology from 25780925
rpoD (AL347_20760)	rpoD	antB (AL347_32095)	antB	+	Homology from 25780925
rpoD (AL347_20760)	rpoD	antA (AL347_32100)	antA	+	Homology from 25780925
rpoD (AL347_20760)	rpoD	PA2501 (AL347_32470)	PA2501	+	Homology from 25780925
rpoD (AL347_20760)	rpoD	PA2497 (AL347_32490)	PA2497	+	Homology from 25780925
rpoD (AL347_20760)	rpoD	PA2496 (AL347_32495)	PA2496	+	Homology from 25780925
rpoD (AL347_20760)	rpoD	mexT (AL347_32559)	mexT	+	Homology from 25780925

rpoD (AL347_20760)	rpoD	mexS (AL347_32565)	mexS	+	Homology from 25780925
rpoD (AL347_20760)	rpoD	PA2478 (AL347_32630)	PA2478	+	Homology from 25780925
rpoD (AL347_20760)	rpoD	PA2477 (AL347_32635)	PA2477	+	Homology from 25780925
rpoD (AL347_20760)	rpoD	PA2476 (AL347_32640)	PA2476 (dsbG)	+	Homology from 25780925
rpoD (AL347_20760)	rpoD	PA2463 (AL347_32705)	PA2463	+	Homology from 25780925
rpoD (AL347_20760)	rpoD	PA2462 (AL347_32710)	PA2462	+	Homology from 25780925
rpoD (AL347_20760)	rpoD	PA2455 (AL347_32740)	PA2455	+	Homology from 25780925
rpoD (AL347_20760)	rpoD	PA2454 (AL347_32745)	PA2454	+	Homology from 25780925
rpoD (AL347_20760)	rpoD	PA2453 (AL347_32750)	PA2453	+	Homology from 25780925
rpoD (AL347_20760)	rpoD	PA2447 (AL347_32775)	PA2447	+	Homology from 25780925
rpoD (AL347_20760)	rpoD	PA2429 (AL347_32880)	PA2429	+	Homology from 25780925
rpoD (AL347_20760)	rpoD	PA2423 (AL347_32910)	PA2423	+	Homology from 25780925
rpoD (AL347_20760)	rpoD	PA2381 (AL347_33110)	PA2381	+	Homology from 25780925
rpoD (AL347_20760)	rpoD	PA2315 (AL347_33450)	PA2315	+	Homology from 25780925
rpoD (AL347_20760)	rpoD	PA2314 (AL347_33455)	PA2314	+	Homology from 25780925
rpoD (AL347_20760)	rpoD	pvcD (AL347_33745)	pvcD	+	Homology from 25780925
rpoD (AL347_20760)	rpoD	pvcC (AL347_00780)	pvcC	+	Homology from 25780925
rpoD (AL347_20760)	rpoD	pvcB (AL347_00785)	pvcB	+	Homology from 25780925
rpoD (AL347_20760)	rpoD	pvcA (AL347_00790)	pvcA	+	Homology from 25780925
rpoD (AL347_20760)	rpoD	bkdB (AL347_00820)	bkdB	+	Homology from 25780925
rpoD (AL347_20760)	rpoD	bkdA2 (AL347_00825)	bkdA2	+	Homology from 25780925
rpoD (AL347_20760)	rpoD	bkdA1 (AL347_00830)	bkdA1	+	Homology from 25780925
rpoD (AL347_20760)	rpoD	qsrO (AL347_33910)	qsrO	+	Homology from 25780925
rpoD (AL347_20760)	rpoD	PA2209 (AL347_01505)	PA2209	+	Homology from 25780925
rpoD (AL347_20760)	rpoD	PA2208 (AL347_01510)	PA2208	+	Homology from 25780925
rpoD (AL347_20760)	rpoD	PA2207 (AL347_01515)	PA2207	+	Homology from 25780925
rpoD (AL347_20760)	rpoD	PA2127 (AL347_01955)	PA2127 (cgrA)	+	Homology from 25780925
rpoD (AL347_20760)	rpoD	PA2118 (AL347_02015)	PA2118 (ada)	+	Homology from 25780925
rpoD (AL347_20760)	rpoD	PA2107 (AL347_02075)	PA2107	+	Homology from 25780925
rpoD (AL347_20760)	rpoD	kynB (AL347_02175)	kynB	+	Homology from 25780925
rpoD (AL347_20760)	rpoD	kynU (AL347_02180)	kynU	+	Homology from 25780925
rpoD (AL347_20760)	rpoD	PA2079 (AL347_02190)	PA2079	+	Homology from 25780925
rpoD (AL347_20760)	rpoD	PA2075 (AL347_02220)	PA2075	+	Homology from 25780925
rpoD (AL347_20760)	rpoD	PA2054 (AL347_02335)	PA2054 (cynR)	+	Homology from 25780925
rpoD (AL347_20760)	rpoD	PA2040 (AL347_02410)	PA2040 (pauA4)	+	Homology from 25780925
rpoD (AL347_20760)	rpoD	PA2029 (AL347_02460)	PA2029	+	Homology from 25780925
rpoD (AL347_20760)	rpoD	PA2028 (AL347_02465)	PA2028	+	Homology from 25780925
rpoD (AL347_20760)	rpoD	PA2027 (AL347_02470)	PA2027	+	Homology from 25780925

rpoD (AL347_20760)	rpoD	PA2017 (AL347_02525)	PA2017	+	Homology from 25780925
rpoD (AL347_20760)	rpoD	liuR (AL347_02530)	liuR	+	Homology from 25780925
rpoD (AL347_20760)	rpoD	liuA (AL347_02535)	liuA	+	Homology from 25780925
rpoD (AL347_20760)	rpoD	liuB (AL347_02540)	liuB	+	Homology from 25780925
rpoD (AL347_20760)	rpoD	liuC (AL347_02545)	liuC	+	Homology from 25780925
rpoD (AL347_20760)	rpoD	liuD (AL347_02550)	liuD	+	Homology from 25780925
rpoD (AL347_20760)	rpoD	liuE (AL347_02555)	liuE	+	Homology from 25780925
rpoD (AL347_20760)	rpoD	dhcB (AL347_02610)	dhcB	+	Homology from 25780925
rpoD (AL347_20760)	rpoD	dhcA (AL347_02615)	dhcA	+	Homology from 25780925
rpoD (AL347_20760)	rpoD	dhcR (AL347_02620)	dhcR	+	Homology from 25780925
rpoD (AL347_20760)	rpoD	PA1997 (AL347_02625)	PA1997	+	Homology from 25780925
rpoD (AL347_20760)	rpoD	PA1996 (AL347_02630)	PA1996 (ppiC1)	+	Homology from 25780925
rpoD (AL347_20760)	rpoD	PA1995 (AL347_02635)	PA1995	+	Homology from 25780925
rpoD (AL347_20760)	rpoD	PA1994 (AL347_02640)	PA1994	+	Homology from 25780925
rpoD (AL347_20760)	rpoD	braZ (AL347_02755)	braZ	+	Homology from 25780925
rpoD (AL347_20760)	rpoD	PA1956 (AL347_02835)	PA1956 (fapA)	+	Homology from 25780925
rpoD (AL347_20760)	rpoD	PA1955 (AL347_02840)	PA1955 (fapB)	+	Homology from 25780925
rpoD (AL347_20760)	rpoD	PA1954 (AL347_02845)	PA1954 (fapC)	+	Homology from 25780925
rpoD (AL347_20760)	rpoD	PA1953 (AL347_02850)	PA1953 (fapD)	+	Homology from 25780925
rpoD (AL347_20760)	rpoD	PA1952 (AL347_02855)	PA1952 (fapE)	+	Homology from 25780925
rpoD (AL347_20760)	rpoD	rbsK (AL347_02865)	rbsK	+	Homology from 25780925
rpoD (AL347_20760)	rpoD	rbsR (AL347_02870)	rbsR	+	Homology from 25780925
rpoD (AL347_20760)	rpoD	PA1948 (AL347_02875)	PA1948	+	Homology from 25780925
rpoD (AL347_20760)	rpoD	PA1947 (AL347_02880)	PA1947	+	Homology from 25780925
rpoD (AL347_20760)	rpoD	PA1946 (AL347_02885)	PA1946	+	Homology from 25780925
rpoD (AL347_20760)	rpoD	PA1940 (AL347_02915)	PA1940	+	Homology from 25780925
rpoD (AL347_20760)	rpoD	PA1925 (AL347_02975)	PA1925	+	Homology from 25780925
rpoD (AL347_20760)	rpoD	PA1924 (AL347_02980)	PA1924	+	Homology from 25780925
rpoD (AL347_20760)	rpoD	PA1923 (AL347_02985)	PA1923	+	Homology from 25780925
rpoD (AL347_20760)	rpoD	PA1922 (AL347_02990)	PA1922	+	Homology from 25780925
rpoD (AL347_20760)	rpoD	qscR (AL347_03130)	qscR	+	Homology from 25780925
rpoD (AL347_20760)	rpoD	PA1864 (AL347_03305)	PA1864	+	Homology from 25780925
rpoD (AL347_20760)	rpoD	PA1856 (AL347_03345)	PA1856	+	Homology from 25780925
rpoD (AL347_20760)	rpoD	PA1855 (AL347_03350)	PA1855	+	Homology from 25780925
rpoD (AL347_20760)	rpoD	nfuA (AL347_03395)	nfuA	+	Homology from 25780925
rpoD (AL347_20760)	rpoD	PA1805 (AL347_03615)	PA1805 (ppiD)	+	Homology from 25780925
rpoD (AL347_20760)	rpoD	hupB (AL347_03625)	hupB	+	Homology from 25780925
rpoD (AL347_20760)	rpoD	PA1796 (AL347_03695)	PA1796 (folD)	+	Homology from 25780925

rpoD (AL347_20760)	rpoD	PA1789 (AL347_03730)	PA1789	+	Homology from 25780925
rpoD (AL347_20760)	rpoD	PA1772 (AL347_03820)	PA1772	+	Homology from 25780925
rpoD (AL347_20760)	rpoD	PA1761 (AL347_03880)	PA1761	+	Homology from 25780925
rpoD (AL347_20760)	rpoD	PA1760 (AL347_03890)	PA1760	+	Homology from 25780925
rpoD (AL347_20760)	rpoD	PA1759 (AL347_03895)	PA1759	+	Homology from 25780925
rpoD (AL347_20760)	rpoD	PA1758 (AL347_03900)	PA1758 (pabB)	+	Homology from 25780925
rpoD (AL347_20760)	rpoD	cysB (AL347_03920)	cysB	+	Homology from 25780925
rpoD (AL347_20760)	rpoD	PA1750 (AL347_03945)	PA1750	+	Homology from 25780925
rpoD (AL347_20760)	rpoD	PA1747 (AL347_03960)	PA1747	+	Homology from 25780925
rpoD (AL347_20760)	rpoD	fadB (AL347_04010)	fadB	+	Homology from 25780925
rpoD (AL347_20760)	rpoD	PA1736 (AL347_04015)	PA1736	+	Homology from 25780925
rpoD (AL347_20760)	rpoD	mucR (AL347_04065)	mucR	+	Homology from 25780925
rpoD (AL347_20760)	rpoD	PA1709 (AL347_04155)	PA1709 (popD, pepD)	+	Homology from 25780925
rpoD (AL347_20760)	rpoD	PA1708 (AL347_04160)	PA1708 (popB, pepB)	+	Homology from 25780925
rpoD (AL347_20760)	rpoD	PA1707 (AL347_04165)	PA1707 (pcrH)	+	Homology from 25780925
rpoD (AL347_20760)	rpoD	PA1706 (AL347_04170)	PA1706 (pcrV)	+	Homology from 25780925
rpoD (AL347_20760)	rpoD	PA1705 (AL347_04175)	PA1705 (pcrG)	+	Homology from 25780925
rpoD (AL347_20760)	rpoD	PA1704 (AL347_04180)	PA1704 (pcrR)	+	Homology from 25780925
rpoD (AL347_20760)	rpoD	PA1703 (AL347_04185)	PA1703 (pcrD)	+	Homology from 25780925
rpoD (AL347_20760)	rpoD	PA1702 (AL347_04190)	PA1702 (pcr4)	+	Homology from 25780925
rpoD (AL347_20760)	rpoD	PA1701 (AL347_04195)	PA1701 (pcr3)	+	Homology from 25780925
rpoD (AL347_20760)	rpoD	PA1700 (AL347_04200)	PA1700 (pcr2)	+	Homology from 25780925
rpoD (AL347_20760)	rpoD	PA1699 (AL347_04205)	PA1699 (pcr1)	+	Homology from 25780925
rpoD (AL347_20760)	rpoD	PA1677 (AL347_04320)	PA1677	+	Homology from 25780925
rpoD (AL347_20760)	rpoD	PA1676 (AL347_04325)	PA1676	+	Homology from 25780925
rpoD (AL347_20760)	rpoD	PA1675 (AL347_04330)	PA1675	+	Homology from 25780925
rpoD (AL347_20760)	rpoD	PA1643 (AL347_04505)	PA1643	+	Homology from 25780925
rpoD (AL347_20760)	rpoD	PA1642 (AL347_04510)	PA1642 (selD)	+	Homology from 25780925
rpoD (AL347_20760)	rpoD	PA1627 (AL347_04585)	PA1627	+	Homology from 25780925
rpoD (AL347_20760)	rpoD	PA1626 (AL347_04590)	PA1626	+	Homology from 25780925
rpoD (AL347_20760)	rpoD	PA1590 (AL347_04775)	PA1590 (braB)	+	Homology from 25780925
rpoD (AL347_20760)	rpoD	PA1584 (AL347_04805)	PA1584 (sdhB)	+	Homology from 25780925
rpoD (AL347_20760)	rpoD	PA1583 (AL347_04810)	PA1583 (sdhA)	+	Homology from 25780925
rpoD (AL347_20760)	rpoD	PA1582 (AL347_04815)	PA1582 (sdhD)	+	Homology from 25780925
rpoD (AL347_20760)	rpoD	PA1581 (AL347_04820)	PA1581 (sdhC, cybA)	+	Homology from 25780925
rpoD (AL347_20760)	rpoD	PA1580 (AL347_04825)	PA1580 (gltA, cisY)	+	Homology from 25780925
rpoD (AL347_20760)	rpoD	PA1570 (AL347_04880)	PA1570	+	Homology from 25780925
rpoD (AL347_20760)	rpoD	PA1519 (AL347_05165)	PA1519	+	Homology from 25780925

rpoD (AL347_20760)	rpoD	PA1518 (AL347_05170)	PA1518	+	Homology from 25780925
rpoD (AL347_20760)	rpoD	PA1507 (AL347_05225)	PA1507	+	Homology from 25780925
rpoD (AL347_20760)	rpoD	PA1505 (AL347_05235)	PA1505 (moaA2)	+	Homology from 25780925
rpoD (AL347_20760)	rpoD	PA1504 (AL347_05240)	PA1504	+	Homology from 25780925
rpoD (AL347_20760)	rpoD	PA1503 (AL347_05245)	PA1503	+	Homology from 25780925
rpoD (AL347_20760)	rpoD	PA1483 (AL347_05350)	PA1483 (cycH)	+	Homology from 25780925
rpoD (AL347_20760)	rpoD	PA1482 (AL347_05355)	PA1482 (ccmH, cycL)	+	Homology from 25780925
rpoD (AL347_20760)	rpoD	ccmG (AL347_05360)	ccmG	+	Homology from 25780925
rpoD (AL347_20760)	rpoD	PA1480 (AL347_05365)	PA1480 (ccmF, cycK)	+	Homology from 25780925
rpoD (AL347_20760)	rpoD	PA1479 (AL347_05370)	PA1479 (ccmE, cycJ)	+	Homology from 25780925
rpoD (AL347_20760)	rpoD	PA1478 (AL347_05375)	PA1478	+	Homology from 25780925
rpoD (AL347_20760)	rpoD	PA1477 (AL347_05380)	PA1477 (ccmC, helC, cycZ)	+	Homology from 25780925
rpoD (AL347_20760)	rpoD	rsaL (AL347_05610)	rsaL	+	Homology from 25780925
rpoD (AL347_20760)	rpoD	PA1412 (AL347_05755)	PA1412	+	Homology from 25780925
rpoD (AL347_20760)	rpoD	PA1269 (AL347_06765)	PA1269	+	Homology from 25780925
rpoD (AL347_20760)	rpoD	PA1262 (AL347_06810)	PA1262	+	Homology from 25780925
rpoD (AL347_20760)	rpoD	PA1252 (AL347_06860)	PA1252 (dpkA)	+	Homology from 25780925
rpoD (AL347_20760)	rpoD	aprA (AL347_06875)	aprA	+	Homology from 25780925
rpoD (AL347_20760)	rpoD	PA1241 (AL347_06925)	PA1241	+	Homology from 25780925
rpoD (AL347_20760)	rpoD	PA1240 (AL347_06930)	PA1240	+	Homology from 25780925
rpoD (AL347_20760)	rpoD	PA1239 (AL347_06935)	PA1239	+	Homology from 25780925
rpoD (AL347_20760)	rpoD	PA1238 (AL347_06940)	PA1238	+	Homology from 25780925
rpoD (AL347_20760)	rpoD	PA1237 (AL347_06945)	PA1237	+	Homology from 25780925
rpoD (AL347_20760)	rpoD	PA1236 (AL347_06950)	PA1236	+	Homology from 25780925
rpoD (AL347_20760)	rpoD	PA1225 (AL347_07005)	PA1225	+	Homology from 25780925
rpoD (AL347_20760)	rpoD	PA1200 (AL347_07130)	PA1200	+	Homology from 25780925
rpoD (AL347_20760)	rpoD	PA1199 (AL347_07135)	PA1199	+	Homology from 25780925
rpoD (AL347_20760)	rpoD	PA1198 (AL347_07140)	PA1198	+	Homology from 25780925
rpoD (AL347_20760)	rpoD	ddaH (AL347_07155)	ddaH	+	Homology from 25780925
rpoD (AL347_20760)	rpoD	dctR (AL347_07225)	dctR	+	Homology from 25780925
rpoD (AL347_20760)	rpoD	PA1149 (AL347_07410)	PA1149	+	Homology from 25780925
rpoD (AL347_20760)	rpoD	PA1139 (AL347_07460)	PA1139	+	Homology from 25780925
rpoD (AL347_20760)	rpoD	PA1135 (AL347_07480)	PA1135	+	Homology from 25780925
rpoD (AL347_20760)	rpoD	PA1126 (AL347_07525)	PA1126	+	Homology from 25780925
rpoD (AL347_20760)	rpoD	PA1122 (AL347_17845)	PA1122	+	Homology from 25780925
rpoD (AL347_20760)	rpoD	PA1050 (AL347_07935)	PA1050	+	Homology from 25780925
rpoD (AL347_20760)	rpoD	PA1049 (AL347_07940)	PA1049 (pdxH)	+	Homology from 25780925
rpoD (AL347_20760)	rpoD	PA1044 (AL347_07965)	PA1044	+	Homology from 25780925

rpoD (AL347_20760)	rpoD	PA1036 (AL347_08005)	PA1036	+	Homology from 25780925
rpoD (AL347_20760)	rpoD	PA1014 (AL347_08130)	PA1014 (wapB)	+	Homology from 25780925
rpoD (AL347_20760)	rpoD	PA1013 (AL347_08140)	PA1013 (purC)	+	Homology from 25780925
rpoD (AL347_20760)	rpoD	PA1012 (AL347_08145)	PA1012	+	Homology from 25780925
rpoD (AL347_20760)	rpoD	PA1011 (AL347_08150)	PA1011	+	Homology from 25780925
rpoD (AL347_20760)	rpoD	PA1010 (AL347_08155)	PA1010 (dapA)	+	Homology from 25780925
rpoD (AL347_20760)	rpoD	PA1009 (AL347_08160)	PA1009	+	Homology from 25780925
rpoD (AL347_20760)	rpoD	PA1008 (AL347_08165)	PA1008 (bcp)	+	Homology from 25780925
rpoD (AL347_20760)	rpoD	PA0960 (AL347_08405)	PA0960	+	Homology from 25780925
rpoD (AL347_20760)	rpoD	PA0952 (AL347_08445)	PA0952	+	Homology from 25780925
rpoD (AL347_20760)	rpoD	PA0941 (AL347_08510)	PA0941	+	Homology from 25780925
rpoD (AL347_20760)	rpoD	PA0940 (AL347_08515)	PA0940	+	Homology from 25780925
rpoD (AL347_20760)	rpoD	PA0939 (AL347_08520)	PA0939	+	Homology from 25780925
rpoD (AL347_20760)	rpoD	PA0938 (AL347_08530)	PA0938 (wzz2)	+	Homology from 25780925
rpoD (AL347_20760)	rpoD	PA0937 (AL347_08535)	PA0937	+	Homology from 25780925
rpoD (AL347_20760)	rpoD	PA0936 (AL347_08540)	PA0936 (lpxO2)	+	Homology from 25780925
rpoD (AL347_20760)	rpoD	PA0925 (AL347_08595)	PA0925	+	Homology from 25780925
rpoD (AL347_20760)	rpoD	PA0922 (AL347_08615)	PA0922	+	Homology from 25780925
rpoD (AL347_20760)	rpoD	PA0909 (AL347_08935)	PA0909 (alpC)	+	Homology from 25780925
rpoD (AL347_20760)	rpoD	PA0908 (AL347_08940)	PA0908 (alpB)	+	Homology from 25780925
rpoD (AL347_20760)	rpoD	rsmA (AL347_08970)	rsmA	+	Homology from 25780925
rpoD (AL347_20760)	rpoD	acsA (AL347_09060)	acsA	+	Homology from 25780925
rpoD (AL347_20760)	rpoD	phhA (AL347_09145)	phhA	+	Homology from 25780925
rpoD (AL347_20760)	rpoD	PA0858 (AL347_09220)	PA0858	+	Homology from 25780925
rpoD (AL347_20760)	rpoD	PA0850 (AL347_09260)	PA0850	+	Homology from 25780925
rpoD (AL347_20760)	rpoD	PA0842 (AL347_09300)	PA0842	+	Homology from 25780925
rpoD (AL347_20760)	rpoD	PA0840 (AL347_09310)	PA0840	+	Homology from 25780925
rpoD (AL347_20760)	rpoD	PA0839 (AL347_09315)	PA0839	+	Homology from 25780925
rpoD (AL347_20760)	rpoD	PA0838 (AL347_09320)	PA0838	+	Homology from 25780925
rpoD (AL347_20760)	rpoD	PA0831 (AL347_09360)	PA0831	+	Homology from 25780925
rpoD (AL347_20760)	rpoD	PA0827 (AL347_09380)	PA0827	+	Homology from 25780925
rpoD (AL347_20760)	rpoD	PA0806 (AL347_09480)	PA0806	+	Homology from 25780925
rpoD (AL347_20760)	rpoD	PA0797 (AL347_09525)	PA0797	+	Homology from 25780925
rpoD (AL347_20760)	rpoD	prpB (AL347_09530)	prpB	+	Homology from 25780925
rpoD (AL347_20760)	rpoD	prpC (AL347_09535)	prpC	+	Homology from 25780925
rpoD (AL347_20760)	rpoD	PA0794 (AL347_09540)	PA0794	+	Homology from 25780925
rpoD (AL347_20760)	rpoD	PA0787 (AL347_09580)	PA0787	+	Homology from 25780925
rpoD (AL347_20760)	rpoD	PA0786 (AL347_09585)	PA0786	+	Homology from 25780925

rpoD (AL347_20760)	rpoD	PA0785 (AL347_09590)	PA0785 (azoR1, azoR)	+	Homology from 25780925
rpoD (AL347_20760)	rpoD	PA0768 (AL347_09675)	PA0768 (lepB)	+	Homology from 25780925
rpoD (AL347_20760)	rpoD	PA0767 (AL347_09680)	PA0767 (lepA)	+	Homology from 25780925
rpoD (AL347_20760)	rpoD	PA0714 (AL347_09975)	PA0714	+	Homology from 25780925
rpoD (AL347_20760)	rpoD	PA0710 (AL347_09995)	PA0710 (gloA2)	+	Homology from 25780925
rpoD (AL347_20760)	rpoD	PA0709 (AL347_10000)	PA0709	+	Homology from 25780925
rpoD (AL347_20760)	rpoD	PA0708 (AL347_10005)	PA0708	+	Homology from 25780925
rpoD (AL347_20760)	rpoD	PA0664 (AL347_21200)	PA0664	+	Homology from 25780925
rpoD (AL347_20760)	rpoD	PA0663 (AL347_21195)	PA0663	+	Homology from 25780925
rpoD (AL347_20760)	rpoD	PA0662 (AL347_21190)	PA0662 (argC)	+	Homology from 25780925
rpoD (AL347_20760)	rpoD	PA0657 (AL347_21165)	PA0657	+	Homology from 25780925
rpoD (AL347_20760)	rpoD	PA0651 (AL347_21135)	PA0651 (trpC)	+	Homology from 25780925
rpoD (AL347_20760)	rpoD	PA0650 (AL347_21130)	PA0650 (trpD)	+	Homology from 25780925
rpoD (AL347_20760)	rpoD	PA0649 (AL347_21125)	PA0649 (trpG, pabA)	+	Homology from 25780925
rpoD (AL347_20760)	rpoD	PA0635 (AL347_21055)	PA0635	+	Homology from 25780925
rpoD (AL347_20760)	rpoD	PA0634 (AL347_21050)	PA0634	+	Homology from 25780925
rpoD (AL347_20760)	rpoD	PA0633 (AL347_21045)	PA0633	+	Homology from 25780925
rpoD (AL347_20760)	rpoD	PA0632 (AL347_21040)	PA0632	+	Homology from 25780925
rpoD (AL347_20760)	rpoD	PA0631 (AL347_21035)	PA0631	+	Homology from 25780925
rpoD (AL347_20760)	rpoD	PA0630 (AL347_21030)	PA0630	+	Homology from 25780925
rpoD (AL347_20760)	rpoD	PA0629 (AL347_21025)	PA0629	+	Homology from 25780925
rpoD (AL347_20760)	rpoD	PA0628 (AL347_21020)	PA0628	+	Homology from 25780925
rpoD (AL347_20760)	rpoD	PA0598 (AL347_20870)	PA0598	+	Homology from 25780925
rpoD (AL347_20760)	rpoD	PA0597 (AL347_20865)	PA0597	+	Homology from 25780925
rpoD (AL347_20760)	rpoD	PA0596 (AL347_20860)	PA0596	+	Homology from 25780925
rpoD (AL347_20760)	rpoD	PA0595 (AL347_20855)	PA0595 (lptD, ostA)	+	Homology from 25780925
rpoD (AL347_20760)	rpoD	surA (AL347_20850)	surA	+	Homology from 25780925
rpoD (AL347_20760)	rpoD	PA0593 (AL347_20845)	PA0593 (pdxA)	+	Homology from 25780925
rpoD (AL347_20760)	rpoD	PA0583 (AL347_20795)	PA0583	+	Homology from 25780925
rpoD (AL347_20760)	rpoD	PA0582 (AL347_20790)	PA0582 (folB)	+	Homology from 25780925
rpoD (AL347_20760)	rpoD	PA0580 (AL347_20780)	PA0580 (gcp, ygjD)	+	Homology from 25780925
rpoD (AL347_20760)	rpoD	PA0579 (AL347_20775)	PA0579 (rpsU)	+	Homology from 25780925
rpoD (AL347_20760)	rpoD	rpoD (AL347_20760)	rpoD	+	Homology from 25780925
rpoD (AL347_20760)	rpoD	PA0544 (AL347_20565)	PA0544	+	Homology from 25780925
rpoD (AL347_20760)	rpoD	PA0540 (AL347_20545)	PA0540	+	Homology from 25780925
rpoD (AL347_20760)	rpoD	PA0539 (AL347_20540)	PA0539	+	Homology from 25780925
rpoD (AL347_20760)	rpoD	PA0538 (AL347_20535)	PA0538 (dsbB)	+	Homology from 25780925
rpoD (AL347_20760)	rpoD	PA0535 (AL347_20520)	PA0535	+	Homology from 25780925

rpoD (AL347_20760)	rpoD	PA0534 (AL347_20515)	PA0534 (pauB1)	+	Homology from 25780925
rpoD (AL347_20760)	rpoD	PA0528 (AL347_20475)	PA0528	+	Homology from 25780925
rpoD (AL347_20760)	rpoD	dnr (AL347_20470)	dnr	+	Homology from 25780925
rpoD (AL347_20760)	rpoD	PA0526 (AL347_20465)	PA0526	+	Homology from 25780925
rpoD (AL347_20760)	rpoD	PA0522 (AL347_20445)	PA0522	+	Homology from 25780925
rpoD (AL347_20760)	rpoD	PA0521 (AL347_20440)	PA0521	+	Homology from 25780925
rpoD (AL347_20760)	rpoD	nirQ (AL347_20435)	nirQ	+	Homology from 25780925
rpoD (AL347_20760)	rpoD	nirS (AL347_20430)	nirS	+	Homology from 25780925
rpoD (AL347_20760)	rpoD	PA0492 (AL347_20295)	PA0492	+	Homology from 25780925
rpoD (AL347_20760)	rpoD	PA0491 (AL347_20290)	PA0491	+	Homology from 25780925
rpoD (AL347_20760)	rpoD	PA0485 (AL347_20260)	PA0485	+	Homology from 25780925
rpoD (AL347_20760)	rpoD	PA0468 (AL347_20175)	PA0468	+	Homology from 25780925
rpoD (AL347_20760)	rpoD	PA0467 (AL347_20170)	PA0467	+	Homology from 25780925
rpoD (AL347_20760)	rpoD	PA0457 (AL347_20110)	PA0457	+	Homology from 25780925
rpoD (AL347_20760)	rpoD	PA0456 (AL347_20105)	PA0456	+	Homology from 25780925
rpoD (AL347_20760)	rpoD	PA0455 (AL347_20100)	PA0455 (dbpA)	+	Homology from 25780925
rpoD (AL347_20760)	rpoD	PA0447 (AL347_20060)	PA0447 (gcdH)	+	Homology from 25780925
rpoD (AL347_20760)	rpoD	PA0444 (AL347_20050)	PA0444	+	Homology from 25780925
rpoD (AL347_20760)	rpoD	PA0443 (AL347_20045)	PA0443	+	Homology from 25780925
rpoD (AL347_20760)	rpoD	PA0442 (AL347_20040)	PA0442	+	Homology from 25780925
rpoD (AL347_20760)	rpoD	dht (AL347_20030)	dht	+	Homology from 25780925
rpoD (AL347_20760)	rpoD	PA0440 (AL347_20025)	PA0440	+	Homology from 25780925
rpoD (AL347_20760)	rpoD	PA0439 (AL347_20020)	PA0439	+	Homology from 25780925
rpoD (AL347_20760)	rpoD	PA0428 (AL347_19960)	PA0428	+	Homology from 25780925
rpoD (AL347_20760)	rpoD	mexR (AL347_19940)	mexR	+	Homology from 25780925
rpoD (AL347_20760)	rpoD	PA0391 (AL347_19765)	PA0391	+	Homology from 25780925
rpoD (AL347_20760)	rpoD	PA0390 (AL347_19760)	PA0390 (metX)	+	Homology from 25780925
rpoD (AL347_20760)	rpoD	PA0389 (AL347_19755)	PA0389	+	Homology from 25780925
rpoD (AL347_20760)	rpoD	PA0360 (AL347_19605)	PA0360	+	Homology from 25780925
rpoD (AL347_20760)	rpoD	PA0338 (AL347_19495)	PA0338	+	Homology from 25780925
rpoD (AL347_20760)	rpoD	PA0337 (AL347_19490)	PA0337 (ptsP)	+	Homology from 25780925
rpoD (AL347_20760)	rpoD	PA0336 (AL347_19485)	PA0336 (rppH, ygdP)	+	Homology from 25780925
rpoD (AL347_20760)	rpoD	PA0335 (AL347_19480)	PA0335	+	Homology from 25780925
rpoD (AL347_20760)	rpoD	PA0334 (AL347_19475)	PA0334	+	Homology from 25780925
rpoD (AL347_20760)	rpoD	PA0328 (AL347_19440)	PA0328 (aaaA)	+	Homology from 25780925
rpoD (AL347_20760)	rpoD	PA0312 (AL347_19355)	PA0312	+	Homology from 25780925
rpoD (AL347_20760)	rpoD	PA0311 (AL347_19350)	PA0311	+	Homology from 25780925
rpoD (AL347_20760)	rpoD	PA0308 (AL347_19335)	PA0308	+	Homology from 25780925

rpoD (AL347_20760)	rpoD	PA0305 (AL347_19310)	PA0305 (hacB)	+	Homology from 25780925
rpoD (AL347_20760)	rpoD	spuH (AL347_19305)	spuH	+	Homology from 25780925
rpoD (AL347_20760)	rpoD	spuG (AL347_19300)	spuG	+	Homology from 25780925
rpoD (AL347_20760)	rpoD	spuF (AL347_19295)	spuF	+	Homology from 25780925
rpoD (AL347_20760)	rpoD	spuE (AL347_19290)	spuE	+	Homology from 25780925
rpoD (AL347_20760)	rpoD	spuC (AL347_19280)	spuC	+	Homology from 25780925
rpoD (AL347_20760)	rpoD	spuB (AL347_19275)	spuB	+	Homology from 25780925
rpoD (AL347_20760)	rpoD	spuA (AL347_19270)	spuA	+	Homology from 25780925
rpoD (AL347_20760)	rpoD	spuI (AL347_19265)	spuI	+	Homology from 25780925
rpoD (AL347_20760)	rpoD	PA0286 (AL347_19215)	PA0286 (desA)	+	Homology from 25780925
rpoD (AL347_20760)	rpoD	PA0285 (AL347_19210)	PA0285	+	Homology from 25780925
rpoD (AL347_20760)	rpoD	PA0275 (AL347_19160)	PA0275	+	Homology from 25780925
rpoD (AL347_20760)	rpoD	PA0271 (AL347_19140)	PA0271	+	Homology from 25780925
rpoD (AL347_20760)	rpoD	PA0270 (AL347_19135)	PA0270	+	Homology from 25780925
rpoD (AL347_20760)	rpoD	PA0269 (AL347_19130)	PA0269	+	Homology from 25780925
rpoD (AL347_20760)	rpoD	PA0264 (AL347_19095)	PA0264	+	Homology from 25780925
rpoD (AL347_20760)	rpoD	PA0259 (AL347_19065)	PA0259 (tla3, tli3)	+	Homology from 25780925
rpoD (AL347_20760)	rpoD	PA0250 (AL347_19020)	PA0250	+	Homology from 25780925
rpoD (AL347_20760)	rpoD	PA0221 (AL347_18875)	PA0221	+	Homology from 25780925
rpoD (AL347_20760)	rpoD	PA0220 (AL347_18870)	PA0220	+	Homology from 25780925
rpoD (AL347_20760)	rpoD	PA0219 (AL347_18865)	PA0219	+	Homology from 25780925
rpoD (AL347_20760)	rpoD	PA0217 (AL347_18850)	PA0217	+	Homology from 25780925
rpoD (AL347_20760)	rpoD	PA0201 (AL347_18800)	PA0201	+	Homology from 25780925
rpoD (AL347_20760)	rpoD	PA0200 (AL347_18795)	PA0200	+	Homology from 25780925
rpoD (AL347_20760)	rpoD	PA0194 (AL347_18760)	PA0194	+	Homology from 25780925
rpoD (AL347_20760)	rpoD	PA0193 (AL347_18755)	PA0193	+	Homology from 25780925
rpoD (AL347_20760)	rpoD	PA0192 (AL347_18750)	PA0192	+	Homology from 25780925
rpoD (AL347_20760)	rpoD	PA0191 (AL347_18740)	PA0191	+	Homology from 25780925
rpoD (AL347_20760)	rpoD	atsR (AL347_18715)	atsR	+	Homology from 25780925
rpoD (AL347_20760)	rpoD	PA0182 (AL347_18695)	PA0182	+	Homology from 25780925
rpoD (AL347_20760)	rpoD	PA0139 (AL347_18475)	PA0139 (ahpC)	+	Homology from 25780925
rpoD (AL347_20760)	rpoD	PA0114 (AL347_18345)	PA0114 (senC)	+	Homology from 25780925
rpoD (AL347_20760)	rpoD	PA0113 (AL347_18340)	PA0113	+	Homology from 25780925
rpoD (AL347_20760)	rpoD	PA0112 (AL347_18335)	PA0112	+	Homology from 25780925
rpoD (AL347_20760)	rpoD	PA0068 (AL347_18110)	PA0068	+	Homology from 25780925
rpoD (AL347_20760)	rpoD	PA0067 (AL347_18105)	PA0067 (prlC, opdA)	+	Homology from 25780925
rpoD (AL347_20760)	rpoD	phzH (AL347_18030)	phzH	+	Homology from 25780925
rpoD (AL347_20760)	rpoD	PA0019 (AL347_17845)	PA0019 (def)	+	Homology from 25780925

rpoD (AL347_20760)	rpoD	PA0004 (AL347_17745)	PA0004 (gyrB)	+	Homology from 25780925
rpoD (AL347_20760)	rpoD	PA0003 (AL347_17740)	PA0003 (recF)	+	Homology from 25780925
rpoD (AL347_20760)	rpoD	PA0002 (AL347_17735)	PA0002 (dnaN)	+	Homology from 25780925
rpoD (AL347_20760)	rpoD	dnaA (AL347_17730)	dnaA	+	Homology from 25780925
rpoD (AL347_20760)	rpoD	sigX (AL347_03799)	sigX	?	Homology from 25780925, 29729420
rpoD (AL347_20760)	rpoD	PA1698 (AL347_04210)	PA1698 (popN)	+	Homology from 25780925, 29729420
rpoD (AL347_20760)	rpoD	aotJ (AL347_09055)	aotJ	+	27242034*, 18974177*, 22587778
rpoD (AL347_20760)	rpoD	fleQ (AL347_07690)	fleQ	+	27242034*, 18974177*, 22587778
rpoD (AL347_20760)	rpoD	ptxR (AL347_33740)	ptxR	+	27242034*, 18974177*, 22587778
rpoD (AL347_20760)	rpoD	rpoS (AL347_24665)	rpoS	?	27242034*, 18974177*, 22587778
rpoH (AL347_19690)	rpoH	nrdJb (AL347_17300)	nrdJb	+	Homology from 25780925
rpoH (AL347_19690)	rpoH	kinB (AL347_17240)	kinB	+	Homology from 25780925
rpoH (AL347_19690)	rpoH	PA5446 (AL347_17040)	PA5446	+	Homology from 25780925
rpoH (AL347_19690)	rpoH	PA5412 (AL347_16850)	PA5412	+	Homology from 25780925
rpoH (AL347_19690)	rpoH	cdhB (AL347_16715)	cdhB	+	Homology from 25780925
rpoH (AL347_19690)	rpoH	PA5383 (AL347_16705)	PA5383	+	Homology from 25780925
rpoH (AL347_19690)	rpoH	PA5243 (AL347_15955)	PA5243 (hemB)	+	Homology from 25780925
rpoH (AL347_19690)	rpoH	ppK (AL347_15950)	ppK	+	Homology from 25780925
rpoH (AL347_19690)	rpoH	PA5203 (AL347_15750)	PA5203 (gshA)	+	Homology from 25780925
rpoH (AL347_19690)	rpoH	PA5183 (AL347_15640)	PA5183	+	Homology from 25780925
rpoH (AL347_19690)	rpoH	emrB	emrB	+	Homology from 25780925
rpoH (AL347_19690)	rpoH	emrA (AL347_15515)	emrA	+	Homology from 25780925
rpoH (AL347_19690)	rpoH	PA5158 (AL347_15510)	PA5158	+	Homology from 25780925
rpoH (AL347_19690)	rpoH	PA5157 (AL347_15505)	PA5157	+	Homology from 25780925
rpoH (AL347_19690)	rpoH	PA5055 (AL347_14920)	PA5055	+	Homology from 25780925
rpoH (AL347_19690)	rpoH	PA5054 (AL347_14915)	PA5054 (hslU)	+	Homology from 25780925
rpoH (AL347_19690)	rpoH	PA5053 (AL347_14910)	PA5053 (hslV)	+	Homology from 25780925
rpoH (AL347_19690)	rpoH	pilM (AL347_14865)	pilM	+	Homology from 25780925
rpoH (AL347_19690)	rpoH	pilN (AL347_14860)	pilN	+	Homology from 25780925
rpoH (AL347_19690)	rpoH	PA5042 (AL347_14855)	PA5042 (pilO)	+	Homology from 25780925
rpoH (AL347_19690)	rpoH	pilP (AL347_14850)	pilP	+	Homology from 25780925
rpoH (AL347_19690)	rpoH	PA5040 (AL347_14845)	PA5040 (pilQ)	+	Homology from 25780925
rpoH (AL347_19690)	rpoH	PA5030 (AL347_14795)	PA5030	+	Homology from 25780925
rpoH (AL347_19690)	rpoH	aceE (AL347_14705)	aceE	+	Homology from 25780925
rpoH (AL347_19690)	rpoH	hfq (AL347_14340)	hfq	+	Homology from 25780925
rpoH (AL347_19690)	rpoH	PA4943 (AL347_14335)	PA4943	+	Homology from 25780925
rpoH (AL347_19690)	rpoH	PA4909 (AL347_14155)	PA4909	+	Homology from 25780925
rpoH (AL347_19690)	rpoH	PA4881 (AL347_14015)	PA4881	+	Homology from 25780925

rpoH (AL347_19690)	rpoH	PA4870 (AL347_13960)	PA4870	+	Homology from 25780925
rpoH (AL347_19690)	rpoH	PA4762 (AL347_13400)	PA4762 (grpE)	+	Homology from 25780925
rpoH (AL347_19690)	rpoH	PA4761 (AL347_13395)	PA4761 (dnaK)	+	Homology from 25780925
rpoH (AL347_19690)	rpoH	PA4760 (AL347_13390)	PA4760 (dnaJ)	+	Homology from 25780925
rpoH (AL347_19690)	rpoH	PA4759 (AL347_13385)	PA4759 (dapB)	+	Homology from 25780925
rpoH (AL347_19690)	rpoH	PA4751 (hflB)	PA4751 (hflB)	+	Homology from 25780925
rpoH (AL347_19690)	rpoH	PA4750 (AL347_13340)	PA4750 (folP, dhpS)	+	Homology from 25780925
rpoH (AL347_19690)	rpoH	PA4749 (AL347_13335)	PA4749 (glmM, yhbF, mrsA)	+	Homology from 25780925
rpoH (AL347_19690)	rpoH	PA4748 (AL347_13330)	PA4748 (tpiA)	+	Homology from 25780925
rpoH (AL347_19690)	rpoH	PA4739 (AL347_13270)	PA4739	+	Homology from 25780925
rpoH (AL347_19690)	rpoH	PA4738 (AL347_13265)	PA4738	+	Homology from 25780925
rpoH (AL347_19690)	rpoH	PA4728 (AL347_13215)	PA4728 (folK)	+	Homology from 25780925
rpoH (AL347_19690)	rpoH	PA4727 (AL347_13210)	PA4727 (pcnB)	+	Homology from 25780925
rpoH (AL347_19690)	rpoH	PA4674 (AL347_12900)	PA4674	+	Homology from 25780925
rpoH (AL347_19690)	rpoH	PA4613 (AL347_12560)	PA4613 (katB)	+	Homology from 25780925
rpoH (AL347_19690)	rpoH	PA4612 (AL347_12555)	PA4612	+	Homology from 25780925
rpoH (AL347_19690)	rpoH	PA4581 (AL347_12390)	PA4581 (rtcR)	+	Homology from 25780925
rpoH (AL347_19690)	rpoH	PA4542 (AL347_12190)	PA4542 (clpB)	+	Homology from 25780925
rpoH (AL347_19690)	rpoH	dppA3 (AL347_11365)	dppA3	+	Homology from 25780925
rpoH (AL347_19690)	rpoH	PA4472 (AL347_11225)	PA4472 (pmbA, tldE)	+	Homology from 25780925
rpoH (AL347_19690)	rpoH	PA4443 (AL347_11080)	PA4443 (cysD)	+	Homology from 25780925
rpoH (AL347_19690)	rpoH	PA4442 (AL347_11075)	PA4442 (cysN)	+	Homology from 25780925
rpoH (AL347_19690)	rpoH	PA4433 (AL347_11030)	PA4433 (rplM)	+	Homology from 25780925
rpoH (AL347_19690)	rpoH	PA4432 (AL347_11025)	PA4432 (rpsI)	+	Homology from 25780925
rpoH (AL347_19690)	rpoH	PA4387 (AL347_10790)	PA4387	+	Homology from 25780925
rpoH (AL347_19690)	rpoH	PA4386 (AL347_10785)	PA4386 (groES, mopB)	+	Homology from 25780925
rpoH (AL347_19690)	rpoH	PA4385 (AL347_10780)	PA4385 (groEL, mopA)	+	Homology from 25780925
rpoH (AL347_19690)	rpoH	pctC (AL347_10370)	pctC	+	Homology from 25780925
rpoH (AL347_19690)	rpoH	rcpC (AL347_10360)	rcpC	+	Homology from 25780925
rpoH (AL347_19690)	rpoH	rcpA (AL347_10355)	rcpA	+	Homology from 25780925
rpoH (AL347_19690)	rpoH	tadA (AL347_10345)	tadA	+	Homology from 25780925
rpoH (AL347_19690)	rpoH	tadB (AL347_10340)	tadB	+	Homology from 25780925
rpoH (AL347_19690)	rpoH	tadZ (AL347_10350)	tadZ	+	Homology from 25780925
rpoH (AL347_19690)	rpoH	tadD (AL347_10330)	tadD	+	Homology from 25780925
rpoH (AL347_19690)	rpoH	mexI (AL347_21645)	mexI	+	Homology from 25780925
rpoH (AL347_19690)	rpoH	PA4188 (AL347_21740)	PA4188	+	Homology from 25780925
rpoH (AL347_19690)	rpoH	cupB5 (AL347_22295)	cupB5	+	Homology from 25780925
rpoH (AL347_19690)	rpoH	PA4079 (AL347_22310)	PA4079	+	Homology from 25780925

rpoH (AL347_19690)	rpoH	mucE (AL347_22545)	mucE	+	Homology from 25780925
rpoH (AL347_19690)	rpoH	PA3931 (AL347_23060)	PA3931	+	Homology from 25780925
rpoH (AL347_19690)	rpoH	PA3782 (AL347_23845)	PA3782	+	Homology from 25780925
rpoH (AL347_19690)	rpoH	PA3733 (AL347_24110)	PA3733	+	Homology from 25780925
rpoH (AL347_19690)	rpoH	PA3731 (AL347_24120)	PA3731	+	Homology from 25780925
rpoH (AL347_19690)	rpoH	alg8 (AL347_25105)	alg8	+	Homology from 25780925
rpoH (AL347_19690)	rpoH	PA3445 (AL347_25495)	PA3445	+	Homology from 25780925
rpoH (AL347_19690)	rpoH	PA3444 (AL347_25500)	PA3444	+	Homology from 25780925
rpoH (AL347_19690)	rpoH	PA3443 (ssuC)	PA3443	+	Homology from 25780925
rpoH (AL347_19690)	rpoH	PA3442 (AL347_25510)	PA3442	+	Homology from 25780925
rpoH (AL347_19690)	rpoH	PA3441 (AL347_25515)	PA3441	+	Homology from 25780925
rpoH (AL347_19690)	rpoH	PA3371 (AL347_25875)	PA3371	+	Homology from 25780925
rpoH (AL347_19690)	rpoH	oprP (AL347_26390)	oprP	+	Homology from 25780925
rpoH (AL347_19690)	rpoH	PA3226 (AL347_00390)	PA3226	+	Homology from 25780925
rpoH (AL347_19690)	rpoH	PA3225 (AL347_00395)	PA3225	+	Homology from 25780925
rpoH (AL347_19690)	rpoH	PA3162 (AL347_27010)	PA3162 (rpsA)	+	Homology from 25780925
rpoH (AL347_19690)	rpoH	PA3133 (AL347_27175)	PA3133 (sawR)	+	Homology from 25780925
rpoH (AL347_19690)	rpoH	PA3132 (AL347_27180)	PA3132	+	Homology from 25780925
rpoH (AL347_19690)	rpoH	PA3126 (AL347_27210)	PA3126 (ibpA, hslT)	+	Homology from 25780925
rpoH (AL347_19690)	rpoH	acpP (AL347_28040)	acpP	+	Homology from 25780925
rpoH (AL347_19690)	rpoH	atuC (AL347_28445)	atuC	+	Homology from 25780925
rpoH (AL347_19690)	rpoH	PA2849 (AL347_28645)	PA2849 (ohrR)	+	Homology from 25780925
rpoH (AL347_19690)	rpoH	PA2829 (AL347_28745)	PA2829	+	Homology from 25780925
rpoH (AL347_19690)	rpoH	PA2828 (AL347_28750)	PA2828	+	Homology from 25780925
rpoH (AL347_19690)	rpoH	PA2815 (fadE)	PA2815	+	Homology from 25780925
rpoH (AL347_19690)	rpoH	PA2764 (AL347_29340)	PA2764	+	Homology from 25780925
rpoH (AL347_19690)	rpoH	PA2701 (AL347_29905)	PA2701	+	Homology from 25780925
rpoH (AL347_19690)	rpoH	PA2700 (AL347_29910)	PA2700 (opdB)	+	Homology from 25780925
rpoH (AL347_19690)	rpoH	PA2699 (AL347_29915)	PA2699	+	Homology from 25780925
rpoH (AL347_19690)	rpoH	PA2698 (AL347_29920)	PA2698	+	Homology from 25780925
rpoH (AL347_19690)	rpoH	PA2697 (AL347_29925)	PA2697	+	Homology from 25780925
rpoH (AL347_19690)	rpoH	PA2674 (AL347_30040)	PA2674	+	Homology from 25780925
rpoH (AL347_19690)	rpoH	fhp (AL347_30090)	fhp	+	Homology from 25780925
rpoH (AL347_19690)	rpoH	ppyR (AL347_30095)	ppyR	+	Homology from 25780925
rpoH (AL347_19690)	rpoH	PA2662 (AL347_30100)	PA2662	+	Homology from 25780925
rpoH (AL347_19690)	rpoH	PA2649 (AL347_30175)	PA2649 (nuoN)	+	Homology from 25780925
rpoH (AL347_19690)	rpoH	PA2648 (AL347_30180)	PA2648 (nuoM)	+	Homology from 25780925
rpoH (AL347_19690)	rpoH	PA2647 (AL347_30185)	PA2647 (nuoL)	+	Homology from 25780925

rpoH (AL347_19690)	rpoH	PA2646 (AL347_30190)	PA2646 (nuoK)	+	Homology from 25780925
rpoH (AL347_19690)	rpoH	PA2645 (AL347_30195)	PA2645 (nuoJ)	+	Homology from 25780925
rpoH (AL347_19690)	rpoH	PA2644 (AL347_30200)	PA2644 (nuoI)	+	Homology from 25780925
rpoH (AL347_19690)	rpoH	PA2643 (AL347_30205)	PA2643 (nuoH)	+	Homology from 25780925
rpoH (AL347_19690)	rpoH	PA2642 (AL347_30210)	PA2642 (nuoG)	+	Homology from 25780925
rpoH (AL347_19690)	rpoH	PA2641 (AL347_30215)	PA2641 (nuoF)	+	Homology from 25780925
rpoH (AL347_19690)	rpoH	PA2640 (AL347_30220)	PA2640 (nuoE)	+	Homology from 25780925
rpoH (AL347_19690)	rpoH	PA2639 (AL347_30225)	PA2639 (nuoD)	+	Homology from 25780925
rpoH (AL347_19690)	rpoH	PA2638 (AL347_30230)	PA2638 (nuoB)	+	Homology from 25780925
rpoH (AL347_19690)	rpoH	nuoA (AL347_30235)	nuoA	+	Homology from 25780925
rpoH (AL347_19690)	rpoH	PA2600 (AL347_30430)	PA2600	+	Homology from 25780925
rpoH (AL347_19690)	rpoH	PA2599 (AL347_30435)	PA2599	+	Homology from 25780925
rpoH (AL347_19690)	rpoH	PA2598 (AL347_30440)	PA2598	+	Homology from 25780925
rpoH (AL347_19690)	rpoH	PA2597 (AL347_30445)	PA2597	+	Homology from 25780925
rpoH (AL347_19690)	rpoH	PA2594 (AL347_30460)	PA2594	+	Homology from 25780925
rpoH (AL347_19690)	rpoH	PA2569 (AL347_31785)	PA2569	+	Homology from 25780925
rpoH (AL347_19690)	rpoH	PA2416 (AL347_32945)	PA2416 (treA)	+	Homology from 25780925
rpoH (AL347_19690)	rpoH	PA2368 (AL347_33180)	PA2368 (hsiF3)	+	Homology from 25780925
rpoH (AL347_19690)	rpoH	PA2327 (AL347_33390)	PA2327	+	Homology from 25780925
rpoH (AL347_19690)	rpoH	ptxR (AL347_33740)	ptxR	+	Homology from 25780925
rpoH (AL347_19690)	rpoH	qsrO (AL347_33910)	qsrO	+	Homology from 25780925
rpoH (AL347_19690)	rpoH	PA2204 (AL347_01530)	PA2204	+	Homology from 25780925
rpoH (AL347_19690)	rpoH	PA2198 (AL347_01560)	PA2198	+	Homology from 25780925
rpoH (AL347_19690)	rpoH	PA2165 (AL347_01745)	PA2165	+	Homology from 25780925
rpoH (AL347_19690)	rpoH	PA2164 (AL347_01750)	PA2164	+	Homology from 25780925
rpoH (AL347_19690)	rpoH	PA2163 (AL347_01755)	PA2163	+	Homology from 25780925
rpoH (AL347_19690)	rpoH	PA2162 (AL347_01760)	PA2162	+	Homology from 25780925
rpoH (AL347_19690)	rpoH	PA2161 (AL347_01765)	PA2161	+	Homology from 25780925
rpoH (AL347_19690)	rpoH	PA2160 (AL347_01770)	PA2160	+	Homology from 25780925
rpoH (AL347_19690)	rpoH	PA2147 (AL347_01845)	PA2147 (katE)	+	Homology from 25780925
rpoH (AL347_19690)	rpoH	PA2086 (AL347_02150)	PA2086	+	Homology from 25780925
rpoH (AL347_19690)	rpoH	PA2070 (AL347_02250)	PA2070	+	Homology from 25780925
rpoH (AL347_19690)	rpoH	PA2029 (AL347_02460)	PA2029	+	Homology from 25780925
rpoH (AL347_19690)	rpoH	PA1946 (AL347_02885)	PA1946	+	Homology from 25780925
rpoH (AL347_19690)	rpoH	PA1921 (AL347_02995)	PA1921	+	Homology from 25780925
rpoH (AL347_19690)	rpoH	lon (AL347_03630)	lon	+	Homology from 25780925
rpoH (AL347_19690)	rpoH	clpX (AL347_03635)	clpX	+	Homology from 25780925
rpoH (AL347_19690)	rpoH	PA1787 (AL347_03745)	PA1787 (acnB)	+	Homology from 25780925

rpoH (AL347_19690)	rpoH	PA1742 (AL347_03985)	PA1742 (pauD2)	+	Homology from 25780925
rpoH (AL347_19690)	rpoH	PA1740 (AL347_03995)	PA1740	+	Homology from 25780925
rpoH (AL347_19690)	rpoH	PA1739 (AL347_04000)	PA1739	+	Homology from 25780925
rpoH (AL347_19690)	rpoH	PA1631 (AL347_04565)	PA1631	+	Homology from 25780925
rpoH (AL347_19690)	rpoH	PA1630 (AL347_04570)	PA1630	+	Homology from 25780925
rpoH (AL347_19690)	rpoH	PA1597 (AL347_04740)	PA1597	+	Homology from 25780925
rpoH (AL347_19690)	rpoH	PA1596 (AL347_04745)	PA1596 (htpG)	+	Homology from 25780925
rpoH (AL347_19690)	rpoH	PA1569 (AL347_04890)	PA1569	+	Homology from 25780925
rpoH (AL347_19690)	rpoH	PA1568 (AL347_04895)	PA1568	+	Homology from 25780925
rpoH (AL347_19690)	rpoH	PA1567 (AL347_04900)	PA1567	+	Homology from 25780925
rpoH (AL347_19690)	rpoH	PA1516 (AL347_05180)	PA1516	+	Homology from 25780925
rpoH (AL347_19690)	rpoH	PA1488 (AL347_05320)	PA1488	+	Homology from 25780925
rpoH (AL347_19690)	rpoH	PA1487 (AL347_05325)	PA1487	+	Homology from 25780925
rpoH (AL347_19690)	rpoH	lasI (AL347_05605)	lasI	+	Homology from 25780925
rpoH (AL347_19690)	rpoH	PA1423 (AL347_05700)	PA1423 (bdlA)	+	Homology from 25780925
rpoH (AL347_19690)	rpoH	PA1343 (AL347_06380)	PA1343	+	Homology from 25780925
rpoH (AL347_19690)	rpoH	PA1324 (AL347_06485)	PA1324	+	Homology from 25780925
rpoH (AL347_19690)	rpoH	PA1311 (AL347_06555)	PA1311 (phnX)	+	Homology from 25780925
rpoH (AL347_19690)	rpoH	PA1221 (AL347_07025)	PA1221	+	Homology from 25780925
rpoH (AL347_19690)	rpoH	PA1220 (AL347_07030)	PA1220	+	Homology from 25780925
rpoH (AL347_19690)	rpoH	PA1219 (AL347_07035)	PA1219	+	Homology from 25780925
rpoH (AL347_19690)	rpoH	PA1218 (AL347_07040)	PA1218	+	Homology from 25780925
rpoH (AL347_19690)	rpoH	PA1217 (AL347_07045)	PA1217	+	Homology from 25780925
rpoH (AL347_19690)	rpoH	PA1216 (AL347_07050)	PA1216	+	Homology from 25780925
rpoH (AL347_19690)	rpoH	cupC3 (AL347_08235)	cupC3	+	Homology from 25780925
rpoH (AL347_19690)	rpoH	PA0920 (AL347_08625)	PA0920	+	Homology from 25780925
rpoH (AL347_19690)	rpoH	PA0919 (AL347_08630)	PA0919	+	Homology from 25780925
rpoH (AL347_19690)	rpoH	asrA (AL347_09620)	asrA	+	Homology from 25780925
rpoH (AL347_19690)	rpoH	PA0574 (AL347_20710)	PA0574	+	Homology from 25780925
rpoH (AL347_19690)	rpoH	dht (AL347_20030)	dht	+	Homology from 25780925
rpoH (AL347_19690)	rpoH	PA0440 (AL347_20025)	PA0440	+	Homology from 25780925
rpoH (AL347_19690)	rpoH	PA0439 (AL347_20020)	PA0439	+	Homology from 25780925
rpoH (AL347_19690)	rpoH	rpoH (AL347_19690)	rpoH	?	Homology from 25780925
rpoH (AL347_19690)	rpoH	PA0283 (AL347_19200)	PA0283 (sbp)	+	Homology from 25780925
rpoH (AL347_19690)	rpoH	PA0254 (AL347_19040)	PA0254 (hudA, ubiD)	+	Homology from 25780925
rpoH (AL347_19690)	rpoH	PA0253 (AL347_19035)	PA0253 (hudR)	+	Homology from 25780925
rpoH (AL347_19690)	rpoH	PA0201 (AL347_18800)	PA0201	+	Homology from 25780925
rpoH (AL347_19690)	rpoH	PA0200 (AL347_18795)	PA0200	+	Homology from 25780925

rpoH (AL347_19690)	rpoH	PA0123 (AL347_18390)	PA0123	+	Homology from 25780925
rpoH (AL347_19690)	rpoH	PA0114 (AL347_18345)	PA0114 (senC)	+	Homology from 25780925
rpoH (AL347_19690)	rpoH	PA0113 (AL347_18340)	PA0113	+	Homology from 25780925
rpoH (AL347_19690)	rpoH	PA0112 (AL347_18335)	PA0112	+	Homology from 25780925
rpoN (AL347_11175)	rpoN	proC (AL347_19780)	proC	+	8479442
rpoN (AL347_11175)	rpoN	rhlI (AL347_25330)	rhlI	-	12644493
rpoN (AL347_11175)	rpoN	flhB (AL347_05520)	flhB	+	14617143
rpoN (AL347_11175)	rpoN	fleN (AL347_05495)	fleN	+	14617143
rpoN (AL347_11175)	rpoN	sadB (AL347_16485)	sadB	-	15231779
rpoN (AL347_11175)	rpoN	fhpR (AL347_30085)	fhpR	+	15937158
rpoN (AL347_11175)	rpoN	alg8 (AL347_25105)	alg8	+	22210761
rpoN (AL347_11175)	rpoN	alg44 (AL347_25100)	alg44	+	22210761
rpoN (AL347_11175)	rpoN	algK (AL347_25095)	algK	+	22210761
rpoN (AL347_11175)	rpoN	algE (AL347_25090)	algE	+	22210761
rpoN (AL347_11175)	rpoN	algG (AL347_25085)	algG	+	22210761
rpoN (AL347_11175)	rpoN	algX (AL347_25080)	algX	+	22210761
rpoN (AL347_11175)	rpoN	algL (AL347_25075)	algL	+	22210761
rpoN (AL347_11175)	rpoN	algI (AL347_25065)	algI	+	22210761
rpoN (AL347_11175)	rpoN	algJ (AL347_25060)	algJ	+	22210761
rpoN (AL347_11175)	rpoN	algF (AL347_25055)	algF	+	22210761
rpoN (AL347_11175)	rpoN	algA (AL347_25050)	algA	+	22210761
rpoN (AL347_11175)	rpoN	pqsA (AL347_08225)	pqsA	+	29760208
rpoN (AL347_11175)	rpoN	pqsB (AL347_08220)	pqsB	+	29760208
rpoN (AL347_11175)	rpoN	pqsC (AL347_08215)	pqsC	+	29760208
rpoN (AL347_11175)	rpoN	pqsD (AL347_08210)	pqsD	+	29760208
rpoN (AL347_11175)	rpoN	pqsE (AL347_08205)	pqsE	+	29760208
rpoN (AL347_11175)	rpoN	mvfR (AL347_08190)	mvfR	+	29760208
rpoN (AL347_11175)	rpoN	ddaR (AL347_07150)	ddaR	+	29760208
rpoN (AL347_11175)	rpoN	lasR (AL347_05615)	lasR	+	29760208
rpoN (AL347_11175)	rpoN	rsaL (AL347_05610)	rsaL	+	29760208
rpoN (AL347_11175)	rpoN	lasI (AL347_05605)	lasI	+	29760208
rpoN (AL347_11175)	rpoN	nirD (AL347_03780)	nirD	+	29760208
rpoN (AL347_11175)	rpoN	nirB (AL347_03775)	nirB	+	29760208
rpoN (AL347_11175)	rpoN	nasA (AL347_03765)	nasA	+	29760208
rpoN (AL347_11175)	rpoN	nasT (AL347_03755)	nasT	+	29760208
rpoN (AL347_11175)	rpoN	nasS (AL347_03750)	nasS	+	29760208
rpoN (AL347_11175)	rpoN	hbcR (AL347_02585)	hbcR	+	29760208
rpoN (AL347_11175)	rpoN	gcsR (AL347_32765)	gcsR	+	29760208

rpoN (AL347_11175)	rpoN	pqsH (AL347_30495)	pqsH	+	29760208
rpoN (AL347_11175)	rpoN	nosR (AL347_25775)	nosR	+	29760208
rpoN (AL347_11175)	rpoN	nosZ (AL347_25770)	nosZ	+	29760208
rpoN (AL347_11175)	rpoN	nosD (AL347_25765)	nosD	+	29760208
rpoN (AL347_11175)	rpoN	nosF (AL347_25760)	nosF	+	29760208
rpoN (AL347_11175)	rpoN	nosY (AL347_25755)	nosY	+	29760208
rpoN (AL347_11175)	rpoN	nosL (AL347_25750)	nosL	+	29760208
rpoN (AL347_11175)	rpoN	hcpB (AL347_19085)	hcpB	+	29760208
rpoN (AL347_11175)	rpoN	amtB (AL347_16190)	amtB	+	29760208
rpoN (AL347_11175)	rpoN	glnK (AL347_16195)	glnK	+	29760208
rpoN (AL347_11175)	rpoN	algB (AL347_17235)	algB	+	29760208
rpoN (AL347_11175)	rpoN	mifR (AL347_17375)	mifR	+	29760208
rpoN (AL347_11175)	rpoN	atpI (AL347_17625)	atpI	+	Homology from 25780925
rpoN (AL347_11175)	rpoN	PA5560 (AL347_17620)	PA5560 (atpB, papD, uncB)	+	Homology from 25780925
rpoN (AL347_11175)	rpoN	PA5530 (AL347_17470)	PA5530	+	Homology from 25780925
rpoN (AL347_11175)	rpoN	PA5516 (AL347_17400)	PA5516 (pdxY)	+	Homology from 25780925
rpoN (AL347_11175)	rpoN	PA5505 (AL347_17345)	PA5505	+	Homology from 25780925
rpoN (AL347_11175)	rpoN	PA5504 (AL347_17340)	PA5504	+	Homology from 25780925
rpoN (AL347_11175)	rpoN	PA5503 (AL347_17335)	PA5503	+	Homology from 25780925
rpoN (AL347_11175)	rpoN	kinB (AL347_17240)	kinB	+	Homology from 25780925
rpoN (AL347_11175)	rpoN	PA5482 (AL347_17230)	PA5482	+	Homology from 25780925
rpoN (AL347_11175)	rpoN	PA5481 (AL347_17225)	PA5481	+	Homology from 25780925
rpoN (AL347_11175)	rpoN	PA5460 (AL347_17110)	PA5460	+	Homology from 25780925
rpoN (AL347_11175)	rpoN	yegQ (AL347_17005)	yegQ	+	Homology from 25780925
rpoN (AL347_11175)	rpoN	gbcB (AL347_16845)	gbcB	+	Homology from 25780925
rpoN (AL347_11175)	rpoN	dgcB (AL347_16785)	dgcB	+	Homology from 25780925
rpoN (AL347_11175)	rpoN	dgcA (AL347_16780)	dgcA	+	Homology from 25780925
rpoN (AL347_11175)	rpoN	PA5397 (AL347_16775)	PA5397	+	Homology from 25780925
rpoN (AL347_11175)	rpoN	gbdR (AL347_16690)	gbdR	+	Homology from 25780925
rpoN (AL347_11175)	rpoN	PA5293 (AL347_16220)	PA5293	+	Homology from 25780925
rpoN (AL347_11175)	rpoN	PA5291 (AL347_16210)	PA5291 (betT2)	+	Homology from 25780925
rpoN (AL347_11175)	rpoN	PA5286 (AL347_16185)	PA5286	+	Homology from 25780925
rpoN (AL347_11175)	rpoN	PA5246 (AL347_15970)	PA5246	+	Homology from 25780925
rpoN (AL347_11175)	rpoN	PA5245 (AL347_15965)	PA5245	+	Homology from 25780925
rpoN (AL347_11175)	rpoN	amgR (AL347_15735)	amgR	+	Homology from 25780925
rpoN (AL347_11175)	rpoN	PA5199 (AL347_15730)	PA5199 (amgS)	+	Homology from 25780925
rpoN (AL347_11175)	rpoN	PA5183 (AL347_15640)	PA5183	+	Homology from 25780925
rpoN (AL347_11175)	rpoN	arcC (AL347_15590)	arcC	+	Homology from 25780925

rpoN (AL347_11175)	rpoN	dctP (AL347_09075)	dctP	+	Homology from 25780925
rpoN (AL347_11175)	rpoN	PA5153 (AL347_15485)	PA5153	+	Homology from 25780925
rpoN (AL347_11175)	rpoN	PA5139 (AL347_15350)	PA5139	+	Homology from 25780925
rpoN (AL347_11175)	rpoN	PA5138 (AL347_15345)	PA5138	+	Homology from 25780925
rpoN (AL347_11175)	rpoN	PA5131 (AL347_15310)	PA5131 (pgm, yibO)	+	Homology from 25780925
rpoN (AL347_11175)	rpoN	PA5127 (AL347_15290)	PA5127	+	Homology from 25780925
rpoN (AL347_11175)	rpoN	ntrC (AL347_15275)	ntrC	+	Homology from 25780925
rpoN (AL347_11175)	rpoN	ntrB (AL347_15270)	ntrB	+	Homology from 25780925
rpoN (AL347_11175)	rpoN	PA5119 (AL347_15245)	PA5119	+	Homology from 25780925
rpoN (AL347_11175)	rpoN	PA5118 (AL347_15235)	PA5118 (thiI, yajK)	+	Homology from 25780925
rpoN (AL347_11175)	rpoN	PA5117 (AL347_15230)	PA5117 (typA, bipA)	+	Homology from 25780925
rpoN (AL347_11175)	rpoN	hutU (AL347_15145)	hutU	+	Homology from 25780925
rpoN (AL347_11175)	rpoN	hutH (AL347_15135)	hutH	+	Homology from 25780925
rpoN (AL347_11175)	rpoN	PA5076 (AL347_15030)	PA5076	+	Homology from 25780925
rpoN (AL347_11175)	rpoN	PA5075 (AL347_15025)	PA5075	+	Homology from 25780925
rpoN (AL347_11175)	rpoN	PA5074 (AL347_15020)	PA5074	+	Homology from 25780925
rpoN (AL347_11175)	rpoN	PA5072 (AL347_15010)	PA5072 (mcpK)	+	Homology from 25780925
rpoN (AL347_11175)	rpoN	PA5059 (AL347_14945)	PA5059	+	Homology from 25780925
rpoN (AL347_11175)	rpoN	PA5058 (AL347_14940)	PA5058 (phaC2, phaC)	+	Homology from 25780925
rpoN (AL347_11175)	rpoN	PA5057 (AL347_14935)	PA5057 (phaD, phaB)	+	Homology from 25780925
rpoN (AL347_11175)	rpoN	PA5056 (AL347_14930)	PA5056 (phaC1, phaA)	+	Homology from 25780925
rpoN (AL347_11175)	rpoN	PA5055 (AL347_14920)	PA5055	+	Homology from 25780925
rpoN (AL347_11175)	rpoN	PA5054 (AL347_14915)	PA5054 (hslU)	+	Homology from 25780925
rpoN (AL347_11175)	rpoN	PA5053 (AL347_14910)	PA5053 (hslV)	+	Homology from 25780925
rpoN (AL347_11175)	rpoN	gltD (AL347_14820)	gltD	+	Homology from 25780925
rpoN (AL347_11175)	rpoN	PA5034 (AL347_14815)	PA5034 (hemE)	+	Homology from 25780925
rpoN (AL347_11175)	rpoN	PA5023 (AL347_14760)	PA5023	+	Homology from 25780925
rpoN (AL347_11175)	rpoN	PA5022 (AL347_14755)	PA5022	+	Homology from 25780925
rpoN (AL347_11175)	rpoN	PA5021 (AL347_14750)	PA5021	+	Homology from 25780925
rpoN (AL347_11175)	rpoN	PA5001 (AL347_14635)	PA5001 (ssg)	+	Homology from 25780925
rpoN (AL347_11175)	rpoN	PA4935 (AL347_14285)	PA4935 (rpsF)	+	Homology from 25780925
rpoN (AL347_11175)	rpoN	PA4934 (AL347_14280)	PA4934 (rpsR)	+	Homology from 25780925
rpoN (AL347_11175)	rpoN	PA4933 (AL347_14275)	PA4933	+	Homology from 25780925
rpoN (AL347_11175)	rpoN	PA4932 (AL347_14270)	PA4932 (rplI)	+	Homology from 25780925
rpoN (AL347_11175)	rpoN	PA4931 (AL347_14265)	PA4931 (dnaB)	+	Homology from 25780925
rpoN (AL347_11175)	rpoN	alr (AL347_14260)	alr	+	Homology from 25780925
rpoN (AL347_11175)	rpoN	PA4929 (AL347_14255)	PA4929	+	Homology from 25780925
rpoN (AL347_11175)	rpoN	PA4927 (AL347_14245)	PA4927	+	Homology from 25780925

rpoN (AL347_11175)	rpoN	PA4926 (AL347_14240)	PA4926	+	Homology from 25780925
rpoN (AL347_11175)	rpoN	PA4913 (AL347_14175)	PA4913	+	Homology from 25780925
rpoN (AL347_11175)	rpoN	PA4894 (AL347_14080)	PA4894	+	Homology from 25780925
rpoN (AL347_11175)	rpoN	PA4893 (AL347_14075)	PA4893 (ureG)	+	Homology from 25780925
rpoN (AL347_11175)	rpoN	PA4892 (AL347_14070)	PA4892 (ureF)	+	Homology from 25780925
rpoN (AL347_11175)	rpoN	PA4891 (AL347_14065)	PA4891 (ureE)	+	Homology from 25780925
rpoN (AL347_11175)	rpoN	PA4880 (AL347_14010)	PA4880	+	Homology from 25780925
rpoN (AL347_11175)	rpoN	ureC (AL347_13950)	ureC	+	Homology from 25780925
rpoN (AL347_11175)	rpoN	ureB (AL347_13945)	ureB	+	Homology from 25780925
rpoN (AL347_11175)	rpoN	PA4866 (AL347_13940)	PA4866	+	Homology from 25780925
rpoN (AL347_11175)	rpoN	ureA (AL347_13935)	ureA	+	Homology from 25780925
rpoN (AL347_11175)	rpoN	PA4864 (AL347_13930)	PA4864 (ureD)	+	Homology from 25780925
rpoN (AL347_11175)	rpoN	PA4863 (AL347_13925)	PA4863	+	Homology from 25780925
rpoN (AL347_11175)	rpoN	PA4862 (AL347_13920)	PA4862	+	Homology from 25780925
rpoN (AL347_11175)	rpoN	PA4860 (AL347_13910)	PA4860	+	Homology from 25780925
rpoN (AL347_11175)	rpoN	PA4858 (AL347_13900)	PA4858	+	Homology from 25780925
rpoN (AL347_11175)	rpoN	retS (AL347_13890)	retS	+	Homology from 25780925
rpoN (AL347_11175)	rpoN	purD (AL347_13885)	purD	+	Homology from 25780925
rpoN (AL347_11175)	rpoN	PA4854 (AL347_13880)	PA4854 (purH)	+	Homology from 25780925
rpoN (AL347_11175)	rpoN	gcbA (AL347_13825)	gcbA	+	Homology from 25780925
rpoN (AL347_11175)	rpoN	PA4826 (AL347_13735)	PA4826	+	Homology from 25780925
rpoN (AL347_11175)	rpoN	PA4762 (AL347_13400)	PA4762 (grpE)	+	Homology from 25780925
rpoN (AL347_11175)	rpoN	PA4760 (AL347_13390)	PA4760 (dnaJ)	+	Homology from 25780925
rpoN (AL347_11175)	rpoN	PA4759 (AL347_13385)	PA4759 (dapB)	+	Homology from 25780925
rpoN (AL347_11175)	rpoN	carB (AL347_13370)	carB	+	Homology from 25780925
rpoN (AL347_11175)	rpoN	PA4744 (AL347_13300)	PA4744 (infB)	+	Homology from 25780925
rpoN (AL347_11175)	rpoN	PA4743 (AL347_13295)	PA4743 (rbfA)	+	Homology from 25780925
rpoN (AL347_11175)	rpoN	PA4742 (AL347_13290)	PA4742 (truB)	+	Homology from 25780925
rpoN (AL347_11175)	rpoN	PA4739 (AL347_13270)	PA4739	+	Homology from 25780925
rpoN (AL347_11175)	rpoN	PA4738 (AL347_13265)	PA4738	+	Homology from 25780925
rpoN (AL347_11175)	rpoN	PA4727 (AL347_13210)	PA4727 (pcnB)	+	Homology from 25780925
rpoN (AL347_11175)	rpoN	cbrB (AL347_13205)	cbrB	+	Homology from 25780925
rpoN (AL347_11175)	rpoN	PA4686 (AL347_12970)	PA4686	+	Homology from 25780925
rpoN (AL347_11175)	rpoN	PA4685 (AL347_12965)	PA4685	+	Homology from 25780925
rpoN (AL347_11175)	rpoN	PA4684 (AL347_12960)	PA4684	+	Homology from 25780925
rpoN (AL347_11175)	rpoN	PA4683 (AL347_12955)	PA4683	+	Homology from 25780925
rpoN (AL347_11175)	rpoN	PA4682 (AL347_12950)	PA4682	+	Homology from 25780925
rpoN (AL347_11175)	rpoN	PA4681 (AL347_12945)	PA4681	+	Homology from 25780925

rpoN (AL347_11175)	rpoN	PA4680 (AL347_12940)	PA4680	+	Homology from 25780925
rpoN (AL347_11175)	rpoN	PA4673 (AL347_12890)	PA4673	+	Homology from 25780925
rpoN (AL347_11175)	rpoN	PA4672 (AL347_12885)	PA4672	+	Homology from 25780925
rpoN (AL347_11175)	rpoN	PA4669 (AL347_12865)	PA4669 (ipk, ychB)	+	Homology from 25780925
rpoN (AL347_11175)	rpoN	PA4657 (AL347_12805)	PA4657	+	Homology from 25780925
rpoN (AL347_11175)	rpoN	PA4645 (AL347_12745)	PA4645	+	Homology from 25780925
rpoN (AL347_11175)	rpoN	PA4644 (AL347_12740)	PA4644	+	Homology from 25780925
rpoN (AL347_11175)	rpoN	PA4643 (AL347_12735)	PA4643	+	Homology from 25780925
rpoN (AL347_11175)	rpoN	PA4624 (AL347_12630)	PA4624 (cdrB)	+	Homology from 25780925
rpoN (AL347_11175)	rpoN	PA4590 (AL347_12440)	PA4590 (pra)	+	Homology from 25780925
rpoN (AL347_11175)	rpoN	PA4585 (AL347_12415)	PA4585 (rtcA, yhgK)	+	Homology from 25780925
rpoN (AL347_11175)	rpoN	PA4584 (AL347_12410)	PA4584	+	Homology from 25780925
rpoN (AL347_11175)	rpoN	PA4583 (AL347_12405)	PA4583	+	Homology from 25780925
rpoN (AL347_11175)	rpoN	PA4582 (AL347_12400)	PA4582	+	Homology from 25780925
rpoN (AL347_11175)	rpoN	PA4574 (AL347_12355)	PA4574	+	Homology from 25780925
rpoN (AL347_11175)	rpoN	PA4568 (AL347_12325)	PA4568 (rplU)	+	Homology from 25780925
rpoN (AL347_11175)	rpoN	PA4561 (AL347_12290)	PA4561 (ribF)	+	Homology from 25780925
rpoN (AL347_11175)	rpoN	PA4560 (AL347_12285)	PA4560 (ileS)	+	Homology from 25780925
rpoN (AL347_11175)	rpoN	PA4559 (AL347_12280)	PA4559 (lspA)	+	Homology from 25780925
rpoN (AL347_11175)	rpoN	PA4558 (AL347_12275)	PA4558	+	Homology from 25780925
rpoN (AL347_11175)	rpoN	PA4557 (AL347_12270)	PA4557 (lytB)	+	Homology from 25780925
rpoN (AL347_11175)	rpoN	PA4523 (AL347_11485)	PA4523	+	Homology from 25780925
rpoN (AL347_11175)	rpoN	PA4520 (AL347_11470)	PA4520	+	Homology from 25780925
rpoN (AL347_11175)	rpoN	dppF (AL347_11400)	dppF	+	Homology from 25780925
rpoN (AL347_11175)	rpoN	dppD	dppD	+	Homology from 25780925
rpoN (AL347_11175)	rpoN	dppC (AL347_11390)	dppC	+	Homology from 25780925
rpoN (AL347_11175)	rpoN	dppB (AL347_11385)	dppB	+	Homology from 25780925
rpoN (AL347_11175)	rpoN	dppA4 (AL347_11380)	dppA4	+	Homology from 25780925
rpoN (AL347_11175)	rpoN	PA4501 (AL347_11375)	PA4501 (opdD, opdP)	+	Homology from 25780925
rpoN (AL347_11175)	rpoN	PA4497 (AL347_11350)	PA4497 (dppA2)	+	Homology from 25780925
rpoN (AL347_11175)	rpoN	PA4496 (AL347_11345)	PA4496 (dppA1)	+	Homology from 25780925
rpoN (AL347_11175)	rpoN	PA4463 (AL347_11180)	PA4463	+	Homology from 25780925
rpoN (AL347_11175)	rpoN	rpoN (AL347_11175)	rpoN	?	Homology from 25780925
rpoN (AL347_11175)	rpoN	lptB (AL347_11170)	lptB	+	Homology from 25780925
rpoN (AL347_11175)	rpoN	PA4438 (AL347_11055)	PA4438	+	Homology from 25780925
rpoN (AL347_11175)	rpoN	PA4433 (AL347_11030)	PA4433 (rplM)	+	Homology from 25780925
rpoN (AL347_11175)	rpoN	PA4432 (AL347_11025)	PA4432 (rpsI)	+	Homology from 25780925
rpoN (AL347_11175)	rpoN	PA4425 (AL347_10990)	PA4425	+	Homology from 25780925

rpoN (AL347_11175)	rpoN	PA4424 (AL347_10985)	PA4424	+	Homology from 25780925
rpoN (AL347_11175)	rpoN	PA4386 (AL347_10785)	PA4386 (groES, mopB)	+	Homology from 25780925
rpoN (AL347_11175)	rpoN	PA4385 (AL347_10780)	PA4385 (groEL, mopA)	+	Homology from 25780925
rpoN (AL347_11175)	rpoN	icmP (AL347_10700)	icmP	+	Homology from 25780925
rpoN (AL347_11175)	rpoN	PA4368 (AL347_10690)	PA4368	+	Homology from 25780925
rpoN (AL347_11175)	rpoN	PA4345 (AL347_10570)	PA4345	+	Homology from 25780925
rpoN (AL347_11175)	rpoN	PA4341 (AL347_10550)	PA4341	+	Homology from 25780925
rpoN (AL347_11175)	rpoN	PA4310 (AL347_10385)	PA4310 (pctB)	+	Homology from 25780925
rpoN (AL347_11175)	rpoN	flp (AL347_10365)	flp	+	Homology from 25780925
rpoN (AL347_11175)	rpoN	rcpA (AL347_10355)	rcpA	+	Homology from 25780925
rpoN (AL347_11175)	rpoN	tadA (AL347_10345)	tadA	+	Homology from 25780925
rpoN (AL347_11175)	rpoN	tadB (AL347_10340)	tadB	+	Homology from 25780925
rpoN (AL347_11175)	rpoN	tadZ (AL347_10350)	tadZ	+	Homology from 25780925
rpoN (AL347_11175)	rpoN	PA4298 (AL347_10325)	PA4298	+	Homology from 25780925
rpoN (AL347_11175)	rpoN	tadG (AL347_10320)	tadG	+	Homology from 25780925
rpoN (AL347_11175)	rpoN	PA4276 (AL347_21290)	PA4276 (secE, prlG)	+	Homology from 25780925
rpoN (AL347_11175)	rpoN	PA4275 (AL347_21295)	PA4275 (nusG)	+	Homology from 25780925
rpoN (AL347_11175)	rpoN	rplA (AL347_21305)	rplA	+	Homology from 25780925
rpoN (AL347_11175)	rpoN	rplJ (AL347_21310)	rplJ	+	Homology from 25780925
rpoN (AL347_11175)	rpoN	rplL (AL347_21315)	rplL	+	Homology from 25780925
rpoN (AL347_11175)	rpoN	rpoC (AL347_21325)	rpoC	+	Homology from 25780925
rpoN (AL347_11175)	rpoN	rpsL (AL347_21330)	rpsL	+	Homology from 25780925
rpoN (AL347_11175)	rpoN	rpsJ (AL347_21350)	rpsJ	+	Homology from 25780925
rpoN (AL347_11175)	rpoN	rplC (AL347_21355)	rplC	+	Homology from 25780925
rpoN (AL347_11175)	rpoN	PA4262 (AL347_21360)	PA4262 (rplD)	+	Homology from 25780925
rpoN (AL347_11175)	rpoN	PA4261 (AL347_21365)	PA4261 (rplW)	+	Homology from 25780925
rpoN (AL347_11175)	rpoN	PA4260 (AL347_21370)	PA4260 (rplB)	+	Homology from 25780925
rpoN (AL347_11175)	rpoN	PA4259 (AL347_21375)	PA4259 (rpsS)	+	Homology from 25780925
rpoN (AL347_11175)	rpoN	PA4258 (AL347_21380)	PA4258 (rplV)	+	Homology from 25780925
rpoN (AL347_11175)	rpoN	PA4257 (AL347_21385)	PA4257 (rpsC)	+	Homology from 25780925
rpoN (AL347_11175)	rpoN	PA4256 (AL347_21390)	PA4256 (rplP)	+	Homology from 25780925
rpoN (AL347_11175)	rpoN	PA4255 (AL347_21395)	PA4255 (rpmC)	+	Homology from 25780925
rpoN (AL347_11175)	rpoN	PA4254 (AL347_21400)	PA4254 (rpsQ)	+	Homology from 25780925
rpoN (AL347_11175)	rpoN	PA4253 (AL347_21405)	PA4253 (rplN)	+	Homology from 25780925
rpoN (AL347_11175)	rpoN	PA4252 (AL347_21410)	PA4252 (rplX)	+	Homology from 25780925
rpoN (AL347_11175)	rpoN	PA4251 (AL347_21415)	PA4251 (rplE)	+	Homology from 25780925
rpoN (AL347_11175)	rpoN	PA4247 (AL347_21435)	PA4247 (rplR)	+	Homology from 25780925
rpoN (AL347_11175)	rpoN	PA4246 (AL347_21440)	PA4246 (rpsE)	+	Homology from 25780925

rpoN (AL347_11175)	rpoN	PA4245 (AL347_21445)	PA4245 (rpmD)	+	Homology from 25780925
rpoN (AL347_11175)	rpoN	PA4244 (AL347_21450)	PA4244 (rplO)	+	Homology from 25780925
rpoN (AL347_11175)	rpoN	PA4243 (AL347_21455)	PA4243 (secY, prlA)	+	Homology from 25780925
rpoN (AL347_11175)	rpoN	PA4239 (AL347_21475)	PA4239 (rpsD)	+	Homology from 25780925
rpoN (AL347_11175)	rpoN	PA4237 (AL347_21485)	PA4237 (rplQ)	+	Homology from 25780925
rpoN (AL347_11175)	rpoN	mexH (AL347_21650)	mexH	+	Homology from 25780925
rpoN (AL347_11175)	rpoN	nmoA (AL347_21670)	nmoA	+	Homology from 25780925
rpoN (AL347_11175)	rpoN	PA4201 (AL347_21675)	PA4201 (ddlA)	+	Homology from 25780925
rpoN (AL347_11175)	rpoN	PA4173 (AL347_21820)	PA4173	+	Homology from 25780925
rpoN (AL347_11175)	rpoN	PA4172 (AL347_21825)	PA4172	+	Homology from 25780925
rpoN (AL347_11175)	rpoN	PA4171 (AL347_21830)	PA4171	+	Homology from 25780925
rpoN (AL347_11175)	rpoN	PA4070 (AL347_22355)	PA4070	+	Homology from 25780925
rpoN (AL347_11175)	rpoN	PA4069 (AL347_22360)	PA4069	+	Homology from 25780925
rpoN (AL347_11175)	rpoN	PA4068 (AL347_22365)	PA4068	+	Homology from 25780925
rpoN (AL347_11175)	rpoN	PA4046 (AL347_22480)	PA4046	+	Homology from 25780925
rpoN (AL347_11175)	rpoN	PA4045 (AL347_22485)	PA4045	+	Homology from 25780925
rpoN (AL347_11175)	rpoN	PA4030 (AL347_22560)	PA4030	+	Homology from 25780925
rpoN (AL347_11175)	rpoN	PA4025 (AL347_22585)	PA4025 (eutC)	+	Homology from 25780925
rpoN (AL347_11175)	rpoN	PA4024 (AL347_22590)	PA4024 (eutB)	+	Homology from 25780925
rpoN (AL347_11175)	rpoN	PA4023 (AL347_22595)	PA4023 (eat, eutP)	+	Homology from 25780925
rpoN (AL347_11175)	rpoN	PA4022 (AL347_22600)	PA4022 (hdhA, exaC2)	+	Homology from 25780925
rpoN (AL347_11175)	rpoN	PA4021 (AL347_22605)	PA4021 (eatR)	+	Homology from 25780925
rpoN (AL347_11175)	rpoN	PA4015 (AL347_22635)	PA4015	+	Homology from 25780925
rpoN (AL347_11175)	rpoN	PA4013 (AL347_22645)	PA4013	+	Homology from 25780925
rpoN (AL347_11175)	rpoN	PA3983 (AL347_22790)	PA3983	+	Homology from 25780925
rpoN (AL347_11175)	rpoN	PA3954 (AL347_22940)	PA3954 (sfnG)	+	Homology from 25780925
rpoN (AL347_11175)	rpoN	PA3891 (AL347_23275)	PA3891 (opuCA)	+	Homology from 25780925
rpoN (AL347_11175)	rpoN	PA3890 (AL347_23280)	PA3890 (opuCB)	+	Homology from 25780925
rpoN (AL347_11175)	rpoN	PA3889 (AL347_23285)	PA3889 (opuCC)	+	Homology from 25780925
rpoN (AL347_11175)	rpoN	PA3888 (AL347_23290)	PA3888 (opuCD)	+	Homology from 25780925
rpoN (AL347_11175)	rpoN	PA3823 (AL347_23640)	PA3823 (tgt)	+	Homology from 25780925
rpoN (AL347_11175)	rpoN	PA3798 (AL347_23765)	PA3798	+	Homology from 25780925
rpoN (AL347_11175)	rpoN	PA3783 (AL347_23840)	PA3783	+	Homology from 25780925
rpoN (AL347_11175)	rpoN	PA3782 (AL347_23845)	PA3782	+	Homology from 25780925
rpoN (AL347_11175)	rpoN	PA3771 (AL347_23905)	PA3771	+	Homology from 25780925
rpoN (AL347_11175)	rpoN	PA3770 (AL347_23910)	PA3770 (guaB)	+	Homology from 25780925
rpoN (AL347_11175)	rpoN	PA3769 (AL347_23915)	PA3769 (guaA)	+	Homology from 25780925
rpoN (AL347_11175)	rpoN	PA3748 (AL347_24030)	PA3748	+	Homology from 25780925

rpoN (AL347_11175)	rpoN	PA3746 (AL347_24040)	PA3746 (ffh)	+	Homology from 25780925
rpoN (AL347_11175)	rpoN	PA3745 (AL347_24045)	PA3745 (rpsP)	+	Homology from 25780925
rpoN (AL347_11175)	rpoN	PA3744 (AL347_24050)	PA3744 (rimM)	+	Homology from 25780925
rpoN (AL347_11175)	rpoN	PA3743 (AL347_24055)	PA3743 (trmD)	+	Homology from 25780925
rpoN (AL347_11175)	rpoN	PA3742 (AL347_24060)	PA3742 (rplS)	+	Homology from 25780925
rpoN (AL347_11175)	rpoN	nalC (AL347_24170)	nalC	+	Homology from 25780925
rpoN (AL347_11175)	rpoN	armR (AL347_24180)	armR	+	Homology from 25780925
rpoN (AL347_11175)	rpoN	lptF (AL347_24315)	lptF	+	Homology from 25780925
rpoN (AL347_11175)	rpoN	PA3691 (AL347_24320)	PA3691	+	Homology from 25780925
rpoN (AL347_11175)	rpoN	PA3655 (AL347_24500)	PA3655 (tsf)	+	Homology from 25780925
rpoN (AL347_11175)	rpoN	PA3651 (AL347_24520)	PA3651 (cdsA)	+	Homology from 25780925
rpoN (AL347_11175)	rpoN	PA3641 (AL347_24570)	PA3641	+	Homology from 25780925
rpoN (AL347_11175)	rpoN	PA3597 (AL347_24795)	PA3597	+	Homology from 25780925
rpoN (AL347_11175)	rpoN	PA3596 (AL347_24800)	PA3596	+	Homology from 25780925
rpoN (AL347_11175)	rpoN	PA3594 (AL347_24815)	PA3594	+	Homology from 25780925
rpoN (AL347_11175)	rpoN	glpD (AL347_24865)	glpD	+	Homology from 25780925
rpoN (AL347_11175)	rpoN	PA3567 (AL347_24965)	PA3567	+	Homology from 25780925
rpoN (AL347_11175)	rpoN	PA3526 (AL347_25180)	PA3526 (motY)	+	Homology from 25780925
rpoN (AL347_11175)	rpoN	rhlA (AL347_25315)	rhlA	+	Homology from 25780925
rpoN (AL347_11175)	rpoN	rhlB (AL347_25320)	rhlB	+	Homology from 25780925
rpoN (AL347_11175)	rpoN	rhlR (AL347_25325)	rhlR	+	Homology from 25780925
rpoN (AL347_11175)	rpoN	PA3460 (AL347_25415)	PA3460	+	Homology from 25780925
rpoN (AL347_11175)	rpoN	PA3440 (AL347_25525)	PA3440	+	Homology from 25780925
rpoN (AL347_11175)	rpoN	PA3436 (AL347_25545)	PA3436	+	Homology from 25780925
rpoN (AL347_11175)	rpoN	PA3405 (AL347_25700)	PA3405 (hasE)	+	Homology from 25780925
rpoN (AL347_11175)	rpoN	PA3403 (AL347_25715)	PA3403	+	Homology from 25780925
rpoN (AL347_11175)	rpoN	PA3402 (AL347_25720)	PA3402	+	Homology from 25780925
rpoN (AL347_11175)	rpoN	PA3401 (AL347_25725)	PA3401	+	Homology from 25780925
rpoN (AL347_11175)	rpoN	PA3400 (AL347_25730)	PA3400	+	Homology from 25780925
rpoN (AL347_11175)	rpoN	PA3386 (AL347_25800)	PA3386	+	Homology from 25780925
rpoN (AL347_11175)	rpoN	PA3371 (AL347_25875)	PA3371	+	Homology from 25780925
rpoN (AL347_11175)	rpoN	PA3370 (AL347_25880)	PA3370	+	Homology from 25780925
rpoN (AL347_11175)	rpoN	PA3369 (AL347_25885)	PA3369	+	Homology from 25780925
rpoN (AL347_11175)	rpoN	lecB (AL347_25945)	lecB	+	Homology from 25780925
rpoN (AL347_11175)	rpoN	PA3360 (AL347_25950)	PA3360	+	Homology from 25780925
rpoN (AL347_11175)	rpoN	PA3359 (AL347_25955)	PA3359	+	Homology from 25780925
rpoN (AL347_11175)	rpoN	PA3353 (AL347_25985)	PA3353 (flgZ)	+	Homology from 25780925
rpoN (AL347_11175)	rpoN	PA3350 (AL347_26000)	PA3350	+	Homology from 25780925

rpoN (AL347_11175)	rpoN	PA3349 (AL347_26005)	PA3349	+	Homology from 25780925
rpoN (AL347_11175)	rpoN	PA3346 (AL347_26020)	PA3346 (hsbR)	+	Homology from 25780925
rpoN (AL347_11175)	rpoN	PA3340 (AL347_26055)	PA3340	+	Homology from 25780925
rpoN (AL347_11175)	rpoN	plcN (AL347_26160)	plcN	+	Homology from 25780925
rpoN (AL347_11175)	rpoN	PA3318 (AL347_26165)	PA3318	+	Homology from 25780925
rpoN (AL347_11175)	rpoN	PA3307 (AL347_26225)	PA3307	+	Homology from 25780925
rpoN (AL347_11175)	rpoN	PA3306 (AL347_26230)	PA3306	+	Homology from 25780925
rpoN (AL347_11175)	rpoN	PA3274 (AL347_26415)	PA3274	+	Homology from 25780925
rpoN (AL347_11175)	rpoN	PA3254 (AL347_00250)	PA3254	+	Homology from 25780925
rpoN (AL347_11175)	rpoN	PA3253 (AL347_00255)	PA3253	+	Homology from 25780925
rpoN (AL347_11175)	rpoN	PA3252 (AL347_00260)	PA3252	+	Homology from 25780925
rpoN (AL347_11175)	rpoN	PA3251 (AL347_00265)	PA3251	+	Homology from 25780925
rpoN (AL347_11175)	rpoN	PA3250 (AL347_00270)	PA3250	+	Homology from 25780925
rpoN (AL347_11175)	rpoN	PA3249 (AL347_00275)	PA3249	+	Homology from 25780925
rpoN (AL347_11175)	rpoN	PA3189 (AL347_26870)	PA3189	+	Homology from 25780925
rpoN (AL347_11175)	rpoN	PA3188 (AL347_00590)	PA3188	+	Homology from 25780925
rpoN (AL347_11175)	rpoN	PA3187 (AL347_00595)	PA3187	+	Homology from 25780925
rpoN (AL347_11175)	rpoN	PA3177 (AL347_26935)	PA3177	+	Homology from 25780925
rpoN (AL347_11175)	rpoN	PA3162 (AL347_27010)	PA3162 (rpsA)	+	Homology from 25780925
rpoN (AL347_11175)	rpoN	PA3108 (AL347_27300)	PA3108 (purF)	+	Homology from 25780925
rpoN (AL347_11175)	rpoN	PA3107 (AL347_27305)	PA3107 (metZ)	+	Homology from 25780925
rpoN (AL347_11175)	rpoN	PA3106 (AL347_27310)	PA3106	+	Homology from 25780925
rpoN (AL347_11175)	rpoN	PA3082 (AL347_27450)	PA3082 (gbt)	+	Homology from 25780925
rpoN (AL347_11175)	rpoN	PA3081 (AL347_27455)	PA3081	+	Homology from 25780925
rpoN (AL347_11175)	rpoN	pelA (AL347_27525)	pelA	+	Homology from 25780925
rpoN (AL347_11175)	rpoN	pelB (AL347_27530)	pelB	+	Homology from 25780925
rpoN (AL347_11175)	rpoN	pelC (AL347_27535)	pelC	+	Homology from 25780925
rpoN (AL347_11175)	rpoN	pelD (AL347_27540)	pelD	+	Homology from 25780925
rpoN (AL347_11175)	rpoN	pelE (AL347_27545)	pelE	+	Homology from 25780925
rpoN (AL347_11175)	rpoN	PA3059 (AL347_27550)	PA3059	+	Homology from 25780925
rpoN (AL347_11175)	rpoN	pelG (AL347_27555)	pelG	+	Homology from 25780925
rpoN (AL347_11175)	rpoN	PA3039 (AL347_27655)	PA3039	+	Homology from 25780925
rpoN (AL347_11175)	rpoN	PA3023 (AL347_27740)	PA3023	+	Homology from 25780925
rpoN (AL347_11175)	rpoN	PA3017 (AL347_27770)	PA3017	+	Homology from 25780925
rpoN (AL347_11175)	rpoN	psrA (AL347_27830)	psrA	+	Homology from 25780925
rpoN (AL347_11175)	rpoN	PA2999 (AL347_27865)	PA2999 (nqrA)	+	Homology from 25780925
rpoN (AL347_11175)	rpoN	PA2998 (AL347_27870)	PA2998 (nqrB)	+	Homology from 25780925
rpoN (AL347_11175)	rpoN	PA2997 (AL347_27875)	PA2997 (nqrC)	+	Homology from 25780925

rpoN (AL347_11175)	rpoN	PA2996 (AL347_27880)	PA2996 (nqrD)	+	Homology from 25780925
rpoN (AL347_11175)	rpoN	PA2995 (AL347_27885)	PA2995 (nqrE)	+	Homology from 25780925
rpoN (AL347_11175)	rpoN	PA2994 (AL347_27890)	PA2994 (nqrF)	+	Homology from 25780925
rpoN (AL347_11175)	rpoN	PA2993 (AL347_27895)	PA2993	+	Homology from 25780925
rpoN (AL347_11175)	rpoN	PA2992 (AL347_27900)	PA2992	+	Homology from 25780925
rpoN (AL347_11175)	rpoN	PA2971 (AL347_28015)	PA2971	+	Homology from 25780925
rpoN (AL347_11175)	rpoN	PA2970 (AL347_28020)	PA2970 (rpmF)	+	Homology from 25780925
rpoN (AL347_11175)	rpoN	PA2969 (AL347_28025)	PA2969 (plsX)	+	Homology from 25780925
rpoN (AL347_11175)	rpoN	PA2968 (AL347_28030)	PA2968 (fabD)	+	Homology from 25780925
rpoN (AL347_11175)	rpoN	PA2956 (AL347_28090)	PA2956	+	Homology from 25780925
rpoN (AL347_11175)	rpoN	PA2955 (AL347_28095)	PA2955	+	Homology from 25780925
rpoN (AL347_11175)	rpoN	PA2954 (AL347_28100)	PA2954	+	Homology from 25780925
rpoN (AL347_11175)	rpoN	sbrR (AL347_28410)	sbrR	+	Homology from 25780925
rpoN (AL347_11175)	rpoN	lipA (AL347_28575)	lipA	+	Homology from 25780925
rpoN (AL347_11175)	rpoN	PA2849 (AL347_28645)	PA2849 (ohrR)	+	Homology from 25780925
rpoN (AL347_11175)	rpoN	PA2793 (AL347_29170)	PA2793	+	Homology from 25780925
rpoN (AL347_11175)	rpoN	PA2770 (AL347_29310)	PA2770	+	Homology from 25780925
rpoN (AL347_11175)	rpoN	PA2769 (AL347_29315)	PA2769	+	Homology from 25780925
rpoN (AL347_11175)	rpoN	PA2754 (AL347_29410)	PA2754	+	Homology from 25780925
rpoN (AL347_11175)	rpoN	PA2747 (AL347_29470)	PA2747	+	Homology from 25780925
rpoN (AL347_11175)	rpoN	PA2742 (AL347_29505)	PA2742 (rpmI)	+	Homology from 25780925
rpoN (AL347_11175)	rpoN	PA2740 (AL347_29515)	PA2740 (pheS)	+	Homology from 25780925
rpoN (AL347_11175)	rpoN	PA2739 (AL347_29520)	PA2739 (pheT)	+	Homology from 25780925
rpoN (AL347_11175)	rpoN	PA2708 (AL347_29865)	PA2708	+	Homology from 25780925
rpoN (AL347_11175)	rpoN	PA2704 (AL347_29885)	PA2704	+	Homology from 25780925
rpoN (AL347_11175)	rpoN	fhp (AL347_30090)	fhp	+	Homology from 25780925
rpoN (AL347_11175)	rpoN	ppyR (AL347_30095)	ppyR	+	Homology from 25780925
rpoN (AL347_11175)	rpoN	PA2662 (AL347_30100)	PA2662	+	Homology from 25780925
rpoN (AL347_11175)	rpoN	PA2654 (AL347_30145)	PA2654 (tlpQ)	+	Homology from 25780925
rpoN (AL347_11175)	rpoN	PA2653 (AL347_30150)	PA2653	+	Homology from 25780925
rpoN (AL347_11175)	rpoN	PA2604 (AL347_30405)	PA2604	+	Homology from 25780925
rpoN (AL347_11175)	rpoN	PA2575 (AL347_31750)	PA2575	+	Homology from 25780925
rpoN (AL347_11175)	rpoN	PA2561 (AL347_31830)	PA2561	+	Homology from 25780925
rpoN (AL347_11175)	rpoN	PA2560 (AL347_31835)	PA2560	+	Homology from 25780925
rpoN (AL347_11175)	rpoN	antR (AL347_32110)	antR	+	Homology from 25780925
rpoN (AL347_11175)	rpoN	PA2509 (AL347_32125)	PA2509 (catB)	+	Homology from 25780925
rpoN (AL347_11175)	rpoN	PA2508 (AL347_32130)	PA2508 (catC)	+	Homology from 25780925
rpoN (AL347_11175)	rpoN	PA2507 (AL347_32135)	PA2507 (catA)	+	Homology from 25780925

rpoN (AL347_11175)	rpoN	mexF (AL347_32505)	mexF	+	Homology from 25780925
rpoN (AL347_11175)	rpoN	gcvH2 (AL347_32780)	gcvH2	+	Homology from 25780925
rpoN (AL347_11175)	rpoN	gcvP2 (AL347_32785)	gcvP2	+	Homology from 25780925
rpoN (AL347_11175)	rpoN	sdaA (AL347_32800)	sdaA	+	Homology from 25780925
rpoN (AL347_11175)	rpoN	PA2416 (AL347_32945)	PA2416 (treA)	+	Homology from 25780925
rpoN (AL347_11175)	rpoN	PA2415 (AL347_32950)	PA2415	+	Homology from 25780925
rpoN (AL347_11175)	rpoN	PA2414 (AL347_32955)	PA2414	+	Homology from 25780925
rpoN (AL347_11175)	rpoN	pvdH (AL347_32960)	pvdH	+	Homology from 25780925
rpoN (AL347_11175)	rpoN	msuE (AL347_33235)	msuE	+	Homology from 25780925
rpoN (AL347_11175)	rpoN	msuD (AL347_33240)	msuD	+	Homology from 25780925
rpoN (AL347_11175)	rpoN	msuC (AL347_33245)	msuC	+	Homology from 25780925
rpoN (AL347_11175)	rpoN	PA2354 (AL347_33250)	PA2354 (sfnR1)	+	Homology from 25780925
rpoN (AL347_11175)	rpoN	PA2351 (AL347_33265)	PA2351	+	Homology from 25780925
rpoN (AL347_11175)	rpoN	PA2350 (AL347_33270)	PA2350	+	Homology from 25780925
rpoN (AL347_11175)	rpoN	PA2349 (AL347_33275)	PA2349	+	Homology from 25780925
rpoN (AL347_11175)	rpoN	PA2348 (AL347_33280)	PA2348	+	Homology from 25780925
rpoN (AL347_11175)	rpoN	PA2347 (AL347_33285)	PA2347	+	Homology from 25780925
rpoN (AL347_11175)	rpoN	PA2346 (AL347_33290)	PA2346	+	Homology from 25780925
rpoN (AL347_11175)	rpoN	PA2345 (AL347_33300)	PA2345	+	Homology from 25780925
rpoN (AL347_11175)	rpoN	PA2326 (AL347_33395)	PA2326	+	Homology from 25780925
rpoN (AL347_11175)	rpoN	PA2266 (AL347_00730)	PA2266	+	Homology from 25780925
rpoN (AL347_11175)	rpoN	gad (AL347_00735)	gad	+	Homology from 25780925
rpoN (AL347_11175)	rpoN	PA2264 (AL347_00740)	PA2264	+	Homology from 25780925
rpoN (AL347_11175)	rpoN	PA2244 (AL347_00845)	PA2244 (pslN)	+	Homology from 25780925
rpoN (AL347_11175)	rpoN	PA2200 (AL347_01550)	PA2200	+	Homology from 25780925
rpoN (AL347_11175)	rpoN	PA2199 (AL347_01555)	PA2199	+	Homology from 25780925
rpoN (AL347_11175)	rpoN	PA2198 (AL347_01560)	PA2198	+	Homology from 25780925
rpoN (AL347_11175)	rpoN	PA2197 (AL347_01565)	PA2197	+	Homology from 25780925
rpoN (AL347_11175)	rpoN	PA2187 (AL347_01620)	PA2187	+	Homology from 25780925
rpoN (AL347_11175)	rpoN	PA2181 (AL347_01645)	PA2181	+	Homology from 25780925
rpoN (AL347_11175)	rpoN	PA2178 (AL347_01670)	PA2178	+	Homology from 25780925
rpoN (AL347_11175)	rpoN	PA2175 (AL347_01685)	PA2175	+	Homology from 25780925
rpoN (AL347_11175)	rpoN	PA2173 (AL347_01700)	PA2173	+	Homology from 25780925
rpoN (AL347_11175)	rpoN	PA2172 (AL347_01705)	PA2172	+	Homology from 25780925
rpoN (AL347_11175)	rpoN	PA2171 (AL347_01710)	PA2171	+	Homology from 25780925
rpoN (AL347_11175)	rpoN	PA2170 (AL347_01715)	PA2170	+	Homology from 25780925
rpoN (AL347_11175)	rpoN	PA2168 (AL347_01725)	PA2168	+	Homology from 25780925
rpoN (AL347_11175)	rpoN	PA2167 (AL347_01730)	PA2167	+	Homology from 25780925

rpoN (AL347_11175)	rpoN	PA2166 (AL347_01740)	PA2166	+	Homology from 25780925
rpoN (AL347_11175)	rpoN	PA2165 (AL347_01745)	PA2165	+	Homology from 25780925
rpoN (AL347_11175)	rpoN	PA2164 (AL347_01750)	PA2164	+	Homology from 25780925
rpoN (AL347_11175)	rpoN	PA2163 (AL347_01755)	PA2163	+	Homology from 25780925
rpoN (AL347_11175)	rpoN	PA2162 (AL347_01760)	PA2162	+	Homology from 25780925
rpoN (AL347_11175)	rpoN	PA2161 (AL347_01765)	PA2161	+	Homology from 25780925
rpoN (AL347_11175)	rpoN	PA2160 (AL347_01770)	PA2160	+	Homology from 25780925
rpoN (AL347_11175)	rpoN	PA2159 (AL347_01780)	PA2159	+	Homology from 25780925
rpoN (AL347_11175)	rpoN	PA2158 (AL347_01785)	PA2158	+	Homology from 25780925
rpoN (AL347_11175)	rpoN	PA2157 (AL347_01790)	PA2157	+	Homology from 25780925
rpoN (AL347_11175)	rpoN	PA2156 (AL347_01795)	PA2156	+	Homology from 25780925
rpoN (AL347_11175)	rpoN	PA2155 (AL347_01800)	PA2155	+	Homology from 25780925
rpoN (AL347_11175)	rpoN	PA2154 (AL347_01805)	PA2154	+	Homology from 25780925
rpoN (AL347_11175)	rpoN	PA2153 (AL347_01810)	PA2153 (glgB)	+	Homology from 25780925
rpoN (AL347_11175)	rpoN	PA2152 (AL347_01815)	PA2152	+	Homology from 25780925
rpoN (AL347_11175)	rpoN	PA2149 (AL347_01830)	PA2149	+	Homology from 25780925
rpoN (AL347_11175)	rpoN	PA2144 (AL347_01860)	PA2144 (glgP)	+	Homology from 25780925
rpoN (AL347_11175)	rpoN	PA2143 (AL347_01865)	PA2143	+	Homology from 25780925
rpoN (AL347_11175)	rpoN	PA2142 (AL347_01875)	PA2142	+	Homology from 25780925
rpoN (AL347_11175)	rpoN	PA2140 (AL347_01885)	PA2140	+	Homology from 25780925
rpoN (AL347_11175)	rpoN	PA2138 (AL347_01895)	PA2138 (ligD)	+	Homology from 25780925
rpoN (AL347_11175)	rpoN	PA2137 (AL347_01900)	PA2137	+	Homology from 25780925
rpoN (AL347_11175)	rpoN	PA2136 (AL347_01905)	PA2136	+	Homology from 25780925
rpoN (AL347_11175)	rpoN	PA2135 (AL347_01915)	PA2135	+	Homology from 25780925
rpoN (AL347_11175)	rpoN	PA2134 (AL347_01920)	PA2134	+	Homology from 25780925
rpoN (AL347_11175)	rpoN	PA2126 (AL347_01965)	PA2126 (cgrC)	+	Homology from 25780925
rpoN (AL347_11175)	rpoN	PA2115 (AL347_02035)	PA2115	+	Homology from 25780925
rpoN (AL347_11175)	rpoN	PA2045 (AL347_02380)	PA2045	+	Homology from 25780925
rpoN (AL347_11175)	rpoN	PA2042 (AL347_02395)	PA2042	+	Homology from 25780925
rpoN (AL347_11175)	rpoN	PA2021 (AL347_02500)	PA2021	+	Homology from 25780925
rpoN (AL347_11175)	rpoN	PA2004 (AL347_02590)	PA2004	+	Homology from 25780925
rpoN (AL347_11175)	rpoN	exaC (AL347_02690)	exaC	+	Homology from 25780925
rpoN (AL347_11175)	rpoN	PA1968 (AL347_02770)	PA1968	+	Homology from 25780925
rpoN (AL347_11175)	rpoN	PA1967 (AL347_02775)	PA1967	+	Homology from 25780925
rpoN (AL347_11175)	rpoN	PA1956 (AL347_02835)	PA1956 (fapA)	+	Homology from 25780925
rpoN (AL347_11175)	rpoN	PA1955 (AL347_02840)	PA1955 (fapB)	+	Homology from 25780925
rpoN (AL347_11175)	rpoN	PA1954 (AL347_02845)	PA1954 (fapC)	+	Homology from 25780925
rpoN (AL347_11175)	rpoN	PA1953 (AL347_02850)	PA1953 (fapD)	+	Homology from 25780925

rpoN (AL347_11175)	rpoN	PA1952 (AL347_02855)	PA1952 (fapE)	+	Homology from 25780925
rpoN (AL347_11175)	rpoN	PA1932 (AL347_02940)	PA1932	+	Homology from 25780925
rpoN (AL347_11175)	rpoN	PA1913 (AL347_03045)	PA1913	+	Homology from 25780925
rpoN (AL347_11175)	rpoN	PA1887 (AL347_03185)	PA1887	+	Homology from 25780925
rpoN (AL347_11175)	rpoN	PA1870 (AL347_03270)	PA1870	+	Homology from 25780925
rpoN (AL347_11175)	rpoN	PA1864 (AL347_03305)	PA1864	+	Homology from 25780925
rpoN (AL347_11175)	rpoN	PA1829 (AL347_03495)	PA1829	+	Homology from 25780925
rpoN (AL347_11175)	rpoN	PA1828 (AL347_03500)	PA1828	+	Homology from 25780925
rpoN (AL347_11175)	rpoN	PA1821 (AL347_03535)	PA1821	+	Homology from 25780925
rpoN (AL347_11175)	rpoN	PA1820 (AL347_03540)	PA1820 (nhaB)	+	Homology from 25780925
rpoN (AL347_11175)	rpoN	PA1782 (AL347_03770)	PA1782	+	Homology from 25780925
rpoN (AL347_11175)	rpoN	PA1779 (AL347_03785)	PA1779	+	Homology from 25780925
rpoN (AL347_11175)	rpoN	PA1778 (AL347_03790)	PA1778 (cobA)	+	Homology from 25780925
rpoN (AL347_11175)	rpoN	PA1771 (AL347_03825)	PA1771 (estX)	+	Homology from 25780925
rpoN (AL347_11175)	rpoN	fadB (AL347_04010)	fadB	+	Homology from 25780925
rpoN (AL347_11175)	rpoN	PA1736 (AL347_04015)	PA1736	+	Homology from 25780925
rpoN (AL347_11175)	rpoN	PA1733 (AL347_04030)	PA1733	+	Homology from 25780925
rpoN (AL347_11175)	rpoN	PA1732 (AL347_04035)	PA1732	+	Homology from 25780925
rpoN (AL347_11175)	rpoN	PA1731 (AL347_04040)	PA1731	+	Homology from 25780925
rpoN (AL347_11175)	rpoN	PA1730 (AL347_04045)	PA1730	+	Homology from 25780925
rpoN (AL347_11175)	rpoN	pscK (AL347_04080)	pscK	+	Homology from 25780925
rpoN (AL347_11175)	rpoN	pscI (AL347_04090)	pscI	+	Homology from 25780925
rpoN (AL347_11175)	rpoN	pscH (AL347_04095)	pscH	+	Homology from 25780925
rpoN (AL347_11175)	rpoN	pscG (AL347_04100)	pscG	+	Homology from 25780925
rpoN (AL347_11175)	rpoN	pscF (AL347_04105)	pscF	+	Homology from 25780925
rpoN (AL347_11175)	rpoN	pscC (AL347_04120)	pscC	+	Homology from 25780925
rpoN (AL347_11175)	rpoN	PA1708 (AL347_04160)	PA1708 (popB, pepB)	+	Homology from 25780925
rpoN (AL347_11175)	rpoN	PA1706 (AL347_04170)	PA1706 (pcrV)	+	Homology from 25780925
rpoN (AL347_11175)	rpoN	PA1703 (AL347_04185)	PA1703 (pcrD)	+	Homology from 25780925
rpoN (AL347_11175)	rpoN	PA1701 (AL347_04195)	PA1701 (pcr3)	+	Homology from 25780925
rpoN (AL347_11175)	rpoN	PA1696 (AL347_04220)	PA1696 (pscO)	+	Homology from 25780925
rpoN (AL347_11175)	rpoN	PA1693 (AL347_04235)	PA1693 (pscR)	+	Homology from 25780925
rpoN (AL347_11175)	rpoN	PA1690 (AL347_04250)	PA1690 (pscU)	+	Homology from 25780925
rpoN (AL347_11175)	rpoN	PA1679 (AL347_04310)	PA1679	+	Homology from 25780925
rpoN (AL347_11175)	rpoN	PA1646 (AL347_04485)	PA1646	+	Homology from 25780925
rpoN (AL347_11175)	rpoN	PA1608 (AL347_04685)	PA1608	+	Homology from 25780925
rpoN (AL347_11175)	rpoN	PA1605 (AL347_04700)	PA1605	+	Homology from 25780925
rpoN (AL347_11175)	rpoN	PA1603 (AL347_04710)	PA1603	+	Homology from 25780925

rpoN (AL347_11175)	rpoN	PA1602 (AL347_04715)	PA1602	+	Homology from 25780925
rpoN (AL347_11175)	rpoN	PA1601 (AL347_04720)	PA1601	+	Homology from 25780925
rpoN (AL347_11175)	rpoN	PA1600 (AL347_04725)	PA1600	+	Homology from 25780925
rpoN (AL347_11175)	rpoN	PA1591 (AL347_04770)	PA1591	+	Homology from 25780925
rpoN (AL347_11175)	rpoN	pauA3 (AL347_04910)	pauA3	+	Homology from 25780925
rpoN (AL347_11175)	rpoN	aer (AL347_04935)	aer	+	Homology from 25780925
rpoN (AL347_11175)	rpoN	PA4728 (AL347_13215)	PA4728 (folK)	+	Homology from 25780925
rpoN (AL347_11175)	rpoN	ccoN2 (AL347_04950)	ccoN2	+	Homology from 25780925
rpoN (AL347_11175)	rpoN	ccoO2 (AL347_04955)	ccoO2	+	Homology from 25780925
rpoN (AL347_11175)	rpoN	PA1545 (AL347_05025)	PA1545	+	Homology from 25780925
rpoN (AL347_11175)	rpoN	anr (AL347_05030)	anr	+	Homology from 25780925
rpoN (AL347_11175)	rpoN	PA1543 (AL347_05035)	PA1543 (apt)	+	Homology from 25780925
rpoN (AL347_11175)	rpoN	PA1531 (AL347_05095)	PA1531	+	Homology from 25780925
rpoN (AL347_11175)	rpoN	PA1517 (AL347_05175)	PA1517	+	Homology from 25780925
rpoN (AL347_11175)	rpoN	PA1516 (AL347_05180)	PA1516	+	Homology from 25780925
rpoN (AL347_11175)	rpoN	alc (AL347_05185)	alc	+	Homology from 25780925
rpoN (AL347_11175)	rpoN	PA1514 (AL347_05190)	PA1514	+	Homology from 25780925
rpoN (AL347_11175)	rpoN	PA1513 (AL347_05195)	PA1513	+	Homology from 25780925
rpoN (AL347_11175)	rpoN	PA1490 (AL347_05310)	PA1490	+	Homology from 25780925
rpoN (AL347_11175)	rpoN	PA1474 (AL347_05395)	PA1474	+	Homology from 25780925
rpoN (AL347_11175)	rpoN	PA1473 (AL347_05400)	PA1473	+	Homology from 25780925
rpoN (AL347_11175)	rpoN	flhA (AL347_05505)	flhA	+	Homology from 25780925
rpoN (AL347_11175)	rpoN	fliR (AL347_05525)	fliR	+	Homology from 25780925
rpoN (AL347_11175)	rpoN	fliQ (AL347_05530)	fliQ	+	Homology from 25780925
rpoN (AL347_11175)	rpoN	fliP (AL347_05535)	fliP	+	Homology from 25780925
rpoN (AL347_11175)	rpoN	fliO (AL347_05540)	fliO	+	Homology from 25780925
rpoN (AL347_11175)	rpoN	fliN (AL347_05545)	fliN	+	Homology from 25780925
rpoN (AL347_11175)	rpoN	fliM (AL347_05550)	fliM	+	Homology from 25780925
rpoN (AL347_11175)	rpoN	fliL (AL347_05555)	fliL	+	Homology from 25780925
rpoN (AL347_11175)	rpoN	PA1441 (AL347_05560)	PA1441	+	Homology from 25780925
rpoN (AL347_11175)	rpoN	PA1440 (AL347_05565)	PA1440	+	Homology from 25780925
rpoN (AL347_11175)	rpoN	PA1439 (AL347_05570)	PA1439	+	Homology from 25780925
rpoN (AL347_11175)	rpoN	PA1404 (AL347_05800)	PA1404	+	Homology from 25780925
rpoN (AL347_11175)	rpoN	PA1342 (AL347_06385)	PA1342 (aatJ)	+	Homology from 25780925
rpoN (AL347_11175)	rpoN	PA1324 (AL347_06485)	PA1324	+	Homology from 25780925
rpoN (AL347_11175)	rpoN	cyoE (AL347_06500)	cyoE	+	Homology from 25780925
rpoN (AL347_11175)	rpoN	cyoD (AL347_06505)	cyoD	+	Homology from 25780925
rpoN (AL347_11175)	rpoN	cyoC (AL347_06510)	cyoC	+	Homology from 25780925

rpoN (AL347_11175)	rpoN	cyoB (AL347_06515)	cyoB	+	Homology from 25780925
rpoN (AL347_11175)	rpoN	cyoA (AL347_06520)	cyoA	+	Homology from 25780925
rpoN (AL347_11175)	rpoN	PA1277 (AL347_06725)	PA1277 (cobQ, cbiP)	+	Homology from 25780925
rpoN (AL347_11175)	rpoN	lhpP (AL347_06820)	lhpP	+	Homology from 25780925
rpoN (AL347_11175)	rpoN	PA1259 (AL347_06825)	PA1259 (lhpH)	+	Homology from 25780925
rpoN (AL347_11175)	rpoN	lhpM (AL347_06830)	lhpM	+	Homology from 25780925
rpoN (AL347_11175)	rpoN	lhpN (AL347_06835)	lhpN	+	Homology from 25780925
rpoN (AL347_11175)	rpoN	lhpO (AL347_06840)	lhpO	+	Homology from 25780925
rpoN (AL347_11175)	rpoN	PA1255 (AL347_06845)	PA1255 (lhpK)	+	Homology from 25780925
rpoN (AL347_11175)	rpoN	PA1243 (AL347_06915)	PA1243	+	Homology from 25780925
rpoN (AL347_11175)	rpoN	PA1197 (AL347_07145)	PA1197	+	Homology from 25780925
rpoN (AL347_11175)	rpoN	ddaH (AL347_07155)	ddaH	+	Homology from 25780925
rpoN (AL347_11175)	rpoN	PA1194 (AL347_07160)	PA1194	+	Homology from 25780925
rpoN (AL347_11175)	rpoN	dctA (AL347_07215)	dctA	+	Homology from 25780925
rpoN (AL347_11175)	rpoN	dctR (AL347_07225)	dctR	+	Homology from 25780925
rpoN (AL347_11175)	rpoN	PA1170 (AL347_07285)	PA1170	+	Homology from 25780925
rpoN (AL347_11175)	rpoN	nrdA (AL347_07355)	nrdA	+	Homology from 25780925
rpoN (AL347_11175)	rpoN	PA1115 (AL347_07585)	PA1115	+	Homology from 25780925
rpoN (AL347_11175)	rpoN	PA1114 (AL347_07590)	PA1114	+	Homology from 25780925
rpoN (AL347_11175)	rpoN	PA1111 (AL347_07620)	PA1111	+	Homology from 25780925
rpoN (AL347_11175)	rpoN	fliJ (AL347_07650)	fliJ	+	Homology from 25780925
rpoN (AL347_11175)	rpoN	fliI (AL347_07655)	fliI	+	Homology from 25780925
rpoN (AL347_11175)	rpoN	fliH (AL347_07660)	fliH	+	Homology from 25780925
rpoN (AL347_11175)	rpoN	fliG (AL347_07665)	fliG	+	Homology from 25780925
rpoN (AL347_11175)	rpoN	fliF (AL347_07670)	fliF	+	Homology from 25780925
rpoN (AL347_11175)	rpoN	fliE (AL347_07675)	fliE	+	Homology from 25780925
rpoN (AL347_11175)	rpoN	fleR (AL347_07680)	fleR	+	Homology from 25780925
rpoN (AL347_11175)	rpoN	fleS (AL347_07685)	fleS	+	Homology from 25780925
rpoN (AL347_11175)	rpoN	PA1096 (AL347_07695)	PA1096	+	Homology from 25780925
rpoN (AL347_11175)	rpoN	fliS (AL347_07700)	fliS	+	Homology from 25780925
rpoN (AL347_11175)	rpoN	fliD (AL347_07705)	fliD	+	Homology from 25780925
rpoN (AL347_11175)	rpoN	PA1093 (AL347_07710)	PA1093	+	Homology from 25780925
rpoN (AL347_11175)	rpoN	PA1091 (AL347_07720)	PA1091 (fgtA)	+	Homology from 25780925
rpoN (AL347_11175)	rpoN	PA1089 (AL347_07730)	PA1089	+	Homology from 25780925
rpoN (AL347_11175)	rpoN	flgG (AL347_07765)	flgG	+	Homology from 25780925
rpoN (AL347_11175)	rpoN	flgE (AL347_07775)	flgE	+	Homology from 25780925
rpoN (AL347_11175)	rpoN	PA1014 (AL347_08130)	PA1014 (wapB)	+	Homology from 25780925
rpoN (AL347_11175)	rpoN	PA1004 (AL347_08185)	PA1004 (nadA)	+	Homology from 25780925

rpoN (AL347_11175)	rpoN	PA0976 (AL347_08325)	PA0976	+	Homology from 25780925
rpoN (AL347_11175)	rpoN	PA0975 (AL347_08330)	PA0975	+	Homology from 25780925
rpoN (AL347_11175)	rpoN	PA0974 (AL347_08335)	PA0974	+	Homology from 25780925
rpoN (AL347_11175)	rpoN	oprL (AL347_08340)	oprL	+	Homology from 25780925
rpoN (AL347_11175)	rpoN	PA0947 (AL347_08475)	PA0947	+	Homology from 25780925
rpoN (AL347_11175)	rpoN	PA0942 (AL347_08505)	PA0942	+	Homology from 25780925
rpoN (AL347_11175)	rpoN	PA0941 (AL347_08510)	PA0941	+	Homology from 25780925
rpoN (AL347_11175)	rpoN	PA0940 (AL347_08515)	PA0940	+	Homology from 25780925
rpoN (AL347_11175)	rpoN	PA0939 (AL347_08520)	PA0939	+	Homology from 25780925
rpoN (AL347_11175)	rpoN	PA0923 (AL347_08605)	PA0923 (dinB, dinP)	+	Homology from 25780925
rpoN (AL347_11175)	rpoN	PA0921 (AL347_08620)	PA0921	+	Homology from 25780925
rpoN (AL347_11175)	rpoN	PA0910 (AL347_08930)	PA0910 (alpD)	+	Homology from 25780925
rpoN (AL347_11175)	rpoN	PA0812 (AL347_09450)	PA0812	+	Homology from 25780925
rpoN (AL347_11175)	rpoN	PA0811 (AL347_09455)	PA0811	+	Homology from 25780925
rpoN (AL347_11175)	rpoN	PA0810 (AL347_09460)	PA0810	+	Homology from 25780925
rpoN (AL347_11175)	rpoN	asrA (AL347_09620)	asrA	+	Homology from 25780925
rpoN (AL347_11175)	rpoN	PA0778 (AL347_09625)	PA0778 (icp)	+	Homology from 25780925
rpoN (AL347_11175)	rpoN	PA0777 (AL347_09630)	PA0777	+	Homology from 25780925
rpoN (AL347_11175)	rpoN	vfr (AL347_21140)	vfr	+	Homology from 25780925
rpoN (AL347_11175)	rpoN	PA0602 (AL347_20890)	PA0602	+	Homology from 25780925
rpoN (AL347_11175)	rpoN	PA0578 (AL347_20770)	PA0578	+	Homology from 25780925
rpoN (AL347_11175)	rpoN	PA0575 (AL347_20755)	PA0575 (rmcA)	+	Homology from 25780925
rpoN (AL347_11175)	rpoN	PA0508 (AL347_20375)	PA0508	+	Homology from 25780925
rpoN (AL347_11175)	rpoN	PA0505 (AL347_20355)	PA0505	+	Homology from 25780925
rpoN (AL347_11175)	rpoN	PA0476 (AL347_20215)	PA0476	+	Homology from 25780925
rpoN (AL347_11175)	rpoN	PA0456 (AL347_20105)	PA0456	+	Homology from 25780925
rpoN (AL347_11175)	rpoN	PA0444 (AL347_20050)	PA0444	+	Homology from 25780925
rpoN (AL347_11175)	rpoN	PA0443 (AL347_20045)	PA0443	+	Homology from 25780925
rpoN (AL347_11175)	rpoN	PA0439 (AL347_20020)	PA0439	+	Homology from 25780925
rpoN (AL347_11175)	rpoN	codB (AL347_20010)	codB	+	Homology from 25780925
rpoN (AL347_11175)	rpoN	codA (AL347_20005)	codA	+	Homology from 25780925
rpoN (AL347_11175)	rpoN	PA0431 (AL347_19975)	PA0431	+	Homology from 25780925
rpoN (AL347_11175)	rpoN	PA0382 (AL347_19720)	PA0382 (micA, yggH)	+	Homology from 25780925
rpoN (AL347_11175)	rpoN	PA0381 (AL347_19715)	PA0381 (thiG)	+	Homology from 25780925
rpoN (AL347_11175)	rpoN	PA0354 (AL347_19575)	PA0354	+	Homology from 25780925
rpoN (AL347_11175)	rpoN	PA0329 (AL347_19445)	PA0329	+	Homology from 25780925
rpoN (AL347_11175)	rpoN	PA0328 (AL347_19440)	PA0328 (aaaA)	+	Homology from 25780925
rpoN (AL347_11175)	rpoN	PA0324 (AL347_19415)	PA0324	+	Homology from 25780925

rpoN (AL347_11175)	rpoN	PA0322 (AL347_19405)	PA0322	+	Homology from 25780925
rpoN (AL347_11175)	rpoN	PA0321 (AL347_19400)	PA0321	+	Homology from 25780925
rpoN (AL347_11175)	rpoN	spuH (AL347_19305)	spuH	+	Homology from 25780925
rpoN (AL347_11175)	rpoN	spuG (AL347_19300)	spuG	+	Homology from 25780925
rpoN (AL347_11175)	rpoN	spuF (AL347_19295)	spuF	+	Homology from 25780925
rpoN (AL347_11175)	rpoN	oprE (AL347_19240)	oprE	+	Homology from 25780925
rpoN (AL347_11175)	rpoN	PA0222 (AL347_18880)	PA0222	+	Homology from 25780925
rpoN (AL347_11175)	rpoN	PA0221 (AL347_18875)	PA0221	+	Homology from 25780925
rpoN (AL347_11175)	rpoN	PA0220 (AL347_18870)	PA0220	+	Homology from 25780925
rpoN (AL347_11175)	rpoN	PA0219 (AL347_18865)	PA0219	+	Homology from 25780925
rpoN (AL347_11175)	rpoN	PA0218 (AL347_18855)	PA0218	+	Homology from 25780925
rpoN (AL347_11175)	rpoN	PA0168 (AL347_18625)	PA0168	+	Homology from 25780925
rpoN (AL347_11175)	rpoN	PA0167 (AL347_18620)	PA0167	+	Homology from 25780925
rpoN (AL347_11175)	rpoN	PA0166 (AL347_18615)	PA0166	+	Homology from 25780925
rpoN (AL347_11175)	rpoN	PA0165 (AL347_18610)	PA0165	+	Homology from 25780925
rpoN (AL347_11175)	rpoN	triC (AL347_18575)	triC	+	Homology from 25780925
rpoN (AL347_11175)	rpoN	triB (AL347_18570)	triB	+	Homology from 25780925
rpoN (AL347_11175)	rpoN	PA0156 (AL347_18565)	PA0156 (triA)	+	Homology from 25780925
rpoN (AL347_11175)	rpoN	PA0147 (AL347_18515)	PA0147	+	Homology from 25780925
rpoN (AL347_11175)	rpoN	PA0146 (AL347_18510)	PA0146	+	Homology from 25780925
rpoN (AL347_11175)	rpoN	PA0144 (AL347_18500)	PA0144	+	Homology from 25780925
rpoN (AL347_11175)	rpoN	PA0138 (AL347_18465)	PA0138	+	Homology from 25780925
rpoN (AL347_11175)	rpoN	PA0137 (AL347_18460)	PA0137	+	Homology from 25780925
rpoN (AL347_11175)	rpoN	PA0136 (AL347_18455)	PA0136	+	Homology from 25780925
rpoN (AL347_11175)	rpoN	PA0116 (AL347_18355)	PA0116	+	Homology from 25780925
rpoN (AL347_11175)	rpoN	PA0115 (AL347_18350)	PA0115	+	Homology from 25780925
rpoN (AL347_11175)	rpoN	PA0046 (AL347_18000)	PA0046	+	Homology from 25780925
rpoN (AL347_11175)	rpoN	PA0045 (AL347_17995)	PA0045	+	Homology from 25780925
rpoN (AL347_11175)	rpoN	sbrI (AL347_28405)	sbrI	?	Homology from 25780925, 29729420
rpoN (AL347_11175)	rpoN	algD (AL347_25110)	algD	d	27242034*, 18974177*, 22587778
rpoN (AL347_11175)	rpoN	flgB (AL347_07790)	flgB	+	27242034*, 18974177*, 22587778
rpoN (AL347_11175)	rpoN	flgC (AL347_07785)	flgC	+	27242034*, 18974177*, 22587778
rpoN (AL347_11175)	rpoN	flgD (AL347_07780)	flgD	+	27242034*, 18974177*, 22587778
rpoN (AL347_11175)	rpoN	flgF (AL347_07770)	flgF	+	27242034*, 18974177*, 22587778
rpoN (AL347_11175)	rpoN	flgH (AL347_07760)	flgH	+	27242034*, 18974177*, 22587778
rpoN (AL347_11175)	rpoN	flgI (AL347_07755)	flgI	+	27242034*, 18974177*, 22587778
rpoN (AL347_11175)	rpoN	flgJ (AL347_07750)	flgJ	+	27242034*, 18974177*, 22587778
rpoN (AL347_11175)	rpoN	flgK (AL347_07745)	flgK	+	27242034*, 18974177*, 22587778

rpoN (AL347_11175)	rpoN	flgL (AL347_07740)	flgL	+	27242034*, 18974177*, 22587778
rpoN (AL347_11175)	rpoN	flhF (AL347_05500)	flhF	+	27242034*, 18974177*, 22587778
rpoN (AL347_11175)	rpoN	PA1442 (AL347_05555)	PA1442	+	27242034*, 18974177*, 22587778
rpoN (AL347_11175)	rpoN	wbpI (AL347_27085)	orfK	+	27242034*, 18974177*, 22587778
rpoN (AL347_11175)	rpoN	wbpK (AL347_27095)	orfM	+	27242034*, 18974177*, 22587778
rpoN (AL347_11175)	rpoN	wbpL (AL347_27100)	orfN	+	27242034*, 18974177*, 22587778
rpoN (AL347_11175)	rpoN	phaG (AL347_07885)	phaG	+	27242034*, 18974177*, 22587778
rpoN (AL347_11175)	rpoN	gacA (AL347_30505)	gacA	?	27242034*
rpoS (AL347_24665)	rpoS	rhlI (AL347_25330)	rhlI	-	10894749
rpoS (AL347_24665)	rpoS	exsC (AL347_04150)	exsC	+	18974177
rpoS (AL347_24665)	rpoS	exsE (AL347_04145)	exsE	+	18974177
rpoS (AL347_24665)	rpoS	exsB (AL347_04140)	exsB	+	18974177
rpoS (AL347_24665)	rpoS	exsA (AL347_04135)	exsA	+	18974177
rpoS (AL347_24665)	rpoS	azu (AL347_14220)	azu	+	18974177
rpoS (AL347_24665)	rpoS	coxB (AL347_18300)	coxB	+	19930444
rpoS (AL347_24665)	rpoS	coxA (AL347_18305)	coxA	+	19930444
rpoS (AL347_24665)	rpoS	PA0107 (AL347_18310)	PA0107	+	19930444
rpoS (AL347_24665)	rpoS	coIII (AL347_18315)	coIII	+	19930444
rpoS (AL347_24665)	rpoS	cioB (AL347_23070)	cioB	+	19930444
rpoS (AL347_24665)	rpoS	cioA (AL347_23065)	cioA	+	19930444
rpoS (AL347_24665)	rpoS	bphO (AL347_22115)	bphO	+	21415115
rpoS (AL347_24665)	rpoS	bphP (AL347_22110)	bphP	+	21415115
rpoS (AL347_24665)	rpoS	PA1351 (AL347_06340)	PA1351	?	29729420
rpoS (AL347_24665)	rpoS	PA5537 (AL347_17505)	PA5537	+	Homology from 25780925
rpoS (AL347_24665)	rpoS	yegQ (AL347_17005)	yegQ	+	Homology from 25780925
rpoS (AL347_24665)	rpoS	PA5439 (AL347_17000)	PA5439	+	Homology from 25780925
rpoS (AL347_24665)	rpoS	PA5359 (AL347_16555)	PA5359	+	Homology from 25780925
rpoS (AL347_24665)	rpoS	PA5343 (AL347_16470)	PA5343	+	Homology from 25780925
rpoS (AL347_24665)	rpoS	PA5342 (AL347_16465)	PA5342	+	Homology from 25780925
rpoS (AL347_24665)	rpoS	PA5319 (AL347_16350)	PA5319 (radC)	+	Homology from 25780925
rpoS (AL347_24665)	rpoS	PA5283 (AL347_16165)	PA5283	+	Homology from 25780925
rpoS (AL347_24665)	rpoS	PA5208 (AL347_15775)	PA5208	+	Homology from 25780925
rpoS (AL347_24665)	rpoS	PA5207 (AL347_15770)	PA5207	+	Homology from 25780925
rpoS (AL347_24665)	rpoS	arcC (AL347_15590)	arcC	+	Homology from 25780925
rpoS (AL347_24665)	rpoS	arcB (AL347_15585)	arcB	+	Homology from 25780925
rpoS (AL347_24665)	rpoS	arcA (AL347_15580)	arcA	+	Homology from 25780925
rpoS (AL347_24665)	rpoS	arcD (AL347_15575)	arcD	+	Homology from 25780925
rpoS (AL347_24665)	rpoS	PA5103 (AL347_15160)	PA5103 (puuR)	+	Homology from 25780925

rpoS (AL347_24665)	rpoS	PA5102 (AL347_15155)	PA5102	+	Homology from 25780925
rpoS (AL347_24665)	rpoS	PA5101 (AL347_15150)	PA5101	+	Homology from 25780925
rpoS (AL347_24665)	rpoS	PA5059 (AL347_14945)	PA5059	+	Homology from 25780925
rpoS (AL347_24665)	rpoS	PA5058 (AL347_14940)	PA5058 (phaC2, phaC)	+	Homology from 25780925
rpoS (AL347_24665)	rpoS	aceE (AL347_14705)	aceE	+	Homology from 25780925
rpoS (AL347_24665)	rpoS	PA4929 (AL347_14255)	PA4929	+	Homology from 25780925
rpoS (AL347_24665)	rpoS	PA4915 (AL347_14185)	PA4915	+	Homology from 25780925
rpoS (AL347_24665)	rpoS	PA4874 (AL347_13980)	PA4874	+	Homology from 25780925
rpoS (AL347_24665)	rpoS	PA4829 (AL347_13750)	PA4829 (lpd3)	+	Homology from 25780925
rpoS (AL347_24665)	rpoS	PA4781 (AL347_13505)	PA4781	+	Homology from 25780925
rpoS (AL347_24665)	rpoS	fur (AL347_13415)	fur	+	Homology from 25780925
rpoS (AL347_24665)	rpoS	carA (AL347_13380)	carA	+	Homology from 25780925
rpoS (AL347_24665)	rpoS	PA4757 (AL347_13375)	PA4757	+	Homology from 25780925
rpoS (AL347_24665)	rpoS	carB (AL347_13370)	carB	+	Homology from 25780925
rpoS (AL347_24665)	rpoS	PA4703 (AL347_13085)	PA4703	+	Homology from 25780925
rpoS (AL347_24665)	rpoS	PA4702 (AL347_13080)	PA4702	+	Homology from 25780925
rpoS (AL347_24665)	rpoS	PA4657 (AL347_12805)	PA4657	+	Homology from 25780925
rpoS (AL347_24665)	rpoS	cupE6 (AL347_12785)	cupE6	+	Homology from 25780925
rpoS (AL347_24665)	rpoS	cupE5 (AL347_12780)	cupE5	+	Homology from 25780925
rpoS (AL347_24665)	rpoS	cupE4 (AL347_12775)	cupE4	+	Homology from 25780925
rpoS (AL347_24665)	rpoS	cupE3 (AL347_12770)	cupE3	+	Homology from 25780925
rpoS (AL347_24665)	rpoS	cupE2 (AL347_12765)	cupE2	+	Homology from 25780925
rpoS (AL347_24665)	rpoS	cupE1 (AL347_12760)	cupE1	+	Homology from 25780925
rpoS (AL347_24665)	rpoS	PA4647 (AL347_12755)	PA4647 (uraA, pyrP)	+	Homology from 25780925
rpoS (AL347_24665)	rpoS	PA4639 (AL347_12715)	PA4639	+	Homology from 25780925
rpoS (AL347_24665)	rpoS	PA4631 (AL347_12665)	PA4631	+	Homology from 25780925
rpoS (AL347_24665)	rpoS	PA4624 (AL347_12630)	PA4624 (cdrB)	+	Homology from 25780925
rpoS (AL347_24665)	rpoS	PA4608 (AL347_12535)	PA4608 (mapZ)	+	Homology from 25780925
rpoS (AL347_24665)	rpoS	PA4607 (AL347_12530)	PA4607	+	Homology from 25780925
rpoS (AL347_24665)	rpoS	PA4573 (AL347_12350)	PA4573	+	Homology from 25780925
rpoS (AL347_24665)	rpoS	PA4540 (AL347_11575)	PA4540	+	Homology from 25780925
rpoS (AL347_24665)	rpoS	PA4523 (AL347_11485)	PA4523	+	Homology from 25780925
rpoS (AL347_24665)	rpoS	PA4497 (AL347_11350)	PA4497 (dppA2)	+	Homology from 25780925
rpoS (AL347_24665)	rpoS	PA4496 (AL347_11345)	PA4496 (dppA1)	+	Homology from 25780925
rpoS (AL347_24665)	rpoS	roxS (AL347_11335)	roxS	+	Homology from 25780925
rpoS (AL347_24665)	rpoS	roxR (AL347_11330)	roxR	+	Homology from 25780925
rpoS (AL347_24665)	rpoS	PA4431 (AL347_11020)	PA4431	+	Homology from 25780925
rpoS (AL347_24665)	rpoS	PA4430 (AL347_11015)	PA4430	+	Homology from 25780925

rpoS (AL347_24665)	rpoS	PA4429 (AL347_11010)	PA4429	+	Homology from 25780925
rpoS (AL347_24665)	rpoS	PA4428 (AL347_11005)	PA4428 (sspA)	+	Homology from 25780925
rpoS (AL347_24665)	rpoS	PA4427 (AL347_11000)	PA4427 (sspB)	+	Homology from 25780925
rpoS (AL347_24665)	rpoS	sodB (AL347_10680)	sodB	+	Homology from 25780925
rpoS (AL347_24665)	rpoS	PA4362 (AL347_10660)	PA4362	+	Homology from 25780925
rpoS (AL347_24665)	rpoS	flp (AL347_10365)	flp	+	Homology from 25780925
rpoS (AL347_24665)	rpoS	rcpC (AL347_10360)	rcpC	+	Homology from 25780925
rpoS (AL347_24665)	rpoS	rcpA (AL347_10355)	rcpA	+	Homology from 25780925
rpoS (AL347_24665)	rpoS	tadA (AL347_10345)	tadA	+	Homology from 25780925
rpoS (AL347_24665)	rpoS	tadB (AL347_10340)	tadB	+	Homology from 25780925
rpoS (AL347_24665)	rpoS	tadZ (AL347_10350)	tadZ	+	Homology from 25780925
rpoS (AL347_24665)	rpoS	tadD (AL347_10330)	tadD	+	Homology from 25780925
rpoS (AL347_24665)	rpoS	pprB (AL347_10315)	pprB	+	Homology from 25780925
rpoS (AL347_24665)	rpoS	PA4294 (AL347_10305)	PA4294	+	Homology from 25780925
rpoS (AL347_24665)	rpoS	pprA (AL347_10300)	pprA	+	Homology from 25780925
rpoS (AL347_24665)	rpoS	PA4282 (AL347_10245)	PA4282	+	Homology from 25780925
rpoS (AL347_24665)	rpoS	PA4281 (AL347_10240)	PA4281 (sbcD)	+	Homology from 25780925
rpoS (AL347_24665)	rpoS	PA4112 (AL347_22135)	PA4112	+	Homology from 25780925
rpoS (AL347_24665)	rpoS	PA3957 (AL347_22925)	PA3957	+	Homology from 25780925
rpoS (AL347_24665)	rpoS	PA3956 (AL347_22930)	PA3956	+	Homology from 25780925
rpoS (AL347_24665)	rpoS	PA3945 (AL347_22990)	PA3945	+	Homology from 25780925
rpoS (AL347_24665)	rpoS	PA3921 (AL347_23115)	PA3921	+	Homology from 25780925
rpoS (AL347_24665)	rpoS	PA3920 (AL347_23120)	PA3920 (copA1, yvgX, cueA)	+	Homology from 25780925
rpoS (AL347_24665)	rpoS	PA3858 (AL347_23445)	PA3858	+	Homology from 25780925
rpoS (AL347_24665)	rpoS	PA3833 (AL347_23585)	PA3833	+	Homology from 25780925
rpoS (AL347_24665)	rpoS	PA3786 (AL347_23825)	PA3786	+	Homology from 25780925
rpoS (AL347_24665)	rpoS	PA3740 (AL347_24070)	PA3740	+	Homology from 25780925
rpoS (AL347_24665)	rpoS	PA3688 (AL347_24335)	PA3688	+	Homology from 25780925
rpoS (AL347_24665)	rpoS	PA3687 (AL347_24340)	PA3687 (ppc)	+	Homology from 25780925
rpoS (AL347_24665)	rpoS	rpoS (AL347_24665)	rpoS	?	Homology from 25780925
rpoS (AL347_24665)	rpoS	PA3593 (AL347_24820)	PA3593	+	Homology from 25780925
rpoS (AL347_24665)	rpoS	PA3592 (AL347_24825)	PA3592	+	Homology from 25780925
rpoS (AL347_24665)	rpoS	PA3591 (AL347_24830)	PA3591	+	Homology from 25780925
rpoS (AL347_24665)	rpoS	PA3590 (AL347_24835)	PA3590	+	Homology from 25780925
rpoS (AL347_24665)	rpoS	PA3589 (AL347_24840)	PA3589	+	Homology from 25780925
rpoS (AL347_24665)	rpoS	PA3588 (AL347_24845)	PA3588	+	Homology from 25780925
rpoS (AL347_24665)	rpoS	PA3582 (AL347_24880)	PA3582 (glpK)	+	Homology from 25780925
rpoS (AL347_24665)	rpoS	glpF (AL347_24885)	glpF	+	Homology from 25780925

rpoS (AL347_24665)	rpoS	PA3572 (AL347_24940)	PA3572	+	Homology from 25780925
rpoS (AL347_24665)	rpoS	bfrB (AL347_25155)	bfrB	+	Homology from 25780925
rpoS (AL347_24665)	rpoS	rhlR (AL347_25325)	rhlR	+	Homology from 25780925
rpoS (AL347_24665)	rpoS	PA3465 (AL347_25390)	PA3465	+	Homology from 25780925
rpoS (AL347_24665)	rpoS	PA3451 (AL347_25465)	PA3451	+	Homology from 25780925
rpoS (AL347_24665)	rpoS	PA3418 (AL347_25630)	PA3418 (ldh)	+	Homology from 25780925
rpoS (AL347_24665)	rpoS	hsbA (AL347_26015)	hsbA	+	Homology from 25780925
rpoS (AL347_24665)	rpoS	PA3346 (AL347_26020)	PA3346 (hsbR)	+	Homology from 25780925
rpoS (AL347_24665)	rpoS	PA3340 (AL347_26055)	PA3340	+	Homology from 25780925
rpoS (AL347_24665)	rpoS	clpP2 (AL347_26125)	clpP2	+	Homology from 25780925
rpoS (AL347_24665)	rpoS	PA3311 (AL347_26200)	PA3311 (nbdA)	+	Homology from 25780925
rpoS (AL347_24665)	rpoS	PA3305 (AL347_26235)	PA3305	+	Homology from 25780925
rpoS (AL347_24665)	rpoS	PA3289 (AL347_26330)	PA3289	+	Homology from 25780925
rpoS (AL347_24665)	rpoS	PA3261 (AL347_26495)	PA3261	+	Homology from 25780925
rpoS (AL347_24665)	rpoS	PA3254 (AL347_00250)	PA3254	+	Homology from 25780925
rpoS (AL347_24665)	rpoS	PA3253 (AL347_00255)	PA3253	+	Homology from 25780925
rpoS (AL347_24665)	rpoS	PA3252 (AL347_00260)	PA3252	+	Homology from 25780925
rpoS (AL347_24665)	rpoS	PA3251 (AL347_00265)	PA3251	+	Homology from 25780925
rpoS (AL347_24665)	rpoS	PA3250 (AL347_00270)	PA3250	+	Homology from 25780925
rpoS (AL347_24665)	rpoS	PA3179 (AL347_26925)	PA3179	+	Homology from 25780925
rpoS (AL347_24665)	rpoS	PA3032 (AL347_27690)	PA3032 (snr1)	+	Homology from 25780925
rpoS (AL347_24665)	rpoS	PA2939 (AL347_28180)	PA2939	+	Homology from 25780925
rpoS (AL347_24665)	rpoS	PA2933 (AL347_28215)	PA2933	+	Homology from 25780925
rpoS (AL347_24665)	rpoS	PA2851 (AL347_28635)	PA2851 (efp)	+	Homology from 25780925
rpoS (AL347_24665)	rpoS	PA2841 (AL347_28685)	PA2841	+	Homology from 25780925
rpoS (AL347_24665)	rpoS	PA2801 (AL347_28895)	PA2801	+	Homology from 25780925
rpoS (AL347_24665)	rpoS	PA2790 (AL347_29185)	PA2790	+	Homology from 25780925
rpoS (AL347_24665)	rpoS	PA2779 (AL347_29245)	PA2779	+	Homology from 25780925
rpoS (AL347_24665)	rpoS	PA2746 (AL347_29480)	PA2746	+	Homology from 25780925
rpoS (AL347_24665)	rpoS	PA2700 (AL347_29910)	PA2700 (opdB)	+	Homology from 25780925
rpoS (AL347_24665)	rpoS	PA2677 (AL347_30025)	PA2677	+	Homology from 25780925
rpoS (AL347_24665)	rpoS	PA2573 (AL347_31760)	PA2573	+	Homology from 25780925
rpoS (AL347_24665)	rpoS	PA2572 (AL347_31765)	PA2572	+	Homology from 25780925
rpoS (AL347_24665)	rpoS	PA2571 (AL347_31770)	PA2571	+	Homology from 25780925
rpoS (AL347_24665)	rpoS	PA2563 (AL347_31820)	PA2563	+	Homology from 25780925
rpoS (AL347_24665)	rpoS	PA2501 (AL347_32470)	PA2501	+	Homology from 25780925
rpoS (AL347_24665)	rpoS	PA2475 (AL347_32645)	PA2475	+	Homology from 25780925
rpoS (AL347_24665)	rpoS	PA2371 (AL347_33165)	PA2371 (clpV3)	+	Homology from 25780925

rpoS (AL347_24665)	rpoS	PA2370 (AL347_33170)	PA2370 (hsiH3)	+	Homology from 25780925
rpoS (AL347_24665)	rpoS	PA2369 (AL347_33175)	PA2369 (hsiG3)	+	Homology from 25780925
rpoS (AL347_24665)	rpoS	PA2368 (AL347_33180)	PA2368 (hsiF3)	+	Homology from 25780925
rpoS (AL347_24665)	rpoS	PA2367 (AL347_33185)	PA2367 (hcp3)	+	Homology from 25780925
rpoS (AL347_24665)	rpoS	PA2366 (AL347_33190)	PA2366 (hsiC3, puuD)	+	Homology from 25780925
rpoS (AL347_24665)	rpoS	PA2365 (AL347_33195)	PA2365 (hsiB3)	+	Homology from 25780925
rpoS (AL347_24665)	rpoS	PA2364 (AL347_33200)	PA2364 (lip3)	+	Homology from 25780925
rpoS (AL347_24665)	rpoS	PA2363 (AL347_33205)	PA2363 (hsiJ3)	+	Homology from 25780925
rpoS (AL347_24665)	rpoS	PA2362 (AL347_33210)	PA2362 (dotU3)	+	Homology from 25780925
rpoS (AL347_24665)	rpoS	PA2361 (AL347_33215)	PA2361 (icmF3)	+	Homology from 25780925
rpoS (AL347_24665)	rpoS	PA2360 (AL347_33220)	PA2360 (hsiA3)	+	Homology from 25780925
rpoS (AL347_24665)	rpoS	bkdB (AL347_00820)	bkdB	+	Homology from 25780925
rpoS (AL347_24665)	rpoS	bkdA2 (AL347_00825)	bkdA2	+	Homology from 25780925
rpoS (AL347_24665)	rpoS	bkdA1 (AL347_00830)	bkdA1	+	Homology from 25780925
rpoS (AL347_24665)	rpoS	PA2076 (AL347_02210)	PA2076	+	Homology from 25780925
rpoS (AL347_24665)	rpoS	PA2072 (AL347_02240)	PA2072	+	Homology from 25780925
rpoS (AL347_24665)	rpoS	cmrA (AL347_02370)	cmrA	+	Homology from 25780925
rpoS (AL347_24665)	rpoS	PA2024 (AL347_02485)	PA2024	+	Homology from 25780925
rpoS (AL347_24665)	rpoS	PA1951 (AL347_02860)	PA1951 (fapF)	+	Homology from 25780925
rpoS (AL347_24665)	rpoS	PA1930 (AL347_02950)	PA1930	+	Homology from 25780925
rpoS (AL347_24665)	rpoS	PA1888 (AL347_03180)	PA1888	+	Homology from 25780925
rpoS (AL347_24665)	rpoS	PA1887 (AL347_03185)	PA1887	+	Homology from 25780925
rpoS (AL347_24665)	rpoS	PA1881 (AL347_03215)	PA1881	+	Homology from 25780925
rpoS (AL347_24665)	rpoS	PA1880 (AL347_03220)	PA1880	+	Homology from 25780925
rpoS (AL347_24665)	rpoS	bapB (AL347_03245)	bapB	+	Homology from 25780925
rpoS (AL347_24665)	rpoS	PA1860 (AL347_03325)	PA1860	+	Homology from 25780925
rpoS (AL347_24665)	rpoS	PA1784 (AL347_03760)	PA1784	+	Homology from 25780925
rpoS (AL347_24665)	rpoS	PA1745 (AL347_03970)	PA1745	+	Homology from 25780925
rpoS (AL347_24665)	rpoS	PA1728 (AL347_04060)	PA1728	+	Homology from 25780925
rpoS (AL347_24665)	rpoS	PA1590 (AL347_04775)	PA1590 (braB)	+	Homology from 25780925
rpoS (AL347_24665)	rpoS	PA1584 (AL347_04805)	PA1584 (sdhB)	+	Homology from 25780925
rpoS (AL347_24665)	rpoS	PA1583 (AL347_04810)	PA1583 (sdhA)	+	Homology from 25780925
rpoS (AL347_24665)	rpoS	PA1582 (AL347_04815)	PA1582 (sdhD)	+	Homology from 25780925
rpoS (AL347_24665)	rpoS	PA1581 (AL347_04820)	PA1581 (sdhC, cybA)	+	Homology from 25780925
rpoS (AL347_24665)	rpoS	PA1563 (AL347_04925)	PA1563	+	Homology from 25780925
rpoS (AL347_24665)	rpoS	PA1562 (AL347_04930)	PA1562 (acnA, acn)	+	Homology from 25780925
rpoS (AL347_24665)	rpoS	gbuR (AL347_05705)	gbuR	+	Homology from 25780925
rpoS (AL347_24665)	rpoS	PA1415 (AL347_05740)	PA1415	+	Homology from 25780925

rpoS (AL347_24665)	rpoS	PA1414 (AL347_05745)	PA1414	+	Homology from 25780925
rpoS (AL347_24665)	rpoS	PA1358 (AL347_06305)	PA1358	+	Homology from 25780925
rpoS (AL347_24665)	rpoS	PA1343 (AL347_06380)	PA1343	+	Homology from 25780925
rpoS (AL347_24665)	rpoS	PA1333 (AL347_06435)	PA1333	+	Homology from 25780925
rpoS (AL347_24665)	rpoS	PA1289 (AL347_06665)	PA1289	+	Homology from 25780925
rpoS (AL347_24665)	rpoS	oprH (AL347_07245)	oprH	+	Homology from 25780925
rpoS (AL347_24665)	rpoS	PA1177 (AL347_07250)	PA1177 (napE)	+	Homology from 25780925
rpoS (AL347_24665)	rpoS	PA1176 (AL347_07255)	PA1176 (napF)	+	Homology from 25780925
rpoS (AL347_24665)	rpoS	PA1175 (AL347_07260)	PA1175 (napD)	+	Homology from 25780925
rpoS (AL347_24665)	rpoS	PA1173 (AL347_07270)	PA1173 (napB)	+	Homology from 25780925
rpoS (AL347_24665)	rpoS	PA1172 (AL347_07275)	PA1172 (napC)	+	Homology from 25780925
rpoS (AL347_24665)	rpoS	PA1163 (AL347_07320)	PA1163 (ndvB)	+	Homology from 25780925
rpoS (AL347_24665)	rpoS	PA1121 (AL347_07555)	PA1121 (yfiR)	+	Homology from 25780925
rpoS (AL347_24665)	rpoS	tpbB (AL347_07560)	tpbB	+	Homology from 25780925
rpoS (AL347_24665)	rpoS	PA1119 (AL347_07565)	PA1119 (yfiB)	+	Homology from 25780925
rpoS (AL347_24665)	rpoS	PA1118 (AL347_07570)	PA1118	+	Homology from 25780925
rpoS (AL347_24665)	rpoS	PA1076 (AL347_07795)	PA1076	+	Homology from 25780925
rpoS (AL347_24665)	rpoS	PA1065 (AL347_07855)	PA1065	+	Homology from 25780925
rpoS (AL347_24665)	rpoS	PA1041 (AL347_07980)	PA1041	+	Homology from 25780925
rpoS (AL347_24665)	rpoS	PA1040 (AL347_07985)	PA1040	+	Homology from 25780925
rpoS (AL347_24665)	rpoS	PA1039 (AL347_07990)	PA1039	+	Homology from 25780925
rpoS (AL347_24665)	rpoS	PA1038 (AL347_07995)	PA1038	+	Homology from 25780925
rpoS (AL347_24665)	rpoS	PA1014 (AL347_08130)	PA1014 (wapB)	+	Homology from 25780925
rpoS (AL347_24665)	rpoS	PA0988 (AL347_08265)	PA0988	+	Homology from 25780925
rpoS (AL347_24665)	rpoS	PA0959 (AL347_08410)	PA0959	+	Homology from 25780925
rpoS (AL347_24665)	rpoS	PA0918 (AL347_08635)	PA0918	+	Homology from 25780925
rpoS (AL347_24665)	rpoS	rsmA (AL347_08970)	rsmA	+	Homology from 25780925
rpoS (AL347_24665)	rpoS	phhA (AL347_09145)	phhA	+	Homology from 25780925
rpoS (AL347_24665)	rpoS	PA0862 (AL347_09200)	PA0862	+	Homology from 25780925
rpoS (AL347_24665)	rpoS	PA0861 (AL347_09205)	PA0861 (rbdA)	+	Homology from 25780925
rpoS (AL347_24665)	rpoS	PA0837 (AL347_09325)	PA0837 (slyD)	+	Homology from 25780925
rpoS (AL347_24665)	rpoS	PA0833 (AL347_09350)	PA0833	+	Homology from 25780925
rpoS (AL347_24665)	rpoS	PA0818 (AL347_09420)	PA0818	+	Homology from 25780925
rpoS (AL347_24665)	rpoS	PA0788 (AL347_09575)	PA0788	+	Homology from 25780925
rpoS (AL347_24665)	rpoS	PA0773 (AL347_09650)	PA0773 (pdxJ)	+	Homology from 25780925
rpoS (AL347_24665)	rpoS	PA0772 (AL347_09655)	PA0772 (recO)	+	Homology from 25780925
rpoS (AL347_24665)	rpoS	PA0771 (AL347_09660)	PA0771 (era)	+	Homology from 25780925
rpoS (AL347_24665)	rpoS	PA0770 (AL347_09665)	PA0770 (rnc)	+	Homology from 25780925

rpoS (AL347_24665)	rpoS	PA0769 (AL347_09670)	PA0769	+	Homology from 25780925
rpoS (AL347_24665)	rpoS	PA0704 (AL347_10025)	PA0704	+	Homology from 25780925
rpoS (AL347_24665)	rpoS	PA0703 (AL347_10030)	PA0703	+	Homology from 25780925
rpoS (AL347_24665)	rpoS	PA0588 (AL347_20820)	PA0588	+	Homology from 25780925
rpoS (AL347_24665)	rpoS	PA0587 (AL347_20815)	PA0587	+	Homology from 25780925
rpoS (AL347_24665)	rpoS	PA0586 (AL347_20810)	PA0586	+	Homology from 25780925
rpoS (AL347_24665)	rpoS	PA0575 (AL347_20755)	PA0575 (rmcA)	+	Homology from 25780925
rpoS (AL347_24665)	rpoS	PA0563 (AL347_20655)	PA0563	+	Homology from 25780925
rpoS (AL347_24665)	rpoS	fadE (AL347_20360)	fadE	+	Homology from 25780925
rpoS (AL347_24665)	rpoS	PA0484 (AL347_20255)	PA0484	+	Homology from 25780925
rpoS (AL347_24665)	rpoS	PA0483 (AL347_20250)	PA0483	+	Homology from 25780925
rpoS (AL347_24665)	rpoS	PA0459 (AL347_20125)	PA0459	+	Homology from 25780925
rpoS (AL347_24665)	rpoS	PA0452 (AL347_20085)	PA0452	+	Homology from 25780925
rpoS (AL347_24665)	rpoS	PA0451 (AL347_20080)	PA0451	+	Homology from 25780925
rpoS (AL347_24665)	rpoS	dht (AL347_20030)	dht	+	Homology from 25780925
rpoS (AL347_24665)	rpoS	PA0440 (AL347_20025)	PA0440	+	Homology from 25780925
rpoS (AL347_24665)	rpoS	PA0439 (AL347_20020)	PA0439	+	Homology from 25780925
rpoS (AL347_24665)	rpoS	PA0423 (AL347_19935)	PA0423 (pasP, yceI)	+	Homology from 25780925
rpoS (AL347_24665)	rpoS	PA0422 (AL347_19930)	PA0422	+	Homology from 25780925
rpoS (AL347_24665)	rpoS	PA0397 (AL347_19800)	PA0397	+	Homology from 25780925
rpoS (AL347_24665)	rpoS	PA0315 (AL347_19370)	PA0315	+	Homology from 25780925
rpoS (AL347_24665)	rpoS	PA0312 (AL347_19355)	PA0312	+	Homology from 25780925
rpoS (AL347_24665)	rpoS	PA0179 (AL347_18680)	PA0179	+	Homology from 25780925
rpoS (AL347_24665)	rpoS	PA0178 (AL347_18675)	PA0178	+	Homology from 25780925
rpoS (AL347_24665)	rpoS	PA0177 (AL347_18670)	PA0177	+	Homology from 25780925
rpoS (AL347_24665)	rpoS	PA0175 (AL347_18660)	PA0175 (cheR2)	+	Homology from 25780925
rpoS (AL347_24665)	rpoS	PA0174 (AL347_18655)	PA0174	+	Homology from 25780925
rpoS (AL347_24665)	rpoS	PA0173 (AL347_18650)	PA0173	+	Homology from 25780925
rpoS (AL347_24665)	rpoS	PA0109 (AL347_18320)	PA0109	+	Homology from 25780925
rpoS (AL347_24665)	rpoS	PA0097 (AL347_18255)	PA0097	+	Homology from 25780925
rpoS (AL347_24665)	rpoS	PA0096 (AL347_18250)	PA0096	+	Homology from 25780925
rpoS (AL347_24665)	rpoS	PA0052 (AL347_18035)	PA0052	+	Homology from 25780925
rpoS (AL347_24665)	rpoS	PA0050 (AL347_18020)	PA0050	+	Homology from 25780925
rpoS (AL347_24665)	rpoS	PA0028 (AL347_17890)	PA0028	+	Homology from 25780925
rpoS (AL347_24665)	rpoS	PA0027 (AL347_17885)	PA0027	+	Homology from 25780925
rpoS (AL347_24665)	rpoS	plcB (AL347_17880)	plcB	+	Homology from 25780925
rpoS (AL347_24665)	rpoS	PA0016 (AL347_17830)	PA0016 (trkA)	+	Homology from 25780925
rpoS (AL347_24665)	rpoS	PA0004 (AL347_17745)	PA0004 (gyrB)	+	Homology from 25780925

rpoS (AL347_24665)	rpoS	PA0003 (AL347_17740)	PA0003 (recF)	+	Homology from 25780925
rpoS (AL347_24665)	rpoS	PA0002 (AL347_17735)	PA0002 (dnaN)	+	Homology from 25780925
rpoS (AL347_24665)	rpoS	dnaA (AL347_17730)	dnaA	+	Homology from 25780925
rpoS (AL347_24665)	rpoS	hcnA (AL347_01585)	hcnA	?	27242034*, 18974177*, 22587778
rpoS (AL347_24665)	rpoS	phzC1 (AL347_21615)	phzC1	?	27242034*, 18974177*, 22587778
rpoS (AL347_24665)	rpoS	phzF1 (AL347_21600)	phzF1	?	27242034*, 18974177*, 22587778
rpoS (AL347_24665)	rpoS	lasB (AL347_24155)	lasB	?	27242034*, 18974177*, 22587778
rpoS (AL347_24665)	rpoS	lecA (AL347_31780)	lecA	?	27242034*
rpoS (AL347_24665)	rpoS	aer2 (AL347_18665)	aer2	?	27242034*
rpoS (AL347_24665)	rpoS	phzF2 (AL347_03100)	phzF2	?	27242034*
rpoS (AL347_24665)	rpoS	phzD1 (AL347_21610)	phzD1	?	27242034*
rpoS (AL347_24665)	rpoS	phzB1 (AL347_21620)	phzB1	?	27242034*
rpoS (AL347_24665)	rpoS	phzG1 (AL347_21595)	phzG1	?	27242034*
rpoS (AL347_24665)	rpoS	hcnC (AL347_01575)	hcnC	?	27242034*
rpoS (AL347_24665)	rpoS	hcnB (AL347_01580)	hcnB	?	27242034*
rpoS (AL347_24665)	rpoS	cttP (AL347_18685)	cttP	?	27242034*
rpoS (AL347_24665)	rpoS	phzA1 (AL347_21625)	phzA1	?	27242034*
rpoS (AL347_24665)	rpoS	phzG2 (AL347_03095)	phzG2	?	27242034*
rpoS (AL347_24665)	rpoS	lasA (AL347_03265)	lasA	?	27242034*
rpoS (AL347_24665)	rpoS	phzE1 (AL347_21605)	phzE1	?	27242034*
rsaL (AL347_05610)	rsaL	triC (AL347_18575)	triC	?	31270321
rsaL (AL347_05610)	rsaL	cerN (AL347_09285)	cerN	?	31270321
rsaL (AL347_05610)	rsaL	PA1333 (AL347_06435)	PA1333	?	31270321
rsaL (AL347_05610)	rsaL	qsrO (AL347_33910)	qsrO	?	31270321
rsaL (AL347_05610)	rsaL	PA2228 (AL347_33900)	PA2228	?	31270321
rsaL (AL347_05610)	rsaL	pqsH (AL347_30495)	pqsH	?	31270321
rsaL (AL347_05610)	rsaL	acpP (AL347_28040)	acpP	?	31270321
rsaL (AL347_05610)	rsaL	gyrA (AL347_26980)	gyrA	?	31270321
rsaL (AL347_05610)	rsaL	PA3342 (AL347_26040)	PA3342	?	31270321
rsaL (AL347_05610)	rsaL	PA3638 (AL347_24585)	PA3638	?	31270321
rsaL (AL347_05610)	rsaL	PA3669 (AL347_24430)	PA3669	?	31270321
rsaL (AL347_05610)	rsaL	PA3722 (AL347_24165)	PA3722	?	31270321
rsaL (AL347_05610)	rsaL	PA3774 (AL347_23890)	PA3774	?	31270321
rsaL (AL347_05610)	rsaL	glyA3 (AL347_12505)	glyA3	?	31270321
rsaL (AL347_05610)	rsaL	PA4767 (AL347_13430)	PA4767	?	31270321
rsaL (AL347_05610)	rsaL	retS (AL347_13890)	retS	?	31270321
rsaL (AL347_05610)	rsaL	sphR (AL347_16375)	sphR	?	31270321
rsaL (AL347_05610)	rsaL	PA5517 (AL347_17405)	PA5517	?	31270321

rsaL (AL347_05610)	rsaL	lasI (AL347_05605)	lasI	-	27242034*, 18974177*, 22587778
rsaL (AL347_05610)	rsaL	phzA1 (AL347_21625)	phzA1	-	27242034*, 18974177*, 22587778
rsaL (AL347_05610)	rsaL	lasB (AL347_24155)	lasB	?	27242034*, 18974177*, 22587778
rsaL (AL347_05610)	rsaL	rsaL (AL347_05610)	rsaL	-	27242034*, 18974177*, 22587778
rsaL (AL347_05610)	rsaL	phzM (AL347_21635)	phzM	-	27242034*, 18974177*, 22587778
rsaL (AL347_05610)	rsaL	hcnA (AL347_01585)	hcnA	-	27242034*, 18974177*, 22587778
rsaL (AL347_05610)	rsaL	phzF1 (AL347_21600)	phzF1	?	27242034*
rsaL (AL347_05610)	rsaL	phzF2 (AL347_03100)	phzF2	?	27242034*
rsaL (AL347_05610)	rsaL	phzB1 (AL347_21620)	phzB1	?	27242034*
rsaL (AL347_05610)	rsaL	phzC1 (AL347_21615)	phzC1	?	27242034*
rsaL (AL347_05610)	rsaL	phzE1 (AL347_21605)	phzE1	?	27242034*
rsaL (AL347_05610)	rsaL	phzD1 (AL347_21610)	phzD1	?	27242034*
rsaL (AL347_05610)	rsaL	phzG1 (AL347_21595)	phzG1	?	27242034*
rsaL (AL347_05610)	rsaL	phzG2 (AL347_03095)	phzG2	?	27242034*
rsaL (AL347_05610)	rsaL	hcnC (AL347_01575)	hcnC	?	27242034*
rsaL (AL347_05610)	rsaL	hcnB (AL347_01580)	hcnB	?	27242034*
rsmA (AL347_08970)	rsmA	fiuI (AL347_20195)	fiuI	?	29729420
rsmA (AL347_08970)	rsmA	PA1300 (AL347_06610)	PA1300 (hxuI)	?	29729420
rsmA (AL347_08970)	rsmA	femI (AL347_03050)	femI	?	29729420
rsmA (AL347_08970)	rsmA	foxI (AL347_32680)	foxI	?	29729420
rsmA (AL347_08970)	rsmA	fecI (AL347_23230)	fecI	?	29729420
rsmA (AL347_08970)	rsmA	pvdS (AL347_32895)	pvdS	?	29729420, 29729420
sbrI (AL347_28405)	sbrI	PA0167 (AL347_18620)	PA0167	?	29729420
sbrI (AL347_28405)	sbrI	PA0839 (AL347_09315)	PA0839	?	29729420
sbrI (AL347_28405)	sbrI	muiA (AL347_05290)	muiA	?	29729420
sbrI (AL347_28405)	sbrI	bexR (AL347_32855)	bexR	?	29729420
sbrI (AL347_28405)	sbrI	sbrI (AL347_28405)	sbrI	+	19846594, 27242034*, 22587778
sbrR (AL347_28410)	sbrR	sbrI (AL347_28405)	sbrI	-	19846594, 27242034*, 22587778
sdsB1 (AL347_09825)	sdsB1	sdsB1 (AL347_09825)	sdsB1	+	31540990
sdsB1 (AL347_09825)	sdsB1	sdsA1 (AL347_09820)	sdsA1	+	31540990
sigX (AL347_03799)	sigX	aguR (AL347_19255)	aguR	?	29729420
sigX (AL347_03799)	sigX	dnr (AL347_20470)	dnr	?	29729420
sigX (AL347_03799)	sigX	vreR (AL347_10170)	vreR	?	29729420
sigX (AL347_03799)	sigX	ddaR (AL347_07150)	ddaR	?	29729420
sigX (AL347_03799)	sigX	fliA (AL347_05490)	fliA	?	29729420
sigX (AL347_03799)	sigX	anr (AL347_05030)	anr	?	29729420
sigX (AL347_03799)	sigX	kdpD (AL347_04540)	kdpD	?	29729420
sigX (AL347_03799)	sigX	PA2384 (AL347_33095)	PA2384	?	29729420

sigX (AL347_03799)	sigX	gacA (AL347_30505)	gacA	?	29729420
sigX (AL347_03799)	sigX	psrA (AL347_27830)	psrA	?	29729420
sigX (AL347_03799)	sigX	PA3225 (AL347_00395)	PA3225	?	29729420
sigX (AL347_03799)	sigX	amrZ (AL347_25805)	amrZ	?	29729420
sigX (AL347_03799)	sigX	PA3458 (AL347_25425)	PA3458	?	29729420
sigX (AL347_03799)	sigX	rhlR (AL347_25325)	rhlR	?	29729420
sigX (AL347_03799)	sigX	narL (AL347_23345)	narL	?	29729420
sigX (AL347_03799)	sigX	fecI (AL347_23230)	fecI	?	29729420
sigX (AL347_03799)	sigX	PA4132 (AL347_22035)	PA4132 (mpaR)	?	29729420
sigX (AL347_03799)	sigX	pprB (AL347_10315)	pprB	?	29729420
sigX (AL347_03799)	sigX	roxS (AL347_11335)	roxS	?	29729420
sigX (AL347_03799)	sigX	cbrB (AL347_13205)	cbrB	?	29729420
sigX (AL347_03799)	sigX	PA4781 (AL347_13505)	PA4781	?	29729420
sigX (AL347_03799)	sigX	yegQ (AL347_17005)	yegQ	?	29729420
sigX (AL347_03799)	sigX	PA5506 (AL347_17350)	PA5506	?	29729420
sigX (AL347_03799)	sigX	lasR (AL347_05615)	lasR	?	29729420, 29729420
sigX (AL347_03799)	sigX	PA5559 (AL347_17615)	PA5559 (atpE, papH, uncE)	+	Homology from 25780925
sigX (AL347_03799)	sigX	PA5558 (AL347_17610)	PA5558 (atpF, papF, uncF)	+	Homology from 25780926
sigX (AL347_03799)	sigX	PA5557 (AL347_17605)	PA5557 (atpH, papE, uncH)	+	Homology from 25780927
sigX (AL347_03799)	sigX	PA5556 (AL347_17600)	PA5556 (atpA, papA, uncA)	+	Homology from 25780928
sigX (AL347_03799)	sigX	PA5555 (AL347_17595)	PA5555 (atpG, papC, uncG)	+	Homology from 25780929
sigX (AL347_03799)	sigX	PA5554 (AL347_17590)	PA5554 (atpD, papB, uncD)	+	Homology from 25780930
sigX (AL347_03799)	sigX	PA5553 (AL347_17585)	PA5553 (atpC, papG, uncC)	+	Homology from 25780931
sigX (AL347_03799)	sigX	PA5546 (AL347_17550)	PA5546	+	Homology from 25780932
sigX (AL347_03799)	sigX	PA5519 (AL347_17415)	PA5519	+	Homology from 25780933
sigX (AL347_03799)	sigX	PA5518 (AL347_17410)	PA5518	+	Homology from 25780934
sigX (AL347_03799)	sigX	PA5517 (AL347_17405)	PA5517	+	Homology from 25780935
sigX (AL347_03799)	sigX	PA5510 (AL347_17370)	PA5510	+	Homology from 25780936
sigX (AL347_03799)	sigX	PA5508 (AL347_17360)	PA5508 (pauA7)	+	Homology from 25780937
sigX (AL347_03799)	sigX	PA5507 (AL347_17355)	PA5507	+	Homology from 25780938
sigX (AL347_03799)	sigX	PA5504 (AL347_17340)	PA5504	+	Homology from 25780939
sigX (AL347_03799)	sigX	nrdJb (AL347_17300)	nrdJb	+	Homology from 25780940
sigX (AL347_03799)	sigX	PA5464 (AL347_17130)	PA5464	+	Homology from 25780941
sigX (AL347_03799)	sigX	PA5463 (AL347_17125)	PA5463	+	Homology from 25780942
sigX (AL347_03799)	sigX	PA5462 (AL347_17120)	PA5462	+	Homology from 25780943
sigX (AL347_03799)	sigX	PA5461 (AL347_17115)	PA5461	+	Homology from 25780944
sigX (AL347_03799)	sigX	dgcA (AL347_16780)	dgcA	+	Homology from 25780945
sigX (AL347_03799)	sigX	PA5303 (AL347_16270)	PA5303	+	Homology from 25780946

sigX (AL347_03799)	sigX	PA5239 (AL347_15935)	PA5239 (rho)	+	Homology from 25780947
sigX (AL347_03799)	sigX	PA5220 (AL347_15835)	PA5220	+	Homology from 25780948
sigX (AL347_03799)	sigX	PA5217 (AL347_15820)	PA5217	+	Homology from 25780949
sigX (AL347_03799)	sigX	PA5174 (AL347_15595)	PA5174 (fabY)	+	Homology from 25780950
sigX (AL347_03799)	sigX	arcC (AL347_15590)	arcC	+	Homology from 25780951
sigX (AL347_03799)	sigX	arcB (AL347_15585)	arcB	+	Homology from 25780952
sigX (AL347_03799)	sigX	arcA (AL347_15580)	arcA	+	Homology from 25780953
sigX (AL347_03799)	sigX	PA5132 (AL347_15315)	PA5132	+	Homology from 25780954
sigX (AL347_03799)	sigX	pilN (AL347_14860)	pilN	+	Homology from 25780955
sigX (AL347_03799)	sigX	pilP (AL347_14850)	pilP	+	Homology from 25780956
sigX (AL347_03799)	sigX	aceE (AL347_14705)	aceE	+	Homology from 25780957
sigX (AL347_03799)	sigX	pncB1 (AL347_14205)	pncB1	+	Homology from 25780958
sigX (AL347_03799)	sigX	PA4899 (AL347_14105)	PA4899	+	Homology from 25780959
sigX (AL347_03799)	sigX	PA4881 (AL347_14015)	PA4881	+	Homology from 25780960
sigX (AL347_03799)	sigX	PA4849 (AL347_13855)	PA4849	+	Homology from 25780961
sigX (AL347_03799)	sigX	PA4848 (AL347_13850)	PA4848 (accC)	+	Homology from 25780962
sigX (AL347_03799)	sigX	PA4847 (AL347_13845)	PA4847 (accB, fabE)	+	Homology from 25780963
sigX (AL347_03799)	sigX	PA4846 (AL347_13840)	PA4846 (aroQ1, aroD1)	+	Homology from 25780964
sigX (AL347_03799)	sigX	PA4783 (AL347_13515)	PA4783	+	Homology from 25780965
sigX (AL347_03799)	sigX	PA4782 (AL347_13510)	PA4782	+	Homology from 25780966
sigX (AL347_03799)	sigX	PA4743 (AL347_13295)	PA4743 (rbfA)	+	Homology from 25780967
sigX (AL347_03799)	sigX	PA4742 (AL347_13290)	PA4742 (truB)	+	Homology from 25780968
sigX (AL347_03799)	sigX	phuS (AL347_13115)	phuS	+	Homology from 25780969
sigX (AL347_03799)	sigX	phuT (AL347_13110)	phuT	+	Homology from 25780970
sigX (AL347_03799)	sigX	phuU (AL347_13105)	phuU	+	Homology from 25780971
sigX (AL347_03799)	sigX	PA4706 (AL347_13100)	PA4706 (phuV)	+	Homology from 25780972
sigX (AL347_03799)	sigX	PA4705 (AL347_13095)	PA4705 (phuW)	+	Homology from 25780973
sigX (AL347_03799)	sigX	PA4657 (AL347_12805)	PA4657	+	Homology from 25780974
sigX (AL347_03799)	sigX	PA4624 (AL347_12630)	PA4624 (cdrB)	+	Homology from 25780975
sigX (AL347_03799)	sigX	PA4620 (AL347_12605)	PA4620	+	Homology from 25780976
sigX (AL347_03799)	sigX	PA4456 (AL347_11145)	PA4456	+	Homology from 25780977
sigX (AL347_03799)	sigX	PA4455 (AL347_11140)	PA4455	+	Homology from 25780978
sigX (AL347_03799)	sigX	PA4454 (AL347_11135)	PA4454	+	Homology from 25780979
sigX (AL347_03799)	sigX	PA4453 (AL347_11130)	PA4453	+	Homology from 25780980
sigX (AL347_03799)	sigX	PA4452 (AL347_11125)	PA4452	+	Homology from 25780981
sigX (AL347_03799)	sigX	PA4441 (AL347_11070)	PA4441	+	Homology from 25780982
sigX (AL347_03799)	sigX	PA4432 (AL347_11025)	PA4432 (rpsI)	+	Homology from 25780983
sigX (AL347_03799)	sigX	PA4419 (AL347_10955)	PA4419 (ftsL)	+	Homology from 25780984

sigX (AL347_03799)	sigX	pctC (AL347_10370)	pctC	+	Homology from 25780985
sigX (AL347_03799)	sigX	PA4276 (AL347_21290)	PA4276 (secE, prlG)	+	Homology from 25780986
sigX (AL347_03799)	sigX	rpsL (AL347_21330)	rpsL	+	Homology from 25780987
sigX (AL347_03799)	sigX	rpsJ (AL347_21350)	rpsJ	+	Homology from 25780988
sigX (AL347_03799)	sigX	rplC (AL347_21355)	rplC	+	Homology from 25780989
sigX (AL347_03799)	sigX	PA4262 (AL347_21360)	PA4262 (rplD)	+	Homology from 25780990
sigX (AL347_03799)	sigX	PA4261 (AL347_21365)	PA4261 (rplW)	+	Homology from 25780991
sigX (AL347_03799)	sigX	PA4260 (AL347_21370)	PA4260 (rplB)	+	Homology from 25780992
sigX (AL347_03799)	sigX	PA4259 (AL347_21375)	PA4259 (rpsS)	+	Homology from 25780993
sigX (AL347_03799)	sigX	PA4258 (AL347_21380)	PA4258 (rplV)	+	Homology from 25780994
sigX (AL347_03799)	sigX	PA4257 (AL347_21385)	PA4257 (rpsC)	+	Homology from 25780995
sigX (AL347_03799)	sigX	PA4256 (AL347_21390)	PA4256 (rplP)	+	Homology from 25780996
sigX (AL347_03799)	sigX	PA4255 (AL347_21395)	PA4255 (rpmC)	+	Homology from 25780997
sigX (AL347_03799)	sigX	PA4254 (AL347_21400)	PA4254 (rpsQ)	+	Homology from 25780998
sigX (AL347_03799)	sigX	PA4253 (AL347_21405)	PA4253 (rplN)	+	Homology from 25780999
sigX (AL347_03799)	sigX	PA4252 (AL347_21410)	PA4252 (rplX)	+	Homology from 25781000
sigX (AL347_03799)	sigX	PA4251 (AL347_21415)	PA4251 (rplE)	+	Homology from 25781001
sigX (AL347_03799)	sigX	PA4243 (AL347_21455)	PA4243 (secY, prlA)	+	Homology from 25781002
sigX (AL347_03799)	sigX	PA4238 (AL347_21480)	PA4238 (rpoA)	+	Homology from 25781003
sigX (AL347_03799)	sigX	phzB1 (AL347_21620)	phzB1	+	Homology from 25781004
sigX (AL347_03799)	sigX	mexI (AL347_21645)	mexI	+	Homology from 25781005
sigX (AL347_03799)	sigX	mexH (AL347_21650)	mexH	+	Homology from 25781006
sigX (AL347_03799)	sigX	PA4131 (AL347_22040)	PA4131	+	Homology from 25781007
sigX (AL347_03799)	sigX	cupB2 (AL347_22280)	cupB2	+	Homology from 25781008
sigX (AL347_03799)	sigX	PA4067 (AL347_22375)	PA4067 (oprG, yciD, ompW)	+	Homology from 25781009
sigX (AL347_03799)	sigX	lis (AL347_22730)	lis	+	Homology from 25781010
sigX (AL347_03799)	sigX	PA3972 (AL347_22845)	PA3972	+	Homology from 25781011
sigX (AL347_03799)	sigX	PA3912 (AL347_23165)	PA3912	+	Homology from 25781012
sigX (AL347_03799)	sigX	nark1 (AL347_23355)	nark1	+	Homology from 25781013
sigX (AL347_03799)	sigX	nark2 (AL347_23360)	nark2	+	Homology from 25781014
sigX (AL347_03799)	sigX	narG (AL347_23365)	narG	+	Homology from 25781015
sigX (AL347_03799)	sigX	narH (AL347_23370)	narH	+	Homology from 25781016
sigX (AL347_03799)	sigX	narJ (AL347_23375)	narJ	+	Homology from 25781017
sigX (AL347_03799)	sigX	narI (AL347_23380)	narI	+	Homology from 25781018
sigX (AL347_03799)	sigX	PA3871 (AL347_23385)	PA3871	+	Homology from 25781019
sigX (AL347_03799)	sigX	PA3870 (AL347_23390)	PA3870 (moaA1)	+	Homology from 25781020
sigX (AL347_03799)	sigX	PA3822 (AL347_23645)	PA3822	+	Homology from 25781021
sigX (AL347_03799)	sigX	PA3819 (AL347_23660)	PA3819	+	Homology from 25781022

sigX (AL347_03799)	sigX	fdx2 (AL347_23710)	fdx2	+	Homology from 25781023
sigX (AL347_03799)	sigX	iscX (AL347_23715)	iscX	+	Homology from 25781024
sigX (AL347_03799)	sigX	PA3799 (AL347_23760)	PA3799	+	Homology from 25781025
sigX (AL347_03799)	sigX	PA3762 (AL347_23960)	PA3762	+	Homology from 25781026
sigX (AL347_03799)	sigX	PA3700 (AL347_24275)	PA3700 (lysS)	+	Homology from 25781027
sigX (AL347_03799)	sigX	PA3645 (AL347_24550)	PA3645 (fabZ, sefA)	+	Homology from 25781028
sigX (AL347_03799)	sigX	PA3644 (AL347_24555)	PA3644 (lpxA)	+	Homology from 25781029
sigX (AL347_03799)	sigX	PA3643 (AL347_24560)	PA3643 (lpxB, pgsB)	+	Homology from 25781030
sigX (AL347_03799)	sigX	PA3642 (AL347_24565)	PA3642 (rnhB)	+	Homology from 25781031
sigX (AL347_03799)	sigX	accA (AL347_24580)	accA	+	Homology from 25781032
sigX (AL347_03799)	sigX	PA3638 (AL347_24585)	PA3638	+	Homology from 25781033
sigX (AL347_03799)	sigX	PA3609 (AL347_24735)	PA3609 (potC)	+	Homology from 25781034
sigX (AL347_03799)	sigX	PA3595 (AL347_24805)	PA3595	+	Homology from 25781035
sigX (AL347_03799)	sigX	algF (AL347_25055)	algF	+	Homology from 25781036
sigX (AL347_03799)	sigX	algJ (AL347_25060)	algJ	+	Homology from 25781037
sigX (AL347_03799)	sigX	PA3517 (AL347_25225)	PA3517	+	Homology from 25781038
sigX (AL347_03799)	sigX	rhlA (AL347_25315)	rhlA	+	Homology from 25781039
sigX (AL347_03799)	sigX	rhlB (AL347_25320)	rhlB	+	Homology from 25781040
sigX (AL347_03799)	sigX	PA3461 (AL347_25410)	PA3461	+	Homology from 25781041
sigX (AL347_03799)	sigX	PA3460 (AL347_25415)	PA3460	+	Homology from 25781042
sigX (AL347_03799)	sigX	PA3459 (AL347_25420)	PA3459	+	Homology from 25781043
sigX (AL347_03799)	sigX	PA3336 (AL347_26075)	PA3336	+	Homology from 25781044
sigX (AL347_03799)	sigX	PA3334 (AL347_26085)	PA3334 (acp3)	+	Homology from 25781045
sigX (AL347_03799)	sigX	fabH2 (AL347_26090)	fabH2	+	Homology from 25781046
sigX (AL347_03799)	sigX	PA3332 (AL347_26095)	PA3332	+	Homology from 25781047
sigX (AL347_03799)	sigX	PA3331 (AL347_26100)	PA3331	+	Homology from 25781048
sigX (AL347_03799)	sigX	PA3330 (AL347_26105)	PA3330	+	Homology from 25781049
sigX (AL347_03799)	sigX	PA3329 (AL347_26110)	PA3329	+	Homology from 25781050
sigX (AL347_03799)	sigX	PA3328 (AL347_26115)	PA3328	+	Homology from 25781051
sigX (AL347_03799)	sigX	PA3327 (AL347_26120)	PA3327	+	Homology from 25781052
sigX (AL347_03799)	sigX	clpP2 (AL347_26125)	clpP2	+	Homology from 25781053
sigX (AL347_03799)	sigX	PA3286 (AL347_00060)	PA3286	+	Homology from 25781054
sigX (AL347_03799)	sigX	PA3284 (AL347_26360)	PA3284	+	Homology from 25781055
sigX (AL347_03799)	sigX	PA3283 (AL347_00080)	PA3283	+	Homology from 25781056
sigX (AL347_03799)	sigX	PA3282 (AL347_00085)	PA3282	+	Homology from 25781057
sigX (AL347_03799)	sigX	PA3281 (AL347_26375)	PA3281	+	Homology from 25781058
sigX (AL347_03799)	sigX	PA3267 (AL347_00165)	PA3267	+	Homology from 25781059
sigX (AL347_03799)	sigX	PA3162 (AL347_27010)	PA3162 (rpsA)	+	Homology from 25781060

sigX (AL347_03799)	sigX	PA3139 (AL347_27125)	PA3139	+	Homology from 25781061
sigX (AL347_03799)	sigX	PA3111 (AL347_27285)	PA3111 (folC)	+	Homology from 25781062
sigX (AL347_03799)	sigX	PA3110 (AL347_27290)	PA3110	+	Homology from 25781063
sigX (AL347_03799)	sigX	PA3109 (AL347_27295)	PA3109	+	Homology from 25781064
sigX (AL347_03799)	sigX	PA3081 (AL347_27455)	PA3081	+	Homology from 25781065
sigX (AL347_03799)	sigX	PA3032 (AL347_27690)	PA3032 (snr1)	+	Homology from 25781066
sigX (AL347_03799)	sigX	PA3012 (AL347_27800)	PA3012	+	Homology from 25781067
sigX (AL347_03799)	sigX	PA3000 (AL347_27860)	PA3000 (aroP1)	+	Homology from 25781068
sigX (AL347_03799)	sigX	PA2968 (AL347_28030)	PA2968 (fabD)	+	Homology from 25781069
sigX (AL347_03799)	sigX	PA2950 (AL347_28120)	PA2950 (fabV, pfm)	+	Homology from 25781070
sigX (AL347_03799)	sigX	PA2777 (AL347_29255)	PA2777	+	Homology from 25781071
sigX (AL347_03799)	sigX	PA2658 (AL347_30125)	PA2658	+	Homology from 25781072
sigX (AL347_03799)	sigX	PA2566 (AL347_31805)	PA2566	+	Homology from 25781073
sigX (AL347_03799)	sigX	PA2565 (AL347_31810)	PA2565	+	Homology from 25781074
sigX (AL347_03799)	sigX	PA2564 (AL347_31815)	PA2564	+	Homology from 25781075
sigX (AL347_03799)	sigX	PA2560 (AL347_31835)	PA2560	+	Homology from 25781076
sigX (AL347_03799)	sigX	PA2541 (AL347_31940)	PA2541	+	Homology from 25781077
sigX (AL347_03799)	sigX	ptrC (AL347_32590)	ptrC	+	Homology from 25781078
sigX (AL347_03799)	sigX	PA2485 (AL347_32595)	PA2485	+	Homology from 25781079
sigX (AL347_03799)	sigX	foxA (AL347_32690)	foxA	+	Homology from 25781080
sigX (AL347_03799)	sigX	PA2462 (AL347_32710)	PA2462	+	Homology from 25781081
sigX (AL347_03799)	sigX	pvdL (AL347_32905)	pvdL	+	Homology from 25781082
sigX (AL347_03799)	sigX	PA2410 (AL347_32975)	PA2410 (fpvF)	+	Homology from 25781083
sigX (AL347_03799)	sigX	PA2409 (AL347_32980)	PA2409 (fpvE)	+	Homology from 25781084
sigX (AL347_03799)	sigX	PA2406 (AL347_32995)	PA2406 (fpvK)	+	Homology from 25781085
sigX (AL347_03799)	sigX	PA2405 (AL347_33000)	PA2405 (fpvJ)	+	Homology from 25781086
sigX (AL347_03799)	sigX	PA2404 (AL347_33005)	PA2404 (fpvH)	+	Homology from 25781087
sigX (AL347_03799)	sigX	PA2318 (AL347_33435)	PA2318	+	Homology from 25781088
sigX (AL347_03799)	sigX	PA2317 (AL347_33440)	PA2317	+	Homology from 25781089
sigX (AL347_03799)	sigX	PA2215 (AL347_01475)	PA2215 (lyxD)	+	Homology from 25781090
sigX (AL347_03799)	sigX	PA2212 (AL347_01490)	PA2212	+	Homology from 25781091
sigX (AL347_03799)	sigX	PA2211 (AL347_01495)	PA2211	+	Homology from 25781092
sigX (AL347_03799)	sigX	hcnB (AL347_01580)	hcnB	+	Homology from 25781093
sigX (AL347_03799)	sigX	PA2072 (AL347_02240)	PA2072	+	Homology from 25781094
sigX (AL347_03799)	sigX	PA1942 (AL347_02905)	PA1942	+	Homology from 25781095
sigX (AL347_03799)	sigX	hvn (AL347_03040)	hvn	+	Homology from 25781096
sigX (AL347_03799)	sigX	phzG2 (AL347_03095)	phzG2	+	Homology from 25781097
sigX (AL347_03799)	sigX	phzB2 (AL347_03120)	phzB2	+	Homology from 25781098

sigX (AL347_03799)	sigX	PA1852 (AL347_03365)	PA1852	+	Homology from 25781099
sigX (AL347_03799)	sigX	PA1848 (AL347_03390)	PA1848	+	Homology from 25781100
sigX (AL347_03799)	sigX	cmpX (AL347_03805)	cmpX	+	Homology from 25781101
sigX (AL347_03799)	sigX	PA1774 (AL347_03810)	PA1774 (crfX)	+	Homology from 25781102
sigX (AL347_03799)	sigX	pscL (AL347_04075)	pscL	+	Homology from 25781103
sigX (AL347_03799)	sigX	pscK (AL347_04080)	pscK	+	Homology from 25781104
sigX (AL347_03799)	sigX	pscJ (AL347_04085)	pscJ	+	Homology from 25781105
sigX (AL347_03799)	sigX	pscI (AL347_04090)	pscI	+	Homology from 25781106
sigX (AL347_03799)	sigX	pscH (AL347_04095)	pscH	+	Homology from 25781107
sigX (AL347_03799)	sigX	pscG (AL347_04100)	pscG	+	Homology from 25781108
sigX (AL347_03799)	sigX	pscF (AL347_04105)	pscF	+	Homology from 25781109
sigX (AL347_03799)	sigX	pscE (AL347_04110)	pscE	+	Homology from 25781110
sigX (AL347_03799)	sigX	pscD (AL347_04115)	pscD	+	Homology from 25781111
sigX (AL347_03799)	sigX	pscC (AL347_04120)	pscC	+	Homology from 25781112
sigX (AL347_03799)	sigX	pscB (AL347_04125)	pscB	+	Homology from 25781113
sigX (AL347_03799)	sigX	exsD (AL347_04130)	exsD	+	Homology from 25781114
sigX (AL347_03799)	sigX	exsB (AL347_04140)	exsB	+	Homology from 25781115
sigX (AL347_03799)	sigX	exsC (AL347_04150)	exsC	+	Homology from 25781116
sigX (AL347_03799)	sigX	PA1708 (AL347_04160)	PA1708 (popB, pepB)	+	Homology from 25781117
sigX (AL347_03799)	sigX	PA1689 (AL347_04255)	PA1689	+	Homology from 25781118
sigX (AL347_03799)	sigX	PA1662 (AL347_04395)	PA1662 (clpV2)	+	Homology from 25781119
sigX (AL347_03799)	sigX	hsiF2 (AL347_04415)	hsiF2	+	Homology from 25781120
sigX (AL347_03799)	sigX	hsiC2 (AL347_04420)	hsiC2	+	Homology from 25781121
sigX (AL347_03799)	sigX	PA1634 (AL347_04550)	PA1634 (kdpB, atkB)	+	Homology from 25781122
sigX (AL347_03799)	sigX	PA1612 (AL347_04665)	PA1612	+	Homology from 25781123
sigX (AL347_03799)	sigX	fabA (AL347_04675)	fabA	+	Homology from 25781124
sigX (AL347_03799)	sigX	PA1609 (AL347_04680)	PA1609 (fabB)	+	Homology from 25781125
sigX (AL347_03799)	sigX	aer (AL347_04935)	aer	+	Homology from 25781126
sigX (AL347_03799)	sigX	ccoP2 (AL347_04965)	ccoP2	+	Homology from 25781127
sigX (AL347_03799)	sigX	PA1530 (AL347_05105)	PA1530	+	Homology from 25781128
sigX (AL347_03799)	sigX	PA1500 (AL347_05260)	PA1500	+	Homology from 25781129
sigX (AL347_03799)	sigX	PA1499 (AL347_05265)	PA1499	+	Homology from 25781130
sigX (AL347_03799)	sigX	PA1498 (AL347_05270)	PA1498 (pykF, pyk-I)	+	Homology from 25781131
sigX (AL347_03799)	sigX	lasI (AL347_05605)	lasI	+	Homology from 25781132
sigX (AL347_03799)	sigX	PA1208 (AL347_07090)	PA1208	+	Homology from 25781133
sigX (AL347_03799)	sigX	PA1207 (AL347_07095)	PA1207 (kefB, kefC, trkB)	+	Homology from 25781134
sigX (AL347_03799)	sigX	PA1181 (AL347_07230)	PA1181	+	Homology from 25781135
sigX (AL347_03799)	sigX	PA1096 (AL347_07695)	PA1096	+	Homology from 25781136

sigX (AL347_03799)	sigX	PA1093 (AL347_07710)	PA1093	+	Homology from 25781137
sigX (AL347_03799)	sigX	fliC (AL347_07715)	fliC	+	Homology from 25781138
sigX (AL347_03799)	sigX	slyB (AL347_07915)	slyB	+	Homology from 25781139
sigX (AL347_03799)	sigX	PA1007 (AL347_08170)	PA1007	+	Homology from 25781140
sigX (AL347_03799)	sigX	PA1006 (AL347_08175)	PA1006	+	Homology from 25781141
sigX (AL347_03799)	sigX	pqsA (AL347_08225)	pqsA	+	Homology from 25781142
sigX (AL347_03799)	sigX	tolB (AL347_08345)	tolB	+	Homology from 25781143
sigX (AL347_03799)	sigX	tolA (AL347_08350)	tolA	+	Homology from 25781144
sigX (AL347_03799)	sigX	tolR (AL347_08355)	tolR	+	Homology from 25781145
sigX (AL347_03799)	sigX	tolQ (AL347_08360)	tolQ	+	Homology from 25781146
sigX (AL347_03799)	sigX	PA0968 (AL347_08365)	PA0968	+	Homology from 25781147
sigX (AL347_03799)	sigX	PA0954 (AL347_08435)	PA0954	+	Homology from 25781148
sigX (AL347_03799)	sigX	PA0953 (AL347_08440)	PA0953	+	Homology from 25781149
sigX (AL347_03799)	sigX	PA0951 (AL347_08455)	PA0951	+	Homology from 25781150
sigX (AL347_03799)	sigX	PA0834 (AL347_09345)	PA0834	+	Homology from 25781151
sigX (AL347_03799)	sigX	PA0798 (AL347_09520)	PA0798 (pmtA)	+	Homology from 25781152
sigX (AL347_03799)	sigX	PA0697 (AL347_10065)	PA0697	+	Homology from 25781153
sigX (AL347_03799)	sigX	phdA (AL347_10095)	phdA	+	Homology from 25781154
sigX (AL347_03799)	sigX	PA0690 (AL347_10100)	PA0690 (pdtA)	+	Homology from 25781155
sigX (AL347_03799)	sigX	tyrZ (AL347_21220)	tyrZ	+	Homology from 25781156
sigX (AL347_03799)	sigX	nirS (AL347_20430)	nirS	+	Homology from 25781157
sigX (AL347_03799)	sigX	nirN (AL347_20380)	nirN	+	Homology from 25781158
sigX (AL347_03799)	sigX	bioD (AL347_20350)	bioD	+	Homology from 25781159
sigX (AL347_03799)	sigX	PA0503 (AL347_20345)	PA0503	+	Homology from 25781160
sigX (AL347_03799)	sigX	PA0502 (AL347_20340)	PA0502	+	Homology from 25781161
sigX (AL347_03799)	sigX	bioF (AL347_20335)	bioF	+	Homology from 25781162
sigX (AL347_03799)	sigX	bioB (AL347_20330)	bioB	+	Homology from 25781163
sigX (AL347_03799)	sigX	PA0469 (AL347_20180)	PA0469	+	Homology from 25781164
sigX (AL347_03799)	sigX	PA0454 (AL347_20095)	PA0454	+	Homology from 25781165
sigX (AL347_03799)	sigX	oprM (AL347_19955)	oprM	+	Homology from 25781166
sigX (AL347_03799)	sigX	mexB (AL347_19950)	mexB	+	Homology from 25781167
sigX (AL347_03799)	sigX	PA0420 (AL347_19915)	PA0420 (bioA)	+	Homology from 25781168
sigX (AL347_03799)	sigX	PA0419 (AL347_19910)	PA0419	+	Homology from 25781169
sigX (AL347_03799)	sigX	PA0418 (AL347_19905)	PA0418	+	Homology from 25781170
sigX (AL347_03799)	sigX	PA0359 (AL347_19600)	PA0359	+	Homology from 25781171
sigX (AL347_03799)	sigX	PA0358 (AL347_19595)	PA0358	+	Homology from 25781172
sigX (AL347_03799)	sigX	PA0346 (AL347_19535)	PA0346	+	Homology from 25781173
sigX (AL347_03799)	sigX	PA0320 (AL347_19395)	PA0320 (carO, ygiW)	+	Homology from 25781174

sigX (AL347_03799)	sigX	PA0256 (AL347_19055)	PA0256	+	Homology from 25781175
sigX (AL347_03799)	sigX	PA0196 (AL347_18775)	PA0196 (pntB)	+	Homology from 25781176
sigX (AL347_03799)	sigX	PA0195.1 (AL347_18770)	PA0195.1 (pntAB)	+	Homology from 25781177
sigX (AL347_03799)	sigX	PA0162 (AL347_18595)	PA0162 (opdC)	+	Homology from 25781178
sigX (AL347_03799)	sigX	coIII (AL347_18315)	coIII	+	Homology from 25781179
sigX (AL347_03799)	sigX	PA0107 (AL347_18310)	PA0107	+	Homology from 25781180
sigX (AL347_03799)	sigX	coxA (AL347_18305)	coxA	+	Homology from 25781181
sigX (AL347_03799)	sigX	coxB (AL347_18300)	coxB	+	Homology from 25781182
sigX (AL347_03799)	sigX	PA0102 (AL347_18280)	PA0102	+	Homology from 25781183
sigX (AL347_03799)	sigX	PA0046 (AL347_18000)	PA0046	+	Homology from 25781184
sigX (AL347_03799)	sigX	PA0009 (AL347_17795)	PA0009 (glyQ)	+	Homology from 25781185
sigX (AL347_03799)	sigX	PA0008 (AL347_17790)	PA0008 (glyS)	+	Homology from 25781186
sigX (AL347_03799)	sigX	PA0006 (AL347_17780)	PA0006	+	Homology from 25781187
sigX (AL347_03799)	sigX	lptA (AL347_17775)	lptA	+	Homology from 25781188
sigX (AL347_03799)	sigX	PA2881 (AL347_28480)	PA2881	+	Homology from 25780925
sigX (AL347_03799)	sigX	PA2692 (AL347_29950)	PA2692	+	Homology from 25780926
sigX (AL347_03799)	sigX	sigX (AL347_03799)	sigX	?	Homology from 25780927
sigX (AL347_03799)	sigX	exsA (AL347_04135)	exsA	+	Homology from 25780928
sigX (AL347_03799)	sigX	exsE (AL347_04145)	exsE	+	Homology from 25780929
sigX (AL347_03799)	sigX	PA1611 (AL347_04670)	PA1611	+	Homology from 25780930
sigX (AL347_03799)	sigX	PA0942 (AL347_08505)	PA0942	+	Homology from 25780931
sigX (AL347_03799)	sigX	PA0515 (AL347_20410)	PA0515	+	Homology from 25780932
sigX (AL347_03799)	sigX	pobR (AL347_19010)	pobR	+	Homology from 25780933
sigX (AL347_03799)	sigX	PA3285 (AL347_00065)	PA3285	+	Homology from 25780934
soxR (AL347_00695)	soxR	PA2274 (AL347_00690)	PA2274	+	15632300
soxR (AL347_00695)	soxR	PA0344 (AL347_19525)	PA0344	?	31270321
soxR (AL347_00695)	soxR	PA0570 (AL347_20690)	PA0570	?	31270321
soxR (AL347_00695)	soxR	cat (AL347_10015)	cat	?	31270321
soxR (AL347_00695)	soxR	PA0874 (AL347_09130)	PA0874	?	31270321
soxR (AL347_00695)	soxR	lysC (AL347_08975)	lysC	?	31270321
soxR (AL347_00695)	soxR	PA1012 (AL347_08145)	PA1012	?	31270321
soxR (AL347_00695)	soxR	PA1197 (AL347_07145)	PA1197	?	31270321
soxR (AL347_00695)	soxR	PA1200 (AL347_07130)	PA1200	?	31270321
soxR (AL347_00695)	soxR	PA2004 (AL347_02590)	PA2004	?	31270321
soxR (AL347_00695)	soxR	lpdV (AL347_00815)	lpdV	?	31270321
soxR (AL347_00695)	soxR	PA2502 (AL347_32465)	PA2502	?	31270321
soxR (AL347_00695)	soxR	acpP (AL347_28040)	acpP	?	31270321
soxR (AL347_00695)	soxR	PA3328 (AL347_26115)	PA3328	?	31270321

soxR (AL347_00695)	soxR	amiR (AL347_25935)	amiR	?	31270321
soxR (AL347_00695)	soxR	amiC (AL347_25930)	amiC	?	31270321
soxR (AL347_00695)	soxR	rhlR (AL347_25325)	rhlR	?	31270321
soxR (AL347_00695)	soxR	PA3592 (AL347_24825)	PA3592	?	31270321
soxR (AL347_00695)	soxR	pyrG (AL347_24590)	pyrG	?	31270321
soxR (AL347_00695)	soxR	glnD (AL347_24485)	glnD	?	31270321
soxR (AL347_00695)	soxR	PA3759 (AL347_23975)	PA3759	?	31270321
soxR (AL347_00695)	soxR	nagE (AL347_23965)	nagE	?	31270321
soxR (AL347_00695)	soxR	PA3892 (AL347_23270)	PA3892	?	31270321
soxR (AL347_00695)	soxR	ppgL (AL347_21660)	ppgL	?	31270321
soxR (AL347_00695)	soxR	mexG (AL347_21655)	mexG	?	31270321
soxR (AL347_00695)	soxR	mexH (AL347_21650)	mexH	?	31270321
soxR (AL347_00695)	soxR	mexI (AL347_21645)	mexI	?	31270321
soxR (AL347_00695)	soxR	PA4289 (AL347_10280)	PA4289	?	31270321
soxR (AL347_00695)	soxR	PA4291 (AL347_10290)	PA4291	?	31270321
soxR (AL347_00695)	soxR	PA4340 (AL347_10545)	PA4340	?	31270321
soxR (AL347_00695)	soxR	PA4510 (AL347_11420)	PA4510	?	31270321
soxR (AL347_00695)	soxR	PA4570 (AL347_12335)	PA4570	?	31270321
soxR (AL347_00695)	soxR	PA4586 (AL347_12420)	PA4586	?	31270321
soxR (AL347_00695)	soxR	PA4676 (AL347_12920)	PA4676	?	31270321
soxR (AL347_00695)	soxR	PA4685 (AL347_12965)	PA4685	?	31270321
soxR (AL347_00695)	soxR	PA4735 (AL347_13250)	PA4735	?	31270321
soxR (AL347_00695)	soxR	PA4987 (AL347_14565)	PA4987	?	31270321
soxR (AL347_00695)	soxR	PA3718 (AL347_24185)	PA3718	+	27242034*, 18974177*, 22587778
soxR (AL347_00695)	soxR	opmD (AL347_21640)	opmD	+	27242034*, 18974177*, 22587778
soxR (AL347_00695)	soxR	soxR (AL347_00695)	soxR	+	27242034*, 18974177*, 22587778
sphR (AL347_16375)	sphR	dnaA (AL347_17730)	dnaA	?	31270321
sphR (AL347_16375)	sphR	PA0042 (AL347_17975)	PA0042	?	31270321
sphR (AL347_16375)	sphR	phzH (AL347_18030)	phzH	?	31270321
sphR (AL347_16375)	sphR	PA0643 (AL347_21095)	PA0643	?	31270321
sphR (AL347_16375)	sphR	tyrZ (AL347_21220)	tyrZ	?	31270321
sphR (AL347_16375)	sphR	phdA (AL347_10095)	phdA	?	31270321
sphR (AL347_16375)	sphR	PA0718 (AL347_09950)	PA0718	?	31270321
sphR (AL347_16375)	sphR	cerN (AL347_09285)	cerN	?	31270321
sphR (AL347_16375)	sphR	PA0874 (AL347_09130)	PA0874	?	31270321
sphR (AL347_16375)	sphR	PA0959 (AL347_08410)	PA0959	?	31270321
sphR (AL347_16375)	sphR	PA0981 (AL347_08300)	PA0981	?	31270321
sphR (AL347_16375)	sphR	PA0984 (AL347_08280)	PA0984	?	31270321

sphR (AL347_16375)	sphR	cupC3 (AL347_08235)	cupC3	?	31270321
sphR (AL347_16375)	sphR	imm2 (AL347_07400)	imm2	?	31270321
sphR (AL347_16375)	sphR	aprA (AL347_06875)	aprA	?	31270321
sphR (AL347_16375)	sphR	PA1383 (AL347_06185)	PA1383	?	31270321
sphR (AL347_16375)	sphR	hsiA2 (AL347_04430)	hsiA2	?	31270321
sphR (AL347_16375)	sphR	qscR (AL347_03130)	qscR	?	31270321
sphR (AL347_16375)	sphR	PA2229 (AL347_00925)	PA2229	?	31270321
sphR (AL347_16375)	sphR	vgrG3 (AL347_33150)	vgrG3	?	31270321
sphR (AL347_16375)	sphR	PA2569 (AL347_31785)	PA2569	?	31270321
sphR (AL347_16375)	sphR	nuoA (AL347_30235)	nuoA	?	31270321
sphR (AL347_16375)	sphR	PA2698 (AL347_29920)	PA2698	?	31270321
sphR (AL347_16375)	sphR	PA2793 (AL347_29170)	PA2793	?	31270321
sphR (AL347_16375)	sphR	PA2795 (AL347_28925)	PA2795 (dusA, yjbN)	?	31270321
sphR (AL347_16375)	sphR	oprI (AL347_28620)	oprI	?	31270321
sphR (AL347_16375)	sphR	acpP (AL347_28040)	acpP	?	31270321
sphR (AL347_16375)	sphR	rocsS2 (AL347_27630)	rocsS2	?	31270321
sphR (AL347_16375)	sphR	rocA2 (AL347_27625)	rocA2	?	31270321
sphR (AL347_16375)	sphR	PA3047 (AL347_27615)	PA3047	?	31270321
sphR (AL347_16375)	sphR	fimV (AL347_27265)	fimV	?	31270321
sphR (AL347_16375)	sphR	wbpM (AL347_27110)	wbpM	?	31270321
sphR (AL347_16375)	sphR	wbpJ (AL347_27090)	wbpJ	?	31270321
sphR (AL347_16375)	sphR	hisF2 (AL347_27070)	hisF2	?	31270321
sphR (AL347_16375)	sphR	hisH2 (AL347_27065)	hisH2	?	31270321
sphR (AL347_16375)	sphR	wbpE (AL347_27050)	wbpE	?	31270321
sphR (AL347_16375)	sphR	wbpA (AL347_27030)	wbpA	?	31270321
sphR (AL347_16375)	sphR	wzz (AL347_27025)	wzz	?	31270321
sphR (AL347_16375)	sphR	gyrA (AL347_26980)	gyrA	?	31270321
sphR (AL347_16375)	sphR	PA3190 (AL347_00580)	PA3190	?	31270321
sphR (AL347_16375)	sphR	oprP (AL347_26390)	oprP	?	31270321
sphR (AL347_16375)	sphR	PA3309 (AL347_26210)	PA3309	?	31270321
sphR (AL347_16375)	sphR	PA3327 (AL347_26120)	PA3327	?	31270321
sphR (AL347_16375)	sphR	amrZ (AL347_25805)	amrZ	?	31270321
sphR (AL347_16375)	sphR	PA3489 (AL347_25275)	PA3489	?	31270321
sphR (AL347_16375)	sphR	PA3519 (AL347_25215)	PA3519	?	31270321
sphR (AL347_16375)	sphR	PA3520 (AL347_25210)	PA3520	?	31270321
sphR (AL347_16375)	sphR	bfrB (AL347_25155)	bfrB	?	31270321
sphR (AL347_16375)	sphR	algD (AL347_25110)	algD	?	31270321
sphR (AL347_16375)	sphR	arnB (AL347_25045)	arnB	?	31270321

sphR (AL347_16375)	sphR	PA3835 (AL347_23570)	PA3835	?	31270321
sphR (AL347_16375)	sphR	PA4080 (AL347_22305)	PA4080	?	31270321
sphR (AL347_16375)	sphR	cupB3 (AL347_22285)	cupB3	?	31270321
sphR (AL347_16375)	sphR	PA4087 (AL347_22270)	PA4087	?	31270321
sphR (AL347_16375)	sphR	PA4139 (AL347_21995)	PA4139	?	31270321
sphR (AL347_16375)	sphR	nmoA (AL347_21670)	nmoA	?	31270321
sphR (AL347_16375)	sphR	phzA1 (AL347_21625)	phzA1	?	31270321
sphR (AL347_16375)	sphR	phzB1 (AL347_21620)	phzB1	?	31270321
sphR (AL347_16375)	sphR	phzC1 (AL347_21615)	phzC1	?	31270321
sphR (AL347_16375)	sphR	phzD1 (AL347_21610)	phzD1	?	31270321
sphR (AL347_16375)	sphR	phzE1 (AL347_21605)	phzE1	?	31270321
sphR (AL347_16375)	sphR	phzF1 (AL347_21600)	phzF1	?	31270321
sphR (AL347_16375)	sphR	rplC (AL347_21355)	rplC	?	31270321
sphR (AL347_16375)	sphR	rpsG (AL347_21335)	rpsG	?	31270321
sphR (AL347_16375)	sphR	rpoC (AL347_21325)	rpoC	?	31270321
sphR (AL347_16375)	sphR	flp (AL347_10365)	flp	?	31270321
sphR (AL347_16375)	sphR	PA4523 (AL347_11485)	PA4523	?	31270321
sphR (AL347_16375)	sphR	gcbA (AL347_13825)	gcbA	?	31270321
sphR (AL347_16375)	sphR	ponA (AL347_14870)	ponA	?	31270321
sphR (AL347_16375)	sphR	PA5184 (AL347_15650)	PA5184	?	31270321
sphR (AL347_16375)	sphR	sphR (AL347_16375)	sphR	?	31270321
sphR (AL347_16375)	sphR	PA5446 (AL347_17040)	PA5446	?	31270321
sphR (AL347_16375)	sphR	PA5546 (AL347_17550)	PA5546	?	31270321
sphR (AL347_16375)	sphR	atpI (AL347_17625)	atpI	?	31270321
toxR (AL347_10010)	toxR	tolQ (AL347_08360)	tolQ	+	18974177
toxR (AL347_10010)	toxR	tolR (AL347_08355)	tolR	+	18974177
toxR (AL347_10010)	toxR	tolA (AL347_08350)	tolA	+	18974177
toxR (AL347_10010)	toxR	tolB (AL347_08345)	tolB	+	18974177
toxR (AL347_10010)	toxR	oprL (AL347_08340)	oprL	+	18974177
toxR (AL347_10010)	toxR	PA3842 (AL347_23525)	PA3842 (spcS, orf1)	+	18974177
toxR (AL347_10010)	toxR	toxA (AL347_07415)	toxA	+	8930909, 7959069
toxR (AL347_10010)	toxR	motD (AL347_05460)	motD	+	27242034*, 18974177*, 22587778
tpbA (AL347_23315)	tpbA	PA4139 (AL347_21995)	PA4139	-	27242034*, 18974177*, 22587778
tpbA (AL347_23315)	tpbA	PA4624 (AL347_12630)	PA4624 (cdrB)	-	27242034*, 18974177*, 22587778
tpbA (AL347_23315)	tpbA	PA4625 (AL347_12636)	PA4625 (cdrA)	-	27242034*, 18974177*, 22587778
tpbA (AL347_23315)	tpbA	pelA (AL347_27525)	pelA	-	27242034*, 18974177*, 22587778
tpbA (AL347_23315)	tpbA	pelB (AL347_27530)	pelB	-	27242034*, 18974177*, 22587778
tpbA (AL347_23315)	tpbA	pelC (AL347_27535)	pelC	-	27242034*, 18974177*, 22587778

tpbA (AL347_23315)	tpbA	pelD (AL347_27540)	pelD	-	27242034*, 18974177*, 22587778
tpbA (AL347_23315)	tpbA	pelE (AL347_27545)	pelE	-	27242034*, 18974177*, 22587778
tpbA (AL347_23315)	tpbA	pelF (AL347_27550)	pelF	-	27242034*, 18974177*, 22587778
tpbA (AL347_23315)	tpbA	pelG (AL347_27555)	pelG	-	27242034*, 18974177*, 22587778
tpbA (AL347_23315)	tpbA	tpbA (AL347_23315)	tpbA	-	27242034*, 18974177*, 22587778
tpbA (AL347_23315)	tpbA	tpbB (AL347_07560)	tpbB	-	27242034*, 18974177*, 22587778
trpI (AL347_17940)	trpI	trpA (AL347_17930)	trpA	+	9858743
trpI (AL347_17940)	trpI	trpB (AL347_17935)	trpB	+	9858743
trpI (AL347_17940)	trpI	trpI (AL347_17940)	trpI	-	27242034*
vfr (AL347_21140)	vfr	toxR (AL347_10010)	toxR	+	17159200
vfr (AL347_21140)	vfr	toxA (AL347_07415)	toxA	+	17159200
vfr (AL347_21140)	vfr	cpdA (AL347_14470)	cpdA	+	17159200
vfr (AL347_21140)	vfr	ptxR (AL347_33740)	ptxR	+	18227247
vfr (AL347_21140)	vfr	fleQ (AL347_07690)	fleQ	-	18974177
vfr (AL347_21140)	vfr	cbpA (AL347_13090)	cbpA	+	19801409
vfr (AL347_21140)	vfr	vfr (AL347_21140)	vfr	+	20494996
vfr (AL347_21140)	vfr	lasR (AL347_05615)	lasR	+	21719541
vfr (AL347_21140)	vfr	rhlR (AL347_25325)	rhlR	+	21719541
vfr (AL347_21140)	vfr	amrZ (AL347_25805)	amrZ	+	25488298
vfr (AL347_21140)	vfr	algR (AL347_16050)	algR	+	25488298
vfr (AL347_21140)	vfr	exsA (AL347_04135)	exsA	+	26929300
vfr (AL347_21140)	vfr	pilP (AL347_14850)	pilP	?	27242034*
vfr (AL347_21140)	vfr	plcH (AL347_09290)	plcH	?	27242034*
vfr (AL347_21140)	vfr	PA0653 (AL347_21145)	PA0653	?	27242034*
vfr (AL347_21140)	vfr	plcN (AL347_26160)	plcN	?	27242034*
vfr (AL347_21140)	vfr	plcR (AL347_09295)	plcR	?	27242034*
vfr (AL347_21140)	vfr	pilM (AL347_14865)	pilM	?	27242034*
vfr (AL347_21140)	vfr	pilO (AL347_14855)	pilO	?	27242034*
vfr (AL347_21140)	vfr	pilN (AL347_14860)	pilN	?	27242034*
vfr (AL347_21140)	vfr	alg8 (AL347_25105)	alg8	?	27242034*
vfr (AL347_21140)	vfr	algZ (AL347_16055)	algZ	?	27242034*
vfr (AL347_21140)	vfr	alg44 (AL347_25100)	alg44	?	27242034*
vfr (AL347_21140)	vfr	algJ (AL347_25060)	algJ	?	27242034*
vfr (AL347_21140)	vfr	algK (AL347_25095)	algK	?	27242034*
vfr (AL347_21140)	vfr	algI (AL347_25065)	algI	?	27242034*
vfr (AL347_21140)	vfr	algL (AL347_25075)	algL	?	27242034*
vfr (AL347_21140)	vfr	lasI (AL347_05605)	lasI	?	27242034*
vfr (AL347_21140)	vfr	algA (AL347_25050)	algA	?	27242034*

vfr (AL347_21140)	vfr	algF (AL347_25055)	algF	?	27242034*
vfr (AL347_21140)	vfr	algG (AL347_25085)	algG	?	27242034*
vfr (AL347_21140)	vfr	algD (AL347_25110)	algD	?	27242034*
vfr (AL347_21140)	vfr	algE (AL347_25090)	algE	?	27242034*
vfr (AL347_21140)	vfr	exoT (AL347_17990)	exoT	?	27242034*
vfr (AL347_21140)	vfr	argH (AL347_16060)	argH	?	27242034*
vfr (AL347_21140)	vfr	pbpG (AL347_09160)	pbpG	?	27242034*
vfr (AL347_21140)	vfr	algX (AL347_25080)	algX	?	27242034*
vqsM (AL347_33905)	vqsM	vqsR (AL347_30475)	vqsR	+	16194239, 27242034*, 18974177*
vqsM (AL347_33905)	vqsM	rpoS (AL347_24665)	rpoS	+	16194239
vqsM (AL347_33905)	vqsM	pprB (AL347_10315)	pprB	+	16194239
vqsM (AL347_33905)	vqsM	PA0007 (AL347_17785)	PA0007	+	25034696
vqsM (AL347_33905)	vqsM	PA0022 (AL347_17860)	PA0022	+	25034696
vqsM (AL347_33905)	vqsM	PA0401 (AL347_19820)	PA0401	+	25034696
vqsM (AL347_33905)	vqsM	PA0440 (AL347_20025)	PA0440	+	25034696
vqsM (AL347_33905)	vqsM	norD (AL347_20460)	norD	+	25034696
vqsM (AL347_33905)	vqsM	PA0526 (AL347_20465)	PA0526	+	25034696
vqsM (AL347_33905)	vqsM	PA1253 (AL347_06855)	PA1253 (lhpG)	+	25034696
vqsM (AL347_33905)	vqsM	cobV (AL347_06705)	cobV	+	25034696
vqsM (AL347_33905)	vqsM	hbcR (AL347_02585)	hbcR	+	25034696
vqsM (AL347_33905)	vqsM	vqsM (AL347_33905)	vqsM	+	25034696
vqsM (AL347_33905)	vqsM	pvdQ (AL347_33090)	pvdQ	+	25034696
vqsM (AL347_33905)	vqsM	cdpR (AL347_30490)	cdpR	+	25034696
vqsM (AL347_33905)	vqsM	cysG (AL347_30370)	cysG	+	25034696
vqsM (AL347_33905)	vqsM	PA2661 (AL347_30110)	PA2661	+	25034696
vqsM (AL347_33905)	vqsM	PA3106 (AL347_27310)	PA3106	+	25034696
vqsM (AL347_33905)	vqsM	trkH (AL347_00470)	trkH	+	25034696
vqsM (AL347_33905)	vqsM	nosD (AL347_25765)	nosD	+	25034696
vqsM (AL347_33905)	vqsM	PA3565 (AL347_24975)	PA3565	+	25034696
vqsM (AL347_33905)	vqsM	accA (AL347_24580)	accA	+	25034696
vqsM (AL347_33905)	vqsM	PA3648 (AL347_24535)	PA3648 (opr86)	+	25034696
vqsM (AL347_33905)	vqsM	PA3670 (AL347_24425)	PA3670	+	25034696
vqsM (AL347_33905)	vqsM	spdH (AL347_24210)	spdH	+	25034696
vqsM (AL347_33905)	vqsM	hscB (AL347_23700)	hscB	+	25034696
vqsM (AL347_33905)	vqsM	fecR (AL347_23225)	fecR	+	25034696
vqsM (AL347_33905)	vqsM	tseT (AL347_23190)	tseT	+	25034696
vqsM (AL347_33905)	vqsM	PA3925 (AL347_23090)	PA3925	+	25034696
vqsM (AL347_33905)	vqsM	phuU (AL347_13105)	phuU	+	25034696

vqsM (AL347_33905)	vqsM	smpB (AL347_13435)	smpB	+	25034696
vqsM (AL347_33905)	vqsM	purD (AL347_13885)	purD	+	25034696
vqsM (AL347_33905)	vqsM	PA4894 (AL347_14080)	PA4894	+	25034696
vqsM (AL347_33905)	vqsM	PA4908 (AL347_14150)	PA4908	+	25034696
vqsM (AL347_33905)	vqsM	waaC (AL347_14685)	waaC	+	25034696
vqsM (AL347_33905)	vqsM	gcvP1 (AL347_15800)	gcvP1	+	25034696
vqsM (AL347_33905)	vqsM	PA5216 (AL347_15815)	PA5216	+	25034696
vqsM (AL347_33905)	vqsM	ppK (AL347_15950)	ppK	+	25034696
vqsM (AL347_33905)	vqsM	PA5290 (AL347_16205)	PA5290	+	25034696
vqsM (AL347_33905)	vqsM	sphR (AL347_16375)	sphR	+	25034696
vqsM (AL347_33905)	vqsM	recG (AL347_16480)	recG	+	25034696
vqsM (AL347_33905)	vqsM	PA5423 (AL347_16905)	PA5423	+	25034696
vqsM (AL347_33905)	vqsM	PA5518 (AL347_17410)	PA5518	+	25034696
vqsM (AL347_33905)	vqsM	PA1333 (AL347_06435)	PA1333	?	31270321
vqsM (AL347_33905)	vqsM	PA2004 (AL347_02590)	PA2004	?	31270321
vqsM (AL347_33905)	vqsM	pqsH (AL347_30495)	pqsH	?	31270321
vqsM (AL347_33905)	vqsM	PA3457 (AL347_25430)	PA3457	?	31270321
vqsM (AL347_33905)	vqsM	PA3638 (AL347_24585)	PA3638	?	31270321
vqsM (AL347_33905)	vqsM	mexK (AL347_24395)	mexK	?	31270321
vqsM (AL347_33905)	vqsM	fecI (AL347_23230)	fecI	?	31270321
vqsM (AL347_33905)	vqsM	PA4767 (AL347_13430)	PA4767	?	31270321
vqsM (AL347_33905)	vqsM	ctpL (AL347_13830)	ctpL	?	31270321
vqsM (AL347_33905)	vqsM	retS (AL347_13890)	retS	?	31270321
vqsM (AL347_33905)	vqsM	PA4925 (AL347_14235)	PA4925	?	31270321
vqsM (AL347_33905)	vqsM	PA5342 (AL347_16465)	PA5342	?	31270321
vqsM (AL347_33905)	vqsM	PA5517 (AL347_17405)	PA5517	?	31270321
vqsM (AL347_33905)	vqsM	cerN (AL347_09285)	cerN	+	25034696, 31270321
vqsM (AL347_33905)	vqsM	PA2228 (AL347_33900)	PA2228	+	25034696, 31270321
vqsM (AL347_33905)	vqsM	PA3342 (AL347_26040)	PA3342	+	25034696, 31270321
vqsM (AL347_33905)	vqsM	PA3749 (AL347_24025)	PA3749	+	25034696, 31270321
vqsM (AL347_33905)	vqsM	recD (AL347_10250)	recD	+	25034696, 31270321
vqsR (AL347_30475)	vqsR	PA0149 (AL347_18525)	PA0149	?	29729420
vqsR (AL347_30475)	vqsR	fiuI (AL347_20195)	fiuI	?	29729420
vqsR (AL347_30475)	vqsR	femI (AL347_03050)	femI	?	29729420
vqsR (AL347_30475)	vqsR	foxI (AL347_32680)	foxI	?	29729420
vqsR (AL347_30475)	vqsR	fecI (AL347_23230)	fecI	?	29729420
vqsR (AL347_30475)	vqsR	PA4896 (AL347_14090)	PA4896	?	29729420
vqsR (AL347_30475)	vqsR	PA0839 (AL347_09315)	PA0839	?	31270321

vqsR (AL347_30475)	vqsR	alc (AL347_05185)	alc	?	31270321
vqsR (AL347_30475)	vqsR	qscR (AL347_03130)	qscR	?	31270321
vqsR (AL347_30475)	vqsR	greB (AL347_28590)	greB	?	31270321
vqsR (AL347_30475)	vqsR	asd (AL347_27255)	asd	?	31270321
vqsR (AL347_30475)	vqsR	PA3471 (AL347_25360)	PA3471	?	31270321
vqsR (AL347_30475)	vqsR	PA3973 (AL347_22840)	PA3973	?	31270321
vqsR (AL347_30475)	vqsR	mexG (AL347_21655)	mexG	?	31270321
vqsR (AL347_30475)	vqsR	mexH (AL347_21650)	mexH	?	31270321
vqsR (AL347_30475)	vqsR	mexI (AL347_21645)	mexI	?	31270321
vqsR (AL347_30475)	vqsR	PA4312 (AL347_10400)	PA4312	?	31270321
vqsR (AL347_30475)	vqsR	PA4320 (AL347_10445)	PA4320	?	31270321
vqsR (AL347_30475)	vqsR	PA4357 (AL347_10630)	PA4357	?	31270321
vqsR (AL347_30475)	vqsR	speD2 (AL347_13460)	speD2	?	31270321
vreI (AL347_10175)	vreI	PA0149 (AL347_18525)	PA0149	?	29729420
vreI (AL347_10175)	vreI	vreR (AL347_10170)	vreR	?	29729420
vreI (AL347_10175)	vreI	PA2384 (AL347_33095)	PA2384	?	29729420
vreI (AL347_10175)	vreI	PA5403 (AL347_16805)	PA5403	?	29729420
vreI (AL347_10175)	vreI	phdA (AL347_10095)	phdA	+	32081993
vreI (AL347_10175)	vreI	tpsB (AL347_32705)	tpsB	+	27242034*
